# Identification and expression profiling of Pht1 phosphate transporters in wheat in controlled environments and in the field

**DOI:** 10.1111/plb.12668

**Published:** 2017-12-19

**Authors:** A. Grün, P. Buchner, M. R. Broadley, M. J. Hawkesford

**Affiliations:** ^1^ Plant Science Department Rothamsted Research Harpenden UK; ^2^ Plant and Crop Science Division School of Biosciences University of Nottingham Loughborough UK

**Keywords:** Deficiency, nutrition, phosphate, transport, wheat

## Abstract

- Phosphorus (P) is an important macronutrient with critical functions in plants. Phosphate (Pi) transporters, which mediate Pi acquisition and Pi translocation within the plant, are key factors in Pi deficiency responses. However, their relevance for adaptation to long‐term Pi limitation under agronomic conditions, particularly in wheat, remains unknown.
- Here, we describe the identification of the complete Pi transporter gene family (*Pht1*) in wheat (*Triticum aestivum*). Gene expression profiles were compared for hydroponic and field‐grown plant tissues of wheat at multiple development stages. *Cis*‐element analysis of selected Pht1 promoter regions was performed.
- A broad range of expression patterns of individual *TaPht1* genes was observed in relation to tissue specificity and the nutrient supply in the soil or in liquid culture, as well as an influence of the experimental system.
- The expression patterns indicate the involvement of specific transporters in Pi uptake, and in Pi transport and remobilisation within the plant, at different growth developmental stages. Specifically, the influence of Pi nutrition indicates a complex regulatory pattern of *TaPht1* gene transcript abundances as a response to low Pi availability in different culture systems, correlating with the existence of different *cis*‐acting promoter elements.

## Introduction

Inorganic phosphate (Pi) acquisition in plants is mediated by plasma membrane‐localised phosphate transporter (Pht) proteins, which belong to the major facilitator superfamily (MFS), and function as Pi/H^+^ symporters within high‐ and low‐affinity ranges (Rae *et al*. 2003; Raghothama 2005; Liu *et al*. 2011; Nussaume *et al*. 2011). The transporter genes have been classified into four families (*Pht1–4*) with distinct molecular structures, localisation and functions (Smith *et al*. 1999; Liu *et al*. 2011; Guo *et al*. 2013; Shukla *et al*. 2016). The transporters are involved in initial root Pi acquisition as well as Pi translocation throughout the plant. Some *Pht1* transporters are expressed in the root, predominantly in root tips and root hairs (Schünmann *et al*. 2004). Such root‐expressed *Pht1* transporters have been identified in a broad range of different plant species including wheat, indicating an involvement in initial root Pi uptake from the soil solution (Liu *et al*. 1998; Smith *et al*. 1999; Davies *et al*. 2002; Mudge *et al*. 2002; Nagy *et al*. 2006; Wang *et al*. 2013). The establishment of arbuscular mycorrhiza (AM) symbiosis is a well‐known adaptation strategy of plants to increase Pi accessibility in low Pi environments (Tarafdar & Marschner 1994; Koide & Kabir 2000), and the expression pattern of some *Pht1* transporters is closely related to root AM colonisation in rice, wheat, *Brachypodium* and *Medicago* (Harrison *et al*. 2002; Paszkowski *et al*. 2002; Glassop *et al*. 2005; Hong *et al*. 2012).

In addition to initial root Pi uptake, the large diversity of expression profiles in plant tissues indicates that Pht1 transporters are also involved in Pi translocation as well as Pi remobilisation, in the aerial parts of the plant, especially during generative growth. In barley, weak expression of root‐expressed *HvPht1;2* and *HvPht1;1* has been reported in leaves (Schünmann *et al*. 2004), whereas *HvPht1;6* was strongly expressed in both lower stem leaves and in flag leaves. In rice, *OsPht1;1* was expressed abundantly in epidermal root cells and in stele cells of leaves, and weakly in spikelets and emerging buds (Sun *et al*. 2012). *Pht1* transporter expression was observed in panicles and flag leaves of rice (Liu *et al*. 2011) and in flowers of soybean (Qin *et al*. 2012).

Phosphate starvation responses influence root Pi uptake mechanisms, as well as Pi partitioning between roots and aerial tissues, *via* altered *Pht1* transporter expression. Increased *Pht1* transporter expression was reported in plants during a short period of Pi starvation or upon mycorrhizal infection (Smith *et al*. 1999; Rae *et al*. 2003; Calderón‐Vázquez *et al*. 2008; Ai *et al*. 2009; Miao *et al*. 2009; Huang *et al*. 2011; Qin *et al*. 2012). In Pi‐starved maize plants, *Pht* expression is present in roots and leaves, and one of those *Pht*s, ZmPht1;6, is expressed in roots independent of the Pi supply, which were colonised by symbiotic mycorrhizal fungi. In the leaves ZmPht1;6 is induced under Pi depletion (Nagy *et al*. 2006). In rice, the expression of the low affinity Pht2 transporter is regulated by a MYB‐domain transcription factor, OsPHR2, influencing P translocation from root to shoot (Zhou *et al*. 2008). Furthermore, the rice high‐affinity OsPT8 transporter plays important roles in both the acquisition of Pi from the external environment, and in the translocation of Pi within plants (Jia *et al*. 2011). Overexpression of the MYB transcription factor PHR1 in wheat led to increased Pht1.2 and Pht1.6 expression, with a promotion of shoot development and root branching as well as increased grain yield (Wang *et al*. 2013).

The *Pht1* family members in wheat (*TaPht1*) may be important targets for enhancing low P tolerance and P acquisition efficiency/P utilisation efficiency in agronomic systems, however the lack of wheat genome sequence information has hindered investigation of this approach (Davies *et al*. 2002; Huang *et al*. 2011). The aim of this study was to identify all putative *TaPht1* family members using genomic sequence information and homologies with model plants and other cereal species, and to compare the wheat *Pht1* expression profiles in relation to P nutrition under controlled environment hydroponic cultures with expression patterns observed in field‐grown wheat plants.

## Material and methods

### Identification of wheat TaPht1 genes and phylogenetic analysis

Putative members of the *Pht1* gene family in *Triticum aestivum* were identified based on *Pht1* gene sequences from barley (*HvPht1*; Rae *et al*. 2003), *Brachypodium distachyon* (*BradiPht1*; Hong *et al*. 2012) and rice (*OsPht1*; Paszkowski *et al*. 2002) by BLAST analysis with a cut‐off threshold of 0.0001–0.01 (Altschul *et al*. 1990) using available wheat genome databases on the EnsemblePlant (Kersey *et al*. 2016), PLAZA3.0 (Proost *et al*. 2015) and the Rothamsted decypher web interface platforms [wheat databases: IWGSC (2014), TGAC (TGACv1 genome assembly of *Triticum aestivum* cv. Chinese Spring, generated by The Genome Analysis Centre, Norwich, as part of the BBSRC‐funded project, Triticeae Genomics for Sustainable Agriculture) and other wheat varieties (opendata.earlham.ac.uk/Triticum_aestivum/EI/v1 http://www.earlham.ac.uk/sequencing-wheat-genome)].

The transcription start sites, including the 5′‐non‐coding regions, could be verified from published sequence information data only for *TaPht1;2* on chromosome 4BL, and for *TaPht1;4* on chromosome 5BL (Kawaura *et al*. 2009). The ATG start sites of the remaining TaPht1s were verified by alignment analysis of DNA as well as amino acid sequences. There may be additional introns in the 5′‐non‐coding regions, which were ignored in this analysis (see Supplemental genomic sequences). Putative *TaPht1* sequences from genomic databases were allocated by multiple sequence alignment to those identified in this study (Table S5). The sequence similarities and phylogenetic relationships of the wheat *homoeologous Pht1s* to *Brachypodium*, rice (*Oryza sativa* ssp. japonica, MonocotPLAZA 3.0 gene locus names; Proost *et al*. 2015) and barley *Pht1s* were analysed based on the coding DNA as well as the protein sequences. Multiple sequence alignments were generated using ClustalX version 2.0 (Larkin *et al*. 2007). MEGA6 (version 6.06; Tamura *et al*. 2013) was used for the calculation of phylogenetic trees (the neighbour‐joining method; Saitou & Nei 1987). The bootstrap consensus tree (expressed as percentages) inferred from 1000 replicates was taken to represent the evolutionary relationships (Felsenstein 1985). The evolutionary distances were computed for both cDNA and amino acid sequences using the p‐distance method (Nei & Kumar 2000). Due to the inconsistent numbering of the *Pht1* transporters in different plant genomes, the *TaPht1* genes were, in part, classified according to the barley nomenclature, when putative homologues could be identified.

**Table 1:**
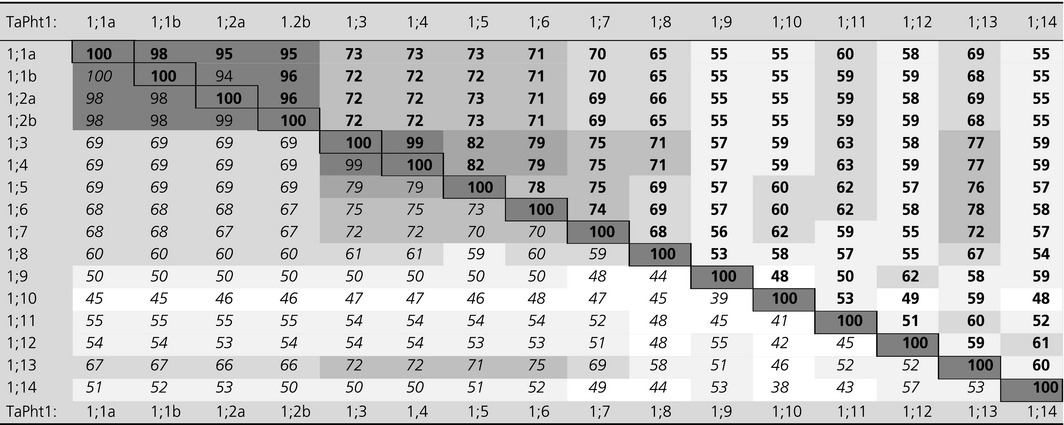
Homology (%) of translated transcripts of A‐genome phosphate transporters (with the exception of TaPht1;12 from the B‐genome) in wheat. The data were generated with the ClustalX version 2.0 software for alignments used for phylogenetic analysis. Nucleotide sequence identities (bold) and protein sequence identities (italics). The degree of shading highlights the level of sequence identities

### Identification of putative transcription factor binding sites in the promoter region of wheat Pht1 genes

In addition to the coding regions, 5′‐non‐coding and promoter regions up to 2000 bases upstream from the ATG start codons of all wheat *Pht1* genes were identified from the genomic databases (Supplemental genomic sequences).

The locations of the putative MYB transcription factor PHR1 promoter element binding site P1BS (GNATATNC), the WRKY transcription factor binding W‐box (C/T TGAC C/T) site and the bHLH transcription factor binding helix‐loop‐helix promoter element (CA[G/T][C/A]TG; Rubio *et al*. 2001; Zhou *et al*. 2008; Lin *et al*. 2009; Wang *et al*. 2014) in the promoter/5′‐non‐coding regions were identified by pair‐wise nucleotide alignment (Table S4, EMBOSS Needle‐Needleman‐Wunsch alignment algorithm to find the optimum alignment (including gaps) of two sequences along their entire length; McWilliam *et al*. 2013; Li *et al*. 2015).

### Plant material

For hydroponic experiments in controlled environments, seeds of *T. aestivum* cv. Hereward were surface‐sterilised for 10 min in a 1% perchlorate solution, rinsed with sterile water and germinated for 5 days on sterile water‐soaked paper tissue. Seedlings were transferred to a single 1‐l aerated hydroponic culture pot (one plant per pot) in a controlled environment chamber (228 Fitotron growth cabinet; SANYO‐Gallenkamp, UK): 12‐h day length, 70% humidity, 20 °C, photon flux rate 500 μmol photons m^−2^ s^−1^ (40 28W/20 49W fluorescent tubes in an air‐cooled light box); night conditions were 16 °C and 80% humidity. Light intensity was measured with a SKP 200 light meter (Skye, UK). The Letcombe nutrient solution (Drew & Saker 1984) was modified for wheat (5 mm KNO_3_, 2 mm NaNO_3_, 1.5 mm Ca(NO_3_)_2_, 1 mm MgSO_4_, 1 mm KH_2_PO_4_, 25 μm FeEDTA, 9.2 μm H_3_BO_3_, 5 μm KCl, 3.6 μm MnCl_2_, 770 nm ZnCl_2_, 160 nm CuCl_2_, 16 nm Na_2_MoO_4_, pH 5.8). The nutrient solution was exchanged three times per week. Pi starvation was initiated after 6 days of growth by replacing 1 mm KH_2_PO_4_ with 1 mm KCl. Roots and shoots were harvested (in triplicate) 0, 3, 6, 9 and 12 days after onset of the Pi depletion. The entire root of each plant was rinsed in deionised water, dried briefly on paper towels before freezing in liquid nitrogen.

Field‐grown *T. aestivum* cv. Hereward was harvested in 2011/2012 from sections 0 and 1 (representing continuous wheat plots, drilled in autumn 2010/2011) of the ‘Broadbalk’ field experiment (Rothamsted Research 2006; Watts *et al*. 2006), with low or high soil Pi availability (Table 2). The soil is a flinty, silty clay loam (Luvisol) with a clay content of 25–35% and with calcareous layers below 2‐m depth in the sampling area (Watts *et al*. 2006). Soil analyses and yield data were provided by the long‐term experiments National Capability (http://www.rothamsted.ac.uk/long-term-experiments-national-capability; eRA data 2010‐2013). Five replicates of root and shoot tissues were sampled at five growth stages (Zadoks *et al*. 1974): tillering (GS25), stem elongation (GS32), early and late booting (GS45/49), anthesis (GS65) and ripening (GS75; 20 days post‐anthesis) from plots receiving no Pi fertiliser (plot 20‐0/20‐1 ‘Pi‐starved’) and a control plot (35 kg Pi ha^−1^ applied as triple superphosphate), which was likely to yield Pi‐replete plants (plot 09‐0/09‐1 ‘Pi‐supplied’). Roots were excavated with a fork‐like spade, rinsed with deionised water, dried briefly on paper towel before freezing in liquid nitrogen and being stored at −80 °C. The shoot tissues were kept on ice and separated into either full shoot samples for chemical analysis or samples of different plant parts, which were frozen with liquid nitrogen.

All frozen tissue samples were ground with a mortar and pestle in liquid nitrogen (glumes and leaves) or with a SPEX SamplePrep 6870 Freezer/Mill (Metuchen, USA) (roots, ears, rachis and grain). Aliquots were stored at −80 °C for RNA extraction. The remaining shoot material was used for chemical analysis.

### Chemical Analysis

Oven‐dried shoot material (72 h/80 °C) was milled (ZM 200 Retsch mill, Haan, Germany) and stored in glass vials at room temperature in the dark. Mineral element concentrations were determined with Inductively Coupled Plasma‐Atomic Emission Spectrometry (Perkin Elmer LAS, Seer Green, UK) as described in Shinmachi *et al*. (2010). Aliquots of 250 mg were digested for 2 h at room temperature with 5 ml 70% nitric/70% perchloric acid mixture (15:85 v/v) in 25 ml glass vials. The vials were then placed into a temperature‐controlled carbolite heating block with a heating regime: 60 °C for 3 h, 100 °C for 1 h, 120 °C for 1 h, 175 °C for 2 h and cooled to room temperature. After adding 5 ml 25% HCl, the block was reheated to 80 °C for 1 h. Subsequently, 20 ml deionised H_2_O was added, heated for another 30 min at 80 °C and removed from the heating block to cool. Deionised H_2_O was added to give a final volume of 20 ml.

### Total plant RNA isolation, cDNA synthesis and sequence analysis

Total RNA was isolated from homogenised plant material using a modified protocol from Verwoerd *et al*. (1989) involving an additional phenol‐chloroform‐isoamyl alcohol extraction and DNase treatment (Promega, Madison, WI, USA). Integrity of the RNA and absence of genomic DNA was confirmed on a 1.5% (w/v) agarose gel. cDNA was synthesised in a total reaction volume of 20 μl using 2 μg total RNA, 1 μl 10 mm dT‐adapter primer and 1 h synthesis time according to the standard protocol for Superscript III Reverse transcriptase (Invitrogen, Carlsbad, CA, USA).

### Determination of transcript abundance by quantitative real‐time PCR

Real‐time quantitative PCR (q‐PCR) was performed in a 25 μl reaction volume for each sample containing 1 μl of cDNA, 12.5 μl SYBR^®^ Green JumpStart™ Taq ReadyMix™ (Sigma‐Aldrich, Gillingham, UK), 0.025 μl ROX reference dye, 250 mm, 150 mm or 100 mm of gene‐specific primer pairs (Table S1; Applied Biosystems 7500 Fast Real‐time PCR System, AB7500 Software 2.0.5). Wheat genes mostly exist as a trio of A, B and D homoeoloci in the hexaploid wheat genome, and each homoeolocus may contribute differentially to wheat phenotypes. The intention of the expression profile analysis presented was to identify the total expression profile of all three potential homoeoloci of TaPht1 transporters, based on the available sequence information. The gene specific *TaPht1* primer combinations cover all three genomes (ABD) for most expressed TaPht1 genes. Exceptions are TaPht1;6 (AB genomes) for which the alignment of D genome contigs was ambiguous, and TaPht1;7 (BD genomes) for which sequence information was only available from the D and B genome (Table S1). The amplification efficiency (E = 10(‐1/s) – 1) was determined for each primer pair (Table S1), using ten‐fold cDNA dilution series in triplicate (n = 3) for different tissues and treatments as well as the LinRegPCR package (Ramakers *et al*. 2003; Tuomi *et al*. 2010). Due to the similarity between TaPht1 gene sequences, locations for efficient primer combinations were limited, with a complete lack of primer combinations for efficient expression analysis in green leaf and shoot material. Primer concentrations were adjusted to improve the PCR efficiency and considered acceptable within a range of 85% to 115%. Efficiency testing included melting curve analysis, blank water control for primer dimer influence and cross‐amplification.

A 500 bp cDNA fragment covering the expression amplicon of each *TaPht1* gene analysed was amplified (Mastercycler^®^ gradient; Eppendorf Scientific, Stevenage, UK) using a gene specific primer pair (Table S5) and RedTAQ ready PCR‐mix (Sigma‐Aldrich, Gillingham, UK). These amplified DNA fragments were cloned into the pGEM‐Teasy vector system (Promega, Southampton, UK) and sequenced (MWG‐Eurofins, Ebersberg, Germany). Sequences were submitted to the EMBL database (Table S5). Due to the low level of transcripts, cDNA fragments for *TaPht1;9* and *TaPht1;14* were not cloned. For *TaPht1;3* and *TaPht1;4* no suitable real‐time q‐PCR primer combinations for expression analyses were found. The real‐time qPCR regime largely followed the SYBR Green JumpStart Taq ReadyMix (Sigma‐Aldrich, St Louis, MI, USA) standard protocol in a 25 μl reaction but used appropriate annealing temperatures for 20 s (Table S1) before the extension for 40 s at 60 °C. Blank water controls as well as melting curve analysis was performed with each real‐time PCR as a standard control. Furthermore, standard dilution series of each *TaPht1* plasmid‐PCR fragment in triplicate were included. The replicate CT‐values per time point/developmental stage were normalised using a reference gene (the heterogenous nuclear ribonucleoprotein Q (hnRNP Q), Ta.10105.1.S1; Long *et al*. 2010) expression. The heterogenous nuclear ribonucleoprotein exhibited the most stable constitutive expression under control as well as P starvation conditions among the reference genes tested (data not shown). The amount of each amplicon was quantified with respect to the standard curve of the individual TaPht1 standard as mRNA copy number per μl cDNA.

### Statistical analysis

Data were analysed using GenStat (16th edition, VSN International, Hemel Hempstead, UK). Expression data and P tissue concentrations in hydroponics experiments were analysed by two‐way anova with a factorial treatment structure (Fig. 3). Mineral concentration in wheat shoots from the field (Fig. S1, Table S2) were analysed by two‐way anova (*P *≤* *0.05). When residuals were not normally distributed, data were log_10_‐transformed (Table S3 for field expression analysis).

## Results

### The wheat phosphate transporter 1 family

**Figure 1:**
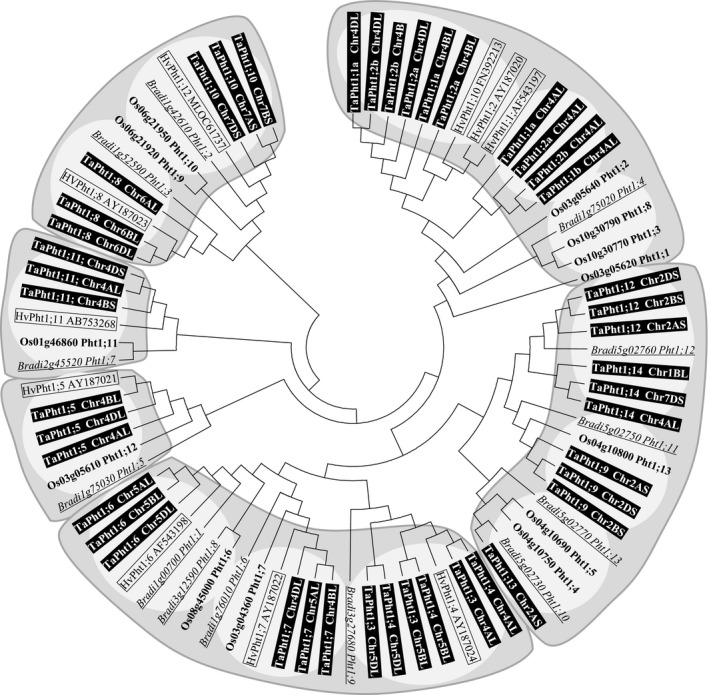
Phylogenetic relationship of coding nucleotide sequence of the phosphate transporter family 1 in cereals. A neighbour joining (Saitou & Nei 1987) unrooted tree was generated for *Brachypodium* (Bradi), rice (Os), barley (Hv) and wheat (Ta) *Pht1* transporters with MEGA6 (version 6.06) software; Tamura *et al*. 2013), from a multiple alignment (ClustalX version 2.0) using the coding nucleotide sequences. The bootstrap consensus tree inferred from 1000 replicates (Felsenstein 1985) is taken to represent the evolutionary history of the taxa analysed. The evolutionary distances were computed using the p‐distance method (Nei & Kumar 2000). The analysis involved 76 nucleotide sequences. All positions containing gaps and missing data were eliminated. Different protein clusters and subclusters are shaded. The different phylogenetic tree branch clusters and subclusters are shaded.

**Figure 2:**
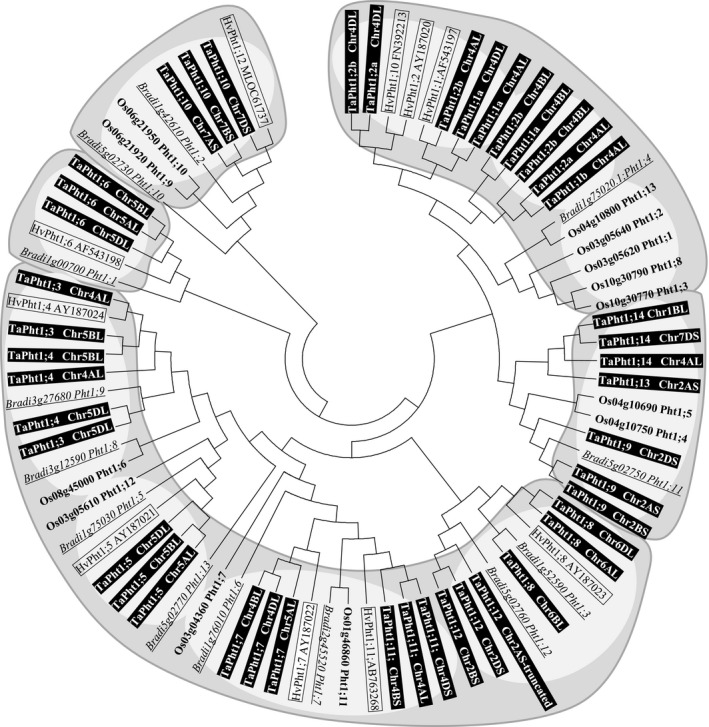
Phylogenetic relationship of protein sequence of phosphate transporter family 1 in cereals. A neighbour joining (Saitou & Nei 1987) unrooted tree was generated for *Brachypodium* (Bradi), rice (Os), barley (Hv) and wheat (Ta) Pht1 transporters with the MEGA6 (version 6.06) software (Tamura *et al*. 2013) from the multiple alignment (ClustalX version 2.0) using protein sequences. The bootstrap consensus tree inferred from 1000 replicates (Felsenstein 1985) is taken to represent the evolutionary history of the taxa analysed. The evolutionary distances were computed using the p‐distance method (Nei & Kumar 2000) The analysis involved 80 amino acid sequences. All positions containing gaps and missing data were eliminated. Different phylogenetic tree branch clusters and subclusters are shaded.

A maximum of 16 wheat A‐genome and 14 B‐ and D‐genome *TaPht1* genes were identified, representing 16 phylogenetically distinct TaPht1 transporters (Figs 1, 2, S1, Tables S1, S5). A trio of A, B and D homoeoloci were verified for 14 *TaPht1* genes (Table S5, Fig. S1, Data S1) on chromosomes 1, 2, 4, 5, 6 and 7. For *TaPht1;11*, homoeoloci were detected on the long arm of the A‐genome chromosome 4 and on the short arm of the B‐ and D‐genome chromosomes 4 (Table S5). The homoeoloci for *TaPht1;3, TaPht1;4* and *TaPht1;14* appeared to be on different chromosomes. *TaPht1;3* and *TaPht1;4* are neighbouring genes with one locus on the long arm of the B‐ and D‐genomes of chromosome 5, and one on the long arm of chromosome 4 AL. The *TaPht1;14* homeologous genes are located on the long arm of chromosomes 1BL and 4AL, as well as on the short arm of chromosome 7DS. Only one locus on the short arm of the A‐genome chromosome 2 was found for *TaPht1;13* and similarly on the long arm of chromosome 4A for *TaPht1;1b*. There are homologous sequences present in the incomplete draft genome sequence database of *Triticum uratu,* the hexaploid wheat A‐genome progenitor (TaPht1;13, PLANT_T.urartu_wgs||scaffold12793; TaPht1;1b, PLANT_T.urartu_wgs||scaffold1959), but none have been identified in the *Aegilops tauschii* genome (data not shown). Eleven of the wheat TaPht1s per genome are intronless in their coding DNA regions. Only a single intron is present in each of all homeoloci of *TaPht1;9*,* TaPht 1;10*,* TaPht 1;11*,* TaPht 1;12* and *TaPht 1;14* (Supplemental wheat TaPht1 genomic sequences).

Several of the 16 phylogenetically distinct TaPht1 transporters share a high degree of homology in their coding DNA as well as amino acid sequences. The sequence similarity data, presented in Fig. S1 and Table 1 for all A‐genome TaPht1s, except for the B‐genome TaPht1;12, are representative for all three genomes. The A‐genome *TaPht1;12* on chromosome 2AS is truncated by missing part of the 3′‐coding region, and has an insertion in the 5′‐region leading to a non‐functional gene. Furthermore, the genomic region of *TaPht1;14* on chromosome 1BL is not fully sequenced and only a partial coding sequence is available containing a deletion. TaPht1;1a, TaPht1;1b, TaPht1;2a and TaPht1;2b share a high degree of identity of >95% for DNA and >98% for amino acid sequences (Table 1), representing very close paralogous genes. These four transporters showed 65% to 73% sequence identity to TaPht1;3, TaPht1;4, TaPht1;5, TaPht1;6 and TaPht1;7, and a lower degree of homology with all other TaPht1 transporters, notably with TaPht1;10 (Table 1). The protein and coding DNA sequence of TaPht1;3 and TaPht1;4 from the A‐genome were both 99% identical. TaPht1;10 did not show a high sequence identity to any of the other TaPht1 transporters. TaPht1;9, TaPht1;12 and TaPht1;14 also exhibited low sequence identity to all other transporters, except to each other (~60%). TaPht1;3 and TaPht1;4 were most similar to TaPht1;5, TaPht1;6, TaPht1;7 and TaPht1;13 with 67–82% homology. TaPht1;12 and TaPht1;13 shared 50–60% homology with TaPht1;14 and TaPht1;9 (Table 1). Reverse Blast analysis of the low homology Pht1 genes, particularly of *TaPht1;9*,* TaPht1;10* and *Pht1;11*, to the NCBI protein database confirmed all identified genes to be members of the *Pht1* gene family.

Based on the phylogenetic analysis of the coding DNA and protein sequences, cereal *Pht1* genes can be subdivided into several clusters (Figs 1, 2). There are some discrepancies between DNA and protein analysis leading to some variation in homologous and/or orthologous relationships depending on which sequence information is utilised. The phylogeny confirms the close relationship of nearly all homoeologous wheat TaPht1 genes except *TaPht1;3, TaPht1;4, TaPht1;1a*,* TaPht1;1b*,* TaPht1;2a* and *TaPht1;2b*. An estimation of the homeologous relationship between the TaPht1;1a/b and Pht1;2a/b type genes could not be determined, even when including part of the 5′‐non‐coding/promoter and 3′‐non‐coding regions (Fig. S1). The only clear difference between the *TaPht1;1a* and *TaPht1;1b* type to the *TaPht1;2a* and *TaPht1;2b* type is a 12 base elongation of the 3′‐end leading to TaPht1;2a and TaPht1;2b proteins which were four amino acids longer. Based on the DNA coding sequences, the A‐genome TaPht1;1a/b and TaPht1;2a/b type genes were found in a separate sub‐cluster, distinct from the B‐ and D‐genome TaPht1;1a/b and TaPht1;2a/b genes, which also show a homologous relationship to the three barley Pht1 genes (Fig. 1). The high similarity in their coding regions, and partial similarity in the 5′‐/3‐’non‐coding regions, suggests two recent duplication events in the A‐, B‐ and D‐genome common ancestor leading to the paralogous *Pht1;1* and *Pht1;2* type genes, with further duplication to paralogous *Pht1;1a/b* and *Pht1;2a/b* type genes. *TaPht1;1b* is only present in the A‐genome chromosome 4 but not in the B and D genome. This A‐genome‐specific duplication, or B/D‐genome gene loss, is confirmed by the presence of *TaPht1;1a* in the genome of the A‐genome progenitor, *Triticum uratu,* but not in the D‐genome progenitor of *Aegilops tauschii*. Based on coding DNA sequence, *TaPht1;1a/b* and *TaPht1;2a/b* comprise a cluster with four phylogenetically closely related rice genes, three barley *Pht1* genes and one *Brachypodium Pht1* gene (Fig. 1). The separate sub‐cluster of the *Brachypodium Pht,* and the four rice *Phts* does not suggest any clear homologous or orthologous relationships to the *TaPht1;1a/b* and *TaPht1;2a/b* wheat genes (Fig. 1). This is also seen for the protein sequence‐based phylogeny. The two barley proteins, HvPht1;2 and HvPht1;10, seem to be orthologous to the D‐genome TaPht1.2a and TaPht1;2b, whilst HvPht1;1 is orthologous to the A‐genome TaPht1;2b.

A second DNA‐based cluster is composed of the four wheat TaPht1s, *TaPht1;9*,* TaPht1;12*,* TaPht1;13* and *TaPht1;14*. No barley gene have been identified homologous to those wheat genes and there is no rice gene homologous to *TaPht1;12* and *TaPht1;14,* with one *Brachypodium* gene homologous only to *TaPht1;12*. One rice and one *Brachypodium* gene are homologous to *TaPht1;9*. Two rice and *Brachypodium* genes are homologous to *TaPht1;13* (Fig. 1). The A‐genome TaPht1;13 protein sequence is much more closely related to the homoeologous TaPht1;14 proteins, building up a common cluster together with the TaPht1;9 homoeologous proteins (Fig. 2). The *Brachypodium* Pht1;11 can be confirmed to be orthologous to TaPht1;9. The rice Pht1;4 and Pht1;5 protein sequences are more closely related to the Pht1;9 proteins, and the rice Pht1;13 protein has a close relationship to the TaPht1;1 and TaPht1;2 cluster, with an orthologous relationship to the *Brachypodium* Pht1;4 protein (Fig. 2).

A third cluster, comprising the coding DNAs of *TaPht1;3*,* TaPht1;4*,* TaPht1;6* and *TaPht1;7,* may be further subdivided into three sub‐clusters. Two *Brachypodium* genes and one barley and rice gene are homologous to *TaPht1;6*; another *Brachypodium*, a barley and a rice gene are more homologous to *TaPht1;7*. There is only one *Brachypodium* and no rice genes which are homologous to *TaPht1;3* and *TaPht1;4* (Fig. 1). The proteins of TaPht1;3, TaPht1;4 and TaPht1;7 are found in one large branch cluster, including TaPht1;5, TaPht1;8 and TaPht1;11 and TaPht1;12, but the latter are found in different clusters based on the phylogeny of the coding DNA.

With some exceptions, the closely related orthologous barley, rice and *Brachypodium Pht1* genes could be verified by common DNA and protein relationships. For *Brachypodium* Pht1;8 and Pht1;13, as well as for the rice Pht1;6, there were no closely related wheat homologues.

The very high sequence similarity between *TaPht1;3* and *TaPht1;4,* in their coding, as well as partially in their non‐coding and promoter regions, as also seen for TaPht1;1a/b and TaPht1;2a/b type genes, is suggestive of gene duplication. Interestingly the paralogous genes are phylogenetic more closely related than the homeologous genes (Figs 1, 2). The coding DNA sequences of *TaPht1;8* and *TaPht1;10* are closely related in another cluster. Both *Pht1s* have orthologous genes in barley and *Brachypodium*, but the two rice genes, *OsPht1;9* and *OsPht1;10* seem to be more closely related to *TaPht1;10*. This is confirmed by the protein phylogeny in which TaPht1;10 is in a separate cluster together with the *Brachypodium* and rice transporters, including *Brachypodium* Pht1;10, which is shifted from the coding DNA similarity to TaPht1;13, to a closer protein relationship to TaPht1;10.

### The TaPht1 gene expression in roots of hydroponically grown wheat seedlings

The real‐time PCR gene expression analysis is the sum of all three homeoloci of each TaPht1 homeologous subfamily, and therefore provides information on the overall expression pattern. The high sequence similarities and/or sequence limitations restricted the generation of oligonucleotide primers able to differentiate between the homeoloci. Due to the very high sequence similarity, including in the non‐coding regions, between both TaPht1;1a and TaPht1;1b and also TaPht1;2a and TaPht1;2b, no differentiation of gene expression of the A and B genome types was possible; this prevented identification of the most strongly regulated genes in the homoeologous sub‐family by PCR. Transcripts of most wheat homeoloci were identified, however, by different RNA‐Seq experiments (Table S1).

**Figure 3:**
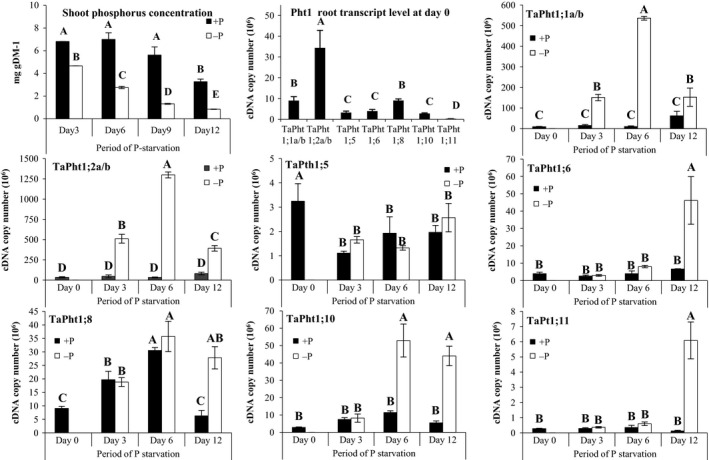
Phosphorus concentrations in wheat seedlings and *TaPht1* transporter gene expression in hydroponically grown wheat roots exposed to Pi starvation. Differential *TaPht1* expression in roots of wheat (cv. Hereward) seedlings grown in hydroponic culture in a controlled environment for 6 days and exposed to 12 days of Pi starvation. Means and SE are presented as cDNA copy number (× 10^6^) in 1 μl corresponding to 0.1 μg total RNA from three biological replicates (*n *= 3). Bars sharing the same letter are not statistically different between Pi availability at different time points during Pi starvation (*P *> 0.05).

Plants grown under Pi starvation in hydroponics had significantly lower total shoot P concentrations than plants grown in the presence of Pi (Fig. 3). No root expression of *TaPht1;3*,* TaPht1;4*,* Pht1;7*;* Pht1;9*,* Pht1;13* and *Pht1;14* was detected in hydroponically grown plants. At day 0 under Pi supply, the strongest expression in the root was seen for *TaPht1;2a/b,* with a three‐ to eight‐fold higher transcript abundance compared to most of the other expressed *TaPht1* genes. The transcript abundance of *TaPht1;11*, with just 0.28 cDNA copies per 0.1 μg total RNA, was particularly low. Pi deprivation led to three expression patterns. A quick response with up‐regulated transcript levels after 3 days of Pi starvation for *TaPht1;1a/b* and *TaPht1;2a/b*, which further increased at day 6 to a maximum level 27‐ to 50‐fold higher compared to day 0, and then declined again by day 12 (Fig. 3). A late Pi starvation response was seen for *TaPht1;6*,* TaPht1;10* and *Pht1;11*. A Pi starvation‐related increase of the transcript level of *TaPht1;10* was not seen before day 6 and for *TaPht1;6* and *TaPht1;10*, not before day 12, with an eight‐ to 15‐fold higher expression compared to day 0 (Fig. 3). Finally, *TaPht1;5* and *TaPht1;8* transcript abundances were influenced more by age than Pi starvation, although a higher transcript level of *TaPht1;8* was observed in Pi‐starved roots at day 12, along with *TaPht1;6* and *TaPht1;11* (Fig. 3).

### The TaPht1 transcript abundance in Pi‐starved field‐grown wheat at different developmental stages

Wheat plants grown on P‐depleted plots with a substantially reduced soil Olsen P content lead to drastically reduced straw and grain yields in comparison to P‐fertilised control plots (Table 2). From tillering until maturity, Pi concentrations in shoot and ear tissues of wheat plants grown on non‐Pi fertilised plots were significantly lower than in plants grown on the Pi fertilised control plots (Fig. S2). Shoot P concentrations decreased with plant development, but increased in ears. Other elements such as shoot Mo and Zn concentrations exhibited the same patterns as shoot P concentrations, whereas shoot K concentrations increased in Pi‐starved plants until booting stage (Fig. S2).

**Table 2:** Soil and yield data from plots at the Broadbalk field site (eRA data). Olsen P, K, Mg, pH and % organic matter determined for air‐dried soil < 2 mm top soil (0–23 cm) sampled in autumn 2010 from compacted stubble from Section 0 (straw incorporated) and Section 1 at Broadbalk. Yields (grain + straw with 85% DM) are from year 2011 and 2012 (eRA data 2010‐2013)*. Plot numbering refers to the data reference: Rothamsted Research (2006)

Treatment Plot (no – Sec)	Olsen P mg kg^−1^	Exch K mg kg^−1^	Exch Mg mg kg^−1^	pH	% Org C	2011 Grain t ha^−1^	2011 Straw t ha^−1^	2012 Grain t ha^−1^	2012 Straw t ha^−1^
Control (09–S0)	72	358	82	8.0	1.19	5.42		6.10	
Control (09–S1)	55	308	76	8.0	1.10	4.83	1.74	6.36	3.52
P deplet (20–S0)	4	386	83	8.2	1.09	1.56		1.12	
P deplete (20–S1)	3	382	78	8.2	1.09	0.78	0.25	0.14	no straw

*Provided from the long‐term experiments National Capability at Rothamsted (Andy Macdonald, Margaret Glendining).

**Figure 4:**
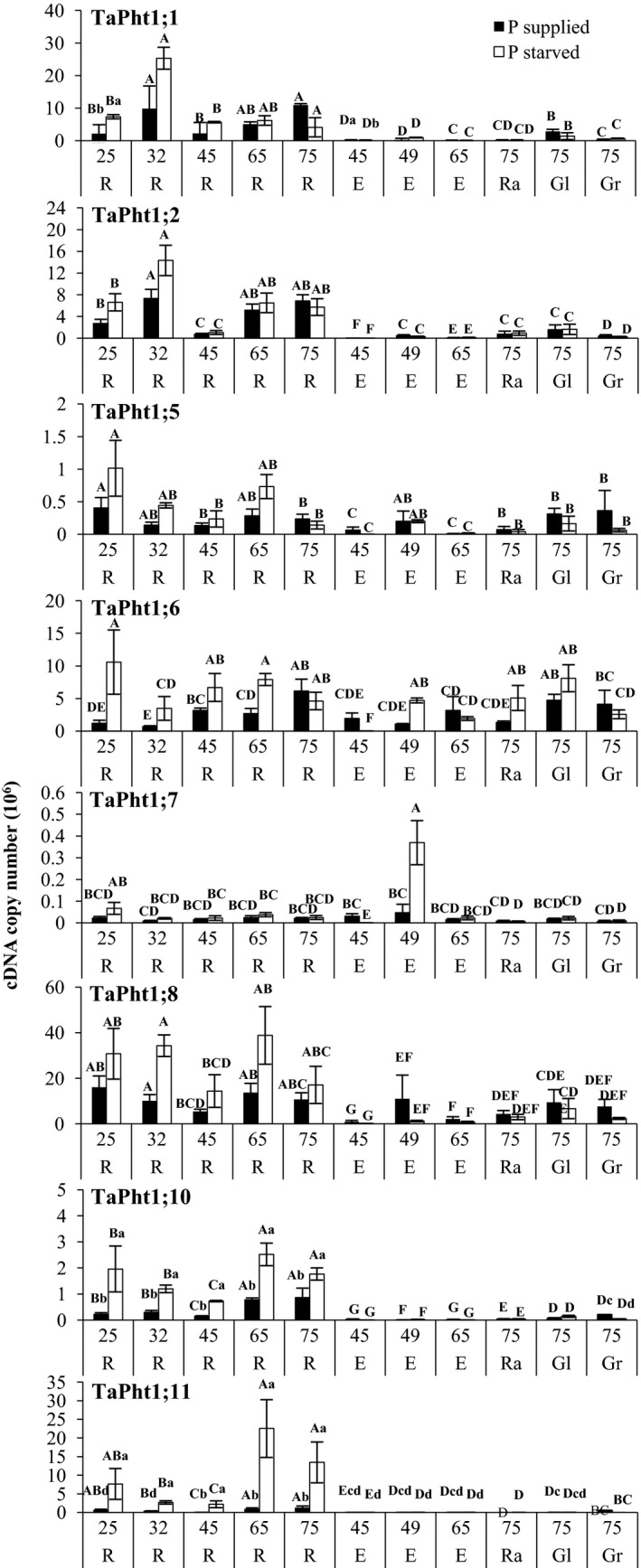
Transcript abundances of *TaPht1;1*,* TaPht1;2*,* TaPht1;5*,* TaPht1;6, TaPht1;7*,* TaPht1;8*,* TaPht1;10 and TaPht1;11* in root and ear tissues of field‐grown wheat in 2012. Gene expression is shown as RNA copy number (× 10^6^) in 1 μl cDNA corresponding to 0.1 μg total RNA in root (R) and ear tissues (E = entire ear, Ra = rachis, Gl = glume, GR = grain), GS = growth stage) at tillering (GS25), stem elongation (GS32), early booting (GS41), full booting (GS45), late booting (GS48), anthesis (GS65) and milk ripening (GS75). For statistical analysis, the data were log_10_‐transformed and predicted means (log_10_‐scale) were compared using the SE of difference (on the relevant degrees of freedom) and least significant difference values at the 5% level of significance. Statistical properties are presented in S4. Bars sharing the same letter are not statistically different between tissues at particular growth stages (upper) and between different soil Pi availability levels (*P *> 0.05; lower).

The transcript abundances of the *TaPht* genes in roots of wheat plants grown in Pi‐fertilised field plots were similar to those grown in hydroponic culture. Transcript abundance in field‐derived plant material was verified for *TaPht1;1a/b*,* TaPht1;2a/b*,* TaPht1;5, TaPht1;6, TaPht1;7*,* TaPht1;8, TaPht1;10* and *TaPht1;11*. A weak trend was observed in which *TaPht1* expression was highest at early growth stages and at maturity, and least during booting and anthesis (Fig. 4). For example, *TaPht1;1a/b* transcript abundance increased in roots at the beginning of stem elongation, decreased at booting and anthesis, and increased again during ripening (Fig. 4). *TaPht1;1a/b* was also expressed in ears, glumes, grains and in the rachis (Fig. 4). Root *TaPht1;6* transcript abundance also increased under Pi starvation at tillering, stem elongation and anthesis (Fig. 4). *TaPht1;6* was the only *TaPht1* transporter with high transcript abundance during Pi starvation in the ear and particularly in the rachis (Fig. 4). *TaPht1;7* transcript abundance was very low in all tissues; an influence of Pi starvation was only seen with increased transcript abundance in ear tissues at late booting stage (Fig. 4).

**Figure 5:**
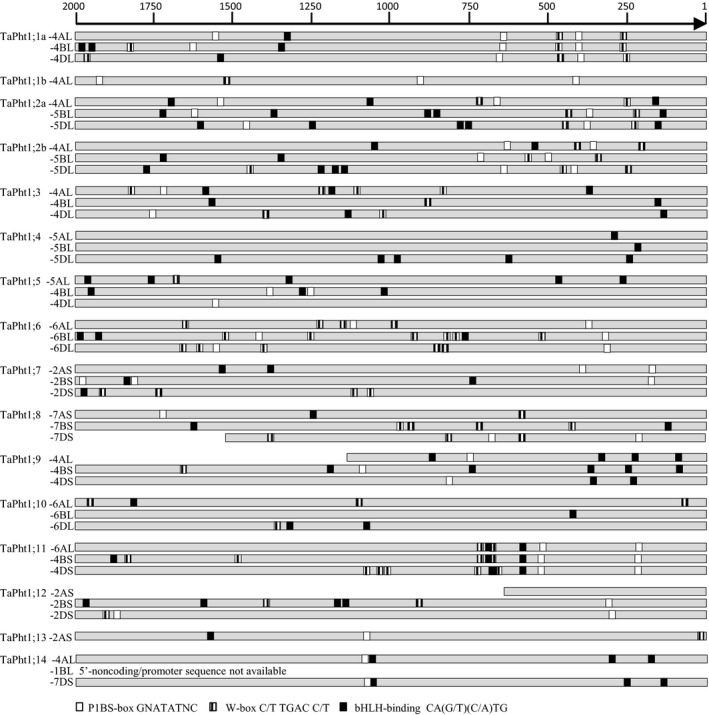
Location of putative MYB transcription factor binding elements P1BS (GNATATNC), WRKY and bHLH transcription factor W‐box (C/T TGAC C/T) and helix‐loop‐helix (CA[G/T][C/A]TG) element in the promoter/5′‐non‐coding region 2000 to 1 bp upstream of the ATG start codon of homoeologous wheat *Pht1* genes.

In contrast to the hydroponic study, *TaPht1;2a/b*,* TaPht1;5* and *TaPht1;8* transcript abundances in roots showed no response to Pi availability (Fig. 5). *TaPht1;2a/b* transcript abundance in the root was highest at tillering and elongation, decreased at booting and increased again at maturity (Fig. 5). Root *TaPht1;5* gene expression was highest at tillering and milk ripening, lowest at elongation and at an intermediate level in ear tissues (Fig. 5). *TaPht1;*8 transcript abundance followed the same pattern as *TaPht1;2a/b* transcript levels but was more abundant in the grain at ripening (Fig. 4).

Both *TaPht1;10* and *TaPht1;11* transcript abundances were much lower in shoot tissues compared to the roots (Fig. 4). In contrast to *TaPht1;1* and *TaPht1;6*, Pi starvation increased transcript abundances of *TaPht1;10* and *TaPht1;11* in the root throughout development (Fig. 4). *TaPht1;10* and *TaPht1;11* transcript abundances followed a similar pattern to other root‐expressed *TaPht1* transporters, being highest at tillering, decreasing after elongation and increasing again at milk and grain ripening stages (Fig. 4). However, *TaPht1;10* and *TaPht1;11* transcript abundances were highest at maturity but decreased in the grain during Pi starvation, similarly to *TaPht1;6* (Fig. 4).

### Putative transcription factor binding sites in the promoter region of selected wheat *Pht1* genes

The putative MYB transcription factor PHR1 promoter element binding site, P1BS‐Box (GNATATNC), the WRKY and bHLH transcription factor W‐box (C/T TGAC C/T) and the helix‐loop‐helix (CA[G/T][C/A]TG) factor have been reported to be involved in the regulation of the Pi starvation response (Rubio *et al*. 2001; Zhou *et al*. 2008; Lin *et al*. 2009; Wang *et al*. 2014). A similar location for putative transcription factor binding sites in the promoter regions of all three homoeologous genes may be an indication of the importance of these *cis*‐elements for the regulation of gene expression. A rapid early or a late response of the root gene expression of the TaPht1 genes implicates differences in their regulation, which may relate with the presence or absence as well as the position of those regulatory promoter elements. The locations of those elements in all available promoter/5′‐non‐coding regions of the wheat Pht1 genes were analysed and are indicated as upstream of the ATG start codon.

In the promoter/5′‐non‐coding regions of *TaPht1;1a,* the locations of two P1BS boxes and two W‐boxes are conserved in the 750 to 1 upstream region for all three homoeologous genes (Fig. 5). Although there is 100% protein and 98% coding DNA identity between the A‐genome *TaPht1;1a* and *TaPht1;1b,* the promoter/5′‐non‐coding regions share only 44% identity. One P1BS box is present at position 437 as in TaPht1;1a, but two additional P1BS boxes are located more 5′‐distally and there is no proximity to a W‐box, as found for TaPht1;1a. No bHLH‐binding element is present in the TaPht1;1b promoter region.

The promoter/5′‐non‐coding region of *TaPht1;2a* contains W‐boxes in similar positions to *TaPht1;1a*, apart from the A‐genome gene promoter, in which the second W‐box is located further 5′‐upstream. The *TaPht1;2a* promoter/5′‐no‐ncoding region contains only one P1BS box in a similar location to that found in *TaPht1;1a*, which in the A‐genome, is also shifted as with the W‐box (Fig. 5). Interestingly, the distance between the first P1BS box to the second W‐box in the *TaPht1;1*a promoter/5′‐non‐coding region is similar to the *TaPht1;2a* promoter P1BS‐box distance to the W‐box, for all three homeologues. Additionally, the *TaPht1;2a* promoter/5′‐non‐coding region contains a bHLH‐binding site at the 200 bp upstream region in all homoeologous promoter/5′‐non‐coding regions, with a similar distance to the first W‐box, which is absent in the *TaPht1;1a* promoter. Further P1BS‐boxes, W‐boxes and bHLH‐binding sites are present upstream for all three *TaPht1;1a* and *TaPht1;2a* genes but with a random and non‐specific distribution in the homoeologous promoter/5′‐non‐coding regions (Fig. 5). Interestingly the promoter/5′‐non‐coding region of the homeologous TaPht1;2b has a similar pattern of P1BS‐boxes and W‐boxes as found for TaPht1;1a, with a similar distance between the boxes and to each other. In the B‐genome promoter, this combination is shifted slightly to 5′. Several bHLH elements with random non‐specific distribution are present in the 1000–2000 upstream region in all three promoters. There are no common bHLH‐binding sites in the 200 bp upstream region as found in theTaPht1;2a promoter. The common region 200 bHLH‐binding site in the TaPht1;2a promoter may allow additional regulation by an pHLH transcription factor.

A specific pattern of the three *cis*‐elements was found in the promoter/5′‐non‐coding region of the homoeologous genes of *TaPht1;11*. In the 750 to 1 region two P1BS‐boxes, as well as two W‐boxes and bHLH‐binding sites, are in nearly identical positions (Fig. 5). The second upstream bHLH‐binding site is partially overlapping with the first W‐box. A P1BS‐box at a similar position was also found in the 350 to 300 promoter/5′‐non‐coding region of the homeologues of *TaPht1;6*. Four or five W‐boxes are present further downstream in all homoelogous promoters/5′‐non‐coding regions, but in more distinct locations without any specific pattern (Fig. 5). Although *TaPht1;10* also responded to P starvation in a delayed manner, no uniform distribution of the three transcription factor binding elements was present (Fig. 5). The promoter/5′‐non‐coding regions of the homoeologous *TaPht1;5* and *TaPht1;8* genes contain only randomly distributed *cis*‐elements with no specific patterns, with exception of a similar position (‐650 bp) of a W‐box in the A‐ and D‐genome promoters of *TaPht1;8*. The B‐genome promoter of *TaPht1;8* contains two W‐boxes, each approximately 150 nucleotides upstream and downstream relative to the single W‐box found in the A‐ and D‐genome promoter (Fig. 5). For those genes, no root transcript could be verified. Only *TaPht1;3*,* TaPht1;4*,* TaPht1;7; TaPht1;9* and TaPht1;14 had similar positions for at least one of the binding sites in the homoeologous promoter/5′‐non‐coding regions. A bHLH‐binding site was present in the 230 to 302 bp region but no W‐boxes or P1BS‐boxes were found in any of the three homeologous *TaPht1;4* promoter/5′‐non‐coding regions. The *TaPht1;7* homeoloci promoter/non‐coding regions had a nearly identical position of the P1BS‐box at position 164 to 172, and no or randomly distribution of the W‐box and bHLH‐binding sites. Two conserved bHLH‐binding sites were present in the 211–363 regions of all homeologous TaPht1;9 promoter/5′‐non‐coding regions. Two conserved bHLH‐binding sites were also present in similar positions in the A‐ and D‐genome promoter/5′‐non‐coding region of TaPht1;14. The corresponding sequence region of the B‐genome TaPht1;14 was not available in the genome databases.

## Discussion

### A complex phylogeny of the wheat *Pht1* gene family

In dicotyledonous genomes, the size of the *Pht1* gene family seems to be highly variable with just five *Pht1* genes in *Vitis vinifera*, and up to 28 *Pht1* genes in *Brassica rapa* (Proost *et al*. 2015). In the cereal genomes analysed, the *Pht1* gene family does not show high variation in the number of Pht1 genes. In contrast to rice, maize (not shown) and *Brachypodium* genomes, which contain all 13 *Pht1* genes, 16 Pht1 genes were identified in the wheat A‐genome and 14 Pht1 genes in the B‐ and D‐genomes. The comparison of the cereal *Pht1* gene sequences allowed a phylogenetic separation into different phylogenetic distinct gene clusters. The differences found in wheat compared to rice, barley and *Brachypodium*, indicate a complexity of the cereal *Pht1* gene family which may be partly explained by additional gene duplication and/or loss of genes during evolution, as seen for the rice or barley or wheat *Pht1* in different clusters. The different gene compositions for the *Pht1* gene families in the different grass species may be explained by different evolutionary development influencing the genomes of the different grass species. The grass genomes differ in size, ploidy level and chromosome number. Two polyploidisation events (Feldman *et al*. 1995) led to the hexaploid wheat genome (AABBDD; 2n = 42) of ~16000 Mbp size. The diploid rice (2n = 24) and *Brachypodium distachyon* (2n = 10) genomes (~430 Mbp and ~272 Mbp) are much smaller than the wheat genome. Although in general the gene order in the nuclear genomes of all grasses has been preserved, genomic rearrangements, duplication and polyploidisation events during the evolution of the different grass species has led to differences in gene copy number. The occurrence of homoeologous *Pht1* genes on different chromosomes or different chromosome arms, as found for *TaPht1;3* and *Pht1;11*, suggest rearrangements between chromosome 4 and 5, and in the A‐genome between the short and long arm of chromosome 4. In comparison to its progenitors, many low copy DNA sequences seem to have been deleted in hexaploid wheat since the polyploidisation event (Feldman *et al*. 1997). Recent complete genome data on wheat chromosome 3B indicated that gene duplication played a major role in the recent evolution of wheat chromosome 3B (Glover *et al*. 2015). On chromosomes 11 and 12 of rice, recent segmental duplications, including large ongoing individual gene duplications, have been identified (Jiang *et al*. 2007). Furthermore at least ten duplicated regions, which represent 67.5% of the genome, were identified in wheat. Salse *et al*. (2008) suggested an ancient duplication of the diploid wheat genomes before their hybridisation into polyploidy wheat. Those differences can be seen in the phylogenetic relationship between wheat, barley, rice and *Brachypodium* in relation to DNA and amino acid sequences. A homologous gene (or homologue) is a gene inherited in two species by a common ancestor. While homologous genes can be similar in sequence, similar sequences are not necessarily homologous. Orthologous are homologous genes where a gene diverges after a speciation event, but the gene and its main function are conserved. Orthologous genes are generally assumed to retain equivalent functions in different organisms and to share other key properties (Gabaldón & Koonin 2013). The homologous phylogenetic closely related DNA sequence similarities found does not always explain the orthologous relationship in relation to the function in the individual cereal species. The parallel analysis of the protein and DNA sequences provided information about the orthologous relationships: the differences of the clustering for the wheat TaPht1 homeologous subfamilies may be explained by differences in the paralogous relationships, which are seen more clearly using the DNA sequences, rather than on homologous similarity based on amino acid sequences.

### Regulation of *TaPht* transcript abundances by mineral nutrition

Phosphate availability regulates root system architecture as well as many transcriptional, biochemical and physiological processes. One response to Pi deficiency is the increase of Pi‐uptake capacity, as shown by Ullrich‐Eberius *et al*. 1981; facilitated by the reported regulation of the *Pht1* genes at the transcriptional as well as the post‐transcriptional levels. This study compared two contrasting experimental systems to further understand the influence of Pi deficiency on wheat *Pht1* gene expression. In many studies, Pi deficiency often leads to rapid increased steady‐state expression of multiple *Pht1* genes. For example, *Pht1* transporter expression in tomato increased within 24 h of Pi starvation and decreased again after 24 h of Pi resupply (Liu *et al*. 1998). A more rapid response of *Pht1* gene expression to Pi deprivation was found in *Arabidopsis* (Misson *et al*. 2005), suggesting a finely coordinated response to Pi availability. In the artificial hydroponic growth system used here, there was a rapid induction of some wheat *Pht1* transporters, whereas others exhibit a delayed but equally strong induction to Pi starvation or no response at all. Similar observations were made for *Pht1* transporters in maize and other plant species (Nagy *et al*. 2006; Morcuende *et al*. 2007; Lapis‐Gaza *et al*. 2014). The pre‐culture of the young wheat seedlings in the high Pi hydroponic experimental setup may have allowed the plants to store P for remobilisation. In contrast the quick reduction of shoot P concentrations resulted in a rapid up‐regulation of Pi transport to attempt to maintain the Pi content in the plant. A rapid up‐regulation of Pi transport suggests the importance of the transporters in initial Pi root uptake to enable a maximum Pi uptake under limited conditions or for increased Pi translocation to support upper plant parts. There are rice *Pht1* genes in the same cluster of the *Pht1* gene family which are putative orthologous to the rapidly and strongly Pi starvation up‐regulated wheat *TaPht1;1* and *TaPht1;2* genes. The phylogenetically closest, *OsPht1;2*, has been characterised as a low affinity Pi transporter with expression up‐regulated by Pi starvation, and localised throughout the stele in primary roots and lateral roots, but not in epidermal and cortical cells of Pi‐deprived roots (Ai *et al*. 2009). In contrast, rice *OsPht1;1* and *OsPht1;8* are constitutively expressed and not influenced by Pi starvation. RNAi interference experiments have revealed the importance of both transporters for Pi uptake, and OsPht1;8 has been characterised as high‐affinity Pi transporter. The characteristics of all three rice genes suggest an involvement in Pi uptake as well as in translocation (Jia *et al*. 2011; Sun *et al*. 2012). The expression patterns of *TaPht1;1* and *TaPht1;2* indicate similar functions in wheat.

The rice genes *OsPht1;9* and *OsPht1.10,* orthologous to wheat *TaPht1;10,* and *OsPht1;6*, orthologous to *TaPht1;6*, have been shown to be important for Pi uptake as well as translocation, and are all up‐regulated by Pi starvation, shown under long‐term Pi starvation. Similarly, to TaPht1;6, the rice OsPht1;6 showed a late up‐regulation at 14 days of Pi starvation (Ai *et al*. 2009; Wang *et al*. 2014). Differential *Pht1* regulation in the Pi starvation response is an important consideration for determining which *Pht1* transporters may be potential targets for Pi efficiency crop improvement.

Several regulators of Pi homeostasis in plants have been identified (Lin *et al*. 2009). The MYB transcription factor PHR1 activates several Pi starvation‐induced genes by binding to the P1BS promoter element (Rubio *et al*. 2001; Zhou *et al*. 2008). Furthermore, WRKY, bHLH as well as the C2H2 zinc finger transcription factor have been reported to be involved in the regulation of Pi starvation response (Lin *et al*. 2009; Wang *et al*. 2014). Miao *et al*. (2009) identified P1BS elements (GNATATNC), WRKY and bHLH transcription factor W‐box (C/T TGAC C/T) and helix‐loop‐helix (CA[G/T][C/A]TG) binding elements in the *TaPht1;2a* promoter. The expression patterns of *TaPht1;1a*/*b* and *TaPht1;2a/b* were identical in relation to Pi starvation in hydroponic cultures. Promoter analysis of *TaPht1;1a* revealed P1BS and W‐box binding elements in similar regions as found in the *TaPht1.2a* and *TaPht1;2b* promoter (Fig. 5, Table S4). Overexpression of the wheat PHR‐MYB transcription factor in transgenic wheat increased expression of *TaPht1;2a/b* (Wang *et al*. 2013; the primer used did not distinguish between the homeologues), suggesting regulation of *TaPht1;2a/b* as well as *TaPht1;1a* by the same molecular regulators. The rice *OsPht1;1* promoter is lacking the P1BS element, and Sun *et al*. (2012) suggested that *OsPht1;1* may not be regulated by PHR. In contrast, the promoter region of the phylogenetic closely related *OsPht1*;*2*, which is also up‐regulated by P starvation, has two W‐boxes as well as two bHLH‐binding sites (data not shown) like TaPht1;2, implying a similar regulation. Wang *et al*. (2014) identified a WRKY45 transcription factor able to activate *Arabidopsis Pht1;1* in response to Pi starvation. In the promoter region of *TaPht1;6* and *TaPht1;11*, which showed a delayed Pi starvation response, there is at least one P1BS element in a similar position as compared to *TaPht1;1a* and *TaPht1;2a/b*, but the positions of the W‐box elements are not in the same proximity to the W‐box as found for *TaPht1;1a* and *TaPht1;2a/b* (Fig. 5, Table S4). The overexpression of TaPHR in wheat increased the expression of *TaPht1;6* (Wang *et al*. 2013), revealing the importance of the MYB transcription factor for the regulation of *TaPht1;6*. The rice OsPht1;6 promoter contains also two W‐boxes very close to each other (20 nucleotides, data not shown) which are also present in comparable location in the promoter of the B‐ and D‐genome *TaPht1;6* genes just three nucleotides from each other explain similar late Pi starvation response. In addition, the delayed Pi starvation response found in the hydroponic starvation experiment indicated involvement of other or additional regulators, in contrast to *TaPht1;1a* and *TaPht1;2a/b*. All *Pht1;11* promoter regions contain two helix‐loop‐helix binding sites in nearly identical positions and partly overlapping the W‐box binding site. This would enable regulation by bHLH transcription factors, in contrast to *TaPht1;6*, with either no or completely different locations in their respective promoter regions (Fig. 5, Table S4). A P1BS‐box is present in the promoter region of the orthologous rice *OsPht1;9* but not of *OsPht1;10*, although both genes are strongly induced by Pi starvation. *TaPht1;10* gene expression was also strongly up‐regulated by Pi starvation from day 6 onwards, and there is no common pattern of W‐box and bHLH binding sites and a complete absence of a P1BS‐box in all three homoeologous promoter regions of *TaPht1;10* (Fig. 5, Table S4), suggesting different regulation. Transcriptome analysis in *Arabidopsis* identified more than 40 Pi‐responsive transcription factor genes (Misson *et al*. 2005; Morcuende *et al*. 2007). Some transcription factors were responsive in the short term and others during medium term or late Pi deficiency, suggesting specific sets are involved in regulating early and late responses of plants to Pi deficiency (Misson *et al*. 2005), and explaining the need for different sets of regulatory binding domains in the promoter region of *Pht1* transporter genes. Root gene expression could not be verified for all wheat *TaPht1* genes under normal and Pi starvation conditions. For some homeologous gene subfamilies, at least one of the described transcription factor binding sites is present in a similar promoter location. In addition to the regulation by P availability, tissue and cellular specific regulation must be considered responsible for the complex gene regulation.

There is a lack of studies investigating gene expression in agronomic systems in relation to nutrient use. Shinmachi *et al*. (2010) demonstrated in some tissues, including roots, of field‐grown wheat that the expression of some sulphate transporter genes was up‐regulated in sulphur‐deficient plots, as also described for hydroponically cultured plants (Buchner *et al*. 2010). Teng *et al*. (2013) described high up‐regulation of *TaPht1;1*, and *TaPht1;2* under high Pi fertilisation, whereas *TaPht1;8* expression decreased with increasing Pi supply, probably due to reduced mycorrhiza infection under such conditions. No information (accession numbers, protein or nucleotide sequences), except for primer sequences, were provided. Alignment of the primer squences verified the specificity for the *TaPht1;1*,* TaPht1;2* and *TaPht1;8* genes. In contrast to the artificial hydroponics the soil P availability is much more complex for a growing plant. The efficiency of P use by plants from soil and fertiliser sources is often poor despite many soils containing a relatively large amount of total P that is not all available to plants. The low P soil mobility and Pi availability to the plants demands different requirements for regulation of the Pi uptake system. Additionally, the root structure of soil‐grown and hydroponically grown plants differs substantially, which will affect the control of any nutrient uptake.

The transcript pattern of the wheat *Pht1* genes exhibits a more complex regulation in field‐derived wheat plant material compared to hydroponic culture systems. *TaPht1* transcript patterns in field‐grown roots exhibited high expression levels at early vegetative growth stages. These high transcript levels coincided with a growth stage when Pi requirement was high (Römer & Schilling 1986). Expression and possibly therefore requirements decreased during the booting stage (Fig. 4). However higher Pi fertilisation during later gowth stages increased thousand‐grain weight in wheat (Römer & Schilling 1986). In addition, Sun *et al*. (2012) observed a considerable increase in Pi transport into the shoot during grain filling by *OsPht1;1* overexpression in rice. The same increase was also seen for the root expression of several *TaPht1* transporter genes at anthesis and post‐anthesis, including substantial post‐anthesis expression in glumes and grains, indicating continued Pi uptake from the soil solution and delivery to the grain during grain development and ripening (Fig. 4).

Transcripts of root‐expressed *TaPht1* transporters were also found in wheat ear (spike) tissues. In contrast to *TaPht1;5* homologues such as *HvPht1;5* (Rae *et al*. 2003; Huang *et al*. 2011) or *OsPht1;12* (Paszkowski *et al*. 2002; Fig. 2), *TaPht1;5* was weakly expressed in root and ear tissues (Fig. 4). Specific *Pht1* transporters are preferentially expressed in green and mature anthers, for instance in *Arabidopsis* (Mudge *et al*. 2002), maize (Nagy *et al*. 2006) and barley (Druka *et al*. 2006). Therefore, it is likely that high transcript abundance of *TaPht1;7* at growth stage 48 in ear tissues (Fig. 4) was coincident with anther or pollen development at booting, with very weak expression in all tissues at other developmental stages, as also found for the rice homologue *OsPht1;7* (Paszkowski *et al*. 2002; Fig. 2). *TaPht1;6* transcripts were present in all tissues analysed. This agrees with the expression pattern described for the rice homologous *OsPht1;6* (Ai *et al*. 2009). RNAi knockout of *OsPht1;6* decreased P uptake as well as Pi transport within the plant. Promoter‐GUS expression was seen in almost all tissues and cell types, except for the epidermis of younger primary roots (Ai *et al*. 2009). The general expression patterns of *TaPht1;6* and *TaPh1;8*, with expression in all tissues, including developing wheat grains, suggests a similar function in wheat, playing a broad role in Pi uptake, translocation and internal transport throughout the plant.

A significant influence of Pi fertilisation on *Pht1* transcript abundance in field‐grown wheat was only found for two *Pht1* genes and restricted mainly to root tissues. The gene expression of *TaPht1;10* and *TaPht1;11* was significant up‐regulated in roots of Pi‐deficient‐grown wheat plants, however *TaPht1;1*,* TaPht1;2* and *TaPht1;6* gene expression patterns were not significantly changed in contrast to the hydroponic Pi starvation experiment. In the experimental field set up, the wheat plants were exposed to long term Pi deficiency, with a drastically reduced soil Olsen P concentration (Table 2). This contrasts with the P starvation applied in the hydroponic system in which the plants under high sufficient Pi content are suddenly P‐depleted. In the hydroponic experiment, the *TaPht1.1* and *TaPht1;2* up‐regulation induced by Pi starvation was reversed by day 12 after reaching a peak at day 6. This implies a different regulation under long‐term deficiencies as found in the field experiment, and this may be an indication that some regulators are restricted to short‐term Pi starvation responses. In *Arabidopsis* the induction of 47 of the 80 genes presumed to be associated with transcriptional regulation under Pi deficiency was more pronounced during long‐term Pi deprivation, and only a small number of transcription factor genes overlapped during different stages of Pi deficiency (Misson *et al*. 2005). The differences in the promoter regions, particularly for *TaPht1;10*, suggests involvement of different regulatory factors acting as long‐term Pi starvation regulators for which the corresponding *cis*‐element still needs to be identified. Sisaphaithong *et al*. (2012) and Duan *et al*. (2015) reported an up‐regulation of wheat *Pht1* genes referred to as *Pht1;10* and *Pht1;11* and *Pht1;12* by arbuscular mycorrhizal fungi. Our phylogenetic analysis revealed those three *Pht1* genes as the homoeologous genes of *TaPht1;11* in the A‐, B‐ and D‐genomes. Whether the up‐regulation of *TaPht1;11* in roots grown in Pi‐deficient field plots is related to increased AM colonisation needs to be verified, but the up‐regulation by Pi starvation under hydroponic AM‐free culture impliees a non‐AM related regulation. An induction by AM colonisation, including localisation in root cortical cell containing mycorrhizal cells, was also reported for *TaPht1;8* (Glassop *et al*. 2005). In our experiments, we did not see a general increase in the transcript amount of *TaPht1;8* in roots derived from field or hydroponic culture.

## Conclusion

**Figure 6:**
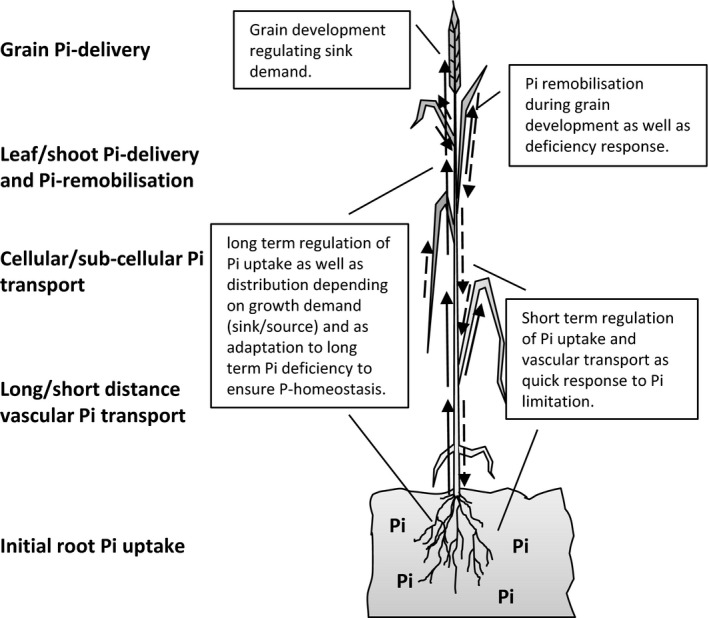
Overview of phosphate transport processes in plants including suggested general regulatory responses.

In conclusion, this study identified 16 A‐ and 14 B‐ and D‐genome genes of the *Pht1* gene family in wheat. The phylogeny implies partly similar orthologous genes to other cereals but also many differences, which may be explained by evolutionary changes in the genome structure. Plant growth and development require different Pi transport processes (Fig. 6), which need to be regulated depending on demand and Pi availability. The two contrasting experimental set‐ups identified different regulatory patterns for the *Pht1* genes. The hydroponic experiment defines a general pattern of regulation of Pht1 gene expression in relation to P deficiencies whereby the long‐term field experiment takes higher complexity into account. Our results suggest differentiation between short‐term and long‐term regulation of gene expression in relation to Pi deficiency. Short‐term regulation enables a rapid response to Pi‐limited conditions to enable rapid Pi uptake and translocation. Long‐term Pi deficiency establishes a long‐term adaptation to Pi deficiency to allow survival and reproduction (Fig. 6). This process requires an up‐regulation of specific *Pht1* genes to enable P homeostasis. In general, the expression patterns of some *TaPht1* genes are important for Pi acquisition, whilst others are likely to be required for Pi translocation from vegetative to generative organs, which seems to be only partly regulated by external Pi availability.

## Supporting information


**Table S1**. Primer sequences used for real‐time qPCR analysis of *TaPht1* transporter expression: Amplicon size (bp), primer concentration (mM) and appropriate annealing temperature (°C).
**Table S2.** Statistical properties (SED, LSD and F‐statistic) of nutritional status analysis of wheat at Broadbalk in 2012 (Fig. S2).
**Table S3.** Statistical properties for *TaPht1* qRT‐PCR expression profiling at Broadbalk field trial 2012 (Fig. 4).
**Table S4.** Location of putative transcription factor *cis*‐regulatory elements in the promoter regions of wheat Pht1 genes. Distances indicated are upstream of the ATG start codon.
**Table S5.** Gene names, accession numbers and related references, chromosome and genome localisation for previously published and identified TaPht1 gene transporter sequences. Primer sequences used for partial TaPht1 cDNA‐PCR cloning (average product size: 500 to 550 bp) including without references accession numbers. Accessions are direct unpublished data submissions.
**Figure S1.** Phylogenetic relationship of the wheat phosphate transporter family 1.
**Figure S2.** Nutritional status of field‐grown wheat at Broadbalk in 2012.

**Data S1.** Wheat Pht1 genomic sequences. List of all available full‐length or partial genomic nucleotides sequences of identified wheat Pht1 phosphate transporter genes, including max 2000 nucleotides of the 5′‐ non‐coding/promoter and 400 nucleotides of the 3′‐genomic non‐coding regions (lowercase). TGAC genomic annotation and IWGSC chromosome scaffold number are given. Coding sequence nucleotides are in uppercase. Intron sequences are lowercase/italic. The identified cloned partial transcripts (Table S5) are highlighted in bold and verified 5′‐ and 3′‐non‐coding sequences are grey‐shaded including accessions.
