# Supplementary material for: Identification and expression profiling of Pht1 phosphate transporters in wheat in controlled environments and in the field
Source: Plant Biol (Stuttg). 2017 Dec 19;20(2):374–89. doi: 10.1111/plb.12668 (PMC5887882; doi:10.1111/plb.12668)

**Supplementary Material**

**Identification and expression profiling of Pht1 phosphate transporters in wheat under controlled and field conditions**

Astrid Grün^1^, Peter Buchner^1^, Martin R. Broadley^2^, Malcolm J Hawkesford, ^1, 3^

^1^ Rothamsted Research, Plant Science Department, Harpenden, AL5 2JQ, UK

^2^ Plant and Crop Science Division, School of Biosciences, University of Nottingham, Sutton Bonington Campus, Loughborough, LE12 5RD UK

^3^ Corresponding author: [malcolm.hawkesford@rothamsted.ac.uk](mailto:malcolm.hawkesford@rothamsted.ac.uk)

**Table S1**. Primer sequences used for real-time qPCR analysis of *TaPht1* transporter expression: Amplicon size (bp), primer concentration (mM) and appropriate annealing temperature (˚C).

| **Gene** | **Absolute - real-time qPCR**  **primer (5' → 3'):**  **Forward, reverse** | **bp** | **mM** | **˚C** |
| --- | --- | --- | --- | --- |
| TaPht1;1a/b | TCCAAGGAGAACGTCGGCGA  SCAAACACTTGTGCATGACTCT | 84  ABD | 150 | 58 |
| TaPht1;2a/b | CGACACCATTGCTCCGACTG  TCAARCACACCAACMATGCACG | 78 A,  75 BD | 200 | 58 |
| TaPht1;5 | AGGCAACGGCGCCAATAAAGTC  GTATGCGTGTGTTGCCTTCTCG | 89A, 85DB | 200 | 60 |
| TaPht1;6 | CAGGACGGTGCCCGTGTGA  CCAAACCATGAAAAGCATCCATAC | 108A,  85B  100B | 200 | 60 |
| TaPht1;7 | CAAGTCCTTGGAGGAGATGTC  AGTGTTCACSGACAGTCATCTAG | 128 A  87B  82D | 150 | 60 |
| TaPht1;8 | TCRCTGGAGGAGGTGTCCG  AGTGGTGCACACAGCCTACG | 110  ABD | 150 | 59 |
| TaPht1;10 | CTAACTCTGACGCCCAAGAG  CGGAACTGCTTATGCGTSG | 128A, 127BD | 200 | 58 |
| TaPht1;11 | GCGACCCCAAGCACATGAAG  GATCGGCCCATCGTCTCAG | 100  ABD | 150 | 60 |
| TaPht1;12 | GTGTTCACGCTCATCTTACCAG  AGTAAATAGAGGTGTTCTCACAAG | 117  BD  (A)* | 150-200 | 57 |
| TaPht1;13 | AATGGAGGAAGAMARCGAACCA  ACACTAAAAYCAKCAACSGGGA | 118 A* | 150-200 | 57 |

*Pht1;12 A-genome truncated gene – incomplete; *Pht1;13 BD genome sequence not available.

Table. S2: Statistical properties (SED, LSD and F-statistic) of nutritional status analysis of wheat at Broadbalk in 2012 (Fig. S2).

| nutrient | P | K | Ca | Mg | S | Mn | Fe | Zn | Cu | Mo |
| --- | --- | --- | --- | --- | --- | --- | --- | --- | --- | --- |
| F_10,50_ = | 49.05 | 57.38 | 28.18 | 27.49 | 23.7 | 7.59 | 20.61 | 34.11 | 25.51 | 10.46 |
| p- value | <0.001 | <0.001 | <0.001 | <0.001 | <0.001 | <0.001 | <0.001 | <0.001 | <0.001 | <0.001 |
| SED | 0.16 | 1.63 | 0.24 | 0.046 | 0.16 | 15.8 | 0.26 | 1.06 | 0.34 | 0.17 |
| LDS | 0.32 | 3.27 | 0.48 | 0.092 | 0.32 | 31.8 | 0.13 | 2.13 | 0.67 | 0.35 |

**Table S3**: Statistical properties for *TaPht1* qRT-PCR expression profiling at Broadbalk field trial 2012 (Figure 4).

Predicted means (log_10_-scale) sharing the same letter are not statistically different and were compared using the standard error of difference (SED) on the relevant degrees of freedom (d.f.) and least significant difference (LSD) values at the 5 % level of significance (P≤0.05). Only max SED and max LSD values with the appropriate d.f. are displayed, rather than the individual LSD and SED values for each comparison. Growth stages according to Zadoks et al. (1974).

| **Gene** | **Tissue** | | **Growth stage** | | **P supply** | | **Log_10_-value** | **P supply** | | | | **Log_10_-value** | | **Max SED** | | | **Max LSD** | **d.f** |
| --- | --- | --- | --- | --- | --- | --- | --- | --- | --- | --- | --- | --- | --- | --- | --- | --- | --- | --- |
| **TaPht1;1** | | Root | | 25 | high | | 6.26 **Bb** | | | low | | 6.81 **Ba** | | | 0.35 | | 0.71 | 54 |
|  |  | Root | | 32 | high | | 6.92 **A** | | | low | | 7.35 **A** | | |  | |  |  |
|  |  | Root | | 45 | high | | 6.35 **B** | | | low | | 6.59 **B** | | |  | |  |  |
|  |  | Ear | | 45 | high | | 5.18 **Da** | | | low | | 4.12 **Db** | | |  | |  |  |
|  |  | Ear | | 49 | high | | 4.91 **D** | | | low | | 4.57 **D** | | |  | |  |  |
|  |  | Root | | 65 | high | | 6.65 **AB** | | | low | | 6.78 **AB** | | |  | |  |  |
|  |  | Ear | |  | high | | 5.38 **C** | | | low | | 5.96 **C** | | |  | |  |  |
|  |  | Root | | 75 | high | | 6.93 **A** | | | low | | 6.61 **A** | | |  | |  |  |
|  |  | Rachis | |  | high | | 5.20 **CD** | | | low | | 5.13 **CD** | | |  | |  |  |
|  |  | Glume | |  | high | | 6.39 **B** | | | low | | 6.03 **B** | | |  | |  |  |
|  |  | Grain | |  | high | | 5.52 **C** | | | low | | 5.82 **C** | | |  | |  |  |
| **TaPht1;2** | | Root | | 25 | mean | | 6.58 **B** | | |  | | |  | 0.209 | | | 0.42 | 51 |
|  |  | Root | | 32 | mean | | 6.98 **A** | | |  | | |  |  | | |  |  |
|  |  | Root | | 45 | mean | | 5.89 **C** | | |  | | |  |  | | |  |  |
|  |  | Ear | | 45 | mean | | 3.64 **F** | | |  | | |  |  | | |  |  |
|  |  | Ear | | 49 | mean | | 5.52 **C** | | |  | | |  |  | | |  |  |
|  |  | Root | | 65 | mean | | 6.72 **AB** | | |  | | |  |  | | |  |  |
|  |  | Ear | |  | mean | | 4.94 **E** | | |  | | |  |  | | |  |  |
|  |  | Root | | 75 | mean | | 6.74 **AB** | | |  | | |  |  | | |  |  |
|  |  | Rachis | |  | mean | | 5.82 **C** | | |  | | |  |  | | |  |  |
|  |  | Glume | |  | mean | | 6.1 **C** | | |  | | |  |  | | |  |  |
|  |  | Grain | |  | mean | | 5.35 **D** | | |  | | |  |  | | |  |  |
| **TaPht1;5** | | Root | | 25 | mean | | 5.67 **A** | | |  | | |  | 0.43 | | | 0.86 | 48 |
|  |  | Root | | 32 | mean | | 5.36 **AB** | | |  | | |  |  | | |  |  |
|  |  | Root | | 45 | mean | | 5.02 **B** | | |  | | |  |  | | |  |  |
|  |  | Ear | | 45 | mean | | 3.88 **C** | | |  | | |  |  | | |  |  |
|  |  | Ear | | 49 | mean | | 5.15 **AB** | | |  | | |  |  | | |  |  |
|  |  | Root | | 65 | mean | | 5.59 **AB** | | |  | | |  |  | | |  |  |
|  |  | Ear | |  | mean | | 3.96 **C** | | |  | | |  |  | | |  |  |
|  |  | Root | | 75 | mean | | 5.08 **B** | | |  | | |  |  | | |  |  |
|  |  | Rachis | |  | mean | | 4.74 **B** | | |  | | |  |  | | |  |  |
|  |  | Glume | |  | mean | | 4.99 **B** | | |  | | |  |  | | |  |  |
|  |  | Grain | |  | mean | | 4.93 **B** | | |  | | |  |  | | |  |  |
| **TaPht1;6** | | Root | | 25 | high | | 6.01**DE** | | | | low | 6.93 **A** | | | 0.27 | | 0.52 | 60 |
|  |  | Root | | 32 | high | | 5.83 **E** | | | | low | 6.4 **CD** | | |  | |  |  |
|  |  | Root | | 45 | high | | 6.49 **BC** | | | | low | 6.73 **AB** | | |  | |  |  |
|  |  | Ear | | 45 | high | 6.22 **CDE** | | | | | low | 3.78 **F** | | |  | |  |  |
|  |  | Ear | | 49 | high | | 6.03 **CDE** | | | | low | 6.67 **AB** | | |  | |  |  |
|  |  | Root | | 65 | high | | 6.39 **CD** | | | | low | 6.89 **A** | | |  | |  |  |
|  |  | Ear | |  | high | | 6.25 **CD** | | | | low | 6.27 **CD** | | |  | |  |  |
|  |  | Root | | 75 | high | | 6.72 **AB** | | | | low | 6.58 **AB** | | |  | |  |  |
|  |  | Rachis | |  | high | | 6.11**CDE** | | | | low | 6.64 **AB** | | |  | |  |  |
|  |  | Glume | |  | high | | 6.65 **AB** | | | | low | 6.79 **AB** | | |  | |  |  |
|  |  | Grain | |  | high | | 6.44 **BC** | | | | low | 6.36 **CD** | | |  | |  |  |
| **Gene** | | **Tissue** | | **Stage** | **P supply** | | **Log_10_-value** | | **P supply** | | | | **Log_10_-value** | | **Max SED** | | **Max LSD** | **d.f** |
| **TaPht1;7** | | Root | | 25 | high | | 4.13 **BCD** | | | | low | 4.76 **AB** | | | 0.49 | | 0.98 | 55 |
|  |  | Root | | 32 | high | | 3.82 **D** | | | | low | 4.30 **BCD** | | | |  |  |  |
|  |  | Root | | 45 | high | | 4.07 **BCD** | | | | low | 4.10 **BC** | | |  | |  |  |
|  |  | Ear | | 45 | high | | 4.38 **BC** | | | | low | 2.55 **E** | | |  | |  |  |
|  |  | Ear | | 49 | high | | 3.33 **BC** | | | | low | 5.54 **A** | | |  | |  |  |
|  |  | Root | | 65 | high | | 4.19 **BCD** | | | | low | 4.33 **BC** | | |  | |  |  |
|  |  | Ear | |  | high | | 4.06 **BCD** | | | | low | 4.18 **BCD** | | | |  |  |  |
|  |  | Root | | 75 | high | | 4.29 **BCD** | | | | low | 4.27 **BCD** | | | |  |  |  |
|  |  | Rachis | |  | high | | 3.78 **CD** | | | | low | 3.56 **D** | | |  | |  |  |
|  |  | Glume | |  | high | | 4.26 **BCD** | | | | low | 3.89 **CD** | | |  | |  |  |
|  |  | Grain | |  | high | | 3.69 **CD** | | | | low | 3.56 **D** | | |  | |  |  |
| **TaPht1;8** | | Root | | 25 | mean | | 7.28**AB** | | |  | | |  | 0.28 | | | 0.57 | 56 |
|  |  | Root | | 32 | mean | | 7.35 **A** | | |  | | |  |  | | |  |  |
|  |  | Root | | 45 | mean | | 6.84 **BCD** | | | |  | |  |  | | |  |  |
|  |  | Ear | | 45 | mean | | 5.42 **G** | | |  | | |  |  | | |  |  |
|  |  | Ear | | 49 | mean | | 6.22 **EF** | | |  | | |  |  | | |  |  |
|  |  | Root | | 65 | mean | | 7.26 **AB** | | |  | | |  |  | | |  |  |
|  |  | Ear | |  | mean | | 5.98 **F** | | |  | | |  |  | | |  |  |
|  |  | Root | | 75 | mean | | 6.94 **ABC** | | | |  | |  |  | | |  |  |
|  |  | Rachis | |  | mean | | 6.54 **DEF** | | | |  | |  |  | | |  |  |
|  |  | Glume | |  | mean | | 6.62 **CDE** | | | |  | |  |  | | |  |  |
|  |  | Grain | |  | mean | | 6.58 **DEF** | | | |  | |  |  | | |  |  |
| **TaPht1;10**  A:  P supply | | Root | | mean | high | | 5.54 **b** | | | low | | | 6.16 **a** | 0.303 | | | 0.61 | 38 |
|  |  | Ear | | Mean | high | | 4.03 (**e**) | | | low | | | 3.83 (**e**) | |  | |  |  |
|  |  | Rachis | | 75 | high | | 4.49 (**d**) | | | low | | | 4.68 (**d**) | |  | |  |  |
|  |  | Glume | |  | high | | 4.83(**cd**) | | | low | | | 4.85 (**cd**) | | |  |  |  |
|  |  | Grain | |  | high | | 5.02 **c** | | | low | | | 4.66 **d** |  | | |  |  |
| **TaPht1;10**  B:  tissues | | Root | | 25 | mean | | 5.76 **B** | | |  | | |  | 0.24 | | | 0.49 | 38 |
|  |  | Root | | 32 | mean | | 5.76 **B** | | |  | | |  |  | | |  |  |
|  |  | Root | | 45 | mean | | 5.53**C** | | |  | | |  |  | | |  |  |
|  |  | Ear | | 45 | mean | | 3.75 **G** | | |  | | |  |  | | |  |  |
|  |  | Ear | | 49 | mean | | 4.18 **F** | | |  | | |  |  | | |  |  |
|  |  | Root | | 65 | mean | | 6.14 **A** | | |  | | |  |  | | |  |  |
|  |  | Ear | |  | mean | | 3.86 **G** | | |  | | |  |  | | |  |  |
|  |  | Root | | 75 | mean | | 6.06 **A** | | |  | | |  |  | | |  |  |
|  |  | Rachis | |  | mean | | 4.58 **E** | | |  | | |  |  | | |  |  |
|  |  | Glume | |  | mean | | 4.84 **D** | | |  | | |  |  | | |  |  |
|  |  | Grain | |  | mean | | 4.84 **D** | | |  | | |  |  | | |  |  |
| **TaPht1;11**  A:  P supply | | Root | | Mean | high | | 5.37 **b** | | | low | | | 6.7 **a** | 0.55 | | | 1.12 | 39 |
|  |  | Ear | | Mean | high | | 3.04 **d** | | | low | | | 3.36 **cd** | |  | |  |  |
|  |  | Rachis | | 75 | high | | * | | | low | | | 3.68 |  | | |  |  |
|  |  | Glume | |  | high | | 4.117 **c** | | | low | | | 3.51 **cd** | |  | |  |  |
|  |  | Grain | |  | high | | 5.772 | | | low | | | * |  | | |  |  |
| **Gene** | | **Tissue** | | **Stage** | **P supply** | | **Log_10_-value** | **P supply** | | | | | **Log_10_-value** | **Max SED** | | | **Max LSD** | **d.f** |
| **TaPht1;11**  B: tissues | | Root | | 25 | mean | | 6.08 **A** | | |  | | |  | 0.52 | | | 1.05 | 39 |
|  |  | Root | | 32 | mean | | 5.88 **B** | | |  | | |  |  | | |  |  |
|  |  | Root | | 45 | mean | | 5.24 **C** | | |  | | |  |  | | |  |  |
|  |  | Ear | | 45 | mean | | 2.39 **E** | | |  | | |  |  | | |  |  |
|  |  | Ear | | 49 | mean | | 3.86 **D** | | |  | | |  |  | | |  |  |
|  |  | Root | | 65 | mean | | 6.54 **A** | | |  | | |  |  | | |  |  |
|  |  | Ear | |  | mean | | 3.34 **D** | | |  | | |  |  | | |  |  |
|  |  | Root | | 75 | mean | | 6.42 **A** | | |  | | |  |  | | |  |  |
|  |  | Rachis | |  | mean | | 3.68 **D** | | |  | | |  |  | | |  |  |
|  |  | Glume | |  | mean | | 3.81 **D** | | |  | | |  |  | | |  |  |
|  |  | Grain | |  | mean | | 5.77 **BC** | | |  | | |  |  | | |  |  |
| **TaPht1;1** | | **Root** | | **32** | high | | 6.92 **BC** | | | | low | 7.35 **AB** | | | 0.20 | | 0.43 | 13 |
| **TaPht1;2** | |  |  |  | high | | 6.82 **CD** | | | | low | 7.13 **ABC** | | | |  |  |  |
| **TaPht1;5** | |  |  |  | high | | 5.07 **F** | | | | low | 5.61 **EF** | | |  | |  |  |
| **TaPht1;6** | |  |  |  | high | | 5.83 **E** | | | | low | 6.4 **D** | | |  | |  |  |
| **TaPht1;7** | |  |  |  | high | | 3.82 **H** | | | | low | 4.30 **G** | | |  | |  |  |
| **TaPht1;8** | |  |  |  | high | | 7.17 **ABC** | | | | low | 7.52 **A** | | |  | |  |  |
| **TaPht1;10** | |  |  |  | high | | 5.31 **F** | | | | low | 6.10 **DE** | | |  | |  |  |
| **TaPht1;11** | |  |  |  | high | | 5.35 **F** | | | | low | 6.41 **D** | | |  | |  |  |

**Table S4:** Location of putative transcription factor cis-regulatory elements in the promoter regions of wheat Pht1 genes. Distances indicated are upstream of the ATG-start codon.

| **Pht1-promoter region** | **P1BS-box GNATATNC** | **W-box**  **C/T TGAC C/T** | | **bHLH-binding**  **CA(G/T)(C/A)TG** | **Accession/ data base** |
| --- | --- | --- | --- | --- | --- |
| AtPht1;1  (1 to 2000) | 995-988; 977-970 | | 1806-1811; 1279-1284; 920-925 | 1860 | At5g43350 |
| HvPht1;2  (1 to 2000) | 1103-1095;  364-357 | | 417-412; 340-335 | none | AY187019 |
| TaPht1;1a-4AL  (1 to 2000) | 634-627; 386-379 | | 1031-1026; 438-435; 216-211 | 763-758; 730-725; 683-678 | IWGSC WGA v0.4 chromosome 4A scaffold 95763 |
| TaPht1;1a-4BL  (1 to 2000) | 1624-1617; 645-638; 424-417 | | 1811-1806; 476-471; 262-257 | 1980-1975; 1964-1959; 1341-1336 | IWGSC WGA v0.4 chromosome 4 B scaffold 128405 |
| TaPht1;1a-4DL  (1 to 2000) | 1557-1550; 641-634; 423-416 | | 475-470; 262-257 | 1310-1305 | IWGSC WGA v0.4 chromosome 4D scaffold 4109; scaffold 227604_chrU |
| TaPht1;1b-4AL | 1925-1919; 904-898; 437-431 | | 1528-1523 | none | TRIAE_CS42_4AL_TGACv1_288569_AA0952320 |
| TaPht1;2a-4AL  (1 to 2000) | 1542-1534; 676-669 | | 729-724; 253-249 | 1701-1695; 1057-1052; 180-175 | IWGSC WGA v0.4 chromosome 4A scaffold 95763 |
| TaPht1;2a-4BL  (1 to 2000) | 1621-1613; 392-385 | | 444-439; 221-216 | 1727-1722; 1366-1361  891-885; 858-849  149-144 | IWGSC WGA v0.4 chromosome 4B scaffold 128405 |
| TaPht1;2a-4DL  (1 to 2000) | 1459-1452; 400-393 | | 452-447; 231-226 | 1608-1603; 1251-1246  787-782; 754-749  159-154 | IWGSC WGA v0.4 chromosome 4D scaffold 58635-1 |
| TaPht1;2b-4AL  (1 to 2000) | 634-627; 386-379 | | 438-433; 216-211 | 1032-1027; 586-581 | TRIAE_CS42_4AL_TGACv1_291993_AA0997320 |
| TaPht1;2b-4BL  (1 to 2000) | 740-733; 519-513 | | 571-566; 357-352 | 1434-1429; 1378-1373 | IWGSC WGA v0.4 chromosome 4B scaffold 128405 |
| TaPht1;2b-4DL  (1 to 2000) | 1485-1478; 663-656; 425-418 | | 1940-1935; 477-472; 257-252 | 1798-1793; 1247-1242; 1194-1189; 1176-1171 | IWGSC WGA v0.4 chromosome 4D scaffold 58635-1 |
| TaPht1;3-4AL  (1 to 2000) | 1720-1743 | | 1827-1832; 1216-1221; 1102-1107; 838-842 | 419-424; 1192-1197; 1592-1597 | IWGSC WGA v0.4 chromosome 4A scaffold 104865 |
| TaPht1;3-5BL  (1 to 2000) | none | | 888-893 | 1585-1590; 152-157 | IWGSC WGA v0.4 chromosome 5B scaffold 11948 |
| TaPht1;3-5DL  (1 to 426) | 1761-1768 | | 1399-1404; 1022-1027 | 1143-1148; 144-149 | IWGSC WGA v0.4 chromosome 5D scaffold 1237 |
| TaPht1;4-4AL  (1 to 2000) | none | | none | 296-301 | IWGSC WGA v0.4 chromosome 4A scaffold 104865 |
| TaPht1;4-5BL  (1 to 2000) | none | | none | 230-235 | IWGSC WGA v0.4 chromosome 5B scaffold 11948 |
| TaPht1;4-5DL  (1 to 2000) | none | | none | 1548-1557; 1029-1034; 975-980;627-632;251-256 | IWGSC WGA v0.4 chromosome 5D scaffold 1237 |
| TaPht1;5-4AL  (1 to 2000) | none | | 1691-1686 | 1964-1959; 1767-1762  1323-1318; 470-465; 273-268 | IWGSC WGA v0.4 chromosome 4A scaffold 95763 |
| TaPht1;54BL  (1 to 2000) | 1395-1388; 1267-1260 | | none | 1953-1948  1287-1282; 1027-1025 | IWGSC WGA v0.4 chromosome 4B scaffold 128405 |
| TaPht1;5-4DL  (1 to 2000) | 1559-1552 | | none | none | IWGSC_CSS_4DL_scaff_14338638/ IWGSC WGA v0.4 chromosome 4D scaffold 4109 |
| TaPht1;6-5AL  (1 to 2000) | 1120-1113; 383-386 | | 1653-1648; 1222-1217; 1155-1150; 1100-1095 | none | IWGSC WGA v0.4 chromosome 5A scaffold 6791 |
| TaPht1;6-5BL  (1 to 2000) | 1423-1415; 317-311 | | 1528-1523; 1268-1263; 931-926; 814-809  806-801; 518-513 | 1993-1988  1934-1929  778-773 | TRIAE_CS42_5BL_TGACv1_404641_AA1307160/ IWGSC WGA v0.4 chromosome 5B scaffold 50324-2 |
| TaPht1;6-5DL  (1 to 2000) | 1569-1564; 1011-1004; 309-302 | | 1677-1672; 1604-1599; 1402-1397; 864-859; 856-851 | none | TRIAE_CS42_5DL_TGACv1_434312_AA1433620/ IWGSC WGA v0.4 chromosome 5D scaffold 82635 |
| TaPht1;7-5AL  (1 to 2000) | 389-396; 165-172 | | none | 1530-1535; 1372-1377 | IWGSC WGA v0.4 chromosome 5A scaffold 34444-3 |
| TaPht1;7-4BL  (1 to 2000) | 1970-1977; 1806-1813; 168-175 | | none | 1835-1840; 791-796 | IWGSC WGA v0.4 chromosome 4B scaffold 85840 |
| TaPht1;7-4DL  (1 to 2000) | 1180-1187; 375-382; 164-171 | | 1908-1913; 1736-1741; 1110-1115; 1051-1056 | 1966-1971 | TRIAE_CS42_4DL_TGACv1_342927_AA1125460 |
| TaPht1;8-6AL  (1 to 2000) | 1723-1716; 697-690 | | 596-591 | 1245-1240 | TRIAE_CS42_6AL_TGACv1_473139_AA1528910 |
| TaPht1;8-6BL  (1 to 2000) | 220-213 | | 977-972; 943-938  628-623; 429-424 | 1626-1621  219-214 | TRIAE_CS42_6BL_TGACv1_499360_AA1579490 |
| TaPht1;8-6DL  (1 to 1729) | none | | 1396-1391; 810-805; 599-594 | none | IWGSC WGA v0.4 chromosome 6D scaffold 44363 |
| TaPht1;9-2AS  (1 to 1146) | 746-752 | | none | 850-855; 325-330; 211-216; 93-98 | IWGSC WGA v0.4 chromosome 2A scaffold 909 Chr2A |
| TaPht1;9-2BS  (1 to 2000) | 1092-1099 | | 1649-1654 | 1196-1201; 745-750; 363-368; 248-253; 92-97 | IWGSC WGA v0.4 chromosome 2B scaffold 21713 |
| TaPht1;9-2DS  (1 to 2000) | 801-808 | | none | 1167-1172; 905-910; 351-356; 236-241 | TRIAE_CS42_2DS_TGACv1_179047_AA0603850 |
| TaPht1;10-7AS  (1 to 2000) | none | | 1962-1957; 1103-1098; 75-70 | 1809-1804; 1088-1083 | IWGSC WGA v0.4 chromosome 7A scaffold 21044 |
| TaPht1;10-7BS  (1 to 2000) | none | | 1540-1535 | 435-430 | IWGSC WGA v0.4 chromosome 7B scaffold 109581 |
| TaPht1;10-7DS  (1 to 2000) | 1376-1369 | | none | 1325-1319 | IWGSC WGA v0.4 chromosome 7B scaffold 131382 |
| TaPht1;11-4AL  (1 to 2000) | 525-518; 238-231 | | 718-713; 692-687 | 695-690; 595-590 | IWGSC WGA v0.4 chromosome 4A scaffold 23037-3-1 |
| TaPht1;11-4BS  (1 to 2000) | 515-508; 226-219 | | 1832-1827; 1500-1495; 716-711; 690-685 | 1883-1878; 693-688; 593-588 | IWGSC WGA v0.4 chromosome 4B scaffold 46890 |
| TaPht1;11-4DS  (1 to 2000) | 523-516; 239-232 | | 1088-1083; 1040-1035, 1025-1020; 721-716; 695-690; 691-686 | 698-693; 594-589 | TRIAE_CS42_4DS_TGACv1_362305_AA1178710 |
| TaPht1;12-2AS  (1 to 653) | none | | none | none | TRIAE_CS42_2AS_TGACv1_112554_AA0340780 |
| TaPht1;12-2BS  (1 to 2000) | 318-325 | | 1391-1396; 908-913 | 1971-1976; 1598-1603; 1166-1171; 1156-1161 | IWGSC WGA v0.4 chromosome 2B scaffold 21713 |
| TaPht1;12-2DS  (1 to 953) | 306-313 | | none | none | IWGSC WGA v0.4 chromosome 2D scaffold 42730 |
| TaPht1;13-2AS  (1 to 2000) | 1883-1890; 1068-1076 | | 1915-1922; 18-23 | 1576-1581 | IWGSC WGA v0.4 chromosome 2A scaffold 909 |
| TaPht1;14-4AL  (1 to 2000) | 1083-1090 | | none | 1078-1083; 298-303;  174-179 | IWGSC WGA v0.4 chromosome 4A scaffold 11991 |
| TaPht1;14-1BL | n.d. | | n.d. | n.d. | No sequence available |
| TaPht1;14-7DS  (1 to 2000) | 1067-1074 | | none | 1060-1065; 254-259; 138-143 | IWGSC WGA v0.4 chromosome 7D scaffold 25190-1 |

**Figure S1****:** *Phylogenetic relationship of the wheat phosphate transporter family 1.* A neighbour joining (Saitou and Nei, 1987) unrooted tree was generated from Pht1 transporters coding DNA sequences including max 200 bases 3’non-coding region and for TaPht1;1a/b type genes including 500 bases of the 5’-DNA region with the MEGA6 (v.6.06) software (Tamura et al., 2013) from the multiple alignment (ClustalX version 2.0). The optimal tree with the sum of branch length = 2.20100877 is shown. The percentage of replicate trees in which the associated taxa clustered together in the bootstrap test (1000 replicates) are shown next to the branches (Felsenstein 1985). The evolutionary distances were computed using the p-distance method (Nei and Kumar, 200) and are in the units of the number of base differences per site. The analysis involved 44 nucleotide sequences. The homoeologus *Pht1* genes are shaded dark grey and unclear homeologous genes are shaded in light grey.


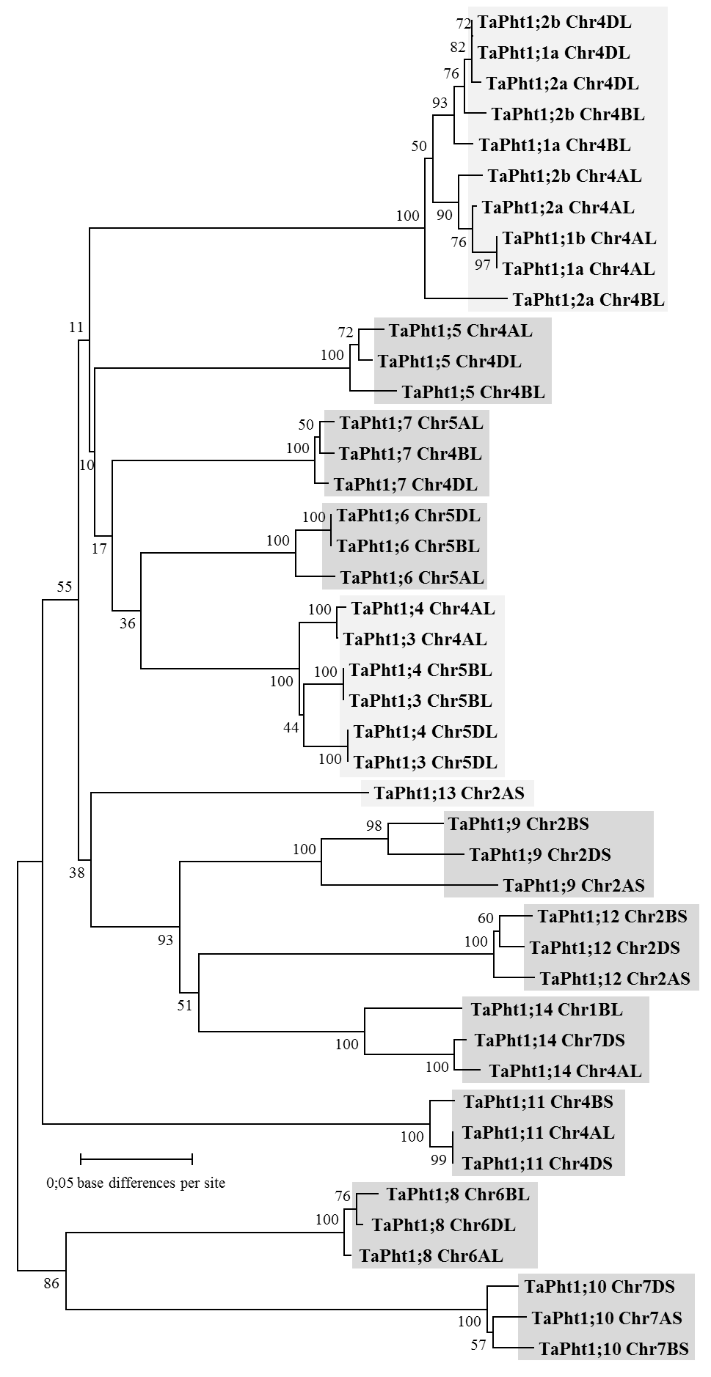


Figure S2: Nutritional status of field-grown wheat at Broadbalk in 2012.

Macro- and micronutrients (ICP-OES analysis) in shoot material sampled at each harvest time point (n=3) from P fertilized (+P: ■) and non-P fertilized (-P: □) plots at Broadbalk, Rothamsted Research, UK in 2012. Statistical properties for all nutritional ions and trace elements are displayed in table S2. Bars sharing the same letter are not statistically different between P availability at different time points during P starvation (P>0.05).


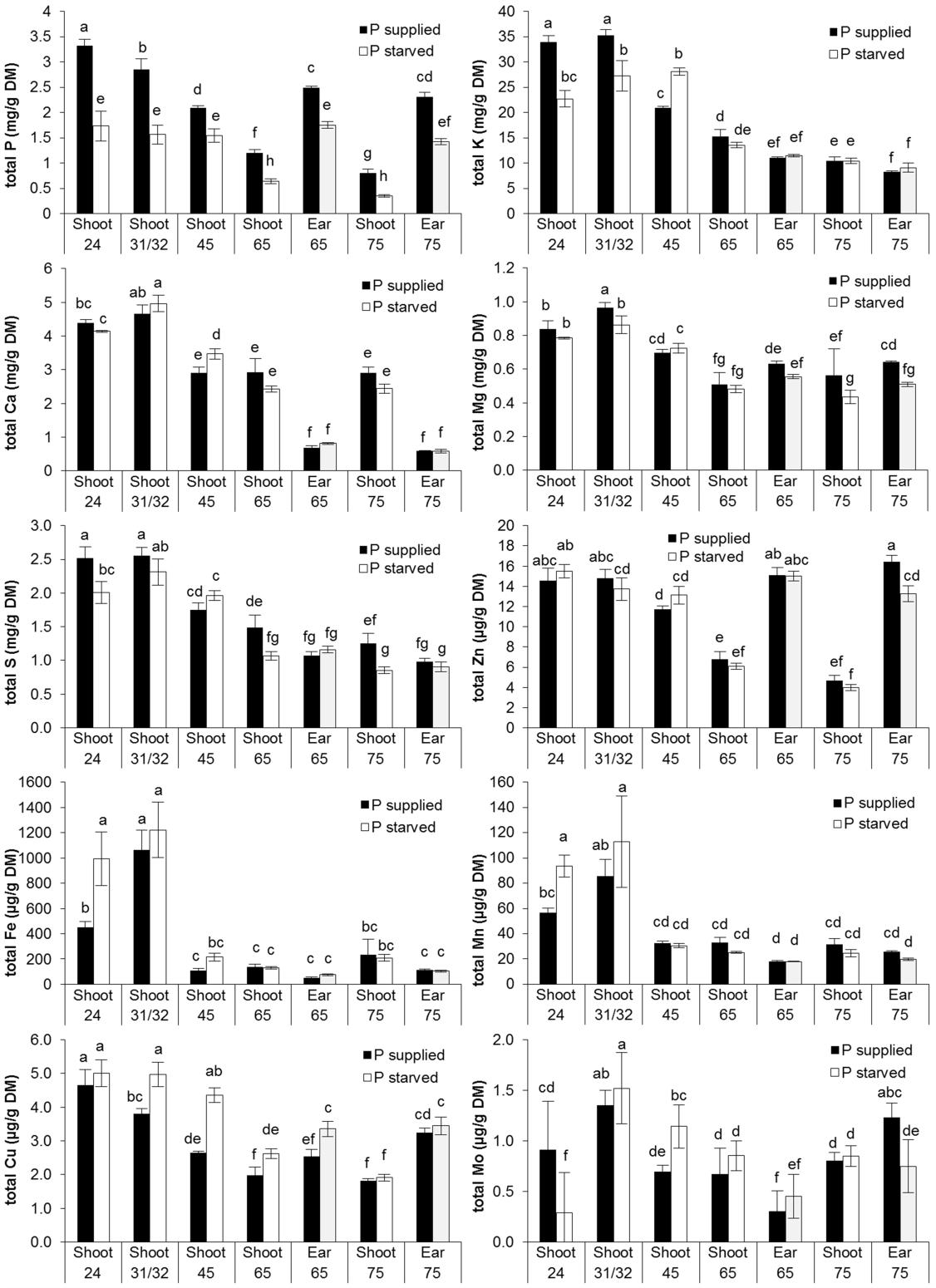

Supplement: Supplementary file 1 — Table S1. Primer sequences used for real‐time qPCR analysis of TaPht1 transporter expression: Amplicon size (bp), primer concentration (mM) and appropriate annealing temperature (°C). Table S2. Statistical properties (SED, LSD and F‐statistic) of nutritional status analysis of wheat at Broadbalk in 2012 (Fig. S2). Table S3. Statistical properties for TaPht1 qRT‐PCR expression profiling at Broadbalk field trial 2012 (Fig. 4). Table S4. Location of putative transcription factor cis‐regulatory elements in the promoter regions of wheat Pht1 genes. Distances indicated are upstream of the ATG start codon. Table S5. Gene names, accession numbers and related references, chromosome and genome localisation for previously published and identified TaPht1 gene transporter sequences. Primer sequences used for partial TaPht1 cDNA‐PCR cloning (average product size: 500 to 550 bp) including without references accession numbers. Accessions are direct unpublished data submissions. Figure S1. Phylogenetic relationship of the wheat phosphate transporter family 1. Figure S2. Nutritional status of field‐grown wheat at Broadbalk in 2012. [file PLB-20-374-s001.docx]
