# Supplementary material for: Identification and expression profiling of Pht1 phosphate transporters in wheat in controlled environments and in the field
Source: Plant Biol (Stuttg). 2017 Dec 19;20(2):374–89. doi: 10.1111/plb.12668 (PMC5887882; doi:10.1111/plb.12668)
Supplement: Supplementary file 2 [file PLB-20-374-s002.docx]

Table S5. Gene names, accession numbers and related references, chromosome and genome localisation for previously published and identified *TaPht1* gene transporter sequences. Primer sequences used for partial *TaPht1* cDNA-PCR cloning (average product size: 500 to 550 bp) including accession numbers. Accessions without references are direct unpublished data submissions.

| Gene | *T. aestivum* accession numbers of previously published and database submitted TaPht1 transporters | Genome/chromosome location of TGAC, IWGSC identified genomic sequences | Primer used for fragment isolation,  cloning and sequencing: Forward, Reverse | Accession numbers  of cloned, partial TaPht1 transporter cDNA fragments |
| --- | --- | --- | --- | --- |
| ***TaPht1;1a*** | AY293828, KJ170110 | Chr 4AL, | GTTCACCGTCGCCTTCATCG,  CACTTGTGCATGACTCTGTGTC | HG764732 |
|  | none | Chr 4BL |  |  |
|  | KJ170111 | Chr 4DL |  |  |
| ***TaPht1;1b*** | none | Chr 4AL | none |  |
| ***TaPht1;2a*** | KJ170112 | Chr 4AL | TTCACCGTCGCCTTCATCGAC,  TCAARCACACCAACMATGCACG | HG764734 |
|  | AK331093 (Kawaura *et al.* 2009) | Chr 4BL |  |  |
|  | AJ344241 (Davis *et al.* 2002), KJ170114 | Chr 4DL |  | HG764733 |
| ***TaPht1;2b*** | none | Chr 4AL | none |  |
|  | none | Chr 4BL |  |  |
|  | none | Chr 4DL |  |  |
| ***TaPht1;3*** | AJ344243 (Davis *et al.* 2002), | Chr 4AL | CCAGCTCATGGGCTTCTTCATG,  CACTAGCACGCACGAGTTGTG |  |
|  | none | Chr 5BL |  | HG764736 |
|  | KJ1170115 | Chr5 DL |  | HG764735 |
| ***TaPht1;4*** | none | Chr 4AL | GCTTCGCCATCCAGCTCATG,  CACGCACGCACGAGTTGTGA |  |
|  | AK333026 (Kawaura *et al.* 2006) | Chr 5BL |  | HG764737 |
|  | KJ1170116 | Chr 5DL |  |  |
| ***TaPht1;5*** | KJ1170118 | Chr 4AL | GTCGTCGGAAGGTTCTGGATC,  CATGCACGTATGCGTGTGTTGC | HG764738 |
|  | KJ1170119 | Chr 4BL, |  |  |
|  | KJ1170120 | Chr 4DL |  |  |
| ***TaPht1;6*** | AF110180, | Chr 5AL, | CAACTCCACCACCTTCATCGTG,  AACYCGACCAGCAACTCTRATG | HG764740 |
|  | KJ1170121 | Chr 5BL |  |  |
|  | KJ1170122 | Chr 5DL |  | HG764739 |
| ***TaPht1;7*** | none | Chr 4AL | CCAGCTCATGGGCTTCTTCATG,  AGTGTTCACSGACAGTCATCTAG | HG764741 |
|  | none | Chr 4DL |  |  |
|  | KJ1170123 | Chr 4BL |  |  |
| ***TaPht1;8*** | KX130944 | Chr 6AL | GATGAAGGGACTCATGCTCGTC,  AGTGGTGCACACAGCCTACG | HG764742 |
|  | AJ830009 | Chr 6BL |  |  |
|  | KJ1170125 | Chr 6DL |  |  |
| ***TaPht1;9*** | AJ344245 (Davis *et al.* 2002) | Chr 2AS | CAATTGCTCGGCTTCACCATGA,  TGACTGATTGGTCTAGATGTGTG  (A genome)/  GCAACCTATTGAGTACAAGACACA (B&D genome) | no transcript verified by RT-PCR |
|  | none | Chr 2BS, |  |  |
|  | none | Chr 2DS |  |  |
| ***TaPht1;10*** | CD871730 (EST) | Chr 7AS | CTGGTACATCGTGCTCTACG,  CGGAACTGCTTATGCGTSG |  |
|  | none | Chr 7BS |  |  |
|  | none | Chr 7DS |  | HG764743 |
| ***TaPht1;11*** | AB753271 (Sisaphaithong *et al.* 2012) | Chr 4AL | GTCACCGTGGCMCTCATCGA,  CGACRTCCTTGTCCACATGC | HG764744 |
|  | AB753270 (Sisaphaithong *et al.* 2012) | Chr 4BS |  |  |
|  | AB753269 (Sisaphaithong *et al.* 2012) | Chr 4DS |  |  |
| ***TaPht1;12*** | KX130942 | Chr 2AS | GACAGAATTGGTCGAATCAAGATG,  CCACATGGTATATTCTTTGGCAC | no transcript verified by RT-PCR |
|  | none | Chr 2BS, |  |  |
|  | none | Chr 2DS |  |  |
| ***TaPht1;13*** | none | chr2 AS | ACGGTGTTCCTCATCGACGTC,  ACTTCTATCAAACGTCTGGTGCC | HG764745 |
| ***TaPht1;14*** | KX130943 | Chr 4AL | CGCATCAAGATCCARCTCATG,  ACACTAAAAYCAKCAACSGGGA | no transcript verified by RT-PCR |
|  | KX154221 | Chr1 BL |  |  |
|  |  | Chr 7DS |  |  |
