# Supplementary material for: Identification and expression profiling of Pht1 phosphate transporters in wheat in controlled environments and in the field
Source: Plant Biol (Stuttg). 2017 Dec 19;20(2):374–89. doi: 10.1111/plb.12668 (PMC5887882; doi:10.1111/plb.12668)
Supplement: Supplementary file 3 — Data S1. Wheat Pht1 genomic sequences. List of all available full‐length or partial genomic nucleotides sequences of identified wheat Pht1 phosphate transporter genes, including max 2000 nucleotides of the 5′‐ non‐coding/promoter and 400 nucleotides of the 3′‐genomic non‐coding regions (lowercase). TGAC genomic annotation and IWGSC chromosome scaffold number are given. Coding sequence nucleotides are in uppercase. Intron sequences are lowercase/italic. The identified cloned partial transcripts (Table S5) are highlighted in bold and verified 5′‐ and 3′‐non‐coding sequences are grey‐shaded including accessions. [file PLB-20-374-s003.docx]

**Supplement wheat Pht1 genomic sequences**

Listing of all available full length or partial genomic nucleotides sequences of identified wheat Pht1 phosphate transporter genes including max 2000 nucleotides of the 5’- non-coding/ promoter and 400 nucleotides of the 3’-genomic non-coding regions (lower case). TGAC genomic annotation and IWGSC chromosome scaffold number are given. Coding sequence nucleotides are in uppercase. Intron sequences are lowercase/ italic. The identified cloned partial transcripts (Tab. 1) are highlighted in bold and verified 5’- and 3’-non-coding sequences are grey shaded including accessions.

**TaPht1;1a Chr4AL**

TRIAE_CS42_U_TGACv1_641994_AA2108990/Traes_4AL_469155166/

IWGSC WGA v0.4 chromosome 4A scaffold 95763

**partial RT-PCR fragment HG764732 (bold)**

atttgacactattaatttcttcattgagctgtcattgacaatttcatttattatgccaagtttgtggttgttgtgatgtcatgctatttacttattttaaagctgttgtagataagagatgttgttatagataagagtttgataaattctctctggatcaatgactgtgctctgatgcatttcgtccaaatatttttaactacgtaaactccaacaccatgagctccactggtaagatgacaggagtggagaccgttatagctagccttaagatggtaagattgtaggagtggagacccttataccgctcttatattagtttagccttagctgcacgccttttataattataactgcattatcgtggagaccaacaccgtgtgcaggccgtgtatactgcaaattagtactacaaactagtgacaagttggcaaggttccaccgcatattcggatcttcacatatttttctcccgccgagaaggttttaaataagctaagtactgtctgcatcagattctgctctcatgatgatctaatgccgcagctatattatgagccgtgatttgccgaaccgactgcaatttggcaaagagaaaccaagaaattaagtgggaggatgcaagtcaacaatttaaatagtacggagcatggctttcaatgctgccctgtctgcttgctgcttaatctgcatatgtttaggagtagtttgttacaaaaaactgtttagtagtttttatgtttaggatgaccagtgtttatatggctgctgcatgctatttgtactgagtgttatctgaattctgaagttattttcctttgtttccgtgacaactatttcatgcacctaatccttcatgtaaatttcttcatgtttcctatttacaaccaaacaacttttaatacatatcccttgtttgcagttcatgtagaattcaagtcaaataacacaaagtacaaaacatgtttaacacaatccacctaatccttcaagaaaatttctatgttttccctgtgctagaaccaagcgatttctattcagattcccatgttctctattcatgtagaattcaaaattgtatgacatcacaattctacatttttggctatgcatttttagtatcacgtggaccaacgagacccttaatatgtatgtttatttgtttgtatactacaaacaggactctaaatgttctgaaacatgtggctgctgcattctattttgttctgaagcgtatctgaattctgaaattgggtttagcaagtgttgaagaacaatttcaaacattgtatttgtatgacaagctatctcagaaatactaaaaagaccgtatgtggctatgtctgaggacctagcactgcatatccatttatcatcgaaattctgtattttcttgctttcctcttggcctactgaaacctgcggtgcgcttaccttcttacgcccgtaccatgtaggtacggaggagagacaaattaactccatgcttgtagtgagaacataagatacagatcatcgtatgatcttgaccttttctcccaaagcttgcttgtgtcaggacagtttgttcctttcaggtatattcccagcataaccatgatctggtttcagtatttttctccacacaattctatagtaccgaaactcaaaggtcaaacctgaaaactaaagttggcatttcttaacaatccttctaataatagaaatgtacataactgtagctctcaaggcaagacatttgacccttgcgtatatttttgtaagaaccatcctgatatgtacaatccgttgtacttgtaagcgatgccgtaataggcaattttatacttacaactagcaatgttgaattggcatactacaatatctccttacctgtatgccttcaccctgaattatgcctatatatacgtagcagaaacacctaacaaagcacacaagctagagagcaagagaagaaagatagaagggggaagagtttagctgagagatcgccggcggccATGGCGACTGAACAGCTCAACGTGTTGAAAGCGCTCGACGTTGCCAAGACGCAGCTGTACCATTTCAAGGCCGTCGTGATCGCCGGCATGGGCTTCTTCACGGACGCCTACGACCTCTTCTGCATCGCCCTCGTCACCAAGCTGCTGGGGCGCATCTACTACACCGACCCTGCCCTCAACGAGCCTGGCCACCTCCCGGCAAACGTGTCGGCCGCCGTGAACGGCGTGGCCCTATGCGGCACACTTGCCGGCCAGCTCTTCTTCGGCTGGCTCGGTGACAAGCTCGGCCGCAAGAGCGTCTACGGCTTCACGCTCATCCTCATGGTCCTCTGCTCCATCGCGTCTGGGCTCTCGTTTGGACACGAGGCCAAGGGCGTAATGGGGACGCTATGTTTCTTCCGCTTTTGGCTCGGCTTCGGCGTCGGCGGCGATTATCCTCTCAGCGCCACCATCATGTCGGAGTATGCTAACAAGAAGACCCGCGGCACCTTTATCGCCGCCGTGTTTGCCATGCAGGGGTTTGGCATCCTATTTGGTACTATCGTCACAATCATCGTCTCGTCCGCATTCCGACATGCATTCCCTGCACCGCCATTCTACATCGACGCCGCGGCGTCCATCGGCCCGGAGGCCGACTACGTGTGGCGCATCATCGTCATGTTCGGCACCATCCCGGCTGCCCTGACCTACTACTGGCGCATGAAGATGCCCGAAACTGCGCGGTACACGGCACTCATCGCCGGCAACACGAAGCAAGCCACATCAGACATGTCCAAGGTGCTCAACAAGGAGATCTTAGAGGAGAACGTCCAGGGTGAGCGGGCCACCGGTGATACCTAGGGCCTCTTCTCCCGACAGTTCATGAAGCGCCACGGGGTGCACTTGCTAGCGACCACAAGCACTTGGTTCCTACTCGATGTGGCCTTCTACAGCCAGAACCTATTCCAGAAGGACATCTTCACCAAGATCGGGTGGATCCCGCCGGCCAAGACTATGAATGCATTGGAGGAGTTGTACCGCATCGCCCGCGCCCAAGCGCTCATCGCGCTCTGTGGCACCGTGCCTGGCTACTG**GTTCACCGTCGCCTTCATCGACATCATTGGAAGGTTTTGGATCCAGCTCATGGGATTCACCATGATGACCATTTTCATGCTAGCAATCGCCATACCGTACGACTACTTGGTGAAGCCAGGGCACCACACCGGTTTCGTCGTGCTCTACGGGCTCACTTTCTTCTTCGCCAACTTCGGCCCCAACAGCACAACTTTCATCGTGCCAGCTGAGATCTTCCCTGCGAGGCTCCGGTCCACATGCCACGGTATCTCTGCCGCTACTGGTAAGGCAGGCGCGATCATCGGCGCGTTCGGGTTCCTGTATGCATCGCAGGACCAGAAGAAGCCCGACACCGGCTACTCACGGGGAATTGGCATGCGCAACTCGCTCTTCGTGCTCGCAGGCACAAACTTCCTGGGCCTGCTCTTTTCCCTGCTGGTGCCGGAGTCCAAGGGCAAGTCGCTCGAGGAGCTCTCCAAGGAGAACGTCGGCGACGATGGCATCGAAGCTTAGgctggtgtacatctccggtgacacagaatcatgcacaagtg**tttgctttttcatttcagcgtttctttccttctgtgtggccacttactgaatttgaggcgtcgcttgccgttcgtgtgtagctgtttatggctgggttttccctaaacctagcattgcatttttgtggtgcggctctgtattcgtttgtgaagaattgtcaataaaataccccgttcattagatttgatttcaagagatcacctcttgcctgtagtttatttttttttaaaacgaaggctcaagaaaagctcggctttaaattaacaacgaaaatgttaacgcccacacgtgtgggcgttaaccaactcgtccacacgcctctatcactgtccagtaacttctgcacgaatcttggcat

**TaPht1;1a Chr4BL**

TRIAE_CS42_4BL_TGACv1_320302_AA1034400/ IWGSC WGA v0.4 chromosome 4 B scaffold 128405

aatgtttttctgagtgggagcatctgagcctgggctcagatgcggattttcgcaaggtcactattcacactattttagccaaaaatttgtctttttgaaaagaagtcaaaggggattttctttttttggaatttttctcacaagtaaaatggaaggtcaagtttatttcaaaaatattttcagaattttttgactttttgttgaattactaattattattattcatatagggtgtagatacgcccagagaaccaaacgtccgcttccacgccctttataattataactgtattatcgtggagaccaacaccgtgtgaaagctgtgtatactgcaaattagtactacaaactagtgacaagttggcaaggttccaccgcatatcccccgcaaaaaaaaaaaaaaaagaaggttccaccgcataatcggatcttcacatatttttctcccgccgagaatgttttaaataggctaagtactgtctgcatcaggttctgctctcacgatgatataatgccgcagctatattatgagctgtgattcgccggccggaccgattgcaatttggcaaagagaaacgaagaaagtgggaggatgcaagacaacaatttaaatagtacggagcacggctttccatgctgtgctcttgctgcttaatctgcatatgtttaggagtagtttgttacaaaaaactgtttagtactgtagttcatatttcaaatgaccaatgtttatatggctgctgttgctatttgtactgagcgttgtctgaattctgaagttattttcctttgtttctgtgacaaccatttcatgcacctaatccttcatgaaaatttcttcatgtttcctatattagaaccaaacaacttttaatacatatcccttgtttgcagttcatgtagaattcaagtcaaataacacaaagtacaagacatgtttaacaccatccacctaatccttcaacaaaatttcctaatcctttaagaaaaattctaggtttttcctgtgctagaaccaagcaatttctatgcagattcccgtgttctctattcatgtagaattcaaaattgtatggcatcacaattctacatttttggctatgcatttttagtatcacgtggaccaacgagacccttaatctttatgtttatttgtttgtatactatatataaaaaactataaatgttctgaaacatgtgggtggtgcattctattttgttctgaaacgtatctgaattctgaaattgggtttagcaagcgttgaagaacaatttcaaacatcgtatttgtatgacaagctatctcaaaaatactgaaaagaccgtatgtggctatgtctggaggacctagcactgcatatccatttatcatcaaaattctgtattttcttgctttcctcttggcctaccgaaaccggcggtgcgcttaccttcttcctgccatgccatgtaggtacggaggagagacaaatcaccaaatccatgcttgtagtgagaacataagatacagatcatcgtatgatcttgaccttttctcccaaagcttgcttgtgtctgtacagtctgttcctttcaggtatattcgcagcataaccatggtctggtttcagtattttcctccacacaattctatagtgcggaaactcaaaggtcaaacctgaatactaaaagttggcatttcttaacaatagttctaacaatagaaatgtacatagctgttgctgtcaaggcaagacatttgacccttgggtatatttttgtaggaaccatcctgatatgtacaatccgttgtacttgtaagcgatgccgtaataggcaattttatacttacaactagcaatgttggattggcatagtacattatctccttgcctttatgccttcaccgtgaattatgcctatatatacgtagcagaagcacctaacaaagcacacaggctagagagcaacagaagaaagatagaaaggagcagagtttagctgacagctcgccggcggccATGGCGACTGAACAGCTCAACGTGTTGAAAGCACTGGACGTTGCCAAGACGCAGTTGTACCATTTCAAGGCGGTCGTGATCGCCGGCATGGGCTTCTTCACGGACGCCTACGACCTCTTCTGCATCGCCCTCGTCACCAAGCTGCTGGGGCGCATCTACTACACCGACCCTGCCCTCAACGAGCCCGGCCACCTCCCGGCAAATGTGTCGGCCGCCGTGAACGGCGTGGCCCTGTGTGGCACACTTGCCGGCCAGCTCTTCTTCGGCTGGCTCGGTGACAAGCTCGGCCGCAAGAGCGTCTACGGCTTCACGCTCATTCTCATGGTCCTCTGCTCCATCGCGTCTGGGCTCTCGTTTGGACACGAGGCCAAGGGCGTAATGGGCACGCTATGTTTCTTCCGCTTCTGGCTCGGCTTCGGCGTCGGCGGTGACTACCCTCTGAGCGCCACAATCATGTCGGAGTATGCTAACAAGAAGACCCGCGGCACCTTTATCGCCGCCGTGTTTGCCATGCAGGGGTTTGGCATCCTATTTGGTACTATCGTCACCATCATCGTCTCGTCTGCATTCCGACATGCATTCCCTGCACCGCCATTCTACATTGACGCCGCAGCGTCCATTGGCCCAGAGGCCGACTACGTGTGGCGCATCATCGTCATGTTCGGCACCATCCCGGCCGCCCTGACCTACTACTGGCGCATGAAGATGCCCGAAACTGCGCGGTACACAGCACTCATCGCCGGCAACACGAAGCAAGCCACATCAGACATGTCCAAGGTGCTCAACAAGGAGATCTCAGAGGAGGATGTGCAGGGTGAGCGGGCCACTGGTGATACATGGGGCCTCTTCTCCCGACAGTTCATGAAGCGTCACGGGGTGCACTTGCTAGCGACCACAAGCACTTGGTTCCTGCTCGATGTGGCCTTCTATAGCCAGAACCTGTTCCAGAAGGACATCTTCACCAAGATCGGGTGGATCCCGCCAGCCAAGACTATGAATGCATTGGAGGAGTTGTACCGCATCGCCCGCGCCCAAGCGCTCATCGCGCTCTGCGGCACCGTGCCCGGCTACTGGTTCACCGTCGCCTTCATCGACATCATTGGGAGGTTTTGGATCCAGCTTATGGGATTCACCATGATGACCATTTTCATGCTCGCAATCGCCATACCTTACGACTACTTGGTGAAGCCAGGGCACCACACCGGCTTCGTCGTGCTCTACGGGCTCACTTTCTTCTTCGCCAACTTCGGGCCCAACAGCACGACCTTCATTGTGCCAGCCGAGATTTTCCCTGCGAGGCTCCGATCCACATGTCATGGTATCTCTGCCGCTACCGGTAAGGCAGGCGCGATCATTGGCGCGTTCGGGTTCCTGTATGCGTCCCAGGACCAGAAGAAGCCCGAGACCGGTTACTCACGAGGAATCGGCATGCGCAACGCACTCTTCGTGCTCGCAGGCACAAATTTCCTGGGCCTGCTCTTTTCCCTGCTGGTGCCGGAGTCCAAGGGCAAGTCACTGGAGGAGCTCTCCAAGGAGAACGTCGGCGACGATGGCATCGAAGCTTAGgctggtgtacatccggagacacagagtcatgcacaagtgtttggattttcattgcagcgtttcttttcttctgtgtggtcgcttgctgaatttgtggggtcgcttgccgtacgtgtgtagctgttgcatttttgtggtggtgctctgtattcatttgtaaagaattgccagtaaagtatcccgttcatttgatttgattccaagagatcacctgttgcatgcaagacgagatttctgaaatgtttggcctggaagaaagatatagggttccagagaaaaaaaatctcttcacaaaatgaaagtaatttacaagcaaattacttaccacatcaaatcccctacaagtttatgcgctgctggctacccgctcgctgaggcctctaccgctcgcgcctcactg

**TaPht1;1a Chr4DL**

TRIAE_CS42_4DL_TGACv1_356725_AA1157450.1 partial/ IWGSC WGA v0.4 chromosome 4D scaffold 4109 and scaffold 227604_chrUn

ttatttcaaaaatagtttcagaattttttgactttttgttgaattactaattattatttttcatatagggtgtagatacgcccagagaaccaaacgtccgcttccgcaccctttataattataattataactgtattatcgtggagaccaacaccgtgtgaaagctgtgtatactgcaaattagtactacaaactagtgacaagttggcaaggttccaccgcataatcggatcttcacatattattctcccgccgagaatgttttaaataagctaagtactgtctgcatcaggttctggcctcacgatgatctaatgccgcagctatattatgagctgtgattcgccggaccgattgcaatttggcaaagagaaacgaagaaagtgggaggatgcaagacaacaatttaaatagtacggagcacggctttcaatgctgtgctcttgctgcttaatctgcatatgtttaggagtagtttgttacaaaaaactgtttagtactttagtttatatttcagatgaccaatgtttatatggctgctgttgctatttgtactgagcgttgtctgaattctgaagttattttcctttgtttctgtgacaactatttcatgcaccaaaatttcttcatgtttcctatgttagaaccaaagattaaaatcgcgggcaaagggaaatagcggaaggaaagtttgccgcgatcgttaaggaagaaacggcgcgatctccggcgattgcggcgcgatctcccgcgattctgggaaggaattaaatcgcggggctgatttcacgcggagatagcggcgtgatagcggtcttttcgcccgcgatttaaaactttgttagaaccaaacaacttttaatacatatcccttgtttgcagttcatgtagaattcaagtcaaataacacaaagtacaagacatgtttaacaccatccacctaatccttcaaaaaaatttcctaatcctttaagaaaatttctacgtttttcctgtgctagaaccaagcaatttttatgcagattcccgtgttctctattcatgtagaactcaaaattgtatggcatcgcaattctacatttttggctatgcatttttagtatcacgtggaccaacgagacccttaatctgtatgtttatttgcttgtatactatataaaaaactatcaatgttttgaaacatgtgggtgctgcattctattttgttctgaaacgtatctgaattctgaaattgggtttagcaagcgttgaagaacaatttcaaacatcgtatttgtatgacaagctatctcagaaatactgaaaagaccgtatgtggctatgtctggaggacctagcactgcatatccatttatcatcaaaattctgtattttcttgctttcctctggcctactgaaaccggcggtgcgcttaccttcttcctgccataccatgtaggtacggaggagagacaaattaactccatctttcaagaaagtcacaaaatccatggttgtagtgagaacataagatacagatcatcgtatgatcttgaccttttctcccaaagcttgcttgtgtctgtacattctgttcctttcaggtatattcccagcataaccatgatctggtttcagtatttttatccccacaattctatagtaccgaaactcaaaggtcaaacctgaaaactaaatgttggcatttcttaacaatccttctaataatagaaatgcacatagctgtagctctcaaggcaagacatttgactctacgtatatttttgtaagaaccatcctgatatgtacaatccgttgtacttgtaagcgatgccgtaataggcaattttatacttacaactagcaatgttggattggcataccacaatattgtatgtcttcaccctggattatgcctgctatatatacgtattagaagcaccgtacaaagcacacaaacaagagagaaccagaagaagacagaagggggcagaagtttagttgagagatcgccggcggccATGGCGACTGAACAGCTCAACGTGTTGAAAGCACTCGATGTTGCCAAGACGCAACTGTACCATTTCAAGGCGGTCGTGATCGCCGGCATGGGCTTCTTCACGGACGCCTACGACCTCTTCTGCATCGCCCTCGTCACCAAGCTGCTGGGGCGCATCTACTACACCGACCCTGCCCTCAACGAGCCCGGCCACCTCCCGGCAAACGTGCGGCCGCCGTGAACGGCGTGGCCCTATGCGGCACACTTGCCGGCCAGCTCTTCTTCGGCTGGCTCGGTGACAAGCTCGGCCGCAAGAGCGTCTACGGCTTCACGCTCATCCTCATGGTCCTCTGCTCCATCGCGTCCGGGCTCTCGTTTGGACACGAGGCCAAGGGCGTAATGGGGACGCTATGTTTCTTCCGCTTCTGGCTTGGCTTCGGCGTCGGCGGCGACTATCCTCTGAGCGCCACCATCATGTCGGAATATGCTAACAAGAAGACCCGCGGCACCTTTATCGCCGCTGTGTTTGCCATGCAGGGGTTTGGCATCCTATTTGGTACCATTGTCACGATCATCGTCTCGTCCGCATTCCGACATGCATTCCCTGCACCGCCATTCTACATTGACGCCGCGGCGTCCATTGGCCCGGAGGCCGACTACGTGTGGCGCATCATCGTCATGTTCGGCACCATCCCGGCCGCCCTGACCTACTACTGGCGCATGAAGATGCCCGAAACTGCGCGGTACACAGCACTCATCGCCGGCAACACGAAGCAAGCCACATCAGACATGTCCAAGGTGCTCAACAAGGAGATCTCAGAGGAGAATGTGCAGGGTGAGCGTGCCACTGGTGATACTTGGGGCCTCTTCTCGCGACAGTTCATGAAGCGCCACGGGGTGCACTTGCTAGCGACCACAAGCACTTGGTTCCTGCTCGATGTGGCCTTCTACAGCCAGAACCTGTTCCAAAAGGACATCTTCACCAAGATCGGGTGGATCCCGCCGGCCAAGACTATGAATGCATTGGAGGAGTTGTACCGCATCGCCCGCGCCCAAGCGCTCATCGCGCTCTGCGGCACCGTGCCCGGCTACTGGTTCACCGTCGCCTTCATCGACATTATTGGGAGGTTTTGGATCCAGCTCATGGGATTCACCATGATGACCATTTTCATGCTCGCAATCGCCATACCTTACGACTACTTGGTGAAGCCAGGGCACCACACCGGCTTCGTCGTGCTCTACGGGCTCACTTTCTTCTTCGCCAACTTCGGCCCCAACAGCACAACCTTCATTGTGCCAGCCGAGATCTTCCCTGCGAGGCTCCGGTCCACATGCCACGGTATCTCTGCTGCTACCGGTAAGGCGGGCGCGATCATCGGCGCGTTCGGGTTCCTGTATGCGTCGCAGGACCAGAAGAAGCCCGAGACCGGCTACTCACGGGGAATCGGCATGCGCAACGCACTCTTCGTGCTCGCAGGCACAAATTTCCTGGGCCTGCTCTTTTCCCTGCTGGTGCCGGAGTCCAAGGGCAAGTCGCTGGAGGAGCTCTCCAAGGAGAACGTCGGCGACGATGGCATCGAAGCTTAGgctggtgtacatccggagacacagagtcatgcacaagtgtttgctttttcattgcagcgtttcttttcttctgtgtggtcgcttgctgaatttgtggggtcgcttgccgtacgtgtgtagctgttgcatttttgtggtggtgctctgtattcatttgtaaagaattgccaataaaatatcccgttcatttgatttgattccaagtgatcacctgttgcatgcaagacgagctttctgaaatgtttggcctggaagaaagatataggattccagagaaaaaaaatctcttcacaaaatcgaagtaatttgcaaacaaattaccacatcaaatcccctacaagtttatgcgctgctggcaacccgctcgctgaggcctctgccgctcgcgcctcactgccat

**TaPht1;1b Chr4AL**

TRIAE_CS42_4AL_TGACv1_288569_AA0952320/ IWGSC WGA v0.4 chromosome 4A scaffold95763

ttacttgaaaccacatagtaatagtaccatatatcggaagttacggtggactctttatggagagtacgccagagactcgtataccttcctagaggcgtggtctccgagctatttcgcgtggatctgcggcgtggttctggctgtcttgctgtagggatgtgctcaaactctgggtgagaaccttgcatcgactttgttgatgtcggcgacagcggcgtctatggacgtcgtcttccttgcaggaggtgccgctgtggagctcctgcacctacggtactttgcattgtggtctccgggtgaaaacctaagatccggctcgccggatcggatgatggtgttgtgtcttcgacagcactacctccttggaggcgtcatcttggagacccaaacgacgacttctgttgctgcgcgtggttgatggtggtgcggccagcttggggccgatggtggtgtcctcttcttcatgtgtttctctttgactgtccttcgcgtggtctccgtcctgttgctgtgcatgctcaagaccctcccatggttgccgttgtgttgcggcgagcttcgggctcttggtgttgtcttggttcacctttgctcaatgacggcgcaatgatgcctccccaacctgctaatcgtttcgatcgcccgtggtggatctctcagtcaaggttcttgttaaggcttgacactcgttgattatggttgatcctttgttgtgccatggggctcgatacgcacgccggtgcggtgtgttaggttgtttgcagagtcttggtattgtgatccctaaacaaaaccccacatctacttgtgtctctcgtggtgttggtcttttgcctgctagctaggtcatgccctctcctaggtggcaatttttttctcatcttctgtaaccttgttacttgtatggttttcggcccggttttcctcataaacggggtcaacttgtttaggtttttgatccggtttcccttataaactggatcaaccaaaccttaaggggtgacccttagctttggcagctttttgagcaatatattcaggtggggatcctctcccccccccccggttcaaaaataaactgatagcacgtctagctgctgcatatcagcacatgcatgtctgcctgtgtacatcccgcgtacacaatcaacaaacatgatgacctccaacttggtataaactacagtcacaactcacacctcatccctaacaacttctcgtgctgatcatccatcaagaaaaaaaattgtgatgccagtcagggaaaatgtccgtcactctaatgtaattaattttttgtgacctttctagttatggcagatttgggacgtgtaaacaacaccatgtcatggtcatctatgctcgtacatgaaactgccaacttgctaattaagtttctcaggcacattcggatcttctcatttttttctctcgtttacaattacaaagtttgatatagcaactaatggtagtagcctactactattcgaagctgtgacccgcttgatgtatttcagcagttcgatatccagcttatgtttatattccatttgaggtatattcctagcataacgatgatgtgggttccaattttatctatatgaaacttcaaagtcaaatctcacactaaacttaaacaagaaatccctagcgcaatccttgtgataattgaaatgcattccaactttgctagctgtagcttaaattaagagaagacattttacattctcgtattctattttatacaagtaccatgtcaaaacatcatgcaatcttgataatgtacggctgtacctacgtgcaatgtcggacattgagattttaatacttacaagtagtaagtgtaccgaagcagaagtgaaataatgtacctttggcatatacatccaccctgaataatggctatatatacgtactagaagcaccttacaaagcacacaacaagagaacaacaaaagaagtacagtaggggcagaagtttagttgagagatcgccggcggccATGGCGACTGAACAGCTCAACGTGTTGAAAGCACTGGACGTTGCCAAGACGCAACTGTACCATTTCAAGGCCGTCGTGATCGCCGGCATGGGCTTCTTCACGGACGCCTACGACCTCTTCTGCATCGCCCTCGTCACCAAGCTGCTGGGACGCATCTACTACACCGACCCTGCCCTCAATGAGCCCGGCCACCTCCCGGCAAACGTGTCAGCCGCCGTGAACGGCGTGGCCCTATGCGGCACACTTGCCGGCCAGCTCTTCTTCGGCTGGCTCGGTGACAAGCTCGGCCGCAAGAGCGTCTACGGCTTCACGCTCATCCTCATGGTCCTCTGCTCCATCGCGTCCGGGCTATCGTTTGGACACGAGGCCAAGGGTGTAATGGGGACGCTATGTTTCTTCCGTTTCTGGCTCGGCTTCGGTGTCGGCGGCGACTATCCTCTCAGCGCCACCATCATGTCGGAGTATGCTAACAAGAAGACCCGCGGCACCTTTATCGCCGCCGTGTTTGCCATGCAGGGGTTTGGCATCCTATTTGGTACTATCGTCACGATCATCGTCTCGTCCGCATTCCGACATGCATTCCCTGCACCGCCATTCTACATCGACGCCGCGGCATCCATTGGCCCGGAGGCCGACTATGTGTGGCGCATCATCGTCATGTTCGGCACCATCCCGGCTGCCCTGACCTACTACTGGCGCATGAAGATGCCCGAAACTGCACGGTACACGGCACTCATCGCCGGCAACACAAAGCAAGCCACATCAGACATGTCCAAGGTGCTCAACAAGGAGATCTCAGAAGAGAACGTCCAGGGTGAGCGGGCCACCGGTGATACCTGGGGCCTCTTCTCCCGACAGTTCATGAAGCGCCACGGGGTGCACTTGCTAGCGACCACAAGCACTTGGTTCCTACTCGATGTGGCCTTCTACAGCCAGAACCTGTTCCAGAAGGACATCTTCACCAAGATCGGGTGGATCCCGCCGGCCAAGACTATGAATGCATTGGAGGAGTTGTACCGCATCGCCCGCGCCCAAGCGCTCATCGCGCTCTGTGGCACCGTGCCTGGCTACTGGTTCACCGTCGCCTTCATCGACATCATTGGGAGGTTTTGGATCCAGCTCATGGGATTCACCATGATGACCATTTTCATGCTAGCAATTGCCATACCGTACGACTACTTGGTGAAGCCAGGGCACCACACCGGTTTCGTCGTGCTCTACGGGCTCACTTTCTTCTTCGCCAACTTCGGTCCCAACAGCACAACTTTCATCGTGCCAGCTGAGATCTTCCCTGCGAGGCTCCGGTCCACATGCCACGGTATCTCTGCCGCTACTGGTAAGGCGGGCGCGATCATCGGCGCGTTCGGGTTCCTGTATGCATCGCAGGACCAGAAGAAGCCCGACACCGGCTACTCACGGGGAATTGGCATGCGCAACTCGCTCTTCGTGCTCGCAGGCACAAACTTCCTGGGCCTGCTCTTTTCCCTGCTGGTGCCGGAGTCCAAGGGCAAGTCGCTCGAGGAGCTCTCCAAGGAGAACGTCGGCGACGATGGCATCGAAGCTTAGgctggtgtacatctccggtgacacagagtcatgcacaagtgtttgctttttcatttcagcgtttctttccttctgtgtggccacttactgaatttgaggcgtcgcttgccgttcgtgtgtagctgtttatggctgggttttccctaaacctagcattgcatttttttggtggtgctctgtattcgtttgtgaagaattgtcaataaaataccccgttcatttgatttgattccaagagatcacctcttgcctgcagtaaaagacgagatttcttggcccggacaaaagatatgggattccagagagaaagaatctcttcacaaaatcgaagtaatttgcatccaaattaccacatgaaatgtccttctctaactgcaacggattttttgttttttactaa

**TaPht1;2a Chr4AL**

TRIAE_CS42_4AL_TGACv1_290760_AA0989060/ IWGSC WGA v0.4 chromosome 4A scaffold 95763

**partial RT-PCR fragment HG764734.1 (bold)**

tccttgttttaaagctgctgtggataagagctgttgtagataagcatttgataaattctcttgggatcaatgattgtgctctttatacatttcgtccaaatatttttaatcacataaactatgacacaggcatgagttgtacgggtaagatggtagaagtggactccattgtagctaatctttcggtatgcaccaattattttggacgatcgcattcgaaattaaactcatacaatagttaatggctgaaaggcgaacttttctgtccctgaggaaacatttcatgttgcaagcggctacagatggaaaatatccctctatagtagttaagctgcgtgatcttcataactatggccgtattatcgtggaggccagtaccaggtcttgctcgcgtataccgggaattactagtattagtgtattattacaaactagtgataagttggcaaggttccacagcatattcggatcttcacatattcttctcccgttgagagggttttaaacaagctaagtactgtcgacatcaggcactgctctcacgatgatctaatgccacagctatgttacgagctgtgattcgcccaactgattgcaatttggcaaagaggatgtaagtcaacagtttaaatagtatggagtacggttttcaatgctgccctgtctgcttgctgctaaatccgcacacgtttagtagtttgttaaaaaaacgcatgtttagtattttttatatttcagacgaccaatgttctaaatgtatggttgctgcgtgctattttgttctgaaccttttctgatttctgaagttgggaattttcctttgtttctgtgactacaatttcgtgcacctattcaagaatatttcctcatttttcctgtgttacagcctcaaatggcatgatatcctacatttccagaaccatgtgaacgaaacagggtgtatcagatgaccaatgtttctgaacatataggcgttgcaaatgttattttgttctgaaactttctgaagtctgaaattagatttagcaactgagcaacaacaatcgcaatggttatatttgtatgactttatgtggctaagtctgcaggacctagcactgcacacctatttatctttgaaattccgtttcttttgctttcccgtgacttgatgaaatcggcggagtgcctaccttcttccagtgatatatagaggagagaaaatattaactccatctctgaagaaatgtcaccaattccatgcttgtagtgatacaactcatcggataatcttgaccttttctcgcaaagctttgcttgtgtctgaacagtttgttcctttcaggtatattcccagcataaccatgatctggtttcaatactattcttcccacaattctatagtactgaaattcggaggtcaaacctgataaaattaaagttggcatcccttaggccttgtttggttcataagtcctaagactttttttagtcccaacttataagtcgcaagtccctaaaaagtccctacatgtttggttctcgagacttataagtctctataaggccatattacaactataagtccctataagtccctccttgagagtcttattccataagtcacaaatgcccactttaagtccttataagtccctcctgtttagtttagatgagacttatatggacttatttaagtccctagaccaataaatctctggaaacaaacaccctcttaatccttctaataatagaaatgtaccatccgttgactctgcgtatatttttgtaagaactatcgtgataatgtacaatccgttgtatttacaagcgatgccgtgcatatgatattttatagttgcaactagcaatgttagattggcataccacaatatatccttacctgtatgttttcacccggaattatgcctatatatacgtcctagaagcgtggagcaaagcacacaacaagagagaaccagaagaagtacagaagtttagccgagagatcgccggcggccATGGCGACTGAACAGCTCAACGTGTTGAAAGCGCTGGACGTTGCCAAGACGCAGCTGTACCATTTCAAGGCGGTCGTGATCGCCGGCATGGGCTTCTTCACGGACGCCTACGACCTCTTCTGCATCGCCCTCGTCACCAAGCTGCTGGGGCGCATCTACTACACCGACCCTGCCCTCAACGAGCCCGGCCACCTCCCGGCAAACGTGTCGGCCGCCGTGAACGGCGTGGCCCTATGCGGCACACTTGCCGGCCAGCTCTTCTTCGGCTGGCTCGGTGACAAGCTCGGCCGCAAGAGCGTCTACGGCTTCACGCTCATCCTCATGGTCCTCTGCTCCATCGCGTCCGGGCTATCGTTTGGACACGAGGCCAAGGGCGTAATGGGGACGCTATGTTTCTTCCGCTTCTGGCTTGGCTTCGGTGTCGGCGGCGACTACCCTCTGAGCGCCACCATCATGTCGGAGTATGCTAACAAGAAGACCCGCGGCACCTTTATCGCCGCCGTGTTTGCCATGCAGGGGTTTGGCATCCTATTTGGTACTATCGTCACGATCATCGTCTCGTCCGCATTCCGACATGCATTCCCTGCACCGCCATTCTACATCGACGCCGCGGCGTCCATTGGCCCGGAGGCCGACTACGTGTGGCGCATCATCGTCATGTTCGGCACCATCCCGGCTGCCCTGACCTACTACTGGCGCATGAAGATGCCTGAAACTGCGCGGTACACGGCACTCATCGCTGGCAACACGAAGCAAGCCACATCAGACATGTCCAAGGTGCTCAACAAGGAGATCTCAGAGGAGAACGTCCAGGGTGAGCGGGCCACCGGTGATACCTGGGGCCTCTTCTCCCGACAGTTCATGAAGCGCCACGGGGTGCACTTGCTAGCGACCACAAGCACTTGGTTCCTACTTGATGTGGCCTTCTACAGCCAGAACCTTTTCCAGAAGGACATCTTCACCAAGATCGGGTGGATCCCGCCGGCCAAGACTATGAATGCATTGGAGGAGTTGTACCGCATCGCCCGTGCCCAAGCACTCATCGCGCTCTGCGGCACCGTGCCAGGCTACTGG**TTCACCGTCGCCTTCATCGACATCATCGGGAGGTTTTGGATCCAGCTCATGGGATTCACCATGATGACCATTTTCATGCTCGCAATCGCGATACCGTATGACTACTTGGTGAAGCCAGGGCACCACACCGGCTTCGTCGTGCTCTATGGGCTCACTTTCTTCTTCGCCAACTTCGGCCCCAACAGCACAACCTTTATAGTGCCAGCCGAGATCTTTCCAGCGAGGCTCCGTTCAACATGCCACGGTATATCGGCTGCAACCGGTAAGGCGGGGGCGATCATCGGCGCGTTCGGGTTCCTGTATGCGTCGCAGGACCAGAAGAAGCCTGAGACCGGGTACTCACGGGGAATCGGCATGCGCAATGCGCTCTTCGTGCTCGCTGGCACAAACTTCCTAGGCCTGCTCTTTTCCCTGCTGGTGCCGGAGTCCAAGGGCAAGTCGCTCGAGGAGCTCTCCAAGGAGAACGTCGGTGACGACGACGCCATTGCTCCGGCTGGTGTCTAGagacattcatgtgtacttggacactcgtgcattgttggtgtgtttga**tttgattttctgcattctgctgttttttgttcctgcgattatttctgaagtcttagcatgtctacctaagaccgtcaggttgatatttagcaacttgtccctcccagttcccggtcacccctctgtcaaatctctgttctgtgtagaagagtccacatctagttgagttgacattatgttctatattacattctttcttaccttatgaaggtagttatgtatatgtatcttgttatgtataaccaaataaaggctgtacctaaatgtctctcaaggtattccgttgggctgaatcgcctagtccttgtgtactctacaattatgactgcttgcagtggaggaacagattgtcc

**TaPht1;2a Chr4BL**

TRIAE_CS42_4BL_TGACv1_320302_AA1034400/ IWGSC WGA v0.4 chromosome 4B scaffold 128405

5’- and 3’-noncoding confirmed by AK331093

cgagccgcgccaattatttcggaggatcacgtttgaaattaaactcatactccctccgttctaaaatagatgactcaactttgtactagctttagtacaaagttgggtcatctattttggaacggagggagtacaatacaatagttgacagatcgaaaggcggaattttccgttcctgaggaaaattgatgactatatggtcacgtttcatgttacaagctgtggtacagatggaaaatatccctctatatattacttaagctgcgtgatcttcataactatgacagtattatcgtggagaccagcaccaggtcatgctcgagtatactacaaattattactactacaaaaactagtgacaagttggcaaggttcccccgcatattcggatcttcacatattcttctcccgttgagagggttttaaataagctaattactgtccacatctatattatgagctgtgattcgcccaactgattgcaatttggcaaagagaaacgaagagagtgggaggttctaagtcaacaatttaaatagtacggagtacggatttcaatgctgccctgtctgcttgctgctaaatctgcatatgttagtagtttgttaaaaaaaacactcagtagtttttattttatatttcagattaccagtgttctaaacgcatggttgctgcatgctattttgttcagaaccttatctaaattctgaagttgggattctcctatgtttctgtgactaccatgtcatgtacctaatccttcaagaatatttcctcattttttctgtcttacaaccaataaaaacaatagcacctatagacttctgagcaatgtcttgctaaatgaaatcaatgtacaattgctcgctatagcgtgaagatagcgttggcttagcgcgatatagcttcgctatagctgattttaagggttgcgctattatgtcagagcatgctattttttgcattggttacaaccaaacaacttctaatacaaatacacccccccccccccccctaaccaaacaacttctaaaacaaatacccccccccccccctcttattcctacatttctagaacaatgagaactaaacattccctttttatgtaacagatgaccaatgttctgaacatatagccgttgcatatgttattttgttctgaaactgtccgaagtacaccctccgtcctgaaatacttgtcagagaaatggatgtattttagttctagatacatccaattttatctatttctgcgacaagtaattccggacggagggagtatgaaattagattttagcaacttagcaacaacaattgaaatcattatatttgtatgcttatgtctgcaggacctagcattgcatatcaatttatggtcgaaattctgtttttttgttgttgctttcctgacttaatgaaatcggcggagtgcctactttcttcctgccataccatgtagttatagagaggtgagaacaaattaaccccatctctgaagaaaggtcaccaaatccatgccggtagtgagaacataagatacaactcaccgtataatcttgaccttttctcgcaaagcttgcttgtgtctatacaatatgtttctttcaggtatattcccagcataaccatgatctggcttcactatttttttatttccacaattctatagtactgaaatttgaaagtcaaacttgataaacttaagtttagcattccttaatccttctaataataacagaaatgtagcccgacatagctgtagctctcaaggcaagacatttgactccgcgtatatttttgtacgaaccatcctgataatgtacaatccgttgttttacaagcgatgccgtacatatgatattatatacgtagttgcaactagcacaatatgtgccttcaccttgcattatttatgcctatatatacgtactagaagcaccaaacatagcacacaacaagagagcagcagaagaagtttagttgagagatcgccggcggccATGGCGACTGAACAGCTCAACGTGTTGAAAGCACTCGACGTCGCCAAGACGCAACTGTACCATTTCAAGGCGGTCGTGATCGCCGGCATGGGCTTCTTCACGGACGCCTACGACCTCTTCTGCATCGCCCTCGTCACCAAGCTGCTGGGGCGCATCTACTACACCGACCCTGCCCTCAACGAGCCCGGCCACCTCCCCGCCAACGTGTCGGCCGCCGTGAACGGCGTGGCCCTATGCGGCACACTTGCCGGCCAGCTCTTCTTCGGCTGGCTTGGCGACAAGCTCGGTCGCAAGAGCGTCTACGGCTTCACGCTCATCCTCATGGTCCTCTGCTCCATCGCGTCCGGGCTCTCGTTTGGACACGAGGCCAAGGGCGTAATGGGGACGCTATGTTTCTTCCGCTTCTGGCTCGGCTTCGGCGTCGGCGGCGACTACCCTCTGAGCGCCACCATCATGTCGGAGTATGCTAACAAGAAGACCCGCGGCACCTTTATCGCCGCCGTGTTTGCCATGCAGGGGTTTGGCATCCTATTTGGTACTATCGTCACGATCGTCGTCTCGTCCGCATTCCGACATGCATTCCCTGCACCGCCATTCTACATTGACGCCGCGGCATCCATTGGCCCGGAGGCCGACTACGTGTGGCGCATCATCGTCATGTTCGGAACAATCCCGGCCGCCTTGACCTACTATTGGCGCATGAAGATGCCCGAAACTGCACGGTACACAGCACTCATCGCCGGCAACACGAAGCAAGCTACATCAGACATGTCCAAGGTGCTCAACAAGGAGATATCAGAGGAGGATGTTCAGGGTGAGCGGGCCACCGGCGATACCTGGGGCCTCTTCTCCCGACAGTTCTTGAAGCGCCACGGGGTGCACTTGCTAGCGACCACAAGCACTTGGTTCCTGCTTGATGTGGCCTTCTACAGCCAGAACCTGTTCCAGAAGGACATCTTCAGCAAGATCGGGTGGATCCCGCCGGCCAAGACTATGAATGCATTGGAGGAGTTGTACCGCATCGCCCGTGCCCAAGCGCTCATCGCGCTCTGCGGCACCGTGCCTGGCTACTGGTTCACCGTCGCCTTCATCGACATCATCGGAAGGTTCTGGATCCAGCTCATGGGATTCACCATGATGACCATTTTCATGCTAGCAATCGCCATACCGTACGATTACTTGGTGAAGCCAGGACACCACACTGGTTTCGTCGTGCTCTACGGGCTCACTTTCTTCTTCGCCAACTTTGGACCCAACAGCACAACCTTCATCGTGCCAGCCGAGATTTTCCCTGCGAGGCTCCGGTCCACATGTCATGGTATCTCTGCCGCTACCGGTAAGGCAGGCGCGATCATCGGCGCGTTCGGGTTCCTATATGCGTCGCAGGACCAGAAGAAGCCCGAGACCGGCTACTCACGGGGAATCGGCATGCGCAACGCACTCTTCGTGCTCGCAGGCACAAACTTCCTGGGCCTGCTCTTTTCCCTGCTGGTGCCGGAGTCCAAGGGCAAGTCGCTCGAGGAGCTTTCAAAGGAGAACGTTGGCGACGACGACGCCATTGCCCCGGGTGGTGTCTAGagacatgcaggcgtacatgcacactcgtgcatggttggtgtgtttgatatgcatttctgtgttctgccatttatattgtttctgcggttactttctgaatttgttgtgttgtaatggtaacaatgtaatgcgttggccgtatcagtgactcaattctcaaaaaaatgtcacctgtattccctcccattgtttcccatgtcatttttttaagataaaaggccacagcccgactttataaataaagccatcaggcagagttaacacagacccaaaagaccagcacgagaacaagcaaagtatcagcccagacaacacgagataatagtctaagtacatggctcccaggccagtacacggctcgcaggccggagatagagagataacgaaagcaagccaccag

**TaPht1;2a Chr4DL**

IWGSC_V3_4DL|IWGSC_V3_chr4DL_scaffold_1932/ IWGSC WGA v0.4 chromosome 4D scaffold 58635-1

**partial RT-PCR fragment HG764733.1 (bold)**

ccaattgtttccgtaagctgtgattgataatctctcatgctgagcttacggttgttgtactgtcacaatctttccttgttttaagctgttgtcgataagagctgttgtagataagggtttgataaatactgtccggatcaatgattgtgttcttgtacatttcatccaaatatttttaatcgcataaactacgacaccatgtgttctacaggtaagatggcaggagtggacacccttattagctaagcttgcgagtcgcgccaattattttggaggatcgcgtccaaaattaaactcatacaatacaatagttgacagatcgaaaggcggacttttccgttcctgaggaaaattgataactatggtcacatttcatgttgcaagctgtggtacagatggaatatatccctctatattacttaagctgcgtgatcttcataactacggcagtattatcgtggaggccagcaccaggtcatgctcgagtatactacaaattattactactacaaaactagtgacaagttggcaaggttcccccgcatattcggatcttcacatattcttcttccgttgagagggttttaaataagctaattactgtccacatctatattatgagctgtgattcgcccaactgattgcaatttggcaaagagaaacgaagagaaaggaggatataagtcaacaatttaaatagtacggcatacggatttcaatgctgccctgtctgcttgctgcgaaatctgcatatgtttagtagtttgttaaaaaaaaacacacttagtagtttttatttcatatttcagattaccaatgttctaaacgtatggttgctgcatgctattttgttcagaaccttatctaaattctgaagttgggaattctcctctgtttctgtgactactatgttatgcacctaatccttcaagaatatttcctcattttttctgtgttacaatcaataaaaaaatagcacgttatagcgttctgagcagtcttgctaaatgaaatcaatgtacaattgttcgctatagtgtggagatagcgttgccttagcgcgatatagcttcgctatagctgattttaagggttgcgctattatgtcatagcacgctattttttgcattggttacaaccaaacaaccccccctcccccccttgttcctacatttctagaacaatgtgaaccaagcatggcctttttatgtaacagatgaccaatgttctgaacatatagcccttgcatatgtattttgttcataaactgtctgaagtatgaaattagattttagcaagctagcaacaacaattgaactaattatatttgtatgcctatgtctgcaagacctagcattgcatccatttatcgtcgaaattctgtttttttgttgttgcttctgatttgatgaaatcggaggagtgcctaccttcttcctgccataccatgtactagttatagagagaacaaattaactccatctctgaagaaaggtcaccaaatccatgccggtagtgagaacataagatacaactcaccgtataatcttgaccttttctcgcaaagcttgcttgtgtctgtacaatatgtttctttcaggtatattcccagcataaccatgatctggcttcattattttttatttccacaattctatagtactaaatttgaaagtcaaacctgataaacttaagtttagcattccttaatccttctaataataacagaaatgtagcccgacatagctgtagctctcaaggcaagacaattgactccgcgtatatttttgtaagaactatcttgacaatgtacaatccgttgttttacaagcgatgccgtacatatgatattatatacgtcgttgcaactagcacaatatgtaccttcaccttgcattatttatgcctatataatagtatatatacgtactagaagcatcgaacaaagcacacaacaagagagcagcagacgaagtttagttgagagatcgccggcggccATGGCGACTGAACAGCTCAACGTGTTGAAAGCACTCGATGTTGCCAAGACGCAACTGTACCATTTCAAGGCGGTCGTGATCGCCGGCATGGGCTTCTTCACGGACGCCTACGACCTCTTCTGCATCGCCCTCGTCACCAAGCTGCTGGGGCGCATCTACTACACCGACCCTGCCCTCAACGAGCCCGGCCACCTCCCGGCAAACGTGTCGGCCGCCGTGAACGGCGTGGCCCTATGCGGCACACTTGCCGGCCAGCTCTTCTTCGGCTGGCTCGGTGACAAGCTCGGCCGCAAGAGCGTCTACGGCTTCACGCTCATCCTCATGGTCCTCTGCTCCATCGCGTCCGGGCTCTCGTTTGGACACGAGGCCAAGGGCGTAATGGGGACGCTATGTTTCTTCCGCTTCTGGCTTGGCTTCGGCGTCGGCGGCGACTATCCTCTGAGCGCCACCATCATGTCGGAATATGCTAACAAGAAGACCCGCGGCACCTTTATCGCCGCTGTGTTTGCCATGCAGGGGTTTGGCATCCTATTTGGTACCATTGTCACGATCATCGTCTCGTCCGCATTCCGACATGCATTCCCTGCACCGCCATTCTACATTGACGCCGCGGCGTCCATTGGCCCGGAGGCCGACTACGTGTGGCGCATCATCGTCATGTTCGGCACCATCCCGGCCGCCCTGACCTACTACTGGCGCATGAAGATGCCCGAAACTGCGCGGTACACAGCACTCATCGCCGGCAACACGAAGCAAGCCACATCAGACATGTCCAAGGTGCTCAACAAGGAGATCTCAGAGGAGAATGTGCAGGGTGAGCGTGCCACTGGTGATACTTGGGGCCTCTTCTCGCGACAGTTCATGAAGCGCCACGGGGTGCACTTGCTAGCGACCACAAGCACTTGGTTCCTGCTCGATGTGGCCTTCTACAGCCAGAACCTGTTCCAAAAGGACATCTTCACCAAGATCGGGTGGATCCCGCCGGCCAAGACTATGAATGCATTGGAGGAGTTGTACCGCATCGCCCGCGCCCAAGCGCTCATCGCGCTCTGCGGCACCGTGCCCGGCTACTGG**TTCACCGTCGCCTTCATCGACATTATTGGGAGGTTTTGGATCCAGCTCATGGGATTCACCATGATGACCATTTTCATGCTCGCAATCGCCATACCTTACGACTACTTGGTGAAGCCAGGGCACCACACCGGCTTCGTCGTGCTCTACGGGCTCACTTTCTTCTTCGCCAACTTCGGCCCCAACAGCACAACCTTCATTGTGCCAGCCGAGATCTTCCCTGCGAGGCTCCGGTCCACATGCCACGGTATCTCTGCCGCTACCGGTAAGGCGGGCGCGATCATCGGCGCGTTCGGGTTCCTGTATGCGTCGCAGGACCAGAAGAAGCCCGAGACCGGCTACTCACGGGGGATCGGCATGCGCAACGCACTCTTTGTGCTCGCAGGCACAAACTTCCTGGGCCTGCTCTTTTCCTTGTTAGTGCCAGAGTCTAAGGGCAAGTCGCTCGAGGAGCTCTCCAAGGAGAACGTCGGCGACGACGACACCATTGCTCCGACTGGTGTCTAGagacatgcaggcgtacatgcacactcgtgcatggttggtg**tgtttgatatgcatttctgttctgccgtttatattgttcctgcggttactttctgaatttgttgtgttgtaatggtgacaatataatgcgttggccgtaacagtgattcaatattcaaaaaagtatcacctgtcttccctcccattgtttccaatgtcaacaattgatggctttaatacataccattaacaattaatttctctcgatttgtcttgttcaacctatgacaagttggacaacacatacacacgagtaatatggttattatccacacaaggagctctatcattgtattccactcttaaacatttagaaggaatgaaaattctacttttgcgcttcagacgttagatcgattaa

**TaPht1;2b Chr4AL**

TRIAE_CS42_4AL_TGACv1_291993_AA0997320/ IWGSC WGA v0.4 chromosome 4A scaffold 95763

ctgagttgtattcgatgagaaaatcacgttcccgaaggtttcattccgtttggactctgtttgatattccttttcttcgtaaccctaaaacaggcaaaaacaacaattctgggctgggccttcggttaataggttagtcccaaaaataatataaaagtggataataaagcccaataatgtccaaaacagtagataatatagcatggagcgatcaaaattatagatacgttggagacatatcaggcgcccccgcctgccttgtggggcgcggtccctggtcgggccggtggagggctggcctctggtggctcctgttgcgcagcggtggtgggatgctagatcagctggatctggcccgtcggcgtcttctcgttcgacggggaggtgaggtgtggtggctctccggtgctgctggcgcggtggcttcatgagaggtgggcgggtcagcgaaggtgcagtgcgtgagggtgctggtggtgttggggatactccgggcgaaagcttggctggtcttgccggccggcggcgtcggcgtctgtggacgtcgtttaacctccttggaggcgtcgttgtggagtccggcatcctcttacgccctcggatcttgctgttcgggtgaaagcctaaggcccggttgggttgggcgacggcgtcgacatcgccgcttccctctttgggcgtcgccttgaagagcttcggatcccggttgcatgcttcatgggctggacgctcgcggcattgtggtcggcaggtgcccgtctggcaatgcggatgttgcgcggcttcctgtttgtgtcaacgatggcggcttcgggcggagtcaggtttgttgatggttcttggtagtcggtctcttccggcgtgttcgaggagtcgccgctctggcgctggctcggcgcaaccccaacgcttttctcctgcaatgctcgtcgacagagtcggagctgcctggtcgccggcgcgtgtggttgactgtttggcatcggccggcgcaagcctcgatgcttcccctcggcggcgtgtgtgcgttcttgtgtgggttgccaggtcgccctgtgtgccttgtttctgcgggccggttgtgacggttttcgtctggttttccgtttattaaccggacaattctcttcgagcttatttaatgaatcgggccctaagagaccgcgtttcaaaaaaaaaaaaccattcccgcaaaaagaaaagaacaatgcgaaccaaacatgccctttttatgtaacagatgaccaatgttctgaacatatagccgttgcatatgttattttgttctgaaactagattttagcaacttagcaacaacagctgcaatcattatatttgtatgcctatgtctacaggacctagcattgcatatccatatatcgtcgaaattctgtttcttttttgctgctttcctctgacttgatgaaatcggcggagtgcctaccttcttcctgccataccatgtagttatagaggagagaacaaattaaccccatctttgaagaaaggccaccaaatccatgccggtagtgagaacataagatacaactcatcgtataatcttgaccttttctcgcaaagcttgcttgtgtgtgtacaatatgtatctttcaggtatattcccagcataaccatgatctggcttcattattttttatttccacaattctctagtactgaaatttgaaagtcaaacctgataaacttaagtttagcattccttagtccttctaataataacagaaatgtagcccgacatagctgtagctctcaaggcaagacatttgactcctcgtatatttttgtaagaactatcctgctaatgtacaatccgttgttttacaagcgatgccgcacatatgatattatatacttgcaactagcacaatatgtgccttcaccctgcatttttatgcctatatatacgtactagaagcaccgaacaaagcacacaacaagagagcaacagaagaagtttaattgagagatcgccggcggccATGGCGACTGAACAGCTCAACGTGTTGAAAGCACTGGACGTTGCCAAGACGCAGCTGTACCATTTCAAGGCGGTCGTGATCGCCGGCATGGGCTTCTTCACGGACGCCTACGACCTCTTCTGCATCGCCCTCGTCACCAAGCTGCTGGGGCGCATCTACTACACCGACCCTGCCCTCAACGAGCCCGGCCACCTCCCGGCAAACGTGTCGGCCGCCGTGAACGGCGTGGCCCTATGCGGCACACTTGCCGGCCAGCTCTTCTTCGGCTGGCTCGGTGACAAGCTCGGCCGCAAGAGCGTCTACGGCTTCACGCTCATCCTCATGGTCCTCTGCTCCATCGCGTCCGGGCTATCGTTTGGACACGAGGCCAAGGGTGTAATGGGGACGCTATGTTTCTTCCGTTTCTGGCTCGGCTTCGGTGTCGGCGGCGACTACCCTCTGAGCGCCACCATCATGTCGGAGTATGCTAACAAGAAGACCCGCGGCACCTTTATCGCCGCCGTGTTTGCCATGCAGGGGTTTGGCATCCTATTTGGTACTATCGTCACGATCATCGTCTCGTCCGCATTCCGACATGCATTCCCTGCACCGCCATTCTACATCGACGCCGCGGCGTCCATTGGCCCGGAGGCCGACTATGTGTGGCGCATCATCGTCATGTTCGGCACCATCCCGGCTGCCCTGACCTACTACTGGCGCATGAAGATGCCCGAAACTGCGCGGTACACAGCACTCATCGCCGGCAACACAAAGCAAGCCACATCAGACATGTCCAAGGTGCTCAACAAGGAGATCTCAGAGGAGAACGTCCAGGGTGAGCGGGCCACCGGTGATACCTGGGGCCTCTTCTCCCGACAGTTCATGAAGCGCCACGGGGTGCACTTGCTAGCGACCACAAGCACTTGGTTCCTACTCGATGTGGCCTTCTACAGCCAGAACCTGTTCCAGAAGGACATCTTCACCAAGATCGGGTGGATCCCGCCGGCCAAGACTATGAATGCATTGGAGGAGTTGTACCGCATCGCCCGCGCCCAAGCGCTCATCGCGCTCTGTGGCACCGTGCCTGGCTACTGGTTCACCGTCGCCTTCATCGACATCATTGGGAGGTTTTGGATCCAGCTCATGGGATTCACCATGATGACCATTTTCATGCTAGCAATCGCCATACCGTACGACTACTTGGTGAAGCCAGGGCATCACACCGGCTTTGTCGTGCTCTACGGGCTCACTTTCTTCTTCGCCAACTTCGGCCCCAACAGCACAACCTTCATCGTGCCAGCTGAGATCTTCCCTGCGAGGCTCCGATCCACATGCCACGGTATCTCTGCTGCTACCGGTAAGGCAGGCGCGATCATCGGTGCATTCGGGTTCCTGTATGCGTCACAGGACCAGAAGAAGCCTGAGACCGGCTACTCACGGGGAATCGGCATGCGCAACGCTCTCTTCGTGCTCGCGGGCACAAACTTCCTGGGTCTGCTCTTTTCGCTGCTGGTGCCGGAGTCCAAGGGCAAGTCGCTGGAGGAGCTCTCCAAGGAGAACGTTGGCGACGATGACTCCATTGCCCCAACTGGTGTCTAGagacgtgcaggtgtacttgcacactcgtgcattatttgtgtgtttgatttgagtttctgtgttctgctgtttgttttgttcatgcaattttctgaacttttagtgttgtaatggcgtgacaatgtaatgcgtaaactgtaaagacaatgattaaattttgagaaaaatatcgtgtgtatctcctctcattatttccatatcatcaataatattgacgacataccgttaacaactaatttttcttgatttgttctgttcagagcgtgcactgaactgttgcctatatgacaagttgacaacacatacacacaagtgatatggttttattatcacataaggagctctatcatgtattcctctctcttatacatttagaaggaaagaaaaatctaccttttgt

**TaPht1;2b Chr4B**

TRIAE_CS42_U_TGACv1_684896_AA2159320/ IWGSC WGA v0.4 chromosome 4B scaffold 128405

agggttcccccgtgccactcgagatgggcgacgcgggggcgggggtctttgaagacaaaatgggattttcttttttttggattttttctcacaagtaaaatggaaggtcaagtttattttaaaaatattttcagaatttgttgactttttgttgaattactaattattatttttcatatagggtgtagatacgcccagagaaccaaaacgtccgcttccgcgccctttataattgtaactgtattatcgtggagaccaacaccgtgtgaaagctgtgtatactgcaaattagtactacaaactagtgacaagttggcaaggctccgccgcataatcggatcttcacatatttttctcccgccgagaatgttttaaataagctaagtactgtctgcatcaggttctgccttcacgatgatctaatgccgcagctatattataagctgtgattcgccggaccgattgcaatttggcaaagagaaacgaagaaagtgggaggatgcaagacaacaatttaaatagtacggagcacggctttcaatgctgtgctcttgctgcttaatctgcatatgtttaggagtagtttgttacaaaaaactgtttagtactgtagtttatatttcagatgaccaatgtttatatggctgctgttgctatttgtactgagcgttgtctgaattctgaagttattttcctttttttctgtgacaactatttcatgcacctaatccttcatgaaaatttcttcatgtttcctatgttagaatcaaacaacttttaatacatatcccttgtttgcagttcatgtagaattcaagtcaaataacacaaagtacaagacatgtttaacaccatccacctaatccttcatgaaaatttgccaatcctttaataaaatttatacgtttttcctatgctagaaccaagcaatttctatgcagattcccgtgttctctattcatgtagaattcaaaattgtatggcatcacaattctacatttttggctatgcatttttagtatcacgtggaccaacgagacccttaatctgtatgtttatttttttgtatactatataaaacactataaatgttctgaaacatgtgggtggtgcattctattttgttctgaaacgtatctgaattctgaaattgggtttagcaagcgttgaagaacaatttcaaacatcgtatttgtatgacaagctatctcaaaaatactgaaaagaccgtatgtggctatgtctggaggacctagcactgcatatccatttatcatcaaaattctgtattttcttgctttcctcttggcctaccgaaaccggcggtgcgcttaccttcttcctgccatgccatgtaggtacggaggagagacaaatcaccaaatccatgcttgtagtgagaacataagatacagatcatcgtatgatcttgaccttttctcccaaagcttgcttgtgtctgtacagtctgttcctttcaggtatattcccggaataaccatgatctggtttcagtattttcctccacacaattctataatgcggaaactcaaaggtcaaacctgaaaacttaaagttggcatttcttaacaatcgttctaacaatagaaatgtacatagctgtagctgtcaaggcaagacatttgacccttgcgtatatttttgtaagaaccatcctaatatgtacaagccgttgtactccctccgttctaaaatagataactcaactttatactaactttaatacaaagttagtataaagttgggtcttctattttgaaacggagggagtacttgtaagcgatgccgtaataggcaattttatacttacaactagcaatgttggattggcatactacaatatctccttgcctttatgccttcaccctgaattatgcctatatatacgtagcagaagcacctaacaaagcacacaggctagagagcaacagaagaaagatagaaaggagcagagtttagctgacagctcgccggcggccATGGCGACTGAACAGCTCAACGTGTTGAAAGCACTGGATGTTGCCAAGACGCAACTGTACCATTTCAAGGCGGTCGTGATCGCCGGCATGGGCTTCTTCACGGACGCCTACGACCTCTTCTGCATCGCCCTCGTCACCAAGCTGCTGGGGCGCATCTACTACACCGACCCTGCCCTCAACGAGCCCGGCCACCTCCCAGCAAACGTGTCGGCCGCCGTGAACGGCGTGGCCCTGTGTGGCACACTTGCCGGCCAGCTCTTCTTCGGCTGGCTCGGTGACAAGCTCGGACGCAAGAGCGTCTACGGCTTCACGCTCATCCTCATGGTCCTTTGCTCCATCGCGTCCGGGCTCTCGTTTGGACACGAGGCcAAAGGCGTAATGGGGACGCTATGTTTCTTCCGCTTCTGGCTCGGCTTCGGCGTCGGCGGCGACTACCCTCTGAGCGCCACCATCATGTCGGAGTATGCTAACAAGAAGACCCGTGGCACATTTATCGCCGCCGTGTTTGCCATGCAGGGGTTTGGCATCCTATTTGGTACTATCGTCATGATCATCGTCTCGTCCGCATTCCGACACGCATTCCCTGCACCGCCATTCTACATTGACGCCGCGGCGTCCATTGGCCCGGAGGCCGACTACGTGTGGCGCATCATCGTCATGTTCGGCACCATCCCGGCCGCCCTGACCTACTACTGGCGCATGAAGATGCCCGAAACTGCGCGGTACACAGCACTCATCCCCGGCAACACGAAGCAAGCCACATCAGACATGTCCAAGGTGCTCAACAAGGAGATCTCAGAGGAGGATGTGCAGGGTGAGCGTGCCACTGGTGATACTTGGGGCCTCTTCTCGCAACAGTTCATGAAGCGCCACGGGGTGCACTTGCTAGCGACCACAAGCACTTGGTTCCTGCTCGATCTGGCCTTCTACAGCCAGAACCTGTTTCAAAAGGACATCTTCACCAAGATCGGGTGGATCCCGCCGGCCAAGACTATGAATGCATTGGAGGAGTTGTACCGCATCGCCCTCGCCCAAGCGCTCATCGCGCTCTGCGGCACCGTGCCCGGCTACTGGTTCACCGTCGCCTTCATCGACATCATCGGCAGGTTTTGGATCCAGCTCATGGGATTCACCATGATGACCATTTTCATGCTCGCAATCGCCATACCTTACGACTACTTGGTGAAGCCAGGGCACCATACCGGCTTCGTCGTGCTCTATGGGCTCACTTTCTTCTTCGCCAACTTCGGCCCCAACAGCACAACCTTTATTGTGCCAGCCGAGATCTTCCCTGCGAGGCTTCGGTCCACATGCCACGGTATCTCTGCCGCTACCGGTAAGGCGGGCGCGATCATCGGCGCGTTCGGGTTCCTGTATGCGTCGCAGGACCAGAAGAAGCCCGAGACCGGCTACTCGCGGGGAATCGGCATGCGCAACGCACTCTTTGTGCTCGCAGGCACAAACTTccTGGGCCTGCTCTTTTCCTTGTTGGTGCCAGAGTCTAAGGGCAAGTCGCTCGAGGAGCTCTCCAAGAAAAACGTCGGCGACGACGACACCATTGCTGCGACTGGTGTCTAGagacatgcatgtgtacttgcacactcgtgcattgttggtgtgcttgatttgcctttttgtattctatttttgttttctttgccgcgaccactttctaaatttctagtgttgtaatggtgacaatgtaatgcgtacactgtaacagtgattcaaatttcaattctgaaaaagtatatcatttgtactccctcccattgtttcccatgtcatcgattatatttaaggttttaaaacatactgtcaatcatattgtcttcttcaacgtgcacttcgatgtagccaatgacaagttagacaacacatacacgcaagtaatatggttattatccacacaaggagctctatcattgtattcctgtcttaaacattgagaaggaaagaaaattctactcccttcgtc

**TaPht1;2b Chr4DL**

IWGSC_V3_4DL|IWGSC_V3_chr4DL_scaffold_2624/ IWGSC WGA v0.4 chromosome 4D scaffold 58635-1

tgttctggattccttctagtgaatattatcaagtagtatgagcgatcatttgcattctcactgactggacggaactttatttatgctatctcacactactaatttcttcattaagctgtcattgataatttcatttcttataccaagtttgtggttgttctgctgccatgctatttacttattttaaagctgttgtagataacagatgttgttatagataagagtttgataaattctctccggatcaatgactttgctcttatacagttcgtccaaatatttttaatcacgtaaactacgacaccatgagttccacaggtatgatggcaggagtggagacccttatagctagccttaagatggtaggagtggagacccttatacagctcttatattagtttagctgcagccctttataattaagactgtattatcgtggagaccaacaccatgtgaaggctgtctacactgcaaattagtactacaaactagtgacaagttggcaaggttccaccgcatattcggatcttcacataattttctcccgccaggaagatcttaaataagctaagtactgtctacatcaggttctgctctcacgatgatctaatgccgcagctatattatgagctgtgattcgctggaccgattgcaatttggcaaagagaaacgaagaaagtgggaggatgcaagtcaacaatttaaatattacggagcgcggctttcaattctgcactcttgttgtttaatctgcatatgtttaggagtagcttgttacaaaaaactgtttagtagtttttatatttcagatgaccaatgttctacatatgactgctgcatgcacctaatatgaattctgaagttattttcctttgtttctgtgacaactatttcatgcacctaatatccttcatgaaaatttcttcatgtttcctgtgttacaacctacaaccaaacaacttctaatacataccccttgttttcagttcatgtaggatttaagtcaaatgacacaaagtacaagacatctttaacaccatgcacctaatccttcaagatttttttttacatttgtcctgtggtacagccaaacaatgtctatacagattcccgtgttctatattcatgtagaattcaaaattatatggtgtcacaattctacatatttgctatgcgtttctagtatcatgtggaccaacgagacccttaatctgtatatgtctattagtttatatatgtaaaatgatctcaaaagttttatttgtatgagtatgacaagctatctcagaaatacgaaagaaaaaaaaagtatgtggctgtcggcaggacctagcactgcatatccatttatcatcgaagttttttttttttccctctggcttactgaaattggcagagtgcctaccttcttcctggcataccatgtaggtatcgaggagagaacaaattaaccccatatttcaagaaaggtcacctgttccgtactagtagggagaacgtaagatacaactcatcgtataatcttgaccttttctcccaaagcttgcttgtgtctgtacattctgttcctttcaggtatattcccagcataaccatgatctggtttcagtatttttatccccacaattctatagtaccgaaactcaaaggtcaaacctgaaaactaaaagttggcatttcttaacaatctttctaataatagaaatgtaccccgacatagctgtagctctcaaggcaagacatttgactctgcgtataatttttgtaagaaccatcctgatatgtacaatccgttgtacttgtaagcgatgccgtaataggcaattttatacttacaactagcaatgttggattggcataccacaatattgtatgtcttcaccctggattatgcctgctatatatacgtattagaagcaccgtacaaagcacacaaacaagagagaaccagaagaagtacagaagggggcagaagtttagttgagagatcgccggcggccATGGCGACTGAACAGCTCAACGTGTTGAAAGCACTCGATGTTGCCAAGACGCAACTGTACCATTTCAAGGCGGTCGTGATCGCCGGCATGGGCTTCTTCACGGACGCCTACGACCTCTTCTGCATCGCCCTCGTCACCAAGCTGCTGGGGCGCATCTACTACACCGACCCTGCCCTCAACGAGCCCGGCCACCTCCCGGCAAACGTGTCGGCCGCCGTGAACGGCGTGGCCCTATGCGGCACACTTGCCGGCCAGCTCTTCTTCGGCTGGCTCGGTGACAAGCTCGGCCGCAAGAGCGTCTACGGCTTCACGCTCATCCTCATGGTCCTCTGCTCCATCGCGTCCGGGCTCTCGTTTGGACACGAGGCCAAGGGCGTAATGGGGACGCTATGTTTCTTCCGCTTCTGGCTTGGCTTCGGCGTCGGCGGCGACTATCCTCTGAGCGCCACCATCATGTCGGAATATGCTAACAAGAAGACCCGCGGCACCTTTATCGCCGCTGTGTTTGCCATGCAGGGGTTTGGCATCCTATTTGGTACCATTGTCACGATCATCGTCTCGTCCGCATTCCGACATGCATTCCCTGCACCGCCATTCTACATTGACGCCGCGGCGTCCATTGGCCCGGAGGCCGACTACGTGTGGCGCATCATCGTCATGTTCGGCACCATCCCGGCCGCCCTGACCTACTACTGGCGCATGAAGATGCCCGAAACTGCGCGGTACACAGCACTCATCGCCGGCAACACGAAGCAAGCCACATCAGACATGTCCAAGGTGCTCAACAAGGAGATCTCAGAGGAGAATGTGCAGGGTGAGCGTGCCACTGGTGATACTTGGGGCCTCTTCTCGCGACAGTTCATGAAGCGCCACGGGGTGCACTTGCTAGCGACCACAAGCACTTGGTTCCTGCTCGATGTGGCCTTCTACAGCCAGAACCTGTTCCAAAAGGACATCTTCACCAAGATCGGGTGGATCCCGCCGGCCAAGACTATGAATGCATTGGAGGAGTTGTACCGCATCGCCCGCGCCCAAGCGCTCATCGCGCTCTGCGGCACCGTGCCCGGCTACTGGTTCACCGTCGCCTTCATCGACATTATTGGGAGGTTTTGGATCCAGCTCATGGGATTCACCATGATGACCATTTTCATGCTCGCAATCGCCATACCTTACGACTACTTGGTGAAGCCAGGGCACCACACCGGCTTCGTCGTGCTCTACGGGCTCACTTTCTTCTTCGCCAACTTCGGCCCCAACAGCACAACCTTCATTGTGCCAGCCGAGATCTTCCCTGCGAGGCTCCGGTCCACATGCCACGGTATCTCTGCCGCTACCGGTAAGGCGGGCGCGATCATCGGCGCGTTCGGGTTCCTGTATGCGTCGCAGGACCAGAAGAAGCCCGAGACCGGCTACTCACGGGGGATCGGCATGCGCAACGCACTCTTTGTGCTCGCAGGCACAAACTTCCTGGGCCTGCTCTTTTCCTTGTTAGTGCCAGAGTCTAAGGGCAAGTCGCTCGAGGAGCTCTCCAAGGAGAACGTCGGCGACGACGACACCATTGCTCCGACTGGTGTCTAGagacatgcaggtgtacttgcacaatcgtgcattgttggtgtgcttgatttgcctttttgtgttctatttttgttttctttgccgcgaccactttctaaatttctagtgttgtaatggtgacaatgtaatgcgtacactgtaacagtgattcaaattccaattctgaaaaaaaataaaaatatcatttgtactccctcccattgtttcccatgtcatcaattatatttaaggttttaatacatactgtcaatcatattgtcttcttcaacgtgcacttcgttgtagccaatgacaagttggacaacacatacacgcaagtaatatggttattatccacacaaggagctctatcattgtattcctctcttaaacattgagaaggaaagaaaattctactccc

**TaPht1;3 Chr4AL**

IWGSC WGA v0.4 chromosome 4A scaffold 104865/ identical to TRIAE_CS42_4AL_TGACv1_289415_AA0970650.1

cggactacaaaatgcatccgtcaccccccatgacatcgttcagtgaattattagggaatattgtttcttgaataagaaataaatgttgcagctctcagacatcaattaacggctccttccgcaccggtgggtacgttgcttcagattgagcacgcttgacacaattgtttgacttgacgttcaaaaactaagcacatcgggctgtaggttcgtgtgaaaggcgcaaggtagacgaattgttaaagcagctagctaagacttgaggagtatatagaatatgcaattatcgttgaatttgcttcttccatttctttttggaactcaacaaatttgcttgttagctatggagtagttgtgagtcatcgaaattaaacaatttaataagggttttgatatctaggttcagatgatatttatcatgacgttgatggagagtgtcgtgtgatgtgtggtgctttcgatttgtctgggatgcccccaccccccccctcctttctcgatggtgtgtggaggattgccaggcgaaagtcctttttttgtggagctccattattccacccccgtggggttgtgggctcatcgggataaatccctagcaggcgacgacggtggttgcgttgcttcctccattggaggtgttgggttcagcacatagcaacatgcatggatctcttatccatcggcggcaatggtgccatgtggttactctttgtgtggctgagggcccggtctcgagaaacaatgtgtgtatgtgttggggggggggtacgacccatgagttgactaaatgtgagaagaagctccagatggagttgggcgggccatggcccattcggccttgcctaagctccgccagtgcttgagccaccagggacgacgagggggcgagggtgttgacttggttgacgcgccccagagcaagcggtggcatgttgaaaccgtggccgtagaaagtgtggtcgacggaccatgcatggtctcgtgtgaggtcattgaggagggctcctccacaacatagtggtattctccgcactcggatatgagtaatgctacaactgcagagggcttagactggttttaatgaggagtgtcatataatagtatcatagcgtatctttttagggtaattgcaatatttttgttgatagtttaccaagctgaccacttggtatattattatagtgtatggtattacatccgtaatgcattgtatcacatgttggtatcatagcggactacatttgttgccatgcataacacaaagtatcacatcatttaatatgatatggtatcatgatatgatactcaagcatctttttcttcatttaattttatgccaactcatcaaaattgcttaattgagatgcataatactacctatgatactcccatcacgggcagccttatcgaagttttacagcctcgactgatgcgggctattttggttggtaatttggaggaaacggggcccaccctaaagcggtgattattgggacgagaaggatgagtccgttaactctgtaaatatcaccttgcaagtcttaacattttgtcttggatgttgatagattgcattcagatgatattatttcccatgccgcatgatatccttgtcaacgatggagcatggaaattccccaggatggtaacaactttacatgcagtgaaacttcatgctcgagggagccaagacaagagaacataataatcagttttgctagaactcatctagatgagatttaatttggtctcattcatcttttataatatggtgctataagatgtgtgtgtgctgaagtgggttgtatctgttcttgttttccaggtgaataataatatatcaaccaaatattcgcggtgacacccagctctcctcgatcggctacttataccagatagccggcccataattcctgcaccacaacacaagaaagtcttgcggccggccggcaccgtcgtctagctcccacgctcgcaccgttgccctgtgcagcagcagcATGGCGCGGTCGGAGCAGCAGGGGCTGCAGGTGCTGAGCGCGCTGGACGCGGCCAAGACGCAGTGGTACCACTTCACGGCCATCGTCGTCGCCGGCATGGGCTTCTTCACCGACGCCTACGACCTCTTCTGCATCTCCCTCGTCACCAAGCTCCTCGGCCGCATCTACTACACCGACCTCTCCAAGCCCGACCCCGGCACGCTGCCCCCCGGCGTGGCCGCCGCCGTCAACGGCGTCGCCTTCTGCGGCACGCTCGCCGGCCAGCTCTTCTTCGGCTGGCTCGGCGACAAGATGGGACGCAAGAGCGTCTACGGCATGACGCTGATTCTCATGGTCATCTGCTCCATCGGCTCGGGGCTCTCCTTCGCGCACACCCCCAAGAGCGTCATGGCCACGCTCTGCTTCTTCCGCTTCTGGCTCGGCTTCGGCATCGGCGGCGACTACCCGCTCTCCGCCACCATCATGTCCGAGTACGCCAACAAGAAGACCCGCGGCGCCTTCATCGCCGCTGTCTTCGCCATGCAGGGCTTCGGCATCCTCGCCGGCGGCATCGTCACCCTCATCATCTCCTCCGCCTTCCGCGCTGGGTTCCACGAGCCCGCCTACCAGGACGACCGCGTCGCCTCCACCGGCACCGAGGCCGACTTCGTGTGGCGCATCATCCTCATGCTCGGCGCCCTGCCGGCCCTGCTCACCTACTACTGGCGGATGAAGATGCCCGAGACGGCGCGCTACACTGCCCTCGTCGCCAAGAACGCCAAGCTCGCCGCCGCCGACATGTCCAAGGTGCTGCAGGTGGAGCTGGAGGACGAGACGGAGAAGATGGACGAGATGGTGAGCCGCGGGGCCAATGACTTCGGCCTCTTCTCGCCGCAGTTCGCGCGGCGCCACGGCCTCCACCTGGTCGGCACGGCCACCACGTGGTTCCTGCTGGACATCGCCTTCTACAGCCAGAACCTGTTCCAGAAGGACATCTTCACGAGCATCAACTGGATCCCCAAGGCGCGCACCATGAGCGCCCTCGACGAGGTGTTCCGCATCTCCCGCGCGCAGACGCTCATCGCCCTCTGCGGCACCGTGCCCGGCTACTGGTTCACCGTCTTCCTCATCGACGTCGTCGGCCGCTTCGCCATCCAGCTCATGGGCTTCTTCATGATGACCGTCTTCATGCTCGGCCTCGCCGTGCCCTACCACCACTGGACCACGCCCGGCAACCAGATCGGCTTCGTCGTCATGTACGCCTTCACCTTCTTCTTCGCCAACTTCGGGCCCAACGCCACCACCTTCGTCGTGCCCGCCGAGATCTTCCCCGCGAGGCTGCGGTCGACGTGCCATGGGATCTCCGCGGCCGCTGGGAAGGCCGGCGCCATGATCGGGGCGTTCGGGTTCCTCTACGCGGCGCAGGACCCGCACAAGCCGGACGCCGGGTACAGGCCCGGCATCGGCGTGCGCAACTCCCTCTTCGTGCTCGCGGGGGTCAACCTGCTGGGCTTCATGTTCACCTTCCTGGTGCCGGAGGCCAACGGGAAGTCGCTGGAGGAGATGTCCGGCGAGGCCCAGGACAACGAGGACCAGGCACGCGCCGCCGCCGTGCAGCCGTCCATGGCGTAGgccgccatatatttcacaactcgtgcgtgcgtgctagtgaagcagcttcaggcaggctgttgagttagtccttaatttgttgggtaatttgttttgtgatacgtacacgcttaaaatactactgtatcttcgcgggccaacccacctgcagggccggctcctcatgtacaatatgcccatgatatatacgtacatcaatcaatcaacttgcagttacaaatgggtattgcactcaaagtttagagctacagtgagcaaagctaacgcttgcttgagagttggtcagtgatgagcctcaagactctcttcagtcaacgaaagagaaatctcaaactataattgcactctgaggtggataattttaattttagttcctgtcactggttacttactagatttc

**TaPht1;3 chr5BL**

TRIAE_CS42_5BL_TGACv1_407907_AA1360190/ IWGSC WGA v0.4 chromosome 5B scaffold 11948

**partial RT-PCR fragment HG764736.1 (bold)**

gatcataatcgcaaccaangtaaaacatttcaatgcgaaaacaaatgcgatcataatcgcaaccaatgtaacaactgatccaatggcataatgataccaagcctcggtatgaatggcatattttctaatctttctaatcttcaagcgcattgcatccatcttgatcttgtgatcatcgacgacatccgcaacatgcaactccaatatcatcttctcctactcaatttttctaatttttttcttcaacaaattgttttcttcttcaactaaatttaacctctcgacaatagggtcggttggaatttccggttcaacaaactcctagataaataaaatctatgtcacattggtcggcataattgtcataaacaataaataaaccaatagttatgaaaagataatatataccacatctgaatcatagacaggacgaggaccgacgggggcggataccaaaaccatcgcactatataagatgcaataataaaagtaagaaaattatacaagtatctatataaatatacaaaattattttttcctttcagaaagaagataagaacaagaggctcaccacggtggtgccggcgacgagatcggcgcgggcgatcgacggcggtgaagacggggacgggacgtgacggaccgctaaatctagacaaatattgaggaaaatggagcttggaggtcgagcttggagaggagaaagcgtaagtagtatggctcgggcattccatcgaacacctcgtgtgcataggaggtgagctagagcaccacaaagttctcccctcgccggtcacgaaaaacaaagcactgggagtgctctgctcgcgggcgaggggtagatataggcaactaattggtcccggttcgtgccacgaaccgggattaaagggcagcctttggtctcggttcacgccaccaaccgggaccaatggtggtgggccaggagcgaggcgcattggtcccggttcgtcccaccaaccggaaccaataggtccagccgaaccgggaccaatgacccacgtggcccggccggcccccggggctcacgaaccgggtccaatgcccccattggtcccggttctggattgaaccgggactaatgggttgacccggcctggaccattgcccccttttctactagtgcaaggggcactgcctcaacattgcttgggtttgtacattgtgccagcaggggcggcgaggggttgagggtgtcggtttgatcgacgcgccccacagtaggcggtgtcatgtcggagtagcgaccatagaaagtgtggtcgatcgaccatgtagggtcttgtgtgagcacagtgaagagggctcctctacatagtgttattctctgcactcggatatgaataatgctacaacaacggaggacttaccggagttttacgggcttagctgatgtggtatattttgattggtaatttggaggaagcagggcccatcctgaaattcaggggagaaagatcagtccgttaactgtaaatatatcacatgtaagtcaaacatttttgctcagatattgacatgttgcattcggatcatattatttcccatgatatccctgtcaacgatcgagcatagaaactgcccaggatgcatagcatgcaatgaaacatcatgctcgcgggagcttagacaagagcgcatataataatatgagagataataaaagaaaaacgagaccaacacagcctagactagcctgttgaaatttttttatagaagaaaactctatgagcaccaagtgcttgagctgtgaactcccgcgccttgccaactaaaatacgctcagttctcataatatggaagatattatatcggtgattaccagctgcggccggtctcctcctcggctacttataccagcagcagccggcccataatttctgcaacacaacaaagtcttccggccggccggcaccgtcgtctagctctcagcctctcacactcgccccgttgccctgtgcagcagcagcaattgcatcATGGCGCGGTCGGAGCAGCAGGGGCTGCAGGTGCTGAGCGCGTTGGACGCGGCCAAGACGCAGTGGTACCACTTCACGGCCATCGTCGTCGCCGGCATGGGCTTCTTCACCGACGCCTACGACCTCTTCTGCATCTCCCTCGTCACCAAGCTCCTCGGCCGCATCTACTACACCGACCTCTCCAAGCCCGACCCCGGCACGCTGcCCCCCGGCGTCGCCGCCGCCGTCAACGGCGTCGCCTTCTGCGGCACGCTCGCCGGCCAGCTCTTCTTCGGCTGGCTCGGCGACAAGATGGGCCGCAAGAGCGTCTACGGCATGACGCTCATTCTCATGGTCATCTGCTCCATCGGCTCCGGCCTCTCCTTCGCGCACACACCCAAGAGTGTCATGGCCACGCTCTGCTTCTTCCGCTTCTGGCTCGGCTTCGGCATCGGCGGCGACTACCCGCTCTCCGCCACCATCATGTCCGAGTACGCCAACAAGAAGACCCGCGGCGCCTTCATCGCCGCCGTCTTCGCCATGCAGGGCTTCGGCATCCTCGCCGGCGGCATCGTCACCCTCATCATCTCCTCCGCCTTCCGCGCCGGGTTCCACGAGCCGGCCTACCAGGACGACCGCGTCGCCTCCACCGGCACCGAGGCCGACTTCGTGTGGCGCATCATCCTCATGCTCGGCGCCCTCCCGGCCCTGCTCACCTACTACTGGCGGATGAAGATGCCCGAGACGGCCCGCTACACCGCACTCGTCGCCAAGAACGCCAAGCTCGCCGCCGCCGACATGTCCAAGGTGCTGCAGGTGGAGCTGGAGGACGAGACGGAGAAGATGGACGAGCTCGTGAGCCGCGGCGGAGCCAACGACTTCGGGCTCTTCTCGCCGCAGTTCGCGCGGCGGCACGGCCTCCACCTGGTGGGCACGGCCACCACGTGGTTCCTGCTGGACATCGCCTTCTACAGCCAGAACCTGTTCCAGAAGGACATCTTCACGAGCATCAACTGGATCCCCAAGGCGCGCACCATGAGCGCCCTCGACGAGGTCTTCCGCATCTCCCGCGCGCAGACCCTCATCGCCCTCTGCGGCACCGTGCCCGGATACTGGTTCACCGTCTTCCTCATCGACGTCGTCGGCCGCTTCGCCAT**CCAGCTCATGGGCTTCTTCATGATGACCGTCTTCATGCTCGGCCTCGCCGTGCCCTACCACCACTGGACCACGCCCGGCAACCAGATCGGCTTCGTCGTCATGTACGCCTTCACCTTCTTCTTCGCCAACTTCGGCCCCAACGCCACCACCTTCGTCGTGCCCGCCGAGATCTTCCCCGCCAGGCTGCGCTCCACGTGCCACGGCATCTCCGCCGCCGCGGGCAAGGCCGGCGCCATGATCGGCGCGTTCGGGTTCCTCTACGCGGCGCAGGACCCGCACAAGCCCGACGCCGGGTACAGGCCGGGCATCGGCGTGCGCAACTCCCTCTTCGTGCTGGCCGGGGTCAACCTGCTGGGCTTCATGTTCACCTTCCTGGTGCCGGAGGCCAACGGGAAGTCGCTGGAGGAGATGTCCGGCGAGGCACAGGACAACGAGGAGGACCAGGCACGCGCCGCCGCCGTGCAGCCGTCCATGGCGTAGgccgccattcacaactcgtgcgtgctatg**ttagtcaactcaaagattctgatttgttttatgatactcaaagcaatatgtttgccggctacccaacctgcagggccagctcatgtatatgtccataatatatagatcaatcaatcaatttgcagctgcaaatgtgtgcaacggtcaaagcttagagagtgagctccacttgctcgacagttggtgagtggtgactgatgagcgtacccaaattaaggcctcattcgatttgaaggattttcgtagaaaaaacataggaaaaagaattttagaggaccattcggtttctaggaaatgcatatcggaatttcgtaggaatactactcatttccttaggatggcaagtgccatcctatcaaattcctgtatta

**TaPht1;3 chr5DL**

TRIAE_CS42_5DL_TGACv1_435451_AA1450800/ IWGSC WGA v0.4 chromosome 5D scaffold 1237

**partial RT-PCR fragment HG764735.1** (**bold)**

gaagtcatgttctttgtagtagagcgtgctgatcagtctcgtctcgactctagttaaaacataatcatggtaccgcggccgccgaatgccattttgtgtttatgcaaggagccaaaacaaaggttgtcaaatttgtctactgacgtccgtagactgtttacatctaggtgtgtcacctcgtcctcctgggacacgctatagctggagactttattagggtctaaagattccaggatatgccatcaaaccgttgatcccaagagttcccacgcagtttggtttagtttaggaaatctggatagcaaccgctcaaaaacaaaaaggaaatctggatagcaaccaaacggcaagaggacaggaaagcgcgttcccggccggactacaaaacgcatctcccgggccatcacccacatgacatcgttcacccattgcatgcatgatcgttggttcagcgaattaatgagtataaactaaaaccacggcaataattatgaaacaagaggagtgataaataaatattccaactcacggataccaattaatggctctttccacaccggtgggtacgttgcttcacgctagcttgacacaattgtttgacttgacgttcaaaaactaagcacagcggactgtaggttcgtgtgaaagacgcgattttcgtactagacaaagtgatcacaataattgttaaagccgctaggtaagacttgaggagtatataaaacatgtgattatcgttgaatttgcttcttcttttctttttggaactcaacaaatgtgcttgttattttatggaggagtcatgagtcaccaaagctgcaccattttattaagggttttgatatctaggttcagatgatatttatcatgatgtcaatggagagtgttgtgcgatgtgcggtgcttccaatgtgtctggatgtccccctttttctcgatggtgtgtggaggagctccaggcaaaagtccagttctgacctttggttggtgccaacaacagtggcgcaacgccatcggtcccctccttggaggcatttttgatggagctccattccacccccgtggtgttgtgggatcatcgggataaatccataggtcaaggtcactatagcgggtaatgacaatgtatgtgtcgcttcctccatggaggagttgggtggatatcttcaccgtcagaggcagcgggcgtcgaggtagcgaggctatgtggttactctattatggccaaggtcccgtctcgagaaacaatgcgcgtggggggagggggggaggcgtggtacttatgaattatgaggcaggggcactgcctcaacgttgcttgggtgagtaccttatgccaccaggacgacgagggggcgagggtgttagcttggctgacacgttccagagcaggcggtggcatgtcggaacagcgaccatagaaagtgtggtcgacggaccatgtagggcctcgcgtgagctcagtgaagagggctcctcgacatggtattctctgcactcggatatgagtaatgctacaactacggagggcttatcgtagttttacaggctcggctgatgtggtctatttctattggtaatttggcggaagcggggcccacactgaaattcaagggagagagattagtgagggaagaagtatcagtctgctaagtctatatataagtctaacaattttgctgggatattgatatgttgcattcagatcatataatttcccatgatatccttgtcaacgatcgagcatggaaattcctcgggatggtaacagctttacatgcagtgaaacttcatgctaccgggagcttagacaagaaagcatataatatgagagatattctcggtgattaccagctgcggccggtctcctcctcgcctacttataccagatagccggcccattatttctgcaccacaacacaagaaagtcttgcggccggccggcaccgtcgtctagctctcacactcgcaccgttgccctgtgcagcagcagcagcatcATGGCGCGGTCGGAGCAGCAGGGGCTGCAGGTGCTGAGCGCGCTGGACGCGGCCAAGACGCAGTGGTACCACTTCACCGCCATCGTCGTTGCCGGCATGGGCTTCTTCACCGACGCCTATGACCTCTTCTGCATCTCCCTCGTCACCAAGCTCCTCGGCCGCATCTACTACACCGACCTCTCCAAGCCCGACCCCGGCACGCTGcCCCCCGGCGTCGCCGCCGCCGTCAACGGCGTCGCTTTCTGCGGCACGCTCGCCGGCCAGCTCTTCTTCGGCTGGCTCGGCGACAAGATGGGCCGCAAGAGCGTCTACGGCATGACGCTCATTCTCATGGTCATCTGCTCCATCGGCTCGGGGCTCTCCTTCGCGCACACCCCCAAGAGCGTCATGGCCACGCTCTGCTTCTTCCGCTTCTGGCTCGGCTTCGGCATCGGCGGCGACTACCCGCTCTCCGCCACCATCATGTCCGAGTACGCCAACAAGAAGACCCGCGGCGCCTTCATCGCCGCTGTCTTCGCCATGCAGGGCTTCGGCATCCTCGCCGGCGGCATCGTCACCCTCATCATCTCATCCGCCTTCCGCGCTGGGTTCCACGAGCCGGCCTACCAGGACGACCGCGTCGCCTCCACCGGCACCGAGGCCGACTTCGTGTGGCGCATCATCCTCATGCTCGGGGCCGTTCCGGCGCTGCTCACCTACTACTGGCGGATGAAGATGCCCGAGACGGCGCGCTACACCGCCCTCGTCGCCAAGAACGCCAAGCTCGCCGCCGCCGACATGTCCAAGGTGCTGCAGGTGGAGCTCGAGGACGAGACGGAGAAGATGGACGAGATGGTGAGCCGCGGGGCCAACGACTTCGGCCTCTTCTCGCCGCAGTTCGCGCGGCGGCACGGCCTCCACCTGGTGGGCACGGCCACCACGTGGTTCCTGCTGGACATCGCCTTCTACAGCCAGAACCTGTTCCAGAAGGACATCTTCACGAGCATCAACTGGATCCCCAAGGCGCGCACCATGAGCGCCCTCGACGAGGTGTTCCGCATCTCCCGCGCGCAGACGCTCATCGCCCTCTGCGGCACCGTGCCCGGATACTGGTTCACCGTCTTCCTCATCGACGTCGTCGGCCGCTTCGCCAT**CCAGCTCATGGGCTTCTTCATGATGACCGTCTTCATGCTCGGCCTCGCCGTGCCCTACCACCACTGGACCACGCCCGGCAACCAGATCGGCTTCGTCGTCATGTACGCCTTCACCTTCTTCTTCGCCAACTTCGGCCCCAACGCCACCACCTTCGTTGTGCCCGCCGAGATCTTCCCGGCGAGGCTGCGCTCCACGTGCCACGGGATCTCGGCCGCCGCGGGGAAGGCCGGCGCCATGATCGGCGCGTTCGGGTTCCTCTACGCGGCGCAGGACCCGCACAAGCCCGACGCCGGGTACAGGCCAGGCATCGGCGTCCGCAACTCCCTCTTCGTGCTCGCCGGGGTCAACCTGCTGGGGTTCATGTTCACCTTCCTGGTGCCGGAGGCCAACGGGAAGTCGCTGGAGGAGATGTCCGGCGAGGCCCAGGACGACGAGGACCAGGCACGCGCCGCCGCCGCCGTGCAGCCGTCCACGGCCTAGtaggccgccatatattccttcacaactcgtgcgtgctagtg**aggcagctgcaggctgttgagctagtcaaagatccgtaatttgttgggtaatttgttttgtgatacgtacacgcataaaatactattacaactataggtttgcgggccacccaacctgcagggccggctcatgtatatgcccatgatatatacgtacatcaatcaatcaacttgcagttgcaaatgggtattgcactcaaagtttagagctagagtgagcaaagctaacgcttgcttgagagttggtcagtgatgagcctaaggacctcaagactctcttcagtcaacgaaagagaaatctcaaactataattgcactctgaggtggataattttaattttagttcctgtcactggtt

**TaPht1;4 Chr4AL**

TRIAE_CS42_4AL_TGACv1_289415_AA0970660/ IWGSC WGA v0.4 chromosome 4A scaffold 104865

ccataaaactcgacgtaagtcttcgtgggtgtagcattgctcgttatctcctataagtctaataactttgtaaatattaccttgtaagtgttaacattttggctcggatgttgataggttgcattcagatgatattatttcccatgccgcatgatatccttgtcaacgatggagcatggaaattccccaggatggtaacagctttacatgcagtgaaacttcatgctcgcgggagcatagacaagagaacataatactctatcaatattcgcggtgacacccagctctcctcggctacttataccagcgatagcccgcccattatttctgcaccacgacacaagaaagtcttgcggccggccggcaccgtcgtctagctctcacactcgcaccgttgccctgtgcagcagaagcagcagcagcggcATGGCGCGGTCGGAGCAGCAGGGGCTGCAGGTGCTGAGCGCGCTGGACGCGGCCAAGACGCAGTGGTACCACTTCACGGCCATCGTCGTCGCCGGCATGGGCTTCTTCACCGACGCCTACGACCTCTTCTGCATCTCCCTCGTCACCAAGCTCCTCGGCCGCATCTACTACACCGACCTCTCCAAGCCCGACCCCGGCACGCTGCCCCCCGGCGTGGCCGCCGCCGTCAACGGCGTCGCCTTCTGCGGCACGCTCGCCGGCCAGCTCTTCTTCGGCTGGCTCGGCGACAAGATGGGACGCAAGAGCGTCTACGGCATGACGCTGATTCTCATGGTCATCTGCTCCATCGGCTCGGGGCTCTCCTTCGCGCACACCCCCAAGAGCGTCATGGCCACGCTCTGCTTCTTCCGCTTCTGGCTCGGCTTCGGCATCGGCGGCGACTACCCGCTCTCCGCCACCATCATGTCCGAGTACGCCAACAAGAAGACCCGCGGCGCCTTCATCGCCGCTGTCTTCGCCATGCAGGGCTTCGGCATCCTCGCCGGCGGCATCGTCACCCTCATCATCTCCTCCGCCTTCCGCGCTGGGTTCCACGAGCCCGCCTACCAGGACGACCGCGTCGCCTACACCGGCACCGAGGCCGACTTCGTGTGGCGCATCATCCTCATGCTCGGCGCCCTGCCGGCCCTGCTCACCTATTACTGGCGGATGAAGATGCCCGAGACGGCGCGCTACACTGCCCTCGTCGCCAAGAACGCCAAGCTCGCCGCCGCCGACATGTCCAAGGTGCTGCAGGTGGAGCTGGAGGACGAGACGGAGAAGATGGACGAGATGGTGAGCCGCGGGGCCAATGACTTCGGCCTCTTCTCGCCGCAGTTCGCGCGGCGCCACGGCCTCCACCTGGTCGGCACGGCCACCACGTGGTTCCTGCTGGACATCGCCTTCTACAGCCAGAACCTGTTCCAGAAGGACATCTTCACGAGCATCAACTGGATCCCCAAGGCGCGCACCATGAGCGCCCTCGACGAGGTGTTCCGCATCTCCCGCGCGCAGACGCTCATCGCCCTCTGCGGCACCGTGCCCGGCTACTGGTTCACCGTCTTCCTCATCGACGTCGTCGGCCGCTTCGCCATCCAGCTCATGGGCTTCTTCATGATGACCGTCTTCATGCTCGGCCTCGCCGTGCCCTACCACCACTGGACCACGCCCGGCAACCAGATCGGCTTCGTCGTCATGTACGCCTTCACCTTCTTCTTCGCCAACTTCGGGCCCAACGCCACCACCTTCGTCGTGCCCGCCGAGATCTTCCCCGCGAGGCTGCGGTCGACGTGCCATGGGATCTCCGCGGCCGCTGGGAAGGCCGGCGCCATGATCGGGGCGTTCGGATTCCTGTACGCGGCGCAGGACCCGCACAAGCCGGACGCCGGGTACAGGCCAGGCATCGGCGTGCGCAACTCCCTCTTCGTGCTCGCCGGGGTCAACCTGCTGGGGTTCATGTTCACCTTCCTCGTGCCGGAGGCCAACGGGAAGTCGCTCGAGGAGATGTCCGGCGAGGCACAGGACAACGAGGACCAGGCACGCGCCGCCGCCGTGCAGCCGTCCACGGCGTAGgccgccatatattcacaactcgtgcgtgcgtgctagtgaggcagctgcatgcaggctgtctacgttagtctgctcaaagattctgatttgttttatgatacgtacacgatagatagatccaggtgcaagtactcaaagcacaatatgtttgccgggccagctcatgtatatgtccataatatatagataaaaaacaatgtgtcaaagcttagagagtgagctaccacttgctcgacagttggtgaatgatgagtcgtaagctgatgcatctcttcgtagccaaattaaagaacctcaggtcaacgaaagagaaagctcaaactgtaattgcactctgagatggacaactttaattagagatcccacccatggatcttctttttgcctttgttcttctccc

**TaPht1;4 chr5BL**

TRIAE_CS42_5BL_TGACv1_407907_AA1360180/ IWGSC WGA v0.4 chromosome 5B scaffold 11948

**Partial RT-PCR fragment HG764737.1 (bold)**

5’ and 3’ end confirmed by AK333026

gtatcacatgttggtatcatagaggacttcatttattgtcatgcataacacaaagtagcatatcatttaacatgattcggtatcaatgatatgataatcaagcatctctttcttcatttagtcctatgccaactcatcaaaattgcttacttggcatgcatgatactacctatgatactcccatcacgggcaggcttatcggagttttacagcctcggctgatgtgggctattttggttggtaatttggaggaagcggggcccatcctaaagcggtgattattgcggcgagaaagatgagtccgttaactctgtaaatatcaccttgcaagtcttaatatttggaaaatcctattggacgctcgctgcgtcgacttgtcgaggcttcgcacaaagagctagcgatagcgatcaagatgggccggcccagttaggagatagcgctggcgctgcagcttccggttttgggaaccttctggagggttcctgaacggttttgggaaccttctagaaggttcttgaaccgggttttcactggttttttctgtttgctttttctttgttgtttttcctgttttttttctttttctttctttgtttttctgtttctttatttcatttttcaattcctttttcttctctttttgttttctttttgttcacatttcaaattaggtttgggagtttcaaaaaatcttcccatttcaattcgcgcttccaaatttgttctccatttcaaaatttgttttcaacattcaaaaattgttcatgctttaaaaaaatgtccgcgcttctaaatttgttctccatttcaaaatttgttctcaacattcaaaaattattcgcgcttaaaaattgttctccatttcaaaatttgttctcaacattcaaaaattgttcgtgctttcaaaaattgttcgtgcttccaaatttgttctccatttcaaaatttgttctcaagattcaaaaattgttcatgctttcaaaaattgttcgagcttccaaatttgttctctatttgaaaatttgttctcaacatttaaaaattgttcgtgctttaaaaaattgttcgtgcttccaaatttgttctttgtttcaaaatttgttcttaagatttagaaaatgtttgtgctttaaagaaatgtcacaaattccaagaatatttgggaaattcaaaagttgtttgtgtttccaaattttgttttgaaatttgaaaaattgttccagttcagttttttgggtagtttaaaaaatgtttccgttccaaaaaaatatgttcgcgtctccaaaattcatttgtgtgttgaaaaagtgtttgagttttctcaaaatttgtaaaataaaactaatagtgttcgcgtttgataaaatattcaccttttgtttttgctatttgggaaatcgtaaagtatgtttttcaaacagtcttatactttctttagttcttgtaggtacccgctgcagtttggtcaaatgagctcgtccatcctagtggctagtagggtgcgcgtgcgagtcgccagtgctgagtttgcaccctactattttttgcaatttatcacctggtatttgcagcgcactgcaatgggccggcccaatcagcgcaccgacgacgtgcctgtgcgaaagtgcagcattttgacgcaaaatgcgtcacataggaactcccttaatattttgtctctgatgttgataggttgcattcagatcatattatttcccatgccggatgatatccttgtcaacgatggagcatggaaattccccagatgtaacagctttacatgcggtgaaacttcattctcgcgggagcctagacaagagaacatataataataatatcaatattcgcggtgacacccagctctcctcggctacttataccagacagccggcccattatttctgcaccacaacacaaggaagatttccggccggccggcaccgtcgtctagctctcacactcgccccgttgccctgtgcagcagcagcagcagcatcATGGCGCGGTCGGAGCAGCAGGGGCTGCAGGTGCTGAGCGCGCTGGACGCGGCCAAGACGCAGTGGTACCACTTCACGGCCATCGTCGTCGCCGGCATGGGCTTCTTCACCGACGCCTACGACCTCTTCTGCATCTCCCTCGTCACCAAGCTCCTCGGCCGCATCTACTACACCGACCTCTCCAAGCCCGACCCCGGCACGCTGCCCCCCGGCGTCGCCGCCGCCGTCAACGGCGTCGCCTTCTGCGGCACGCTCGCCGGCCAGCTCTTCTTCGGCTGGCTCGGCGACAAGATGGGCCGCAAGAGCGTCTACGGCATGACGCTCATTCTCATGGTCATCTGCTCCATCGGCTCCGGCCTCTCCTTCGCGCACACACCCAAGAGTGTCATGGCCACGCTCTGCTTCTTCCGCTTCTGGCTCGGCTTCGGCATCGGCGGCGACTACCCGCTCTCCGCCACCATCATGTCCGAGTACGCCAACAAGAAGACCCGCGGCGCCTTCATCGCCGCCGTCTTCGCCATGCAGGGCTTCGGCATCCTCGCCGGCGGCATCGTCACCCTCATCATCTCCTCCGCCTTCCGCGCCGGGTTCCACGAGCCGGCCTACCAGGACGACCGCGTCGCCTCCACCGGCACCGAGGCCGACTTCGTGTGGCGCATCATCCTCATGCTCGGCGCCCTCCCGGCCCTGCTCACCTACTACTGGCGGATGAAGATGCCCGAGACGGCCCGCTACACCGCACTCGTCGCCAAGAACGCCAAGCTCGCCGCCGCCGACATGTCCAAGGTGCTGCAGGTGGAGCTGGAGGACGAGACGGAGAAGATGGACGAGCTCGTGAGCCGCGGCGGAGCCAACGACTTCGGGCTCTTCTCGCCGCAGTTCGCGCGGCGGCACGGCCTCCACCTGGTGGGCACGGCCACCACGTGGTTCCTGCTGGACATCGCCTTCTACAGCCAGAACCTGTTCCAGAAGGACATCTTCACGAGCATCAACTGGATCCCCAAGGCGCGCACCATGAGCGCCCTCGACGAGGTCTTCCGCATCTCCCGCGCGCAGACCCTCATCGCCCTCTGCGGCACCGTGCCCGGATACTGGTTCACCGTCTTCCTCATCGACGTCGTCGGCC**GCTTCGCCATCCAGCTCATGGGCTTCTTCATGATGACCGTCTTCATGCTCGGCCTCGCCGTGCCCTACCACCACTGGACCACGCCCGGCAACCAGATCGGCTTCGTCGTCATGTACGCCTTCACCTTCTTCTTCGCCAACTTCGGCCCCAACGCCACCACCTTCGTCGTGCCCGCCGAGATCTTCCCCGCCAGGCTGCGCTCCACGTGCCACGGCATCTCCGCCGCCGCGGGCAAGGCCGGCGCCATGATCGGCGCGTTCGGGTTCCTCTACGCGGCGCAGGACCCGCACAAGCCCGACGCCGGGTACAGGCCGGGCATCGGCGTGCGCAACTCCCTCTTCGTGCTGGCCGGGGTCAACCTGCTGGGCTTCATGTTCACCTTCCTCGTGCCGGAGGCCAACGGGAAGTCGCTCGAGGAGATGTCCGGCGAGGCCCAGGACAACGAAGACCAGGCACGCGCCGCCGCCGTGCAGCCGTCCACGGCGTAGctaggccggccgccatatattcacaactcgtgcgtgc**tagtgaagctgcaggatgttgagtgggtcaaagatccttaatctgttgggtaattttttttgtgatacgtacacgcttaaaatactactgtatgtttgccggctcatgtatatgtccacaatatattatctacatcaatcaatcaacttgcagttgcaaatgtgctagagagttggtcaatgatatgatgagcctaagctgtggcatctcttcgtatccgatccaagaacctcaagactctcttcagtcaatgaaagagaaatttcaaactataattgcactctgaggtggataatttaattttagttcctgtcactggttacttattggatttgatttgattaattctgtgagtagttctag

**TaPht1;4 Chr5DL**

TRIAE_CS42_5DL_TGACv1_433845_AA1423630/ IWGSC WGA v0.4 chromosome 5D scaffold 1237

atgaaataaatggagtaataaataattgtttcaactcatggatatcaattaatggctctctccacaccggtgggtacgttgcttcgaattaagtacgcttgatgcagttgtttcttccgcaaaaaaatgcagttgttggacttaacgatcaaaaactaagcacatcggactgtgtgttcgtgtgaaagacgcaatttgatagataaattgatcacaataattgttaaagcagctagctaaggctagtcatagtgggagtaacttagatagtaacatagcgcattccaagaaatttaataaggagagaggtgtttagagtaacataatatgttactgtaacataacgcttcccgagaaaggatgagtctacaagctaataaatgaagccatctatgatattactactgtgttactttgcattatgaaggtagtaacttagactagtgtcatatgcatgacactagtatgacactagtataagttagtacccactatgaccagcttaagactttaggagtatgaaatatgccattatccttaaatttgcttcttcttttccttttggaactcaacaaatttgcttgttagttgtggcggagtcatgagttatcgaagctaaaccatttaataatggttctgatatctaggttcacatgatatctatcatgacgccgatggagagtgtcatgcgatgtgcggtgcatccaatgtatcttgatgtcacccctttctcgatggtgtgtggaggagctcctcgcgaaagtccaattccaaccttggttgctgccaacaatggcggcatgatgccgccggacctttccttggaggcttttttgtggagcttcgttccacccccgtggggttgtgggctcatcgggataaatcagcagctcaaggtcgctatagcgcgcaatgacagtggttgtgtcacttcctccttggaggtggtggatagtaagcgcatagcagctgtatggatctcttcgccttcagaggcagtggacgtcgaggcagcgaaaccatatggttactctttgtgtggccgaggtcctggtctcgagaaacaatgcgctcggtggaagggggttgtacttatgagttatgatgcgaggggcactgcctcaacattgcttcggtgtgtacattgtgccagcaggggcggcgaggggttgagggtgtcggtttgatcgacgcgccccagagtaggtggtgtcatgtcggagtagcgaccgtagaaagtgtggtcaatagaccatgcagggtcttgtgtgagcaccgtgaagagggctcctcgacatagtggtattctctgcactcggatatgaataatgctacaacaacggaggacttaccggagttttgcgggctcagctgatgtggtatattttgattggtaatttggaggaagcagggcccatcctgaaattcaggggagatcagtgagggggagaaagatcagtccgttaactataaatatcacacgtaagtcaaacatttttgctcagatattgacatgttgcattcagatcatattatttctaatgatatccttgttaacgaccgagcatggaaactccccaggatgctaacagcatgcaatgaaacatcatgctcgcgagagcttagacaagagcgcatataatagtacgggagatactaaaataaaaacgagaccaactgagctagactagcgtgttggcatttttttatagaaaaaaactccgtgaacaccaagtgcttgagccagctggccgcgaacttccgcgcgccttgccaactaagctatgctcacttctcataatataagagatattatatcggtgatgtgattaccagctgcggccggtctcctcctcgcctacttataccagcagcagccggcccataatttctgcaacacaacaaagtcttccggccggccggcgacgtcgtctagctctgacactcacagtcacacattgccgcggccaaacgtccctcccctgagcagcagcagcagcATGGCGCGGTCGGAGCAGCAAGGGCTGCAGGTGCTCAGCGCGCTGGACGCGGCCAAGACGCAGTGGTACCACTTCACGGCCATCGTCGTCGCCGGCATGGGCTTCTTCACCGACGCCTACGACCTCTTCTGCATCTCCCTCGTCACCAAGCTCCTCGGCCGCATCTACTACACCGACCTCTCCAAGCCCGACCCCGGCACGCTGCCCCCCGGCGTCGCCGCCGCCGTCAACGGCGTCGCCTTCTGCGGCACGCTCGCCGGCCAGCTCTTCTTCGGCTGGCTCGGCGATAAGATGGGCCGCAAGAGCGTCTACGGCATGACGCTGATTCTCATGGTCATCTGCTCCATCGGCTCCGGCCTCTCCTTCGCGCACACCCCCAAGAGCGTCATGGCCACGCTCTGCTTCTTCCGCTTCTGGCTCGGCTTCGGCATCGGCGGCGACTACCCGCTCTCCGCCACCATCATGTCCGAGTACGCCAACAAGAAGACCCGCGGCGCCTTCATCGCCGCCGTCTTCGCCATGCAGGGCTTCGGCATCCTCGCCGGTGGCATCGTCACCCTCATCATCTCCTCCGCCTTCCGCGCTGGGTTCCACGAGCCGGCCTACCAGGACGACCGCGTCGCCTCCACCGGCACCGAGGCCGACTTCGTGTGGCGCATCATCCTCATGCTCGGGGCCGTTCCGGCGCTGCTCACCTACTACTGGCGGATGAAGATGCCCGAGACGGCGCGCTACACCGCCCTCGTCGCCAAGAACGCCAAGCTCGCCGCCGCCGACATGTCCAAGGTGCTGCAGGTGGAGCTCGAGGACGAGACGGAGAAGATGGACGAGATGGTGAGCCGCGGGGCCAACGACTTCGGCCTCTTCTCGCCGCAGTTCGCGCGGCGGCACGGCCTCCACCTGGTGGGCACGGCCACCACGTGGTTCCTGCTGGACATCGCCTTCTACAGCCAGAACCTGTTCCAGAAGGACATCTTCACGAGCATCAACTGGATCCCCAAGGCGCGCACCATGAGCGCCCTCGACGAGGTCTTCCGCATCTCCCGCGCGCAGACGCTCATCGCCCTCTGCGGCACCGTGCCCGGATACTGGTTCACCGTCTTCCTCATCGACGTCGTCGGCCGCTTCGCCATCCAGCTCATGGGCTTCTTCATGATGACCGTCTTCATGCTCGGCCTCGCCGTGCCCTACCACCACTGGACCACGCCCGGCAACCAGATCGGCTTCGTCGTCATGTACGCCTTCACCTTCTTCTTCGCCAACTTCGGCCCCAACGCCACCACCTTCGTCGTGCCCGCCGAGATCTTCCCGGCGAGGCTGCGCTCCACGTGCCACGGGATCTCGGCCGCCGCAGGGAAGGCCGGCGCCATGATCGGCGCGTTCGGGTTCCTCTACGCGGCGCAGGACCCGCACAAGCCCGACGCCGGGTACAGGCCAGGCATCGGCGTCCGCAACTCCCTCTTCGTGCTCGCCGGGGTCAACCTGCTGGGGTTCATGTTCACCTTCCTGGTGCCGGAGGCCAACGGGAAGTCGCTGGAGGAGATGTCCGGCGAGGCCCAGGACAACGAGGACCAGGCACGCGCCGCCGCCGTGCAGCCGTCCACGGCCTAGtaggccgccatatattccttcacaactcgtgcgtgctagtgaggcagctgcatgcaggctgtctacgttagtcaactcaaagattctgatttgttttatgatacgtacgcgatagatccacgtgcaagtactcaaagcaatatgtttgccggctacccaacctgcagggctggctcatgtatatgtccataatatatagataaatcaatcaacttgcagctgccaatgtgtgcaacggtcaaagcttagagagtgagctaccacttgctccacactcggtgaatgatgagcctaaggcttgtcatagtgagagtaacttatactaatgtcatgcatataacattaatttaaattactatctttataatataaagtaacataacagtagtgttatggatgg

**TaPht1;5 Chr4AL**

TRIAE_CS42_4AL_TGACv1_290489_AA0985980/ IWGSC WGA v0.4 chromosome 4A scaffold 95763

gggaacattttgttttaaaaatacactaaaagtactttcatatgcgtggatatttttccttgcgacaacaccatgttttctttttcatgcacaaaacattactagcaattttgtttcatagagcccctagatgttcccatcctaaaaaacttgagaagttgtgaacagtataagtgcattgtcaagtagagacaaggaagaaggaaaatgcagtatccttatgttatgaaaagttcatctgacaatgggtgaagtaatcgatcatcgaagagttatttggggaagattgctctaaggacatggaagaagtcttgaccttcattgagaaggaggatgttttgcctcccattgaagaaaataaaatctgtaaagcaaacaaacccattcttggggaaggtgagatgaactaactattccttccgtcatggaacatagagccagagtaaataaaattgctaatattgatcttactttcctagttatgtatgtcatgtacgcgatacaaagttcctgatagagaccttctttgagttgaaccatagggttttctgagaaaaactaaggtacattatcccaaaaatgttctagaatattgtttatagactagataggtgtccgcgacataaaatagagatcagacttttgtgtctccctagtggctaggaagcattagctttaccagctggaatgcaagatcttcgccatagtgccaaggaaagactctagctccacaaaaaaccaaaccaacaaaagcaccaagacactccaaaaaaaaactcagcgtattcaacagatctctcttgcaacaacataaaccttaactccgcaacaacagttgccccaaactgtcataggaaacgtacctcttgctatagtagagaaaataccagacaacatttatgttcaacgacaccacgtccaccctcaatacgttgtctttaggaccaaaaatcagcttgaaatgagtattaaaacacatgtatgaacactctaggattccccgcctcacatcgccaaaatgacggctaggagtgaggagacgagtagaaattccgacgacggttgtattcttttttttagagagagcacccgaacttttattaagtaatgattgagtttacatagcccgagggttcgacaaagccaaacgtggcgacctaatgaatttcttgttgtacaatgggcatcttcattaacttacattcccttatgtttgaaaatcacttcctcgcaaccactcattttagagacagcctcccaaatgatcatggcatgtgctccaaggtttccttcaattgatatttcggatgaacggctgagcagtctgaagcgatcaccatggaatttagatctatatcgcgagcaaggcaaagggcctccccgcatgctattgtttccacacatgacaaattagttatgccctccatcaccggggttgatgcgcctaagaaattgtcttcagcagattgtaatataatcttcaccgcacttctgaaattcgcagccgataccggtgcatcaacatcaccccttcagctgtcctcggggaggggggatccgctttcttagaggtctcaagattctctctggccgaacatgctctggtaaacatcttcacggacaacatccgatatgtagtgtcacaactactttcatatactcctaccaaccctaaataaaatgagtcttcatatcctaggaaaacattagctacctttttaatgggctgccagatgatgaggcccatgtgagcgatgcctacctattgctcgcggtaggcagcacatacgttgtctcaaaaaaaaagaagtatccgtgcaagcaacagactctggctacctagctgccagtcgtccaactccatgccgaatcggttttgtacgccacgcacccaaacctataaacgtgccgctcgatcgccagggcgcagcagcgacagatcgaagcagcaacacgaactttggcacgagacctgcgacacggcgacaccccgcggcgcgATGGCGAGCCGGCAGCTGCAGGTGCTGAGCGCGCTGGACGGCGCCAAGACGCAGTGGTACCACTTCAGGGCGATCGTCGTCGCCGGCATGGGCTTCTTCACCGACGCCTACGACCTCTTCTGCATCTCGCTCGTCACCAAGCTCCTCGGCCGCATCTACTACGCCGACCCCTCCAGCCCCACCCCTGGCTCGCTGCCGCCCAACGTGGCCGCGGCCGTCAACGGCGTCGCGCTCTGCGGCACCCTCGTCGGCCAGCTCTTCTTCGGCTGGCTCGGCGACAGGCTCGGCCGGAAGAGCGTCTACGGCATGACGCTCATGCTCATGGTCGTCTGCTCCGTCGCCTCCGGGCTCTCGTTCGGACACTCCCCGGCCAGCGTCATGGCCACGCTCTGCTTCTTCCGCTTCTGGCTCGGCTTCGGCATCGGCGGCGACTACCCTCTGTCCGCCACCATCATGTCCGAGTACGCCAACAAGAAGACAAGAGGGGCGTTCATCGCCGCCGTCTTCGCCATGCAGGGCTTCGGCATCCTCGCCGGCGGCGTCGTCACGCTCGTCCTCTCCACGGTTTTCCGTAACGCTTTCCCGGCCCCGGCGTACCAGGTCGACGCCGCCGCGTCCACCGTGCCGCAGGCGGACTACGTGTGGCGCATCATCCTCATGCTCGGCGCGCTGCCTGCGGCGCTCACGTACTACTGGCGAACGAAGATGCCGGAGACGGCGCGGTACACGGCGCTGGTCGCAAAGGACGCGAAGCAGGCCTCGCTGGACATGTCCAAGGTGCTTCAATCGGAGATCGAGGCGGAGCCGGAGAAGCTGGACGAGATCATGGCCAGAGGCGAGGACTACGGCCTCTTCACGTCGCGGTTCGCCAAGCGCCACGGCCTGCACCTCCTCGGCACGGCGACGGCGTGGTTCCTGGTCGACGTCGCGTACTACAGCCAGAACCTGTTCCAGAAGGACATCTTCGGCAGCATCGGGTGGATCCCCAAGGCGCGCACCATGGACGCGCTCGAGGAGGTGTTCCGCATCTCCCGCGCGCAGACGCTCATCGCGCTCTGCGGCACCGTGCCGGGCTACTGGTTCACCGTGTTCCTCATCGACGTCGTCGGAAGGTTCTGGATCCAGCTCGTGGGGTTCGCCATGATGACCGTTTTCATGCTCGGCCTCGCGCTGCCGTACCACCACTGGACGACGCCGGGCAACCACGTGGGCTTCGTCGTCATGTACGGGCTCACCTTCTTCTTCGCCAACTTCGGGCCGAACGCAACCACGTTCATCGTGCCGGCCGAGATCTTCCCGGCGCGGCTCCGGTCGACCTGCCACGGCATCTCCGCTGCGGCGGGGAAGGCCGGTGCCATCATCGGGGCGTTCGGGTTCCTCTACGCGGCGCAGTCGCAGGACCCGGCGCACGTGGACGCCGGGTACAAGCCTGGGATCGGCGTGCAGAAGGCGTTGTATGTGCTCGCTGCGTGCAACCTCCTGGGGTTCTTGGTGACGTTCCTCGTGCCGGAGTCGAAGGGGAAGTCGCTCGAGGAGATGTCCGGCGAGGCCGACGCCGAGGAAGGCAACGGCGCCAATATCGTCCGCCCGTCGGGAGAGCAGCTGGTTTGAatcgttttgatcctcgagaaggcaacacacgcatacgtgcatgttctgtaagtgaagtagcaagcatgcagctagccagctagccgctaggcctttagccccacactattatacttagcaacgtgtataacttggcctttattcccgcaaggaaaatagcacttgtgttttagtacaaaattcatgataacatctctaatggaagaatggcttatgggagtactttaaatacttatattattgtcaaactatttttctaatatgtgacaattagaagacaagaacacaaattacacaaaatgtgcactcttccatactgtgcataaacaagcataaagtagtgcaactttagtattatgaaaatgaatattcccctcaaaaaataaaaatgaatttcatt

**TaPht1;5 Chr4BL**

TRIAE_CS42_4BL_TGACv1_320302_AA1034390/ IWGSC WGA v0.4 chromosome 4B scaffold 128405

**partial RT-PCR fragment HG764738.1 (bold)**

ccgagagttcgacaaagctaaacatggcgaccagctgaaagttctaacgaatttcttgtcatagaatgggcatcttcattaacttccatccctttatgcttaaatatcacttcttggcagctactcattttagagacaatctcccaaatcatcatggcatgtgctccaaggcttcattgatattttggatgagcggctgagcagtttgaagcgatcaccatggaatttagatctatatcccgagcaaggaaaagggcctccatgcatgctttccacacataacgaattagttatggcctccattaccatgactgatgcgcctaaggaattgtcttcatcagatcataatataattgtcacgaaacttctcaaattcgcaactgataccggtgcatcaaattacccttcaatcgtcctcagggaggggagggggatccaccttcgcggaggtctcaagcttctctctgggcgaaaatgcattattcgaacgtttatatcaagcttttgaatgaagtctgatatgaagttgcatgttgtgaactttttttttgcgggtaaaataggagtttcttcaactgtaataaggctagaatatgctaatacaggatcgtgaataaaggggggcctcttggcaacaacaggcagtgctccgagccgtacaagtatagttagctaagcaatatgtaaccctattttgcatatgagcaaccataactagaatatactcccggtctgccataagcgtcttaatgttctggctgatgttaccatatagagatatggagagcaaagcatccttcatggtgtataaggctcccattgagtttgagtgaaccaattcgggaaggttagaccaccgtagggctagtgacatacccctctaatactgccttgatctttgcttcaagtgcacattgcaataaaaattgtagcttacatgatgcaaagataatatggcatgtgctaatctccagtatcatatcaacacatgttgaaccatcctccagagagaaggacccatccacagaaagagccagtcaatcctgctggtggcgctggccatgggaaggttgtgaacatgctttggtaaacatcttcacgaacaacatcccatatataatgtcacgattactttcacatactcctacgaaccctaaataaactgagtcctaggttgaagttcatgtcataggaattacttcaaagatgaacacgtcttggtattttttttttcttttcttataagaaacagtaatagctagtatctttttaatgggctgcccatgtgagcgctgtctacttattgctcgcggtaggcagcacatactcctactaagggcctgttcggcaactgcccaactcctggtttctctgaatcagcgggcgaagctggaccgaacaggtcagctcccagaagctagcttcccagatctaagacgggtcgtagtataaaaactgggagcgagtgtttcatggatcccagaaatcgcagaagctggagttcaagttatttacggcgagctgccaccgctaaatgaaagaaaacgcttcgtacagggaagaatagaacgcagcaactaccttcgctccctaacaggccagaatcgcttaatgggccagccggctaccagcccaagactagcgaactgggatctgtgaattctgttgccgaacggtttttctgatccactattttcgtcgggaagctggaggtgagatcgagaagccggatttgaggatttctggaagctgggcagttgccgaacaggccctaattatcaaatcgtgtccgccccggttttccctagctgtatccgtgtaagcaacagactctgcttacctttgctgccagtcgtccaactccatgccgaatcggttttcgtctcccacgcatccaaatctataaacgtgctgctcgatagccagggcgcagcagggcagcgacagatcgaagtagcaagacgaactttggcacgagatctgcgaaaccccgaggcgcgATGGCGAGCCGGCAGCTGCAGGTGCTGAGCGCGCTGGACGGCGCCAAGACGCAGTTGTACCACTTCAGGGCGATCGTCGTCGCCGGCATGGGTTTCTTCACTGACGCCTACGACCTCTTCTGCATCTCGCTCGTCACCAAGCTCCTCGGCCGCATCTACTACGCCGACCCCTCCAGCCCTAACCCTGGCTCGCTGCCGCCCAACGTGGCCGCGGCCGTCAACGGCGTCGCGCTCTGCGGGACCCTCGCCGGCCAGCTCTTCTTCGGCTGGCTCGGCGACAAGCTCGGCCGGAAGAGCGTCTACGGCATGACGCTAATGCTCATGGTCGTCTGCTCCGTCGCCTCCGGGCTCTCGTTCGGACACACCCCGGCCAGCGTCATGGCCACGCTCTGCTTCTTCCGCTTTTGGCTCGGCTTTGGCATCGGCGGCGACTACCCTCTGTCCGCCACCATCATGTCCGAGTACGCCAACAAGAAGACGCGCGGAGCCTTCATCGCCGCTGTCTTTGCAATGCAGGGCTTCGGCATCCTCGCCGGCGGCGTCGTCACGCTCGTCCTCTCCACGGTTTTCCGTAACGCGTTCCCGGCGCCAGCGTACCAGGTCGACGCCGCCGCGTCCACCGTGCCGCAGGCCGACTACGTGTGGCGCATCATCCTCATGCTCGGCGCGCTGCCAGCGGCGCTCACGTACTACTGGAGAACGAAGATGCCGGAGACGGCGCGGTACACGGCGCTGGTCGCAAAGAACGCGAAGCAGGCCTCGCTGGACATGTCCAAGGTGCTTCAATCGGAGATCGAGGCGGAGCCGGAGAAGCTCGACGAGATCATGGCCAGCGGCGAGGAGTACGGCCTCTTTACGTCGCGGTTTGCCAAGCGCCACGGCCTCCACCTCCTCGGCACGGCGACGGCGTGGTTCCTGGTCGATGTCGCGTACTACAGCCAGAACCTATTCCAGAAGGACATCTTTGGCAGCATCGGCTGGATCCCCAAGGCGCGCACCATGAACGCGCTCGAGGAGGTGTTCCGCATCTCCCGCGCGCAGACGCTCATCGCGCTCTGCGGCACCGTGCCGGGCTACTGGTTCACCGTGTTCCTCATCGACGTCATCGGAAGGTT**C**TGGATCCAGCTCGTGGGGTTCGCCATGATGACCGTTTTCATGCTCGGCCTCGCGGTGCCTTACCACCACTGGACGACGCCGGGCAACCACGTCGG**CTTCGTCGTCATGTACGGGCTCACCTTCTTCTTCGCCAACTTCGGGCCGAACGCAACGACGTTCATCGTGCCGGCCGAGATCTTCCCGGCGCGGCTCCGGTCGACCTGCCACGGCATCTCGGCTGCGGCGGGGAAGGCCGGTGCCATCATCGGGGCGTTCGGGTTCCTCTACGCGGCACAGTCGCCGGACCCGGCGCACGTGGACGCCGGGTACAAGCCTGGGATCGGCGTGCAGAAGGCGCTGTATGTGCTCGCTGCGTGCAACCTCCTGGGGTTTTTGGTCACGTTCCTCGTGCCGGAGTCGAAAGGGAAGTCGCTCGAGGAGATGTCCGGCGAGGCCGACGCCGAGGAAGGCAACGGCGCCAATAAAGTCCGCCCGTCGGGAGAGCAGCTGGTTTGAatagttttgatcctcgagaaggcaacacacgcatacgtgcatg**tttgcatgcgacgagttttttttgtgtcgtgaccggaactttgtgtaggtgtatacagtaaattttgtcactgttacatggtaggcatcggttgatattacgcttgatcttagccaaaaggtcgacaaaaatatactatttgttagagaaaggaaacatcaagattttagatctctcgactttttaattttttttgcagagagaaacttgtgttacttaattaatgaagattagaatcaagatgacataaatgttttgagtcgaatgattgaactgtcattaacgatcgcgagaagatgcatcggttccaaaaaagagcgagaggcatggtttgcagatggaagcgctcaacggttg

**TaPht1;5 Chr4DL**

TRIAE_CS42_4DL_TGACv1_342800_AA1122460/ IWGSC WGA v0.4 chromosome 4D scaffold 4109

attcaaggcatagtccattgtcaagttgtgaatggatgtagcttaaagttctaggcagaagttcaacttaacagtctctgctgaaacactggtatattaaacaagtggtgagagatctgctgaaacactggtatattaaacaagtggtgagagaaggcaaatctctaaatgggtatttgagatctggtgggggattgttagaattaatgggctaggcccatagcaatttctgaaatctcaaagcccatgtgtaaaatggcaagtggtggtgctaagtttagtcccaccccggaagttgaagaagagttggacctatttatatagtgggttctctccaccactctaagtggtgtgtgagaagagaaagggaaaaccacacgcgcgcgctcgctcgcctcgcctcgcctcgctgggccgtggcgaggcgaggcgaggcggcgcgtatatgcgacatgcgcgtgaatggtccgccgaaatccagtccgtctccttgcaggagcgcagcttccttttgccgttttatttttaggtcttggcagacaagtttttgatttcttgtccggtaagtatacgaattagaaaccgagtcggtttgggattgtggtcgcgacacaataccacctctggtcctaatatatatacatctaccggctgcggccagagacacaccgaaaaacacctagggttttgcctcatctcacaacttgcgccgccatcgtagtctactccatcccaaacgccggcgtgcatcggcgcgtgggagagcaggtctccagaaccgttcgtctttgcgatcctgcaccgggagaggacgaattaggtttttgggaagcgttgtgcgcgactgctcaaattcgtcatcaagggtcgtcttccgtccaagtcgggcggtgctactcatcgtcgtattcatcgccgtcagcagcagatcgtcgccaacatcgtcatcaaccctgtcgcacccataatagctttttaaacgatcagtacgtccaacatcctctgttcatgtctgtttctacagctattgttacgtgtttgctgctgttatgcatgtcttgctgttcttctagtttgctagatttttgcatgctagtatctcttctagtcatgaattatttactgaaattaatcatgaacttgcctaatattccaacaaagatatggtgagcaaagcatccttcatggtgtataaggctcccatcgagtttgattgaaccaatacggaaggttagaccaccgtagggctagtgacataccctctaatactgccttgatctttgcttcaagtgcatcaatgcaataaaaactgtagcttacatgatgcaaagataacctggcgtgtgctaatctccagtatcataccaaagcctgtcgaaccaacctccgaagagaaggacccatccacagaaagagccagccagtcccgctggtggcgctggccatgggaaggttgtgaacatgctctggtaaacatcttcgtgaacatcccatatataatgtcacaatttctttcacatactccgacaaaaaccctaaataaaatgagtcctaggttgaagttcatgtcgtaggacttacttcaaagatgaacacgtcttagtaattttttttcttataagaaacagtagctacctttttaatgggctgcccatgtgagcgatgcctacctattgctcgcggtaggcagcacatacgttgtctcaagtctaaaaaaaaaaggtaggcagcacatgtactgatatcgtttccgccccggttttccctagctgtatccgtgcaagcaacagactatggctacctagctgccagtcgtccaactccatgccgaatcggttttgtctcccacgcatccaaatctataaatgtgccgctcgatcgccagggcgcagcagcgacagatcgaagcagcacgatcgccagggcgcagcagcgagagatcgaagcagcaacacgaactttggcacgagatctgcgacacggcgacaccccacggcgcgATGGCGAGCCGGCAGCTGCAGGTGCTGAGCGCGCTGGACGGCGCCAAGACGCAGTTGTACCACTTCAGGGCGATCGTTGTCGCCGGCATGGGCTTCTTCACCGACGCCTACGACCTCTTCTGCATCTCGCTCGTCACCAAGCTCCTCGGCCGCATCTACTACGCCGACCCCTCCAGCCCAAACCCTGGCTCCCTGCCGCCCAACGTGGCCGCGGCCGTCAACGGCGTCGCGCTCTGCGGGACCCTCGCCGGCCAGCTCTTCTTCGGCTGGCTCGGCGACAAGCTCGGCCGGAAGAGCGTCTACGGCATGACGCTCATGCTCATGGTCGTCTGCTCCGTCGCCTCCGGGCTCTCGTTCGGACACACCCCGGCCAGCGTCATGGCCACGCTCTGCTTCTTCCGCTTCTGGCTCGGCTTCGGCATCGGCGGCGACTACCCTCTGTCTGCCACCATCATGTCTGAGTACGCCAACAAGAAGACGCGCGGAGCCTTCATCGCCGCTGTCTTTGCAATGCAGGGCTTCGGCATCCTCGCCGGCGGCGTCGTCACGCTCGTCCTCTCCACGGTTTTCCGTAACGCGTTCCCGGGGCCAGCGTACCAGGTCGACGCCGCCGCGTCCACCGTGCCGCAGGCCGACTACGTGTGGCGCATCATCCTCATGCTCGGCGCGCTGCCTGCGGCGCTCACGTACTACTGGCGAACGAAGATGCCAGAGACGGCGCGGTACACGGCGCTGGTCGCAAAGAACGCGAAGCAGGCCTCGCTGGACATGTCCAAGGTGCTTCAATCGGAGATCGAGGCGGAGCCGGAGAAGCTCGACGAGATCATGGCCAGAGGCGAGGACTACGGCCTCTTCACGTCGCGGTTCGCCAAGCGCCACGGCCTCCACCTCCTCGGCACGGCGACGGCGTGGTTCCTGGTCGACGTCGCGTACTACAGCCAGAACCTATTCCAGAAGGACATCTTCGGCAGCATCGGCTGGATCCCCAAGGCGCGCACCATGGACGCGCTCGAGGAGGTGTTCCGCATCTCCCGCGCGCAGACGCTCATCGCGCTCTGCGGCACCGTGCCGGGCTACTGGTTCACCGTGTTCCTCATCGACGTCATCGGAACGTTCTGGATCCAGCTCGTGGGGTTCGCCATGATGACCGTTTTCATGCTCGGCCTTGCGGTGCCGTACCACCACTGGACGACGCCGGGCAACCACGTCGGCTTCGTCGTCATGTACGGGCTCACCTTCTTCTTCGCCAACTTCGGGCCGAACGCAACGACGTTCATCGTGCCGGCCGAGATCTTCCCGGCGCGGCTCCGGTCGACCTGCCACGGCATCTCGGCTGCGGCGGGGAAGGCCGGTGCCATCATCGGGGCGTTCGGGTTCCTCTACGCGGCACAGTCGCCGGACCCGGCGCACGTGGACGCCGGGTACAAGCCTGGGATCGGCGTGCAGAAGGCGCTGTATGTGCTCGCTGCGTGCAACCTCCTGGGGTTCTTGGTCACGTTCCTCGTGCCGGAGTCGAAAGGGAAGTCGCTCGAGGAGATGTCCGGCGAGGCCCACGCCGAGGAAGGCAACGGCGCCAATAAAGTCTGCCCGTCGGGAGAGCAGCTGGTTTGAatagttttgatcctcgagaaggcaacacacgcatacgtgcatgtttgcatgcgacgagttttttttttgtgtgtgtcgtgactagaactttgggtaggtgtatacagtaaattttgtcactgttacatggtaggcatcgttgatattacgcttgatcttagccaaaagggcgacaaaaatatactttttgttagagaaaggaagcatcaagattttagatctcttgacattttaaattttttgcaaagaaaaacttgtgttactcaattaatgaagattagaatcaagatgacataaatgtttttagtcgaacgattgaactgtcattaacgatcgggagaagatgcgtcggttccaaaaaagagcgagaggcgtcgtttgcggatggatccgcacaacg

**TaPht1;6 Chr5AL**

TRIAE_CS42_5AL_TGACv1_375089_AA1215870/ IWGSC WGA v0.4 chromosome 5A scaffold 6791

**partial RT-PCR fragment HG764740 (bold)**

caagcttgcccgctaaacctggcatccttggcaaacacaatttggcatgaaaaacgttcaatttgccatgcctaaaaatcaaacgttcgttgaatgtgtttagcaaccatggcaaatcctcgccatgtttagttttttgtcaggatttgacaagctgctaaacgaaattgccgtcaccacaatgactataatgatcataaaaacgttttatttgtcatgattaagaatctaactttagattttttttttagcattactgaataataaaacacggtaaatacaaggatttgctgtgtttttcccgcaaaaaaacggatttgccgtgtttagataaggacgagcgtcctttgactttttttttaaacatcagtacagacacaagcactcatatacacacgcatacactcatccctatgaatgcacacacgcataccctatccctatgagcatctccggaagagggcacctcgtcgtcgaaaatcctgaaataaactcaggaataaatgcaagcaccaggatttaaagcctggtgggttgggaataccacggtccctctaatcatctaaattgaattgcgttgcgttgatcattggtcacaactcacaacccatgcgcgtcgaggtccagaaggtgtgaggggcggacggacggtggtcatgcactgaacgtgacacagtcttaaacatttggctaagctgctggaggctgcagccagagagagccatgatagatcgggcaccaggaccagaggaccatgcgcgtcgaccgaccgcaggagttgactcgatctactgcgttaattgcctggccgggccggaaatttcgtggagccgtcgtcgtcgtggttgaccgacgaggctggccgggagcaaggcggggcggatatccccctgcagcgaactgactcactccgttttttttgtcgtcttcaaatcagatcagccgtcgatcgatccatcaccgaactcgataagctgagtgaagaatcgattctcaagattctgggagaattgttcaaaagaactgagcctatagccacgtcgcactctattctcttttattttgttttcaaaagtagcccaggcaaggcaatatttttacacttgtgtgcaagtagttctaatttttttctgcgttgaaaagtccacactctcccatggcatgcgatgcgagtgcaagcagtgccccgtcatgcactggcggagctacatgtaatgaaaatgggctatggcccgcccattccaaagctaatacagtgtaggattaaaataaactgagccacaaaaaaaaatataatgatcttcctttatattggcccgcccagctttggttgggtagctccgccactgccgtcacgtcaccttgttacttgccccgtatttcttcctgcatgcctccttgatcgtgacaacaacctcaatggtttcctcttttccaaccccaccccgccaacaaccacaaccgcacgcacgaatatctcatctcatctcacccaaatgaaaaatactacgtaacggatttttcttttattagtactattggaattagaattagaaagcgaaaaatgcagatatgattttctcgccaacaaaactggaataaggtaacatactgggcgaatattcccctcgagaattgaacaaaaacaactgccagccggttcgagtcgccagtaaaatcgtaactgcacgaggtttattcccagcgatcccaccaaaataccttttttttttttgagaataccaaaatacctttgatcccagcgttccctcacggcagtcaacaaccgagcccaagagtcccggggggaagcccttcaaaaaaaaaaaaaaagagtcccggggaagcaggagccgcgctatttatctccccccacccgacccccttcccccacaccccaccctaaaatctccaatcttctcatctttgatcgatcttctctccacagaggaaactgcagcgatccccggcgagagaccgagggaggagggctcgcggccATGGCGCGCGAGCAGCTGGAGGTGCTCACGGCGCTGGACGCCGCCAAGACGCAGTGGTACCACTTCACGGCCATCGTCATCGCCGGCATGGGCTTCTTCACCGACGCCTACGACCTCTTCTGCATCTCCCTCGTCACCAAGCTGCTCGGCCGCATCTACTACTACCGGGAAGGGGCGGACGCGCCCGGCTCGCTGCCGCCCAACGTCGCCGCCGCCGTCAACGGCGTCGCCTTCTGCGGCACGCTCTCCGGCCAGCTCTTCTTCGGCTGGCTCGGCGACCGCATGGGCCGCAAGCGCGTCTACGGCATGACGCTCATGTGCATGGTGCTCTGCTCCATCGCCTCGGGCCTCTCCTTCGGGTCCACCCCCGGCTCCGTCATGGCCACGCTGTGCTTCTTCCGCTTCTGGCTTGGGTTCGGCATCGGCGGCGACTACCCGCTCTCCGCCACCATCATGTCCGAGTACGCCAACAAGAAGACGAGGGGCGCCTTCATCGCCGCCGTGTTTGCGATGCAGGGCTTCGGCATCCTCACCGGCGGCGTCGTCACGCTCATCGTCTCCGCCGCCTTCCGCGCCGCCTTCCCCACCCAGGCCTACCAGGACGCCCCCCTCGCCTCCACGCCGGCGCAGGCCGACTTCGTGTGGCGCTTCATCCTCATGTTCGGCGCCGTCCCGGCCCTCATGACCTACTACTGGCGGATGAAGATGCCCGAGACGGCGCGCTACACGGCGCTCGTCGCCAAGAACGCCAAGCAGGCGGCCGCGGACATGTCCAAGGTGCTCCAGGTGGACATCGGCGCCGAGGAAGAGGACCCCAAGGCGAACGACGGCGTTGGAGCCGCCGACGACCGCAACTCGTTCGGGCTCTTCTCCGGCGAGTTCCTGCGCCGGCACGGGCTGCACCTCCTCGGCACGGCCACCTGCTGGTTCCTCCTCGACATCGCCTTCTACTCGCAGAACCTGTTCCAGAAGGACATCTTCACGGCGATCAACTGGATCCCCAAGGCCAAGACGATGAGCGCCCTCGAGGAGGTGCACCGCATCGCGCGCGCGCAGACGCTCATCGCGCTCTGCGGCACGGTGCCGGGCTACTGGTTCACGGTGGCGCTGATCGACCGGATCGGGCGGTTCTGGATCCAGCTCGGCGGCTTCTTCTTCATGGCCGTGTTCATGCTGGGGCTCGCCTTCCCGTACCACCACTGGACGACGCCGGGGAACCACATCGGGTTCGTGGTGCTGTACGCGCTCACCTTCTTCTTCGCCAACTTCGGGCC**CAACTCCACCACCTTCATCGTGCCAGCGGAGATCTTCCCGGCGAGGCTCCGGTCGACGTGCCACGGCATCTCGGCGGCGGCGGGGAAGCTGGGGGCCATCGTGGGGTCGTTCGGGTTCCTGTACCTGGCGCAGAACAAGGACCCGGCCAAGGTGGACCACGGGTACAAGGCCGGCATCGGGGTGCGGAACTCGCTCTTCATCCTCGCCGCCTGCAACTTCCTCGGCATGGGCTTCACCTTCTGCGCGCCCGAGTCCAACGGCATCTCGCTCGAGGAGCTCTCAGGCGAGAACGACGACGGCGAGGCGGCCGCGCCGGCGCACGCCAGGACGGTGCCCGTGTGAgacggacgtcgtcggacactagtggtggtatactatgcaggtgttggacggacgggtgtatggatgcttttcatggtttggaccgtgaaacgcggcgacgagtgcaacaaataatcgtgtgttggtgtgatgggtcgagttgtccaggttaattcatcagagttgctggtcgagtt**agcagtagtaaaatagggagtgatacaagtcgggtcgagtggtagtgcatcaaagaggcacgcatatgtacgcgatacgatggaaaactttcatgcattttggcgtgtgtggactggacaatatattatacagtacgtactaaattctagtattgctagttccgtacctaccttgaaaattcattttctatttatttatttatttatttttcacacaatagtat

**TaPht1;6 Chr5BL**

TRIAE_CS42_5BL_TGACv1_404641_AA1307160/ IWGSC WGA v0.4 chromosome 5B scaffold 50324-2

gtccggccagctgacgcagcggacaaaagcaaccagcgagagtatccgtttatgtcctcacaaatccatctgtggtttaaatttgtttcagatttgaagtcgtgccagacaaaaaagggacacctgcagacaattttttttgtttggatacccccccattgggccaagttttgctctaaacggacacgatcggacgatttgtcatctaccgagttctaaaaaaaaagaaaaatgttgtctagcgttggaattggccttgttttttcccgatatatattgtctaaaaattagttgtagcaagtccagaaggtgtgaagggcggacggacggtggtcatgcactgaacgtgacacagttttaaacatttggccaagccgttggaggctgcagagagagagacagagagaacgccatgacagatcgggcaccagtacaagaggggcacacggtgtgcgtcgaccgaccgcaggagttgactcaatcgaccgagttaattgcctgtccgggccggaaaattcatggagccgtcgtcgtcgtcgtggttgacgggcgaggctggctgggagtgaggtggagcggatattccccccgccgatccctgccgtgtgcaatccacactgtgcgcggcctccataaatagggcctccccgccggcggcctcctccaaccattcctcggacaatcttcttcctctacactctagctcatcttgcccatgcatttctccctggcttgactggccgttgatgaagctgcaggagccagccccctagctagctatctgtctgcctagctgcttcgtcggtgagtcctgcttcctcgcccgctcgctcgctggccggacttgtattaacaatactatttatttcccttctcaattggtgatcggttttcagtttggtagtagtagtagcagcagattcttttgtgatcactggacttgcacatgcatgattccggctcttaaactgctgcagcacatcatcagttgttggtagctaggagtagctccggtggtgccccgaagtcgagaaaagccaagagaatcttccaaatttcgtctttctttctgactagcaagttgtggtgaggtgagtggaggcagtatcctttcctccggaactgaaactcaactcaaaaggcaggcggatcaaatcaacccaaccccacctgcacctgcagcgaactgactcactgactggctctgttttcttgtcttcttcagatcagatgagccatcgatccatcacggagctcgatcgctccgtagccacgtcgcactccattctcttttcttttcttttcttttcttttcaaaagtagcccaggcaaggcaatatatttacacttgtgtgtgcgcaactagttctatatattttttttcctttgaaaagtccacacccccccatgcatgcgatgcgagtgtcgaacagtgccccgtcacgtcaccttataacttgccccaaatttcttcacgcctgcctgactccttgatcgtgacaagagccttcattggtttcctcttttccaaccctacctcaacaatcacaaccgtacgcacgaatatctcatctcgcctgaaaaataacggatttctcttttctttttattattattagaaaagcaaaaattgcagatatgattttctcgccaacaaaattggaataagataacatgccgggcgaatattcccccggagcgtagaacaaaaacaactgtcagccgcttcgactcgccagtaaaatcgtaacagccaaggtttattcccaacgatcccaccaaaataccttttgatcccagcgttcacagcagtcaacaaccaagcccaagagtcccggggaagcagcaggagccgcagtatttatctcatctctcatcccacccgacgccctccatcctcctcccaccccaccctaaaatctccaatcttctcttgacaggaacaagagatccccggcggcgatccccggcgagagatcagtcaaggagggctcgcggcgATGGCGCGCGAGCAGCTGGAGGTGCTGACGGCGCTGGACGCGGCCAAGACGCAGTGGTACCACTTCACGGCCATCGTCATCGCCGGCATGGGCTTCTTCACCGACGCCTACGACCTCTTCTGCATCTCCCTCGTCACCAAGCTGCTCGGCCGCATCTACTACTACCGCGAGGGCGCCGACGCGCCCGGCTCGCTCCCGCCCAACGTCGCCGCCGCCGTCAACGGCGTCGCCTTCTGCGGCACGCTCTCCGGCCAGCTCTTCTTCGGCTGGCTCGGCGACCGCATGGGCCGCAAGCGCGTCTACGGCATGACGCTCATGTGCATGGTGCTCTGCTCCATCGCCTCCGGCCTCTCCTTCGGCTCCACCCCCGGTTCCGTCATGGCCACGCTCTGCTTCTTCCGCTTCTGGCTCGGCTTCGGCATCGGCGGCGACTACCCGCTCTCCGCCACCATCATGTCCGAGTACGCCAACAAGAAGACGAGGGGCGCGTTCATCGCCGCCGTCTTCGCCATGCAGGGCTTCGGCATCCTCACCGGCGGCGTCGTCACGCTCATCGTCTCCGCCGCGTTCCGCGCCGCCTTCCACACGCCCGCCTACCAGGACGCCCCCCTCGCCTCCACGCCGCCGCAGGCCGACTTCGTGTGGCGCTTCATCCTCATGTTCGGCGCCGTCCCGGCCCTCATGACCTACTACTGGCGGATGAAGATGCCCGAGACGGCGCGCTACACGGCGCTCGTCGCCAAGAACGCCAAGCAGGCCGCGGCCGACATGTCCAAGGTGCTCCAGGTGGACATCGGCGCCGAGGAAGAGGACCCCAAGGCCAACGACGGCGGCGCCGGAGCCGCCGACGACCGCAACTCGTTCGGGCTCTTCTCGGGCGAGTTCCTGCGCCGGCACGGGCTCCACCTCCTCGGCACGGCCACCTGCTGGTTCCTGCTGGACATCGCCTTCTACTCGCAGAACCTGTTCCAGAAGGACATTTTCACGGCGATCAACTGGATCCCCAAGGCCAAGACGATGAGCGCCCTCGAGGAGGTGCATCGCATCGCGCGCGCGCAGACGCTCATCGCGCTCTGCGGCACGGTGCCGGGCTACTGGTTCACGGTGGCGCTGATCGACCGGATCGGGCGGTTCTGGATCCAGCTGGGCGGCTTCTTCTTCATGGCGGTGTTCATGCTGGGGCTCGCCTTCCCGTACCACCACTGGACGACGCCGGGGAACCACATCGGGTTCGTGGTGCTGTACGGGCTCACCTTCTTCTTCGCCAACTTCGGGCCCAACTCCACCACCTTCATCGTTCCCGCCGAGATCTTCCCGGCGAGGCTCCGGTCGACGTGCCACGGCATCTCGGCGGCGGCCGGGAAGCTGGGCGCCATCGTGGGGTCGTTCGGGTTCCTGTACCTGGCGCAGAACCAGGACCCGGCCAAGGTGGACCACGGGTACAAGGCCGGCATCGGGGTGAGGAACTCGCTCTTCATCCTCGCCGCCTGCAACTTCCTCGGCATGGGCTTCACCTTCTGCGCGCCCGAGTCCAACGGCCTCTCGCTCGAGGAGCTCTCCCGCGAGAACGAGGAGGAGGCGCCGGACGCCAGGACGGTGCCCGTGTGAgaaacacgtcgtcagatagtagtatattctgcgagcaggtttggatggatgtatggatgttttttatggtcggagagcgttcaactcaggagtcgagtcgagtcgagtacaacaataatttatctaatcttgtgatgggtcgagttgtccaggttaattagggctgctgctcgagttattagcagtaatataggtacggtgtaataccaagttagagaagtgcatcgaagaggcaaagcttatatgtacgtgacgtgtgattgaaaattttcatgcattataaggggttttgcatattttaatactgcatatgcataacctacatatggactgaaatgactgaacaaacacgctgaaaaatgttcaattcgggaaaaaagaacaacttataacagtgaac

**TaPht1;6 Chr5DL**

TRIAE_CS42_5DL_TGACv1_434312_AA1433620/ IWGSC WGA v0.4 chromosome 5D scaffold 82635

**partial RT-PCR fragment HG764739 (bold)**

ttgggcacgtcccgtttgggccaagtttggctctaaacgaaagcggccggacgatttatcgtctggcgacttctcaaagaaagaaaaaaaggaaaatgtcgtctagcgttggagttggcttactttttccaaatatatattgtctaaaaattagttcttcactaggaagtccagaaggtgtgaagggcggacggacggtggtcatgcactgaacgtgacacagttttaaacatttggctaagctgctggaggttgcagagagagccatgacagatcgggcaccagtgccagagcacacggtgtgcgtcgaccgaccgcaggagttgactcgatcgagtcgagttaattgcctggcctggccggaccggaaatttggtggagccgtcgtcgtcgtggttgaccgacgaggctggccgggagtgaggtggagcggatattcccccctccgatccctgccgtgtgcaatctacactttgcgtggcctccataaatagggcctccccgccggcggcctcctccaaccattccttggacaatcttcttcctcttcccctgctatacactctagcccttcttgtccgtgcatttctccctggctttgactggttgttgatgaagctgcaggagccagccccctagctatctgtctgtctagctgcttcgttggtgagtcctgtttccttgctcgctcgccggacttgtattactagcattaattattttctttctcagttagtgatcgattttcattcagtttggtagtagctagctctgctccggtgacgctcatagccatataatcatcttcacacacacataaaaagaggagtctggattagtatcacgcgcgcacacacacgggtcccatttgattcgagtaactcgaatacatgcatgcatcacgcatcacgggctactgccctctccctacctccatcctccatcgtagcattcggagtgagtggtaccccgaagtccagaaaagccaagagaatattccaaatttcgtctttctttctaactactcaagtggaggcaagttgtggtgagtggaggcaagtatcctccggaactgaagctcaactcaaaaggcaggcggatcaaatcaacccaaccccacctgcacctgccgcgaactgactcactgactcactgcctctgtttacttgtcttgttcaaaaatatcacatcagccatcgatccatcacgaaactcgatcgctccatagccacgtcgcactcctttttccttccttttcttttgaaaagtagcccaggcaaggcaacatattttttcccgcaaaaaaaaaggcaatatatttacacttgcaactagtactagtactcatttttttctccgttgaaaagtccacggtctcccatgcatgcgatggagtgcgagcgtcaagccgtgtaaacccatccagggagccccgtcacgtcaccttataacttgccccgtatttcttcctgcctgcatgcctccttgatcgtgacaacagccttcattggtttcctcttttccaaccccaccccctcaacagccacaaccgcacgcacgaatatctcatctcatctcacccaaataaaaaatactaacagatttttcttttattactattagaattagaaagcgaaaatgcagatatgattttctcgccaacaaaactggaataggattacttaccacgcgaatattccccaggagagttgaacaaaaacaactgtcagccggttcaactcgccagtaaaatcgtaacagccaaggtttattcccaacgatcccaccaaaatacctttgatcccagcagtcaacaaccaagcccaagagtcccggggaagcaggagccgcactatttatctcctccaacccggcgccctccctcctcgtctcatcccaccctaaaatctccaatcttctcatcttctctcaacaggaacgaacgagagagcaccggcgacgatccccggcgagagatcaatcaaggagggctcaccgcgATGGCGCGCGAGCAGCTGGAGGTGCTGACGGCGCTGGACGCGGCCAAGACGCAGTGGTACCACTTCACGGCCATCGTCATCGCCGGCATGGGCTTCTTCACCGACGCCTACGACCTCTTCTGCATCTCCCTCGTCACCAAGCTGCTCGGCCGCATCTACTACTACCGGGAGGGCGCCGACGCCCCCGGCTCGCTCCCGCCCAACGTCGCCGCCGCCGTCAACGGCGTCGCCTTCTGCGGCACGCTCTCCGGCCAGCTCTTCTTCGGCTGGCTCGGCGACCGCATGGGCCGCAAGCGCGTCTACGGCATGACGCTCATGTGCATGGTGCTCTGCTCCATCGCCTCCGGGCTGTCCTTCGGGTCCACCCCCGGCTCCGTCATGGCCACGCTCTGCTTCTTCCGCTTCTGGCTCGGCTTCGGCATCGGCGGCGACTACCCGCTCTCCGCCACCATCATGTCCGAGTACGCCAACAAGAAGACGAGGGGCGCCTTCATCGCCGCCGTCTTTGCGATGCAGGGCTTCGGCATCCTCACCGGCGGCGTCGTCACGCTCATCGTCTCCGCCGCCTTCCGCGCCGCCTTCCCCACGCCCGCCTACAAGGACGGCGCGCTCGCCTCCACGCCGCCGCAGGCCGACTTCGTGTGGCGCTTCATCCTCATGTTCGGCGCCGTCCCGGCCCTGATGACCTACTACTGGCGGATGAAGATGCCCGAGACTGCGCGCTACACGGCGCTCGTCGCCAAGAACGCCAAGCAGGCCGCGGCCGACATGTCCAAGGTGCTCCAGGTGGAGATCGGCGCCGAGGAAGAGGACCCCAAGGCCAACGACGGCGGCGCCGGAGCCGCCGACGACCGCAACTCGTTCGGGCTCTTCTCCGGCGAGTTCCTGCGCCGGCACGGGCTGCACCTCCTCGGCACGGCCACCTGCTGGTTCCTGCTCGACATCGCCTTCTACTCGCAGAACCTGTTCCAGAAGGACATCTTCACGGCGATCAACTTGATCCCCGAGGCCAAGAAGATGAGCGCCCTCGAGGAGGTGCACCGCATCGCGCGCGCGCAGACGCTCATCGCGCTCTGCGGCACGGTGCCGGGCTACTGGTTCACGGTGGCGCTGATCGACCGGATCGGGCGGTTCTGGATCCAGCTGGGCGGCTTCTTCTTCATGGCCGTCTTCATGCTGGGCCTCGCCTTCCCGTACCACCACTGGACGACGCCGGGGAACCACATCGGGTTCGTGGTGCTGTACGCGCTCACCTTCTTCTTCGCCAACTTCGGGCC**CAACTCCACCACCTTCATCGTGCCGGCGGAGATCTTCCCGGCGAGGCTCCGGTCGACGTGCCACGGCATCTCCGCCGCCGCCGGGAAGCTGGGGGCCATCGTTGGGTCGTTCGGGTTCCTGTACCTGGCGCAGAACAAGGACCCGGCCAAGGTGGACCACGGGTACAAGGCCGGCATCGGGGTGAGGAACTCGCTCTTCATCCTCGCCGCCTGCAACTTCCTGGGCATGGGCTTCACCTTCTGCGCGCCCGAGTCCAACGGCATCTCGCTCGAGGAGCTCTCCGGCGAGAACGACGACGGCGAGGCGGCCGCGCCGGCGCACGCCAGGACGGTGCCCGTGTGAgacagtcctcgtcagatagtatattctgcaggtttggatggatgtatggatcattttaatggttggagcgttcaaactcaggagtcagagtacaagaataatcgtgtgatgggtcgagttgtccaggttaattcattagagttgctggtcgggtt**agcagtaatagggagtgatacaagttaagagtgtagtagtgcatcaaagaggcacgcatatgtacgcgatatgatggaaaactttcatgcattttggcctgtgtggactggacaatatattatacagtactaaattctatcgctagctcctactacctaccttgaaatttcattttctatttatttatttatttaattaattttcacacagtagctacacacgagtatataagttgactgaattt

**TaPht1;7 Chr5AL**

TRIAE_CS42_5AL_TGACv1_377225_AA1245130/ IWGSC WGA v0.4 chromosome 5A scaffold 34444-3

**partial RT-PCR fragment HG764741 (bold)**

agtagcaaaattgttccacgaaggtggaagcttggcaatgatgcctccggcaacaaatttttccaacaacacacacttgaagtactcaaattcttttgcgagcgactgtatctcatgagcctgctgtacaacatggcgctcatcagtcatcttgtagtcatagaattgctccatgaagtacaactcgcagccggcgtctgaggcaccaaacttggcctcgagcgcagcccacatgtccttgccgttgtcaaatgacatatacgaatccacaatggagtcatcaagaacactcagaagagcgcctttaaagagggtatcgatcttctcaaaagcttctagctgtgctggattaagatcgccctcaggcttgcccttggtggcatcatagcagctcatggtctgaaaccagtagactgctctcgtgcgccacctcttatattgcgcccccttaaaggcaggcggcttcagatgcgcagcaaaaccactcggagtaaattgcctataatcaggtttttggattgttggaaatatgagcaaattactacgagatttaatccgaataaacagaagataaatcatgaccacagcagcagagattaaactaattatgcgaactagcatagcagatgaacatatcacatctagggcacatactagaaacatgaattctaccacgatctcgaacaggaaggatagaatcacatacgttgcagcgggtgcagcaccgccggcgttgacgttgcgcccatgtcgtcgaggacgaggttgccgaggtcggggaagaagtcgtcgttcgcgaagtcgtcgctgccagcagtcgcgcgagtgcgctccctaaaaacctgatcacccctctcccgtacaggatcacgagaggcggggtttcggaggcctgctgtgccttctcgcggtgcatgccggaaggagggatggagaagacttgcttggcggcgcaatgatctggaacggtgatgagaaaccatacgaagcggcggcggctagggtagacgttcggaacccgcggcgcggcgcgtcgtgacgaggcgtggcgtggcgaggcgagcgaggaggaggagcgcgcgtgtaggtctcctcttctcatgctcatacaagtggtagaagaactcaccttataaagaggtgcaactctctctcaacttctggggtgggactaaactttagcctcactcactccactcacatgtgtgcatgaatgggccaagagaatttcagaattttagttgggatttgggccaaagggctactagcaaaattccaacaatcccccacaaagtctcattggcacatctcatcatttagttccaaaacattgttttatatatcggtgcttagtggaaactgtgaagttgaacttccacttagaaatttatgctacactagatcacaacttgaatagtggaacttgacaagtcaaagcaactttaacatcacggtgttattcggattttttggtacacttgtaggttacacgcatgttttggttcatgtgacggagaccatgttctggttcatgtggcgaagtttgatcatgtgtggctatatacacaaaaatgtacggatcttggatactgcagactaaacaagaatatgcccctggctccaaacattttcattttcacatagaaaacgaaagcaaaacctccgcagtccgcatgcacgtaaaagggcttcgcgatgcacgggaaactctgagcccctccatgttgttctttgcacaagggaaccgatttttcttttgggggagagaagagaaccgttaaacagggagaagttgttggtctccaaggaacgaaggaccaggagccccgaatatgctcgcctctccgtatatacatggctggcagacacctgtgccttctccctcagttcactcccatcctaaacctcaacaaccttccaccgcggcggatcgcaccaccactttcgccgccggccggaacgcacgcataggcatagaggttggcgcgcgcgcggtggcaATGGCGGGCGACCAGGTGCACGTGCTCTCGGCGCTGGACGGCGCCAAGACGCAGTGGTACCACTTCACGGCCATCATCGTCGCCGGCATGGGCTTCTTCACCGACGCCTACGACCTCTTCTGCATCTCCCTCGTCACCAAGCTCATCGGCCGCATCTACTACACCGTCCCGGGCTTGCCCCGCCCGGGAAGCCTCCCGCCGACCGTCTCCGCGGTCGTCAACGGCGTGGCCTTCGTCGGCACGCTCTCCGGCCAGCTCTTCTTCGGCTGGCTGGGTGACAAGGTCGGCCGGAAGAGCGTGTACGGCATGACGCTGATGCTGATGATCCTCTGCTCCGTCGCGTCGGGGCTCTCGTTCGGCAACACGCCCACCAGCGTCATGGCCACGCTCTGCTTCTTCAGGTTCTGGCTCGGCTTCGGGATCGGCGGCGACTACCCTCTCTCCGCCACCATCATGTCCGAGTACGCCAACAAGCGGACGCGCGGGGCGTTCATCGCCGCCGTCTTCGCGATGCAAGGGTTTGGCATCCTCGCCGGCGGCGGCGTGGCTATCGGGATCACGGCGCTGTTCAGGGACCTGTTCCCGGCGCCTCCGTACGCGGCGGACCCCGCGGCGTCCACCCCGGCGCAGGCGGACTACGTGTGGCGCATCGTGCTCATGCTCGGCGCGCTCCCCGCCGCGCTCACCTTCTACTGGCGGATGAAGATGCCGGAGACGGCGCGGTACACGGCGCTCATCGCCAAGAACGCCGAGCGCGCCGCGGCCGACATGTCCAAGGTGCTCCACGTGGAGATCACCAAGGAGCAGGCCGGCGATCTGGAGACCGTGATTTCCATCAAGTCCCACACGCCGCCGCCGTCGTTCGGCCTCTTCTCCCGGGAGTTCGTGCGCCGGCACGGGCTCCACCTCGTCGGCACCGCGTCGACGTGGCTCCTCCTGGACATCGCCTACTACTCGCAGAACCTGTTCCAGAAGGACATCTTCAGCGCCATCGGGTGGATCCCGCCGGCGGCGACGATGAGCGCGCTGGACGAGCTGTTCCACATCGCGCGGGCCCAGATCCTGATCGCGCTCTGCGGCACCGTGCCGGGCTACTGGTTCACCGTCGCCTTCATCGACTCCGTCGGCCGCTTCAAGATC**CAGCTCATGGGCTTCTTCATGATGACCGCCTTCATGGTCGGCCTCGCCGTGCCCTACGACTACTGGACGGGCCAGGGCCACCAGGCCGGCTTCGTCGTCATGTACGCGCTCACCTTCTTCTTCGCCAACTTCGGGCCCAACGCCACCACCTTCATCGTCCCCGCCGAGATCTACCCCGCCAGGCTCCGCGCGACGTGCCACGGGATATCGGCCGCCTCGGGGAAGGTGGGCGCCATCATCGGGTCCTTCGGGTTCTTGTACCTCGCCCAGAGCCCCGACCCGGCCAAGACCGCCCATGGATACAAGCCCGGCATCGGCGTGCGCTGCTCCCTCCTCGTGCTCGCTGGGTGCAGCTTGATGGGGTTCATGCTCACCTTCCTCGTCCCGGAGCCCAAGGGCAAGTCCTTGGAGGAGATGTCGCGCGAGACCGAGCCCGACCATTGCTAGCTAGggggcgtctcgtcgttgcgtcaagatccgctgcgtagctagtccatctactcctagatgactgtccgtgaacact**gcatttagttttggcaattaggggatggcgacatggtgaaataatcgtcgtcacagaaattaaagcgcgtaagctttattttttcttgttttttgaccttttctctttactgtggtgtgaaaaattaggtggctgccattgggaagctatttacctgagaagggctttgggtatgttctgacatacatctagccagttttgaccgagtcactcaatctttttccaactgctcaacgtgagtaaggctagtcatagtgggagtaacttagctagctagtaacatagcgcacttcaagaaatttttacttatgtggcaagtatttaa

**TaPht1;7 Chr4BL**

TRIAE_CS42_U_TGACv1_643698_AA2134370 – chr4BL/ IWGSC WGA v0.4 chromosome 4B scaffold 85840

atagtgggaagcattggtggtgagtatattctttcgcaaaaactaaatggagtgacaactgtggcactataattgggaaggacaacttttttcttcacgagatactaattcttttttcttaaatatcttctcgataaatacatgatttgtttgtgaaattcatatgctctatggtgttgttctaatggtatatgcttgttgacgatatatgggcacatattggaagactatcatacatgttagatggtgaccatgtttcacaggtcatggataagccgacatcagtcccattagtatggattcatgttggtagaacacgttgtcaaacatgcatattttgagacgtaacaagaagtgccatgttttattttcaagtgcaatatttatgctatgtcctagacttgaagtcgtcccatgttttgctaacatgaaacaaccttcttagggactatgaggagaggggattcaaacccgagacctgcaagaaccaatagactcatgtacatcttatttttctaaggataaccatttaattttgatttcatttgattatttttagctaacaatagccaacatttccctacaatattggtaatgagttattaggtggtaacaatccttgatgttaaactcatggtaataaagtattggaaaagttgtgaatgggcaaggatgtattaactttagacatgattttgtaatatatattttgcatggggatcttgcgatactataatataacataacaaaaattctacaaaaccatggctatagtacaacactataaaatgttaaacacaaaacatcaggaacacgcaattgtacttggtacacacaagatgaaggattggtagcctataaatacaaattcatagaaatgcatgcctccccccccctccccaaatggcacaacatatatcaccatgaaactttacaaatcaaagcaactttaatcaattaaaaaagcaaccttaacacgacggtgtatacaatcttatctggaattcttggtacagtagtaggttacacgcgagcccctctcaacagcgaaggctggcatgttttggttcatgtgacatagtttgatcatgtgtggatatataaacacaaaaaagtaaggacgttggatactgcagacaaagggattggctataggtaagtcgactgaattttcaaagtcggccagtcgattcattttgtatgccagatgcagatctaacgacatgcgacccttcttcttcctctcacctacgacctcccaatgttccaccggccgaaccgccggccaccaccacccgcagtggcggagctacgttgaaggctgcaggggccatggccccccagccttggtcaaatttgtgaaagaatttccgcctcgccacactgccctcccctcaacctcccctcaccaccagtcttttgccgcccatggcaatccctctagccccggcctcgccggttttctgtccggcgaatctgcaccattgcccccaccctaaaccctaagtatagataccggggtagcctgcccaacaagcttcttcctcccctccgacgagtttcttcctcccctccgactatttcatcaaccaaaattgattcaccccaaattcgaaattcaggtgattgaatgtacctattgtggcagacaaaacaagaatgtgtcccccgcatgcgcgcaaaagagcttcgctacgcacgggaaactctgagcccatccatgttttttctctacaaagaagagaatttatttttgcgggagagaagagaaccgttaaacagggagaagtagttggtctcgaaggaacgaaggaccaggagccccgaatatgcccacctctccgtatatacatggctgacagacacctgtgccttctccctcagttcactcccatcctaaacctcaacaaaccttccaccgcgccggatcgcaccaccactttttttcgccgcctgccggaacgcacgcatagagagaggttggcgcgcgcgcggtggcaATGGCGGGCGACCAGGTGCACGTGCTCTCGGCGCTGGACGGCGCCAAGACGCAGTGGTACCACTTCACGGCCATCATCGTCGCCGGCATGGGCTTCTTCACCGACGCCTACGACCTCTTCTGCATCTCCCTCGTCACCAAGCTCATCGGCCGCATCTACTACACCGTCCCGGGCTCGCCCCGCCCGGGCAGCCTCCCGCCGACCGTCTCCGCGGTCGTCAACGGCGTGGCGTTCGTCGGCACGCTCTCCGGCCAGCTCTTCTTCGGCTGGCTGGGTGACAAGGTTGGCCGGAAGAGCGTGTACGGCATGACGCTGATGCTCATGATCCTCTGCTCTGTCGCGTCGGGGCTCTCGTTCGGCAACACGCCCACCAGCGTCATGGCCACGCTCTGCTTCTTCAGGTTCTGGCTCGGCTTTGGGATCGGCGGCGACTACCCGCTCTCCGCCACCATCATGTCCGAGTACGCCAACAAGCGGACGCGCGGGGCGTTCATCGCCGCCGTCTTCGCGATGCAGGGGTTTGGCATCCTCGCTGGCGGCGGCGTGGCGATCGGGATCACGGCGCTGTTCAGGGACCTGTTCCCGGCGCCGCCGTACGCGGCGGACCCCGCGGCGTCCACCCCGGCGCAGGCGGACTACGTGTGGCGCATCGTCCTCATGCTCGGCGCGCTCCCCGCCGCGCTCACCTTCTACTGGCGGATGAAGATGCCCGAGACGGCGCGGTACACGGCGCTCATCGCCAAGAACGCCGAGCGCGCCGCGGCCGACATGTCCAAGGTGCTCCAGGTGGAGATCACCAAGGAGCAGGCCGGTGATCTGGAGACCGTGATTACCATCAAGTCCCACACGCCGCTGCCGTCGTTCGGCCTCTTCTCCGGGGAGTTCGTGCGCCGGCACGGGCTCCACCTCGTCGGCACCGCGTCGACGTGGCTCCTCCTGGACATCGCCTACTACTCGCAGAACCTGTTCCAGAAGGACATCTTCAGCGCGATCGGATGGATCCCGCCGGCGCCGACGATGAGCGCGCTGGACGAGCTGTTCCACATCGCGAGGGCCCAGATCCTGATCGCCCTCTGCGGCACCGTGCCGGGCTACTGGTTCACCGTCGCCTTCATCGACTCCATCGGCCGCTTCAAGATCCAGCTCATGGGCTTCTTCATGATGACTGCCTTCATGGTCGGCCTCGCCGTGCCCTACGACTACTGGACGGGCCAGGGCCACCAGGCCGGCTTCGTCGTCATGTACGCGCTCACCTTCTTCTTCGCCAACTTCGGCCCCAACGCCACCACCTTCATCGTCCCCGCCGAGATCTACCCCGCGAGGCTCCGCGCGACGTGCCACGGGATATCGGCCGCCTCGGGGAAGGTGGGCGCCATCATCGGCTCCTTCGGGTTCTTGTACCTCGCCCAGAGCCCCGACCCGGCCAAGACCGCCCATGGATACCACCCCGGCATCGGCGTGCGCTACTCGCTCCTCGTGCTCGCTGGGTGCAGCTTGATGGGCTTCCTGTTCACCTTCCTCGTTCCCGAGCCCAAGGGCAAGTCCTTGGAGGAGATGTCACGTGAGACCGAGCCCGACCACTGCTAGgtagtctatctactcctagatgactgtcggtgaacactgcattcagttttggcaattaggggatggcgacatggtgaaataatcgtcagaattattttaagaagtagatgttcttctttctctgtatcgtagtacggttgaataggataaataacattttatttgacctatcattgaggtttgggggatttttgcccacacccccacccatcaccagcaacactaaaatttaggacgactattccaatgtgtttcttagcaagtttatttctcagagtataaacgtgtaaccagagaagtggtttgagggaagacgcctcacaacatgggcgatcggctcatatatatatatgactcggttacaaaggcttggaagccaagaaacaatctaaatgtatcaacaacagatcgggaagatacggtatgtacaatacgttttcatctatacaagatctacatacgttaacacagaaccctcgttgtgtgttacaatactacaattatggagatatgatttaagcacatcaaggataaaatagacatccccgcaaaaaaaggataaaataaacatgcaaaccaggtgagctgccctcgaaaccatatacgctaagagcggttttggagtggctccgccacatctagggatgacaacattgtgaaataatcacagaagttgttcttagaagtagaagctcttctctttacctgtcgaactagagt

**TaPht1;7 Chr4DL**

TRIAE_CS42_4DL_TGACv1_342927_AA1125460/ IWGSC WGA v0.4 chromosome 4D scaffold 58635-1

catgcatgatcaacctacatcacttttcccatatgaaactggcccctttgaatcaaagaggctctataaatctgtgttttctcaaacttgacttctccttaacttgtaatctgaaattagagtatgtggcgcacgatgctgaagtttagaccttggccattgggagaagtcgtgcagtgtgacattttcaaaattggcaagtagactgctcccctgtgacattatgagaatgatactacagtatctttggctttataatttgactcatgttgagttttcaaatgggtgtcttcttgtttgcgcagatcttgtcaggagcttgtgcttgctggcatcaagctatggacttgcaagaaaaagatataaggcctcctttggttcataggaattttgtagaaattctagaggataggaactttgtaggaaaaattcttttggagccctttggtttgtaggaacggattcctattcctatgtatggtaggaatcaatccttcacattttaaaggaaaaaaacattagtctagactcaatggaaaaattcttatcctatgcatcaaatgacatatctttttctataagaattgagatgcatgtcatctcacttcctttatttttcctattcctatattattcttatcctatgaaccaaaggaggcctcaaggtgtgtttggtgcttatatgagatcccagaagatgattatatgtactactttgctttattctttgtatttatctgtatattagtaaacagatctagagcatagccattgcattatgtggtatcggtgtgtgttcactgcaatctttttgtatattcataaacgtggaaatactctgtcagagtgggtcaatttgtatatctgagtgtctgcacatgaatcttgactatatatagcaatataggaacaagaaccccttaaattttgtcatgtcacatctactgacttttccttccaaatgtttaagtattgagatttttaaaagaacatattttgtttttgtaatgtagcataaacattcaacaaccatgtattgttagcatgatctgagaacaaatttgagtgcacaaggacgagaaatatcattttgatcatgtgtcataaaacataagttcttttttttttgcggggacataaaacataagttcaatgcgatgtgtaatttgtgagaggtctgcaagacgtgattactgcaagtttaaaccctggggtttcaaaggcacctcaaagtgtcactgaaatagcagagtaaattgaattgaaaacccgattgaaagaaaggcttgctgatgatataaaaggagttaacgacttgttcatcacacaaagttgagaactggaaaaatatatgcgccccccgtgcaacgcgcgtgcattgtcctagtaaatataaattcatagaaatgactctattttttcccaaacggcacgacatatcaccatggaacttgacaagtcaaagcaactttaacatcacggtgtatcgaatttgattcggattttttggtacacgtgtactaggttacacgcatgttttggttcatgtggcgaagtttgatcatgtgtggctatatacacaaaaatgtatggatcttgggtactgcagactaaacaagaatatgcccttggctccacacattttcgcatagaaaacgaaagcaaaacctccgcatgcacgtaaaagagcttcgcaatgcatgggaaactctgagcccctccatgttgttctttgcagaagggaatcgatttttcttttggggcagagaagagaaccgttaaacagggagaagttgttggtctcgaaggaacgaaggaccaggagccccgaatatgctcagctctccgtatatacatggctgacagacacctgtgccttctccctcagttcactcccatcctaaacctcaacaaccttccaccgcggcggatcgcaccaccactttcgccgccggccggaacgcccgcatagagagaggttgtcttggcgtgcggtggcATGGCGGGCGACCAGGTGCACGTGCTCTCGGCGCTGGACGGCGCCAAGACGCAGTGGTACCACTTCACGGCCATCATCGTCGCCGGCATGGGCTTCTTCACCGACGCCTACGACCTCTTCTGCATCTCCCTCGTCACCAAGCTCATCGGCCGCATCTACTACACCGTCCCGGGCTCGTCCCGCCCGGGCAGCCTCCCGCCGACCGTCTCCGCGGTCGTCAACGGCGTGGCCTTCGTCGGCACGCTCTCCGGCCAGCTCTTCTTCGGCTGGCTGGGTGACAAGGTCGGCCGGAAAAGCGTGTACGGCATGACGCTGATGCTGATGATCCTCTGCTCTGTCGCGTCGGGGCTCTCCTTCGGCAACACGCCCACCAGCGTCATGGCCACGCTCTGCTTCTTCAGGTTCTGGCTCGGCTTCGGCATCGGCGGCGACTACCCGCTCTCCGCCACCATCATGTCCGAGTACGCCAACAAGCGGACGCGCGGCGCCTTCATCGCCGCCGTCTTCGCGATGCAGGGGTTCGGCATCCTCGCCGGCGGCGGCGTGGCGATCGGGATCACGGCGCTGTTCAGGGACCTGTTCCCGGCGCCGCCGTACGCGGCGGACCCCGCGGCGTCCACCCCGGCGCAAGCGGACTACGTGTGGCGCATCGTCCTCATGCTCGGCGCCCTCCCCGCCGCGCTCACCTTCTACTGGCGGATGAAGATGCCGGAGACGGCGCGGTACACGGCGCTCATCGCCAAGAACGCCGAGCGCGCCGCGGCCGACATGTCCAAGGTGCTCCAGGTGGAGATCACCAAGGAGCAGGCCGGCGATCTGGAGACCGTGATTACCATCAAGTCCCACACGCCGCCGCCGTCGTTCGGCCTCTTCTCCGGGGAGTTCGTGCGGCGGCACGGGCTCCACCTCGTCGGCACCGCGTCCACGTGGCTCCTCCTGGACATCGCCTACTACTCGCAGAACCTGTTCCAGAAGGACATCTTCAGCGCCATCGGGTGGATCCCGCCGGCGCCGACGATGAGCGCGCTGGACGAGCTGTTCCACATCGCACGGGCCCAGATCCTGATCGCGCTCTGCGGCACCGTGCCGGGCTACTGGTTCACCGTCGCCTTCATCGACTCCGTCGGCCGGTTCAAGATCCAGCTCATGGGCTTCTTCATGATGACCACCTTCATGGTGGGCCTGGCCGTGCCCTACGACTACTGGACGGGCCAGGGCCACCAGGCCGGCTTCGTCGTCATGTACGCGCTCACCTTCTTCTTCGCCAACTTCGGGCCCAACGCCACCACCTTCATCGTCCCCGCAGAGATCTACCCCGCGAGGCTCCGCGCGACGTGCCACGGCATATCGGCGGCGTCGGGGAAGGTGGGCGCCATCATTGGGTCGTTCGGGTTCTTGTACCTTGCCCAGAGCCCCGACCCGGCCAAGACCGCCCATGGATACCACCCCGGCATCGGCGTGCGCTACTCGCTCCTCGTGCTCGCTGGCTGCAGCTTGATGGGGTTCCTGCTCACCTTCCTCGTTCCCGAGCCCAAGGGCAAGTCATTGGAGGAGATGTCGCGTGAGACCGAGCCCGACCATTGCTAActgtctactcacatatgactgtcggtgaacactgcatttagttttggcaattaggagatggcgacacggtgaaataatcgtcagaattattttaaggagtagaggttcttctttctctgtatcgtagtatggttgaatagaataaataacattttattttacctgtcattgaggtttgggggatttttgtccacacccccagcaacactaaaatttagaacgactatttcaatgtgtttcttcacgagtttatttctcagagagctcgttgtgtgttatacaatactacaattatggggatatgatttaagcacatcaaggataaaataaacatccccgcaaaaaaaaggataaaataaacatgcaaaccaggtgagctgccctcgaaaccatatacgct

**TaPht1;8 Chr6AL**

TRIAE_CS42_6AL_TGACv1_473139_AA1528910/ IWGSC WGA v0.4 chromosome 6A scaffold 11767

**Partial RT-PCR fragment HG764742 (bold)**

cacctgcagcgcaaccgccgacaacaaccgtagccacctttgtcgccgcagagccgcccgcgcgagcacctgccaggcgtcgccaccccgctcccggatgccacgccgccatgcccctgcacacgccgtagatacaaatgggatgaagatggaacttgatgcgtatgatcacgagcgtggaagggggagctttggaaacttatatgatatgaacccagttatatgcaagaggcttgcaattgtttctttagctaagaagctaccaaataaacagaacgaatatccggagaagaacaagaaggtggctgctatggtagtaccttttgaagatgtcgataatgatgataaagaagttgatggtgatggtacaccctagaagtcatttcgcaataagaagtgaaagattggtggtggggacaatgatataagtcaaaacaatgagatgtcagggagcaataaaaaaaattaaaattggaactgccaaaataatgagatatcagagagcaataaaaaattaaaaatggaaccgtcacgggttgagctctgcccgagcagttcgtgcgcttcaggtacataagcaaatacagtcggatgtaactttcctgttcgagacacatttgtcgaaagctaaggtggggaaaattccgagttataaagggttccatcacatgctcatttatgagagtgatttgaaaagtggcggtcctttgatgttgtagtggaatcatgtgaaaataaccgactttgctgaggcccatctgagaattcaagggaagaacataaagaaatcatgtggacctacatgcgttgttcaacatcccatggatggtcctagggaattcaacgaattttgattaaatagtgaaaaggaaggggggcaattcaagacatctcaggttcacggaagcttttcaagattgcttgggtcggtgtggcctaatggatatgtactatattggtgataggtttacttagagaagaggaaagataagagagaggcttgatagagccatcgacaatgtggaatgtgccgttgatcttatgatctaaaagtagtcatggaagcttgtagataggagggttgtatccgaagggatctcggcgacgatgatgatgacacggcggttttatgtaggtttaggcccatttatgttgctttgtgattcatgctcatgggtagagtacgtggttgatgatgttgcattcgttttcaatcgattttctagccgggttttcttctgcagttgggccatggtcgagtactagctaaacaacatttttttacctaaaaagaaagaagctagcatattcgcgggacgcgcgggattctcaccaatcatgtacacgaaagctgtgagccaacccatttggtcggttatattaattcggacacaaagggctgggttgaccgagtctgttttttttaaactagctaaaaaatgatgtagaatttcaaaaaactacggaagaacaccggctagaaaatcgactgaaaacacatgcaataccagccaaccaggtgctctacccacgagcacaaatcacaaagtaataaaatggacgataccagacttaactgaaagctaaagagataagttgcctcacaaatcaaaccggttagtcacacctgcattcaaaaagacactatcatccccatgccgtatgttgcgcattgcgccggccctacgatgaacccttcgctgcacaccggcgccttggagtttccaccccctttgttccttctccccgcattcagcaagttcattgtcttgtacatgcacatgctacaagatagccacatgcagctataccaccctcgtatcttgctattgttcccatttggcacatctagacaaagcaaagcagtaagccggcttttataccccccttgctcgcctatgacttggctcacattccctgtacacacacgatcgagggccgtcttgaatcttgctttgcaccggccaacaagtgtggacgggggagacggcgcgcgggccATGGCACGGCAGCAGCTGCAGGTGCTTCACGCGCTGGACGTGGCGAGGACGCAGAGGTACCACGCGTGGGCGGTGGTGATCGCCGGCATGGGCTTCTTCGCCGACGCCTACGACATCTTCTGCATCACCCTGGTCACCAAGCTCCTCGGACGCATCTATTACCAAGTCCCGGGCCAACGAGAGCCCGGGATGCTCCCCCGCCGGATCGAGGCGGCCATCAACGGCGTCACCTTCTGCGGCATGATCGTGGGCCAGCTCTTGTTTGGCTGGCTCGGCGACAAGGTCGGCCGGAAGAAGTTCTACGGCAAGACCATCATGCTCATGATCATGGGCTCCTTCCTCTCCGGCTTGTCGTTCGGGAACACGGCCGACGGCGTCATGGCCACGCTCTGCTTCTTCCGGTTCTGGCTCGGCGTCGGCATCGGCGGAGACTATCCGCTCTCCGCGACCATCATTTCCGAGTACTCCAACAAGAGGACGCGCGGGAGCCTCATCGCGGCCGTGTTTGCCATGGAAGGGTTTGGCATCCTCGCAGGTTGCATTGTCACCTTGGTCGTCTCCGCCACGTTCCAGGCGCGCTTCGACCCGCCGACGTACGCGGAAGACCCCATGGCCTCGGTCCCGCCGCAGGCTGACTACGTGTGGCGCATCATCCTCATGGTGGGCGCCATCCCAGCCGTCTTCACCTACCGCTGGAGGGTGATGATGCCGGAGACGGCGCGCTATACGGCGCTGGTCGCCCGCGACGCCGAGAAGGCCGCGCGCGACATGTCCAAGGTGCTCAAGGTGGAGTTCAGCGGCGAGCAGGACAAGATCGAGGGCTTCACCAAGGACAGGGACTACGGCGTCTTCTCCCGCCGTTTCGCCCGCCGCCACGGCTGGCATCTCGTCGGCGCCGTTGCGTCCTGGTTCGTGCTCGACATCGTCTTCTACTCCCAGATCATTCTCCAGGAGGAGATCTTCAGGGACATCAAGTGGATCCCCGAGGCAAACAGCATGAGCGCGCTCGAGGAAGCGTACCGCGTCGCCCGTGCACAGGCGATCATCGCGCTCTGCGGCACACTACCTGGCTACTGGTTCACCATCGCCTTTGTGGACGTCGTCGGGCGGAAGGCCATCCAGTTCCTCGGCTTCACCAT**GATGAAGGGTCTCATGCTCGTCGTCGCCGGCTTCTACCACCAACTGACGCAGCCTGGCCGGCGAATCTGGCTGGTGGTCATGTACGCCTTCACCTTCTTCTTTGCCAACTTCGGGCCCAACAGCACCACCTTCATCATACCGGCCGAGATTTTTCCGGCGCACGTCCGGACGACCTGCCACGGGATATCGTCAGCGGCAGGCAAGGTCGGCGCCATTGTCGGGACGTTTGGCTTCCTGTACGCCTCGCAGAGGGCGGACGGCAGCAATGAGACGGAGACCGGGTACCCGTCGGGCATCGGCGTGCGTGCCTCACTGTTCGTGCTGGCCGCGTGCAATGTGTTGGGAATAATTTTCACCTGTCTCCTGCCTGAGCCGAACGGGAGGTCACTGGAGGAGGTGTCCGGCGAGCCCATCAACGGGGAGGACGCAGATTTGGGTGATTCCAAGGTTCTTCCCTTGTAGaacctgcctgcacgtaggctgtgtgcaccact**cgatgaaaaagaagctgtttgaatgtgttttgtgctgggaacccgggattgggattagatgcattccaaggaccctaggtacactgcgtttgacaattatcgcggaggggaaatgtgaattaagaggaaggaattgtcgagattagacttacgatttctcattttactagtaagcatgcgtgtgcaacttatgttttgatcgacacacatagtttttttaggacaaatacacatggtttagaaccaattcaattggacatatcacaagtatgcaagccaaatgtgcacacatcaatactattctagtgtctcttctttctcctgatgcttgatattgtttctcctctttgatattgatcacaccacat

**TaPht1;8 Chr6BL**

TRIAE_CS42_6BL_TGACv1_499360_AA1579490/ IWGSC WGA v0.4 chromosome 6B scaffold

tctttagctaaggagctaccaaataaacagaactaagattcagagaagaacaagaaggtgtctgctatggtgggactttttgaagatgtcgacaacgttgataaagaagttgatggtgatggtacaccctagaagtcatttcacaataagaagtgaaagattggtggtggggacgatgatataagtcaaaacaatgagatatcaggcagcaataaaaaaaactaaaattgaaactgtcaaaacaatgagatataagggtgcaacaaaaaaactaaaattagaactgtcatgagttgagctcagcccgagcagttcgtgcgctcctagaggtacataagcaaataaagtcggatgtaactttcctgcccgagacacatatgtcgaatgctaaggcaggaaaaattctgagtcatacatggttccatcacatgcttattgatgagagtgatgtgaaaagtggcggtcctttgatgttgtggtagaatcatgtgaaaataaccgagttaagtatgccaaatcaacatatagatgttatcgtggaagaaagagagaaatggaggctcagaatgtacacgctgaggcccaattgggaattcaagggaagaacatagagaaatcatgtggacctacatgcgttgttaaacatcccatggatggtcctagagacttcaacgaaatttgattaaatagtgaaaaggaaggggggggggggcaattcaagacatcccaagttcatggaagctcttcaagattgcttggactggtgtggcctaatggatatggactatattggtgataggcttacttagataagggggaagataagagagagccttgatgtggaatgtgtcgatgaataatgctccattgatcctatgatctaaaagtagtcatggaatctcgtagataggagggtggaatccaaagggatctcgtcgacgatgatgatgacacggtggttttatgcaagtttaggcccatttatgttgctttgcgattcatgctcatgggttgagtacgttgttgactgatgtttcgttcgttttcaatcgattttctgaccgggctttttctgtagttgggccatggccgagtgctagctaaacaacaacatttacctaataagaaagaccacttgataagagtggtaagctatatcaaaaacattgaacaatcctgcttcaaaaaaaaaacctaataagaaagaagtttatcaaaaaataaataaaataaaaaagaagctagcatatttgcgggatcgcgcgggattcccatcaatcacgctcccgagcgctgtgagccaacccatttggtcggttatataattctaaaaaaaaggtcggttatattaatcgggatacaaagggctgggttgaccgagtctgtttttttagaccagagctaaaaaaatgttctagaatttcaaaatagtacggaagaatttccatgcaaaaaaaaagtacggaagaacacctggctagaaaatcgactgaaaacacatgcaacaccagccaaccaggtgctctacccatgagcacgaatcacatagtaataaatggacggtaacagatttgactgaaagcttaaaaaaatcagttgcctgacaaatcaaaccggttagtcacccctgcattcaacaagacactatcatccccgtgctgtataccattgcgcattgcaccggccctacgatgaacccttcgccgcacaccggcgccttggagtttccacccactttgttccttctcccagcattcagcaagttcgttgtcttgtacatgcatatgctacaagatagccacatgcagctataccaccctcgtatcttgctattgttcccatttggcacatctagacaaagcaaagcaataagccggcttttatacccccttgctcgcctacggttatttggctcacattccctgtacacacacgatcgagggccgtcttgaatcttgctttgcaccggccaagaagtgtggacggggagacggcgggccATGGCACGGCAGCAGCTACAGGTGCTTCACGCGCTGGACGTGGCGAGGACGCAGAGGTACCACGCGTGGGCGGTGGTGATCGCCGGCATGGGCTTCTTCGCCGACGCCTACGACATCTTCTGCATCACCCTGGTCACCAAGCTCCTCGGACGCATCTATTACCACGTCCCGGGCCAGCAAGTGCCCGGGATGCTCCCCCGGCGGATCGAGGCGGCCATCAACGGCGTCACCTTCTGCGGCATGATCGTGGGCCAGCTCTTGTTTGGCTGGCTCGGCGACAAGGTCGGCCGGAAGATGTTCTACGGCAAGACCATCATGCTCATGATCATGGGCTCCTTCCTCTCCGGCTTGTCGTTCGGGAACACGGCCGACGGCGTCATGGCCACGCTATGCTTCTTCAGGTTCTGGCTCGGCGTCGGCATCGGCGGAGACTATCCGCTCTCCGCGACCATCATTTCCGAGTACTCCAACAAGAGGACGCGCGGGAGCCTCATCGCGGCCGTGTTTGCCATGGAAGGGTTTGGCATCCTTGCAGGTTGCATTGTCACCTTGGTCGTGTCGGCCACGTTCCAGGCGCGCTTCAACCCGCCGGCGTATGACGAAGACCACATGGCCTCGGTCCCGCCGCAGGCTGACTACGTGTGGCGCATCATCCTCATGGTGGGTGCCATCCCAGCCGTCTTCACCTATCGCTGGAGGGTGATGATGCCGGAGACGGCGCGCTATACGGCGCTGGTCGCGCGCGACGCCGAGAAGGCCGCGCGCGACATGTCCAAGGTGCTCAAGGTGGAACTCAGCGGCGAGCAGGACAAGATCGAGGGCTTCACCAAGGACAGGGACTACGGCGTCTTCTCCCGCCGTTTCGCCCGCCGCCACGGCTGGCATCTCGTCGGCGCCGTTGCATCCTGGTTCGTGCTCGACATCGTCTTCTACTCCCAGATCATTCTCCAGGAGGAGATCTTCAGGGACATCAAGTGGATCCCCGAGGCAAACAGCATGAGCGCGCTCGAGGAAGCGTACCGCGTTGCCCGTGCACAGGCGATTATCGCGCTATGCGGCACACTACCTGGCTACTGGTTCACCATCGCCTTTGTGGACGTCGTCGGGCGGAAGGCCATCCAGTTCCTCGGGTTCACCATGATGAAGGGACTCATGCTCGTCGTGGCCGGCTTGTACCACCAACTGACGCAGCCTGGCCGGCGAATCTGGCTGGTGGTCATGTACGCCTTCACCTTCTTCTTTGCCAACTTTGGGCCCAACAGCACCACCTTCATCATACCGGCCGAGGTTTTTCCGGCGCACGTCCGGACGACCTGCCACGGGATATCATCAGCGGCAGGCAAGGTCGGCGCCATTGTCGGGACGTTTGGCTTCCTGTACGCCTCGCAGAGGGCGGACGGCAGCAATGAGAGGGAGACCGGGTACCCGTCGGGCATCGGCGTGCGTGCCTCACTGTTCGTGCTGGCCGCGTGCAATGTGTTGGGAATAATTTTCACCTGTCTCCTGCCTGAGCCGAACGGGAGGTCGCTGGAGGAGGTGTCCGGCGAGCCCATCAACGGGGAGGACGCAGATCTGGGTGATTCCAAGGTTCTTCCCTTGTAGaacctgcctgaacgtaggctgtgcgcaccactcgatgaaaaagaagctatttgaatgtgtgttgtgctgggaacccgggattagatgcactccaaggaccctacgtacacggcgttcggcaattatcgcggaggggaaatgtgaattaagaggaaggaattgtcgagattagacttaggatttctcatcttactagtatgcgtgtgcgtgcaacccacgttttgatggatacacatattttttttagggtacatggtccagaaccaatccaattggatataacacaagtatgcaacccaacactactaggaaagaccttatatatggatgattatcagtagcgctggttaaaaagagacgctactgatacttagcagtagcgcatggttatcacgagcgc

**TaPht1;8 Chr6DL**

TRIAE_CS42_6DL_TGACv1_528876_AA1716670/ IWGSC WGA v0.4 chromosome 6D scaffold 44363

agaggtacataagcaaatatagtcggatgtaactttcttgtctgagacacatatgttgaaagctaaggcgggaaaaattccgagtcatacagggttccatcacatgcttattgatgagagtgatgtgaaaagtggcgatcctttgatgttgtggtggaatcatgtgaaaataaccgagttaagtatgccaaatcaacatatagatgttatcgtggaagaaagagagaaatggaggctcatggtgtatacgctgaggcccaattaggaattcaagggaagaacacagagaaatcaatgaacctacatgcattgttcaacatcccatggatggtcctagggacttcaacaaaatttgattaaatagagaaaaggaggggggggggggggggcaattaagacatcccaagttcacggaagctttccaagattgcttggatcggtgtggcctaatgtatatggactatattgatgataggtttacttggagaagaggaaagataaaagagaggcttgatagagtcatcggtgacggtgttctggactagggggtactcatcacgtcgtctctcgaccaggtggatcgggccgaggacccccatggcggttgactgatgggccacttcgggcaacccatgacgcatacaaggaggattccacaagacttggcgatcaagacgaggactcctctccgccaacgtattcggctaggactcttattatcctaggcctatggtatattatataagccgaggccaggctagtcgatagagatacgctagatcatcagaatcatacacaacatacgatctcgaggtagatcaactctgtactcgatacgtcatcatcaatataaagaatatcaggacgtagtgttttacctccatcaacagggcccgaacttgggtaaacaccgtctccttcgtctcctattccccatcgatctaaggtccacagctcgggacccagtaccccgaggtctgccggttttgacaccgacgatcggtaatgtggaatgtgtcgatgaataatgctccgtcgatcttatgatctaaaagtagtcatggaagcatgtagataggagggtggtatccaacaggatctcgtcgacgatgatgatgacacggtggttttatgcaggtttaggcccatttatgttgctttgtgattcatgctcatggtagagtacctggttgactgatgttgctttcgttttcaatcgattttctagccgggttttcttgtgcagttgggccatggtcgagtactagctaaacaacattacctaaaaagaaagaagctagcatatttgcgggacgcgggggattctcaccaatcatgcacccgagcgccgtgagccaacccatttggtcggttatattaaccccgatacaaagggctgggttgaccgagtctgtttaaaaaaaaaaactagcaaaaaaatgttctagaatttgaaaaaactagggaagaacacctgtctagaaaatcgactgaaaacacatgcaacaccaaccaaccaggtgctctacgcatgagcacgaatcacaaagtaataaaatggacggtaccagatttaacttgaaagctaaaaagataaagttgcctcacaaataaaaccggttggtcacccctgcattcaacaagacactatcatcccgtgccgtatgttgcgcattgcgccggccctacgatgaacccttcgctgcatactggcgccttggagtttccaccccctttgttcgttctcccagcattcagcaagttcattgtcttgtacatgcacatgctacaagatagccacatgcagccataccaccctcgtatcttgctattgtttccatttggcacatctagacgaagcaaagcaataagccggcttatataacccccgtgctcgcctatggttacttggctcacattccctgtacacacacgatcgagggccgtcctgaatcctgttttgcaccggccaagaagtgtggacgtggagacggccggcgATGGCACGGCAGCAGCTGCAGGTGCTTCACGCGCTGGACGTGGCGAGGACGCAGAGGTACCACGCGTGGGCGGTGGTGATCGCCGGCATGGGCTTCTTCGCCGACGCCTACGACATCTTCTGCATCACCCTAGTCACCAAGCTCCTCGGACGCATCTATTACCAAGTCCCGGGCCAACGAGAGCCCGGGATGCTCCCCCGGCGGATCGAGGCGGCCATCAACGGCGTCACCTTCTGCGGCATGATCGTGGGCCAGCTCTTGTTTGGCTGGCTCGGCGACAAGGTCGGCCGGAAGATGTTCTACGGCAAGACCATCATGCTCATGATCATGGGCTCCTTCCTCTCCGGCTTGTCGTTCGGGAACACGGCCGACGGCGTTATGGCCACGCTCTGCTTCTTCCGCTTCTGGCTCGGCGTCGGCATCGGCGGAGACTATCCGCTCTCCGCGACCATCATTTCCGAGTACTCCAACAAGAGGACGCGCGGGAGCCTCATCGCGGCCGTGTTTGCCATGGAAGGGTTTGGCATCCTTGCAGGTTGCATTGTCACCTTGGTCGTCTCCGCCACGTTCCAGGCGCGCTTCAACCCTCCGGCGTACGCAGAAGACCACATGGCCTCGGTCCCGCCGCAGGCTGACTACGTGTGGCGCATCATCCTCATGGTGGGCGCCATCCCAGCCGTCTTCACCTACCGCTGGAGGGTGATGATGCCGGAGACGGCGCGCTATACGGCGCTGGTCGCGCGCGACGCCGAGAAGGCCGCGCGCGACATGTCCAAGGTGCTCAAGGTGGAATTCAGCGGCGAGCAGGACAAGATCGAGGGCTTCACCAAGGACAGGGACTACGGCGTCTTCTCCCGCCGTTTCGCCCGCCGCCACGGCTGGCATCTCGTCGGCGCCGTTGCGTCCTGGTTCGTGCTCGACATCGTCTTCTACTCCCAGATCATTCTCCAGGAGGAGATCTTCAGGGACATCAAGTGGATCCCCGAGGCAAACAGCATGAGCGCGCTCGAGGAAGCGTACCGCGTCGCCCGTGCGCAGGCGATCATCGCGCTCTGCGGCACACTACCTGGCTACTGGTTCACCATCGCCTTTGTGGACGTCGTCGGGCGGAAGGCCATCCAGTTCCTCGGCTTCACCATGATGAAGGGTCTCATGCTCGTCGTGGCCGGCTTCTACCACCAACTGACGCAGCCTGGCCGGCGAATCTGGCTGGTGGTCATGTACGCCTTCACCTTCTTCTTTGCCAACTTCGGGCCCAACAGCATCACCTTCATCATACCGGCCGAGATTTTTCCGGCGCACGTCCGGACGACTTGCCACGGGATATCATCAGCGGCAGGCAAGGTCGGCGCCATTGTCGGGACGTTTGGCTTCCTGTACGCCTCGCAGAGGGCGGACGGCAGCAATGAGACGGAGACCGGGTACCCGTCGGGTATCGGCGTGCGTGCCTCACTGTTCGTGCTGGCCGCGTGCAATGTGTTGGGAATAATTTTCACCTGTCTCCTGCCTGAGCCGAACGGGAGGTCGCTGGAGGAGGTGTCCGGCGAGCCCATCAACGGAGAGGACGCAGATTTGGGTGATTCCAAGGTTCTTCCCTTGTAGaacctgcgtgaacgtaggctgtgcgcaccactccatgaaaaagagtcattttacagcgcatccggacgatatggaccgtccgtcggagcgaattcgacggtccagatccgatgcaatacgctgatgtcaatcagaccccgaggtgcattttcgggagaaaaaaatatttcgaactgatctgattttccctcctaaatatttcgagggtattatcggtacgaatttcagggctattttcggttcgatatttatcccgttcgctagggtttattcctcgccgccgctacggttttaatcctcgccggcaggatctagccgctcgtcgcggcccctccctccctcgttgccggcaggatctagccgcgcgtcgcggcccctcccttcctccaccccaattccctcgcgatctcgcaccaccttttcaacgaacac

**TaPht1;9 Chr2AS**

TRIAE_CS42_2AS_TGACv1_112554_AA0340800/ IWGSC WGA v0.4 chromosome 2A scaffold 909 Chr2A

ggcgccggcccctcgcgagcgcggcgcccccgcccggccagatcgtcccgccccaaccccaggctgagcaaagggggggaaagcttccggccgccgccggtgccaccagggctttaccccgcggctctagccggcggcagcgggggagggagacgggtgggggaagagggaggccggcggctagggttggctcgctcggccgcccgcggggccgacacgagggggagaggggaaaggggatgaaagaatcagaccagcttccaacttcaccgggctgcactaccgcacaacaggaggtgttctcgctcgcagctggtctccgtgggccgcagtcaccatcgcttgcaagttgcagtcaactacattggtaatatcagtctaacggaactggaatctgcaccagattccagaggatataccctaagacgcgtgtgatctcaacatgtcgcgtcagtgtgtcacagaccctctaatatattctcaagacgcaccaatcatttcttgcatggatgaacacaaaaataattcttattatagttggttcctgccgtccaagctagacttcgtggttcgtacgcacgaagaccggcggcagattcctgccaagtttgtcgctcacacaattaataaagatccagtacgaaattttcagtagatccacaggttaaatagtattccagtgacatattttttaccacaatgtcaagaatatattaaccattagtctttcagtttcatgagttcttttagattttctatttcatgagtttgcttcagatatcgcatggagatttcggccgtttctcaaatgttcattcctctaaacttagtggccatctggacgatgcagctacacatccaacaaggtcttggatcttgcagttaaccttatgctagtgtcttggtgcacaatcatattctaacatcttggcaacgcatgcagggcaacatatgtcccttaggagacaacaatgaactgtcttaggaaattaagcctaattattagtaacaaaacaataataatatttgaacaaaaccttaactgcgtgaaagcaacgtacaatttcatatgggactctaggggccactctacgcacatatacatatctgtctgcaggattatcactgcaacaacaacaaacggcaggaaaatagcaaggaaccATGGCTCGCAACCAGCAACTGAGGGTGCTGCAGGCACTGGACGTGGCTAGGACGCAGTTGTACCACTTCATGGCGATCGGCATCGCGGGCATGGGCTTCTTCACCGACGCCTACGACCTCTTCTCCCTCTCCCTCGTCGTCGACCTCATCGGCCACAGGTACTACACAGGCGGCCCGATACCAACCTACATCTCCGTCTACATCAACGCGGCCGCCCTCTGCGGCACTGTGCCTGGGCAGCTCGTCTTCGGCTGGCTTGGCGACAAGATGGGCCGGAAGCGCATCTATGGCGTCACTCTACTCCTCATGGTCTTCTGCTCCCTCGCCTCCGGCTTCACCTTCGGCAAGAGCAATTACAAAAGCGTCGTGATCACGCTCTGCTTCTTCCGCTTCTGGCTCGGCGTAAGCATCGGCGGCGACTACCCACTCTCGGCCACCATTATGTCTGAGTATGCTAACAAGAGGACCCGCGGTGCCTTCATGTCTGCCGTGTTCGCCATGCAA*gtaaggatgacatggcttgattatctagttctttagacaaaggaagacgattattgcctccctgactgttttaatttatatacatagcaattgcaatataagcacactttatttccaactaattccag*GGTTTCGGAAATGTTGTGGCGGGGCTCGTTGGCATGATCACATCACAAGTCTTCAGGAAATCATCGCCAGGCAATATAGATGTTGTCTGGCGCATTGTTCTCATGTTTGGCGCCGTTCCTGCACTGCTCACCTACTACTGGCGCATGAAGATGCCAGAGACGGCACGGTACACGGCCCTCATAGCCAAGGACGCAAACATGGCTGCAGCGGACATGTCCGCAGTCCTCAGCATGCACATCGTCCCAGAAGAAGACAATCTCGGCAGCCACGACCAGTATGGCCTCTTCTCCTCGGAGTTTCGCCAACGTCATGGGCTCCATCTCCTCAGTACCACCGTCTGCTGGTTCGTCCTTGACGTCACCTTCTACTCCCTCAACCTCTGTATGAAGGACATCTTTACTACAGTCGGGCTGCTTAAAGGTCATGATGATGAAGTCTGTAAGGTTAATACTTGTCAAGTGGCTAGGTATCATAAAGCTGACAATCCGTTCCAGCGAACAATCGACATCACTGCAGTGCACATGGCCATCGCAGCGGGGTGTACATTGCCAGGATACTTCTTCGCGGTCGCCTTCATTGATCGCATAGGCCGCGTGAAAATCCAATTGCTCGGCTTCACCATGATGACGGTCTTTCAACTCTGCCTTGCCATCCCCTATGATAAATGGCTAGAATGCAACAACAACAAATATGGATTCGCCGTCATGTATGGCTTCATCTTCTTCTTCGCGAACTTTGGGCCAAACACTACAACTTTTATCTTTCCAGCGGAACTCTTCCCGGCAAGGTTGCGCTCCACATGCCATGGTATATCGGGGGCGGTCGGGAAGATCGGTGCCATCTTCGGCGTGTTAGCCTTCTCCTTCTTGAGGTGCCGTTTCAAGATCTTGTTGTTTGTCCTTGTTGGTTGCAATCTAGTTGGCATTGTGTTCACTCTTCTATTGCCGGAAACTAAGGGAAAATCTCTTGAGGAGATTACTGGGGAGATAGAAGAAGGTCAGCCTCTGGATGATGCAGCAGCAGTAGTCAATGCAGCTGACGGCATACACGTTGTGCCTGTTTAATTAGcctttttgatttgactgattggtctagatgtgtgttcttttaaaaggttgcctgggggaatggatagtatatgaatgtgtatatttcacattaattatcttgattattgcctagttataaagaatgtaaatcaaattcgagattaataattgaaaatggggatcaacgttttccatgtcgggggataacccatggttttttctagaagtaagagcatctaaagccgaactttaaaagtctggcccgacaaacgccagcggacgcgtctgcgggcactgaccgggcgcttctcaaaaaatacaatccatatccggacaactcaattatcatatctcaaatccatacaaactcatgcagctacgtcgatgcacacgcatcgtccggctactcaggggcgggc

**TaPht1;9 Chr2BS**

TRIAE_CS42_2BS_TGACv1_145945_AA0450360/ IWGSC WGA v0.4 chromosome 2B scaffold 21713

ggcataggcgcactttaacgcctccatggctgaggataagcccacacccgtgcctattttgctgttatggcaagcacaggaggagcagtggtacaacctgatactcctcgagcagcacctgccaacggagggccagatctacaatgagcgaacaaggaagccgtggcggccatggccgcggcgaaccacaacttcatcgccgaggcgctctacgaggccgcccgcgctcaagccgccgttgcaacgcacaagctacacagctcggcgtggaggcggagctagctgcacaggcgagtgtggacgagaacgccgccagcggtgcatcctacgcgcccccaaacactggttgaccctgccgatgggacaatgacggtgtcggcccctccatttccatcgtggacctcacgcccaccggcgacggacaggtgcagactccgccgagaatgagtacggcttgggaggcggcagggccttgtgttacaagtgggccggtctgtctttcatattctactcttcctcgctggcgatatagcaaacacatcgcactcttcggcatccttggtccttcgcctcaactcgaccatctacctcctgctccggcactggcgacttcattactccggcgagcggcggtgcctttgtcaacagaaacaccatcttttaacttcaaaactactactccctctattagtattaaactccaaaaaggtcgtatgtttaggaagcttccagctcaatgcacactagtttccaacttcactgggctgcactaccgtacaacaggaggtgttctcggtcgcagctggtctccgtggtccgcagtcaccatcgcttgcaagttgcagtcaactacataggtaatatcagtctaacggaaccggaatctgcaccagattccagaggatataccctaagacgcgtgtgacctcaaacatgtcgcgtcagtgtgttacacaccctctaatatattctccagttgcaccaatcatttcatccatggatgaacagaaaaataataattattactagttggttcctgccgtccaagctagacttcgtggttcgtacgtacgaagttcgaccggccgcagattcctgccaacttcgtcgctcacatatattataaagatcaagtacgtaattttcagtagatccactggttaaatagtactagaggataccccacacgttggtccgagaattgattgatgaaatttttggttccagcatgcaaactaaaattaaaaatacatatgacttattatatttgattatgtataattgtaaaatatttattgaatataagtagaataactgaatggaaaacattttcccatgtatggttgaatgttgtggtggtttttttcacatgcaagattgcatgttgaggtgggccttatcccatttataattatatgatgaggtgacatgcttgcatgttgagataaatgagttagtgaggatgaccttcttagatatataggattccagtgacatattattttccacaatgtcaagaatacattcaccattagtctttcagtttcatgagttcttttagattttctgtttcatgagtttgcataagatttcggccgtttctcaaatatttattcctctaaactttgtggccatctggacgatgcagctacacatccaacatggtctcggatttttgcagttaaccttatgctagtgtcttggtgcacaatcatattctaacatcttggcaacacatgcagagcaacatatgtccctttggagacaaccatgaactgtcttataggaaataaactaagtagtaagaccatgaactgtcttagggaaacaaactaattattagtagcaccacaacactaataattgaactaaaccttaactgcgtgatagcaacgtaccatttcatgtgggactgggccattgtatataggagtagatatctaccgggaagaatatcactgcaacaacaccgaacggcaggagagtagcaaggaaccactATGGCTCGCAACCAGCAGCTGAGGGTGCTGCAGGCACTGGACGTGGCGAGGACGCAGCTGTACCACTTCATGGCGATCGTGATCGCCGGCATGGGCTTCTTCACCGACGCCTACGACCTCTTCACCATCTCCCTTGTCGCCGACCTCATCGACCACAGGTACAACCCAGATGCCCGGAGAGGAAACTTTATCCCCGTCCTCATCAACGCCGTCTCCCTCTGCGGCACTGTGCCTGGGCAGCTCGTCTTCGGCTGGCTCGGCGACAAGATGGGCCGGAAGCGCATATATGGCGTCACTCTGCTCCTCATGGTCTTCTGCTCCCTCGCCTCCGGCTTCACCTTCGGCAAGAGCACTAACAAAAGCGTCCTGATCACACTCTGCTTCTTCCGCTTCTGGCTCGGCTTCAGCATCGGCGGCGACTACCCGCTATCGGCCACCATCATGTCTGAGTATGCTAACAAGAGGACACGCGGTGCCTTCATGGCCGCTGTTTTCGCGATGCAAgtaagaatcttgtatatttttagatttataaaggaagaggattatggcctccctgcctcacttgacttacatacgtgtatgtgaattctacattcaccaaactttatttcaaactaattctagGGTTTCGGAAACGTTGCGGCGGGGCTCGTTGGCACAATCACATCAGCAGCCTTCGTGAACTCATCGCCAGACAAAATTGATTATGTCTGGCGCATTGTTCTCATGTTTGGTGCCGTTCCTGCTCTCCTCACCTACTACTGGCGCATGAAGATGCCCGAGACAGCACGGTACACCGCCCTCATTGCCAAGGATGCAAAGATGGCCGCGTCAGACATGTCTGCTGTCCTCAACATGCACATCGTTCCCGAAGAAGACGGCGTCAACGAGCTCGCCAGCCAGGACCAATATGGCCTCTTCTCCTCGGAGTTCCTCCGCCGCCACGGGCTCCACCTCCTCAGCACCACCGTCTGCTGGTTCGTCCTTGACGTCACCTTCTACTCCCTCAACATGTTTATGAAGGACATCCTTACTAAAGTCAGGCTGCTTGACATTGACGATGACCTTCGTAAGGTATATTGTCAAATGGCTAGCCCACCTGTGGCGCCAGGGTATCATAAATCCGTCAATCAGTTCAAGCGAACGATGCGCATCACTGGAGTGCACACGGCCATCGCAGCGGCATGTACATTGCCAGGGTACTTCTTCGCGGTGGCCTTCATTGACCGCATAGGACGCGTGAAAATCCAATTGCTCGGCTTCACCATGATGACGGTCTTTCAACTTTGCCTTGCCATCCCTTATCCTAAATGGCTAGAATGCAACAGAAACAAATATGGATTCGCCGTCCTGTATGGCTTCACCTTCTTCTTCGCCAACTTTGGGCCAAATACTACGACTTTCATCCTTCCAGCAGAACTCTTCCCGGCACGGTTGCGCTCCACATGCCATGGTATATCAGGGGCGGTCGGGAAGATCGGCGCCGTCGTCGGCGTGTTTGCCTTCCACTTCTTGAGGAACCAATTCAGGGCCTTGCTGTTTGTCCTTGTTGGTTGCAATCTAGTTGGCATTATGTTCACTCTTCTATTGCCGGAAACCATGGGTAAATCTCTTGAGGAGATTACTGGAGAGACAGAAGAAGGTCAGACTCTGGATGATGGAGCTACAACGGTCAACGCAGACGACGGCATACACGTTGTGCCTGTCTAAttaaccttgttgatttcattgatcaatctagatgtgtcttgtactcaataggttgccttggggatggtacatgaaagtgtatatttcatcctaattatcttgattattgtgtataagaatataaattggaatttgagctttataactgaaactggggatcaacgattttcatgtcagggaacaacctatggtttttttctagaagtaatagccataaatctcgacacgagatgaatctggaggtgtcggacgagcgaacgataatgcccgactgtactgtggatcactgttcaagaaatcttccgattcagcgattaatctttcgatttgtctctactcatggggtgaccgataagattatgccttatcttcaccgtttatcttttggaccaatatatcg

**TaPht1;9 Chr2DS**

TRIAE_CS42_2DS_TGACv1_179047_AA0603850/ IWGSC WGA v0.4 chromosome 2D scaffold 42730

agcactcgaccacactagtaagagtgaaagggatcacctttcactcctagcccggcatcagccatcgggtttggggaggcacctccgcagtaggaagcgcccacgcctgtatccccatccggtcccggtggaccagggggagacaacctgttgattaccgacggccaccgttgagcacgctcgaacgacgccatgtctgtggtcaatggtgctgatctgtacgacgacatggcgacgcccgacgagcaatctcggatcgacgccaccaacgcgcaaaaaggaagaagagcactaatgaataagcttgcaccccgtgcggaccaagtcggctccctacaactacgcgccatcgaaccacatccaccaccgcataccggatccgcaccgtcatgacaagccgaagagacaaatcgccagcaccgacctcctgaagcatggaccaccactgccaaagagcagcagggtgccccgcagccgcccgagggatcccaagcagcaccacctcgccggaactacgccaccctgccagatcagccccagatccaggccgccggcccgagtccgctgcccaccatgccgcccgcccgaagagcgagctgccgcaccccgtagcgccccaaactgtgccgccacgacgcccgcagaccgccaggccgccagattgggctacccgatggtgcccgcatccgccagagtgccgccgcgcgaagccgccgcacagcctgctcgcgcacttggccccgccgcctgcgcggccgcgcacgccgtcaccacccacgtctgctgccacgccgccggcccctcgcgagcacggcgccaccgcccggccagatggtcccgccccaacccctgtctgagcaaaggggggaaaagcttccggccgccgccagggctttgcctcgcggcgctagccggcggcggcgggggagggagacaagacggggaagagggaggccggcggctagggttggctcgctcggccgaccgcggggccgacgcgagggggagaggggaaaggggatgaaagaatcagaccagctacctacttcaccgggctgcactacctcacaacaggaggtgttctcgctcgcagctggtctccgtggtccgcagtcaccatcgcttgcaagttgcagtcaactacattggtaatatcagtctaacggaaccggaatctgcaccagattccggaggatataccctaagacgcgtgtgatctcaacatgtcgcgtcagcgtgtcacagaccctctaatatattctcaagacgcaccaatcatttcttgcatggatgaacacaaaaaaattaattattactagttggttcctgccgtccaagctagacttcgtggttcgtacgtacgaagaccggcggcagattcctgccaacttcgtcgctcacatactccacaggttaaatagtattccacaggttaaataaagatcaagtacgtaattttcactagatccacaggttaaatagtattccagtgacatatttttttccacaatgtcaagaatacattcaccattagtctttcagttccatgagttcttttagatttttagtttcatgagtttgcatcagatagcgcatggagatttcggccgtttctcaaatatttattcctctaaaccttgtggccatctggacgatgcagctacacatccaacatggtctcggatctttgcagttaaccttatgctagtgtcttggtgcacaatcatattctaacatcttggcaacgcatgcagggcaacatatgtcccttaggagacaaccatgaactgtcttaggaaaataaactaagtagtaagaccatgaactctcttaggaaaacaaactaattattagtagcaccagaacactaataattgaactaaaccttaactgcgtgatagcaacgtaccatgtcatatatatatatatataggagtagatatctaccgggaagaatatcactgcaacaacaccgaacggcaggagagtagcaaggaaccATGGCTCGCAACCAGCAGCTGAGGGTGCTGCAGGCACTGGACGTGGCGAGGACGCAGCTGTACCACTTCATGGCGATCGTGATCGCCGGCATGGGCTTCTTCACCGACGCCTACGACCTCTTCACCATCTCCCTTGTCGCCGACCTCATCGACCACAGGTACTACCCAGATGGCCAGAGAGGAGACTACCTCCCCGTCCTCATCAACGCCGTCTCCCTCTGCGGCACTGTGCCTGGGCAGCTCGTCTTCGGCTGGCTCGGCGACAAGATGGGCCGGAAGCGCATATATGGCGTCACTCTGCTCCTCATGATCTTCTGCTCCCTCGCCTCCGGCTTCACCTTCGGCAAGAGCACTAACAAAAGCGTCGTGACCACACTCTGCTTCTTCCGCTTCTGGCTAGGCTTCAGTATCGGCGGCGACTACCCGCTGTCGGCCACCATCATGTCTGAGTATGCTAGCAAGAGGACCCGCGGTGCCTTCATGGCCGCCGTTTTCGCCATGCAA*gtaagaatctagtatatgtttagatgtataaaggaagaggattatggcctccctgtctgacctgacttacatacgtgtatatgagttctacattcaccaaactttatttccaactaattccag*GGTTTCGGAAACGTTGCTGCGGGGCTAGTTGGCACGATCACATCAGCAGCCTTCGTGAACTCATCGCAGGACAAAATTGATTATGTCTGGCGCATTGTTCTCATGTTTGGTGCCATTCCTGCACTCCTCACCTACTACTGGCGCATGAAGATGCCAGAGACGGCACGGTACACCGCCCTCATAGCCAAGGATGCAAAGATGGCCGCATCAGACATGTCTGCCGTCCTCCACATGCCCATCGTTCCTGAAGATGACGGCGTCAACGAGCTCGCCAGCCAGGACCAATATGGCCTCTTCTCCTCAGAGTTCCTCCGCCGTCACGGGCTCCACCTCCTTAGCACCGCCGTCTGCTGGTTCGTCCTTGACGTCACCTTCTACTCCCTCAACATGTTTATGAAGGACATCTTTACTAAAGTCAGGCTGCTTGACATTGACGATGACCTTCTTAAGGTATATTGTCAAATGGCTAGCCCACCTGTGGCGCCAGGATATCATAAATCCGACAATCCGTTCAAGCGAACGATACGCATCACTGGAGTGCACACGGCCATCGCAGCGGCATGTACATTGCCAGGGTACTTCTTCGCGGTGGCCTTCATTGACCGCATAGGACGCGTGAAAATCCAATTGCTCGGCTTCACCATGATGACGGTCTTTCAACTTTGCCTTGCCATCCCTTATCCTAAATGGCTAGAATGCAACAGAAACAAATATGGATTCGCCGTCCTGTATGGCTTCACCTTTTTCTTCGCCAACTTTGGGCCAAACACTACGACTTTCATCCTTCCAGCAGAACTCTTCCCGGCACGGTTGCGCTCCACATGCCATGGTATATCAGGTGCGGTCGGGAAGATCGGCGCCGTCGTCGGCGTGTTTGCCTTCCACTTGTTGAGGAACCAATTCAGGACCTTGCTGTTTGTCCTTGTTGGTTGCAATCTAGTTGGCATTATGTTCACTCTTCTATTGCCGGAAACCATGGGTAAATCTCTTGAGGAGATTACTGGAGAGACAGAAGAAGGTCAGACTCTGGATGATGAAGCTACAACGGTCAACGCAGACGACGGCATACACGTTGTGCCTGTTTAAttaactttgttgatttcattgatcaatctagatgtgtcttgtactcaataggttgccttggggatggtacatgaaagtgtattttcatcctaattatcttgattattgtgtataagaatataaattggaatttgatattaataactgaaactggggatcaacgattttcatgtctgggaacaaactatggtttttttctagaaggtgtcggacgagcgaacggtaatgcacgactgtactgtggataccaccaatgtgtcgctaaccatatctcaagtgtgcataatgagtgggtgtgtatgatgcttgccaaaaaccatggtcaccaactgccctacccaaagtcatgaagtgtttgaaatgctcttgtcgagcaagtattttctaatgtatcgatcct

**TaPht1;10 Chr7AS**

TRIAE_CS42_7AS_TGACv1_570336_AA1834060/ IWGSC WGA v0.4 chromosome 7A scaffold 21044

tcactcttagggccatcagcacttgtaactgtaagtttttgactccaaggcctccatagctcagagcttgcagattgttttccacgccaccatacactgcccttcattcacctcttcctttcctggctagaagaatgaacacatgcatccatttatgtcctcaaacaccaaagtcggtgcctccatgaccaacagatgatgaattggccctgctgctatgacaaatttaaccaagaccaatcaacttgatctctgcatcattccccttttccatgttggtatgaagtgtctcacttgatctagcattggttgccatcgcggcctagtcagttgcttgatagccagttgtaagccaaggtacttgcacaagaatgctttgatgggacgatgggacaattcagtaagctctccactcgggctcgatcttcctggttgtctgcaatcaaaatcgctaaagatttcatgtagttcacacgcaacccagtcacttccccgaacacctgtagggcatatctaacgaaccgaagatcattctcagttggccttaagaagaaggcggcgtcatcagcatagatagattggctctgagttgctgtgatccctctgtaattgctcagcactccttctttcagcgcccggtttattattgttgtgagggcattcatcgcaatgatgaacagtaacagtgagttggggtcaccttgacgtagcccttttgcatgccggatccgtcatcctggggctccattcactagcaccttagtgtttgcagtttgtagtaatgtgacaatccatcgtaactaggaatcgccaaaacctttagctctgaggatctcgaataagaacggccaggatagcgaatcgaacgtgcgtgagatgtccaattcaaaaagactccttgactttttttttgcatctggattttcttggccacttgacacaccagcaggtagttatcatgtaagctatatccctggataaacgtcgtttgattagttcccacaagcacatgcatgtgtcttcgggccctagtggcaagcatcttggccaccaattttgcaaagttgtgccgcaggcttatagtgcggaaatctccaactagtttttcatcaaccttcttcaggattaatacgatcaaagctctgttaagcctagcacaacctctgtcgtctcctacgtaaaacttcagcaaagctgccatgatattcttcttgatgatcggccatgctttctgatagaaaactcctataaatccatccgggcctgaagtacggtccgggtgtaactcttttatggtcgaccagtttgtagtttagtcaaaaccccttttatttattgaatattattgaaaaaaattaaaaaaaacttttattattgctttcaatagaatagactatttttaaactatgatttgtgtagtcatgtccatgacaacgtagctgcgggtttggtagttgtcagctcatcgctccctgtgggttcgacaaacatttatcaccaattacaatagtcatgtacactcgcaggtcactgtgcttttctgacgccgttgccgaggagaatggcacttggtttggaaaaaatgaatccagttttcgtttgacaatttcggacgaaaaaataacgaaaaaaatcccttcgactatttctcgattgagaaattagcagatcaatttattttttctgatggctccctaaaatgctcttggcccgccaatggagctggagagtcttagatagcgaggcagtggcatcttcctccgcatctcagttccacccggcacatactctccatgtcaacgcgcacggccgtttctttactccagcacgtagccatcaccggtacggccaccatctacctcgcgcctcataaaacaaagcaaaatcctaagttgcagtcagaccagtaccctccctaatcaaacctgaccgcaggttccaatcctttccccgcgggctctcgctcgatcgaccggtagctgccgcggcggcggtccggaATGGCGCCGATACGCGTGCTCACGGCGCTGGACCACGCCCGCACGCAGTACTACCACTTCAAGGCCATCATCATCGCCGGCATGGGCCTCTTCACCGACTCCTACGACCTCTTCTGCATCGTGCCCGTCATGAAGATCATCGGGCGCGTGTACTACCCTTCGCCCGCCGGCGACGGGAGGCCGGGCGTGACGCCGCCCGCCGTCGTGTCCGCCACGGTCGGCGTCGCCCTGCTGGGCGCGGTGGTCGGGAACCTCCTCTTCGGCGCGCTCGGCGACCGCGTCGGGCGGCGGCGCGTCTACGGCCCGTGCCTGCTGCTGCTGGTGTGCAGCTCCATCGGGAGCGGCTTCTCCATCTGCCGCACGCGCGGGTGCGTGCTCTCCAGCCTCTGCTTCTTCCGCTTCATCCTCGGGGTGGGCGTCGGGGGAGACTACCCGCTGTCAGCGACCATCATGTCGGAGTTCGCCAACAAGCGCACTCGGGGAGCGTTCATCGCCGCCGTGTTCTCCATGCAAGGGTTCGGGATACTGGCCAGCTCCGGCGTCACCATGGTCGTCGCCGCCGCGTTCGACCGGTTCACGGGCCACCCGGACCCGCTTGACACTCCGGAGGCCGCCGACCTCGCCTGGCGCGTCATACTCATGGCCGGCGCCGTCCCCGCCGGGCTCACCTTCTACTGGAGGATGGCCATGCCAGAAACAGCCAG*gtactacgtcggttgtcctaactccaactggtgcactacgtgtctcttcgaccacgtacgttgtatttccctaacgtagtactagtactctgtacgagtgacattcatacagggtgttatcgtacgtacgtatgtttcagtacgttgtcaataccttagagaaagctccaattttcatatgagtcaagccgtagtctcacgtttgggagtaactttgatcaaaatattgtctgtgatgaccaatgcccacctcacatgaaaacccggatttaagctgatacatctgaaaggggtaaaaaaaatgaacagtttaagagttctaggctgcgtttagttccattgcacagtcagtttagtttgcaggtgattttctcgtactgtgtgtccgtttctaagctgtgaacgaagtacctttgaacggctccatggtaagttaacaataccagctgttgctgccctgattgttaatccttgattatactatacttttttgagttggcatcctgggttatattcgagtaccatcgtaagacacacgagcgcgagttgcacaccttgcactaaacatttcagcaagtcatcgcattgattggtagactaaactaaactgcgatgatcgatgtgtacactgactggtatctcaaacaaatggcgtggagaagaatataccgtgggatacttgagatccgacgaagcagcagcttcctgctgaccaacgaagaagttcagtctgttccccgtaaacgaacctgggcgcagtttgcctaattattttctctgacccaaagcttaagtgtactgtcaggctctggccaccatcataaggttgctggttatactttctctgtttttatttagtctgcatattaggtttgaccgaagtcaaactttacaaaatttgatcaagtttacagaaaaatatataaacatttgccataacaaatctatatgatgtgaaagtacattcaataatgaatctagtggtattgattttttattgtgtatattaatatttttatttataaacttagtcaaaacttacaaagcttgactttgaccaaagctaatatggggactaaataaaaacggagggagtatttgtctagttttagtattagtatatcttatcgtcaagttgcctggctctgtttgaccttgcactatcttctagaacgagtcgtgcatctggctgtcatgtttaagcatacttgaacaagttgcataagcatacttaaacaggttgcatacgttgtaaaataagcaagttgagcatatctatatctggatcgatctgcgtctcgttggattggcctcgtcacaccacacgtcaggccggcatatcaaattgaagtggcatatactggcaatctggcattgaatctggttcgctttagttttccttttgcagttaacaaaccaggtcgatcaggaggtgattctttacaccaaaagctaaagtagtcacgtactactgtagtgtcaaaaaagaaagttaaagtagtaaccaactaaagttcactaattaactgagctaactactctatctgatgggtgctggtggtgcag*GTTTACGGCGCTGGTACAGCGCGACGTGCTCAAGGCGACCAGCGACATGGGCTGCGTCCTCACCGACCTCGACCTGAACGGGATATCCGAGGGGGAGGACGCGGCGGCCATGCCCCGCACCCCGTCGCCGTTCGGGCACGCGCCGACGGCCCAGTACGGCCTCTTCTCGCGCGGGTTCCTGCGGGAGCACGGCCGGGACCTGTTCGGGTGCGCGGCGACGTGGTTCCTGCTCGACATCCCCTACTACAGCAGCACCCTGTTCCAGTCGCAGATCTACCGGCCGTGGTTCCCGCCGGCGAGCCACCAGAACGTCTTCCAGGAGGCGTACAACGTGGCGAGGTTCCAGGCCATCATCGCCGTCGCATCCACCATCCCGGGGTACTTCGCCGCCGTCCTGCTCATCGACCGCACCGGCCGGCGCCGCCTGCAGATGGCCGGCTTCTTCCTCATGGCCGCGTTCCTCTTCGCGCTCGCCGGCCCCTACGACCACTACTGGCGCGGCAACGCCAAGAACGCCTGGTACATCGTGCTCTACGCGCTCACCTTCTTCTCCGCCAACCTCGGGCCCAACACCACCACCTTCATCCTGCCGGCCGAACTATTCCCGGCGCGGTTCCGGTCCACCTGCCACGGGATATCCGCCGCCGCCGGGAAGGTCGGCGCGCTCGTTGGCTCGGTGGGGTTCCTCTGGGCGTCGCAGTCGCGTGACAGGGGAGATGTGCAGGCCGGGTACGAGCCCGGCATCGGCATGATGTACGCGCTCGTCATTCTCGGAGCCATCAGCCTGCTCGGGCTCGTCGTCACCTACTTCTTCACGCCGGAGACGATGAGGCGGTCGCTGGAGGAGAACGAGAGCGAGCGGGACCAGAACCAGGACGGCGACGGCGGGATGTGCTTCCAGGAACTAACTCTGACGCCCAAGAGCCCGGGGTCCTTGGTGAGCTCGCACGTTAGCACGTCGCCTATCCATCCGCACCGCTTCTCTGTCTGACGGAACTTTGAaaacatagcacgacgcataagcagttccgtttttggctactggcacgtgaaatgcagatgtagataatcctaaaaagaagaaacacatatgtagatttccccgcatggttgttccgtttgattggttgagatgagacaaaattgcatagctcaaactttgtaaaggtctgtcgtatgtaactgggctatgttattatatgcaagttggtgacggtgattgtggtgtcacacaaaacacaaatatagtcgggtcggtatgagcatcagtgcgcaacatcaagcccatcccacttaaaaataccatatgggtaatattttcgaaaaagaacgctttattacttaaaaggtttgatttaagcattacacccaacctctgcataattatgatgcacacagccaa

**TaPht1;10 Chr7BS**

TRIAE_CS42_7BS_TGACv1_592271_AA1934790/ IWGSC WGA v0.4 chromosome 7B scaffold 109581

ctaaccctcgatgggggaggcaaacacgcatacctggctagtaacttattcgccgttcgaagaagagctggactggtcggtccaagaaaccatggcggagcggcgaatggcggcgacttgacagtggcgccgccggcggtggtcgcagtcgagagagaatagagcgagaggggtgaactggtctagtctggtccgaacagtgactggtgcaggccactcatacacggggcaggaacggccgtctgtcgccgtctaacggcgttcgtcaaccggacagacgggacccacctgtcggattcgctgttttccgagctaaaaccagcatcagtgcttcgcgttacagattaaccccattgttggtggttttttgtgtcctacctcaaatatagtacagatttgttaccctaacctcaagtgtgatggtttttggtaaataactcctccgtaaaatgctcgtggcctgccaatgaaacgggagagtatacatggccatacctcgggtcgggcctagccaagcccgacgcaaaaaactcaggcccgagccggtcatagggcctattttctgggcccaagcccggcctgaacacgtaaaagcccgttgggcttcgggccggcccggcccgcctttaaggaaaacacaaaaatgacgggcccggcccagcccggccttcaggcttaaaatctaggcccgggagcagcgtcgggtcatgccgggtcgggctttttcgggccgggccgttcgggccgggctgcccatggccacgactacgggagagtcttagatagccaggcagtggcatcttcctccgcatctcagttccacccggcacatactcgcccatgtcaacgcgcacggccgtttctttgacgaaaaagggtttcccccgctttatattataaagcacaaccacaaccacaacaacgagcatccgatacaaacacatgccacactgacccaaggcaagatacatgagtgccgggcaccgacacgcaacctcaacgacacaaagcatctaaaagataagcaaaaaaaggtccgctaagagtcgaccgagaccaccaccaaggagcgatcgtccgggaggatgcgcgccggacacgccggaccgaggactccaagacggtgcctccaggaagggcacgacgaagaacgccgccaccgtctgatccgaagatcaagttttcacccggagtaagatggagggagaagaagcaccacgacggagcctgcaaggaggaacacgacgtccacggacgccgtcaccgtcgaccagaacgggactgggtaggtgatgaaccccggtgcttcagctcctccaccgccaccccggtccaccaacgcccacaaccatgccgctcgagcggccatggttgcctgcccgcattcgagtcgtgcgatccgcccacgaccaacgcgactcccaccgccagggccgccaccccgccatgagacctccccgaggcccccaaaggccacgcctttgtcaagatccacaaccccgaccacctccagaaccgccgccggccaagctgcctcgagaagggccgcgaccacatcgatcgggggaaggggaagacggagaagtggcacatggccacgaccgacacccgtgtcggctccaacttccccccgaaaccgagtttcgcccccgcttcgaaggcccggagcatcaggaggcatcggccgtcgccagaagccagagagggagggttgcggagccgtcgccgtcgccgcctgaagacgccaccaggggatccacccgagccgagcgccaccacccagccgcgagggcccgactaggtgggagcaacccaccacctccgctgccgcgccgcccccccaccagagtaccacggcgagagggccgcccacgacccggtcaaccgcgacccgtggcgccggaggcgaagccgaagcactgccggaagcaccgccgccgactccaccaccggcccgcaccaccaccatgccgcccgccgcacgtcgaacaccaccatcgccggcctgctccggggcgccgccgcgccaccacacccagcaccgagcgccgcgcccagctgcgaaatctggcccacgccatacctcacaagcgggggccgaaggagccccgccgccgccggcgacgcccgggcttcgcccggccgcgccccttggcggcggcgagggagggggagggaggaggaggggggtgcggcggcggggatctggagcactccccggcgcctcgcaggtggcggcgggggaggaagggagggggggattttttttttccgggtcaatcgatttttcagcccacggccgtttctttactccagcacgtagccatcaccggtacggccaccatctacctcgcgcctgataaaacaaagcaaaatctaagttgcagtcagatcagtgccctccctaatcaaacccgaccgcaggttccaatcttttccccgcgggctctcactcggccgatcggtagctgccgcggcggcggtctcggaATGGCGCCGATACGCGTGCTCACGGCGCTGGACCACGCCCGCACGCAGTACTACCACTTCAAGGCCATCATCATCGCCGGCATGGGCCTCTTCACCGACTCCTACGACCTCTTCTGCATCGTGCCCGTCATGAAGATCATCGGGCGGGTGTACTACCCTTCGCCCGCCGGCGACGGGAGGCCGGGCGTGACGCCGCCCGCCGTCGTCTCGGCCACGGTCGGCGTCGCCCTGCTGGGCGCGGTGGTCGGGAACCTCCTCTTCGGCGCGCTCGGCGACCGCGTCGGGCGGCGGCGCGTGTACGGCCCATGCCTGCTGCTGCTGGTGTGCAGCTCCATCGGGAGCGGCTTCTCCATCTGCCGCACGCGCGGGTGCGTGCTCTCCAGCCTCTGCTTCTTCCGCTTCATCCTCGGGGTGGGCGTCGGCGGAGACTACCCGCTGTCGGCGACCATCATGTCGGAGTTCGCCAACAAGCGCACGCGGGGGGCGTTCATCGCCGCCGTGTTCTCCATGCAAGGGTTTGGGATACTGGCCAGCTCCGGCGTCACCATGGTCGTCGCCGCCGCGTTCGACCGGTTCACGGGCCACCCGGACCCGCTTGACACTCCGGAGGCTGCTGACCTTGCCTGGCGCGTCATACTCATGGCCGGCGCCGTCCCTGCCGGGCTCACCTTCTACTGGAGGATGGCCATGCCAGAAACAGCCAG*gttcgtcggtcgtcctaacccccaactggcgcactacgtgtctcttcgaccacgtacgttatatttccctaacgtagttccagtactctgtaggagtgacattcatacagggagttatcatgtacgcatgtacatgtttcggcacattgtcagtacacagtaaaaatttagctatgtctaggacacatctagagttatgtcacatctaaactgctgtccactttgtttgtggtctatctttttttttgtttcatgttgttgcgttatatatttgtgagagattagatgtgacatttttaaaaaaacatctatgtgaattagataaattgtaaaaaaatgctccctctaggcaccttagagattactctaatttccagacgaggcaaaccgtagtctccgcagatttttttagtccatcacattcatgcatttaccatacatgtgactggagtaactttgatcaaaatattgtctgcccacctcacatgaaaacccggatttaagctgatacatcttaaaggggtaaaaaatgaacggttcaagagttcgaggctgcatttggaagttttcaacatttagttccattgcacagtcagtttgcaggtgattttctcgtactgtgtgtctgtttctaagctgtgaacgaagtacctttgaacggccccatggtaagttaataataccagctggtgctgccatgactgttaatcctggattatactatatttttttgagttggcatcctgggttatattcgagtaccatcgtaagacacacgagcgcgagtggcacgccttgcactaaacatttcggcaagtcaccgcattgattggtagactaaactaaactgcgatgatcgatgtgtacactgactggtatctcaaacaaatggcgcgaagaagaatataccgcgggatacttgagatccgaagaagcagcagcttcctgctgaccaacgaaggaattcagtctgttccccgtaaacgaacctgggcacagtttgcctaattattttctctaagccggagcttaactaagtgccaggctctggtcgccatcataaggttgctgcttatacttgtctagttttggtattactatatatcttatcgtcaagttgcctagctccgtttgaccttgcactagcttctagaactagtcgtgcatctggctgtcaagtttaagcatacttgaacaagttgcataagcatacttaaacaagtttgcatacgctgtaaaataagcaagttgagcatatctatatctggatcgatctgcgtctcgttggattggcctcgtcacaccacacgtcaggccggcatatcaaattgaagtggcatataccggtgctgaatctggctcactttagttttcattttgcagttaacaaaccaggtcgatcaggaggtgattcattacaccaaaagataaagtagaaacgtaccgtactactgtagtgtcaaaaaagaaagttaaactagtagtaaccaactaaagttcagtaattgaccgaactaactaactgctccacctgatgggtgctggctggtggtggtgcag*GTTTACGGCGCTGGTACAGCGCGATGTGCTCAAGGCGACCAGCGACATGGGCTGCGTCCTCACGGACCTCGACCTGAACGCGATATCCGAGGGGGAGGACGCGGCGGCCATGCCCCGCACCCCGGCGCCGTTCGGGTACGCGCCGGCGGCTCAGTACGGCCTCTTCTCGCGCGGGTTCCTGCGGGAGCACGGCCGGGACCTGTTCGGGTGCGCGGCGACGTGGTTCCTGCTCGACATCCCCTACTACAGCAGCACCCTGTTCCAGTCCCAGATCTACCGGCCGTGGTTCAAGCCGGCGAGCCACCAGAACGTCTTCCAGGAGGCGTACAACGTGGCGAGGTTCCAGGCCATCATCGCCGTCGCCTCCACCATCCCGGGGTACTTCGCCGCCGTCCTGCTCATCGACCGCACCGGCCGGCGCCGACTGCAGATGGCCGGCTTCTTCCTCATGGCCGCGTTCCTCTTCGCGCTCGCCGGCCCCTACGACCACTACTGGCGCGGCAACGCCAAGAACGCCTGGTACATCGTGCTCTACGCGCTCACCTTCTTCTCCGCCAACCTCGGGCCCAACACCACCACCTTCATCCTGCCGGCCGAGCTCTTCCCGGCGCGGTTCCGGTCCACGTGCCACGGGATATCCGCCGCCGCCGGGAAGGTCGGCGCGCTCGTTGGCTCGGTGGGGTTCCTTTGGGCGTCGCAGTCGCGGGACAGGGGCGATGTGCAGGCCGGGTACGAGCCCGGCATCGGCATGATGTACGCGCTCATCATTCTTGGAGCCATCAGCCTGCTCGGGCTCGTCGTCACCTACTTCTTCACGCCGGAGACGATGAGGCGGTCGCTCGAGGAGAACGAGAGCGAGCGGGATCAGAACCAGGACAGCGACGGCGGGATGTGCTTCCAGGAACTAACTCTGACGCCCAAGAGCCCGGGGTCCTTGGTGAGCTCGCACGTTAGCACGTCGCCTATCCACCCGCACCGCTTTTCTGTATGAtggaactttgaaaacttggcgccacgcataagcagttccgcttttcggttactggcacgtgaaatacagatgtagataatcctacaaaaaaaatacagatgtagatttcccgcatggttgcttcgtttgattggttgagacaaaattacaacatagctcaaactttgtaaaggtctggtcctatgtaactagctatgctattacaggagtatatgtgagttggtgatggtgattgtggtgtcactcaaaacacaaatatgttagtcaggtttttttagggtaaaccacactttattgattgctagaagcacggtttacagggataacaaaaaggttgtggggtaaacccatccacaagtggcgatcaccagctaaagaagaagaaaacttagctaaactg

**TaPht1;10 Chr7DS**

TRIAE_CS42_7DS_TGACv1_622182_AA2034730/ IWGSC WGA v0.4 chromosome 7B scaffold 131382

**partial RT-PCR fragment HG764743 (bold)**

tccgggacattcccttcctgggtctcgccgtcgtccatcgcgcttgtgttctcggcacggcgtggtcaacattgtcaacaaacgacaatatcggaagagggatgtacatggagaggctgaaagttgggacccacatggtccatggccgcacgcaagcaagtatctccttattacgccaaaaagaatgaatactcccaatgacagctcggacccaccagctatatcttcgcacgcaaggaagtgcctccttattacgcacaaaaaaatgaattacaatagccatgtgtactcgcaagtcatcgtgcttttccggcgccgttgccgagaagaatagcacttggtttgaaaaaaatgaatccagttttcgtttgacaatttcggacgaaacaataacaaaagtttcgagagttttaggaaaccgggttttttggtccgaatcgaaagaaatggtaacccaaaaaagtcccttagatagagatgccctactatttctcgattgagaaattagcagatcaattattatttttctgatggctccgtaaaatgctcgtggcccgccaatggagcgggagagtcttagggcgtgtttggttcaagtttaaaacagtagtcgcattgcatacacttctcaacccagcctgggtgagcaaaaacagccccaaatgcgtcatctgcaagttgtttggttgcctgcatcgagggtcgctgcatgaggtaatacccaagtgcactttgtttggttgcctgcattagcccaagtggcacatgaacacagtgtttagttgcccgcatggggtgtgattaagagctagcacttgcacttagcacacagggttaagagctagcagagggagggggagaagaaatgcagtgtgcgccagaccatggcgactgcggtggatagtggatgtggcgccgtgtccgacatgacgagcatggagtcgtcgacgagcgaccccgtcggcggcatggcgagcgacaccgtcgatgacctagttgcggtgctcgcgcaaagacagtcgacagaggaggagaatgtagtgctcgcgccatcccggaccggagcacagcgttggcgagagagacaaatgggggcggggaccaggccgtccgcgccagcggcggctctgcttgccgtcaccgcctactgcgcggtgctcctcgtcgcggccgcaccgccgcagcaccaccagccgcagcagcagcacaagcacgtgaggatcgtcagtgcagtagactgctgtcgaaagagagatgaagctacgatcgtcgaaaccaaaaacttacaacacgaatattgtgaggaactgcgagattaggctactggtcaaaggaaaactcaaacgatcgatcgtgcgcgacgaatctaacaaaattcgtcaagaacagcttcaaacgggaagacgaaattaagaacttacagccgacgaaactacgaaccgatcgcctgaatggaaccgtagcatcgaggtgcatctttttcgtgtggagcttacctcgaatcgaagcaccgttgtgtagatcggaaggggcaaggaagaacagaattagagagaaggttacttgatgtaggagaagataagcacagcgaggcaggccggaggtgctttaagtttcgtgctatacaagttttgtgctatacaggtccaatagtaaaatcagacaaccaaacagcccattcgctttccgcgcgggcctgattgggcttcacacatgcaatcaaacacgcccttacatagcgaggcagtggcatcttcctccgcatctcagttccacccggcacatactcgcccatgtcaacgcgcacggccgtttctttactccagcacgtagccatcaccggtacggccaccatctacctcgcgcctgataaaacaaagcaaaatctaagctgcagtcagatcagtgccctccctaatcaaacccgaccgcaggttccaatcttttccccgcgggctctcgctcggccgatcggtcgctgccgcggcggcggtctcggaATGGCGCCGATACGCGTGCTCACGGCGCTGGACCACGCCCGCACGCAGTACTACCACTTCAAGGCCATCATCATCGCCGGCATGGGCCTCTTCACCGACTCCTACGACCTCTTCTGCATCGTGCCCGTCATGAAGATCATCGGGCGCGTGTACTACCCTTCGCCCGCCGGCGACGGGAGGCCGGGCGTGACGCCGCCCGCCGTCGTCTCGGCCACGGTCGGCGTCGCCCTGCTGGGCGCGGTGGTCGGGAACCTCCTCTTCGGCGCGCTCGGCGACCGCGTCGGGCGCCGGCGCGTCTACGGCCCGTGCCTTCTGCTGCTGGTGTGCAGCTCCGTCGGGAGCGGCTTCTCCATCTGCCGCACGCGCCGCTGCGTGCTCTCCAGCCTCTGCTTCTTCCGCTTCATCCTCGGGGTGGGCGTCGGGGGAGACTACCCGCTGTCCGCGACCATCATGTCGGAGTTCGCCAACAAGCGCACGCGGGGAGCGTTCATCGCCGCCGTGTTCTCCATGCAAGGGTTTGGGATACTGGCCAGCTCCGGCGTCACCATGGTCGTCGCCGCCGCGTTCGACCGGTTCGCGGGCCACCCGGACCCGCTCGACACTCCGGAGGCCGCCGACCTCGCCTGGCGCGTCATACTCATGGCCGGCGCCGTCCCTGCCGGGCTCACCTTCTACTGGAGGATGGCCATGCCAGAAACAGCCAG*gtacgtcggttgtcctaactcccaactggtgcactacgtgtctcttggaccacgtacgttgtactccctccgttcccaagtataagtctttctagagatttcaacaggtgactacatacgaagcaaaatgagtgaatctacacactaaaatatgtctacatgcatccgtatattatattccatttaaaatgtctacaaagacttatatttaggaacggagggagtatttccctaacgtagtactagtactctgtacgagtgacattcatacatggtgtcatcatgtacttccttgtaaattaatataagagcgtttagatcagtactttagtgttttataaatattcttatattagtttacgaagggagtagtacatacgtatgtttcagcacattgtcagtacacagtaaaaatttctccctctaggcaccttagagattgctctaattttcatacaagtcaaaccgtagtctcacgtttgggagtaactttgatcaaaatattgactgtgataaccactgcccacctcacatgaaatcccggatttaagctgatacatctgaaagtggtatcaaatggacagttcaagagttctaggctgcgtttaggaagttttcaacatttagttccattgcacagtcagttaattcgcaggtgattttctcgtactgtgtgtctgtttctaagctgtgaatgaagtacatttgaacggctccatggtaagttaataataccagctgttgctgccatgattgttaatcctgggattatacaatattttttgagttggcatcctgggttatattcgagtacaatcataagacacacgagcgcgagtggcacaccttgcactaaacatttcagcaagtcgtcgcattgattggtagactaaactaaactgcgatgatcgatgtgtacactgactggtatctcaaacaaatggcgcggagaagaatataccgcgggatacttgagatccgaagaagcagcagcttcctgctgaccaacgaaggaattcagtctgttccccgtaggcgaacctgggcgcagtttgcctaattattttctctgagccaaagtttaagtgtactgtcaggctctggtcaccatcataaggttgctggttatacttgcctagttttggtattatatcttatcgtcaagttgcctagctcagtttgaccttgcactagcttctagaactagtcgtgcatctggctgtcaagtttaagcatacttgaacaagttgcataagcatacttaaacagtttgcatacgttgtaaaataagcaagttgagcgtgtctatatctggatcaatctgcatctcgttggattggcttcgtcacaccacacgtcaggccggcatatcaaattcaagtggcatataccggcactgaatctggctcactttagttttctctttgcagttaacaaaccagctcgatcaggataaccaactaaagctaaagtagtaaagtactattgtagtgtcaaaaaagaaagttaaagtagtaaccaactaaagttcagtaattaactgaacaagtactctatctgatgggtgctggtggtgcag*GTTTACGGCGCTGGTACAGCGCGACGTGCTCAAGGCGACCAGCGACATGGGCTGCGTCCTCACCGACCTCGACCTGAACGCGATGTACGAGGGGGAGGACGCGGCGGCCATGCCCCGCACCCCGGCGCCGTTCGGGTACGCGCCGGCCGCTCAGTACGGCCTCTTCTCCCGCGGGTTCCTACGGGAGCACGGCCGGAACCTCTTCGGGTGCGCGGCGACGTGGTTCCTGCTCGACATCCCCTACTACAGCAGCACCCTGTTCCAGTCCCAGATCTACCGGCCGTGGTTCCCGCCGGCGAGCCACCAGAACGTGTTCCAGGAGGCGTACAACGTCGCCAGGTTCCAGGCCATCATCGCAGTCGCCTCCACCATCCCGGGATACTTCGCCGCCGTCCTGCTCATCGACCGCACCGGCCGGCGCCGCCTGCAGATGGCCGGCTTCTTCCTCATGGCCGCGTTCCTCTTCGCGCTCGCGGGCCCGTACGACCACTACTGGCGCGGCAACGCCAAGAACGC**CTGGTACATCGTGCTCTACGCGCTCACCTTCTTCTCCGCCAACCTCGGGCCCAACACCACCACCTTCATCCTGCCGGCCGAGCTCTTCCCGGCGCGGTTCCGGTCCACGTGCCACGGGATATCCGCCGCCGCCGGGAAGGTCGGCGCGCTCGTTGGCTCGGTGGGGTTCCTTTGGGCGTCGCAGTCGCGGGACAGGGGAGATGTGCAGGCCGGGTACGAGCCCGGCATCGGCATGATGTACGCGCTCATCATTCTTGGAGCCATCAGCCTGCTCGGGCTCGTCGTCACCTACTTCTTCACGCCGGAGACGATGAGGCGGTCGCTGGAGGAGAACGAGAGCGAGCGGGACCAGAACCAGGACGGCGACGGCGGGATGTGCTTCCAGGAACTAACTCTGACGCCCAAGAGCCCGGGGTCCTTGGTGAGCTCGCACGTCAGCACCTCGCCCATCCATCCGCACCGCTTTTCGGTATGAtggaactttgaaaacttagcgccacgcataagcagttccg**cttttcggttactggcacgtgaaatacagatgtagataatcctacaaaaaaattacatatgtgctgtctgtcatcttcgtctccggcgtttctcaaaaaaaaaatacatatgtagatttcccgcatggttgcttcgtttgattggttgagataaaattacaacatagctcaaactttgtaaaggtctcgtcgtatgtaactagctatgctattatatatgtgagttggtgatggtgattgtggtgtcacacaaaacacaaatatgttagtcaggtcggtcggtacgagcatcagtgcgcaacatcaagcccatcccacttaaaaatatatatgagttggtgggctgatatttggttgccc

**TaPht1;11 Chr4AS**

TRIAE_CS42_4AL_TGACv1_290006_AA0980290/ IWGSC WGA v0.4 chromosome 4A scaffold 23037-3-1

**partial RT-PCR fragment HG764744 (bold)**

attgagaattcctacaatctgggttgaggaatagtagtacctaatggtagctgcaagtccctatactgagaattaatttgattccaatgccgagttttatttgctttccacgtcagtagttttgctacactatagtagtgataatccaagtggcaatttttttggtgattagatcatcacacagggacaatacgaggagctttcaagcaacaattccagagacagttgtttggccttgggagatgctcaactataaagcacggacacggggacggaaagacggggatacaagatacaacattttccggaaacagccattcagtgatacggtgagtatataaaaaatataaattaaataaaaatatgacatgtaatatagagttaagacagaaaattgatgagaaagagatcataatagagaatactgccccatttggtgtctcttttatcaagtgcttgattgatcctcatccccacatgtcatgttgtccttgcaagctgcatccatcttgcaaaacacatgaaacacaaacagtaaagaagattagaagacaagacataattggaagaaaatacaaacacatgaaaacagttcatgatttatgcctaccaagcatgcctattttgtcatttctttattatttcatgttgagtttgctatggtaatttgtttcacaatgatgatctttgtattctttatgaataaaacatagatttcacagttataatgatatgtaatttctttattatttaatggctagaagttagttttccggacttagtaagtaagcactgtatgttaagagtcagcttactttaaactttgccttagcgctgttattccgaactgaacatcaatattctattaaatatttgaattctaaagcctaggagttcattactaataccatgccctcagaccaaatataattaaccttctttttattgacaaataccgtgctccattgttgtctaccgattaaagttttgtactttgattaccgacaaaatatggctctacttatcagcttatagcaatacctacaattctatcgtaaacatggacgagcgtggcaatgtgcgccatcaacggtatagtactcgttgacagtggcagagcttcggcaaggccgagtgggccgtggcccgcccaggtcaggtttaaaaatattttttgtacggagcgcatcccatgacgatggacagcgatactgaaatacatcagccattacggcatcaagcctcacataatctcaatgctagtgatcgaatggccacgaacttgaccgggactctgctaaatcccagctgactgagtgaggtctagcaaaacagtcttttttatcaccacacacgaccgatgattcagacagttggcctgcaaccgttggaaaactaagtttgacagctggacctccctttgttcttacggtacctacctatcgccgaccagcaaggggggtcgactggttgcggaatatcctgccaaaaagacacatttgtgccttagttaccttcgctcctgccatgtcgaggtgcgttccccagcactagcctccaagtaaatttcacgagagacaaaataagcgcacgcctcgcttcccgccattttctagatgggaacggctgcttgtttattttatttttcgtctttgtcaagggtattattctgtcgtgtatgtttgtcccggtgtgttaaacttgtagaagtatgcccgtgtgtgttcaatcggtgagaaatgtatgatccgagtagtatgcggaatatgctgccagccaaccagcacgaggcagacggaggctacgagcgatgaggacagttttcccttgttgccgccgtcctctgcatcatcgtcataatctccggccggagcacagttataagaaccagggtgagcaagcagcaaagcaccaaattaacacgggagttgagttgagagttgagacacggtctcatagtctcataaaagcgccctttatcgaaaaaaagtcggaccgggATGGCGGAGAATGGGGCGGGCGGCGGCGGAGGGGGGCAGAACCTCGCGGTGCTGGACGCGCTCGACTCGGCGCGCACCCAGATGTACCACATGAAGGCCATCGTCATCGCCGGCATGGGCTTCTTCACCGACGCCTACGACCTCTTCTGCATCACCACCGTCTCCAAGCTGCTCGGCCGCCTCTACTACCCGGACAGCAACGCCGACATCGGCAAACCCGGCACCATGCCCGTGCGCATCAACAACATGGTCACGGGGGTCGCGCTCGTCGGCACACTCATGGGCCAGCTCGTCTTCGGCTACTTCGGGGACAAGCTCGGCCGCAAGCGCGTCTACGGGATCACGCTCGTCCTCATGGCCGTGTGCGCCATCGGCTCCGGCCTCTCCTTCGGGAGCTCTGCCAGCGCCGTCATCGGCACGCTCTGCTTCTTCCGCTTCTGGCTCGGCTTCGGCATCGGCGGGGACTACCCGCTGTCCGCCACAATCATGTCCGAGTACGCCAACAAGAAGACCCGCGGCGCCTTCATCGCCGCCGTCTTCGCCATGCAGGGCGTCGGCATCATCTTCGCGGGCCTCGTGTCCATGATCGTCTCCGCCATTTTCCTCCACTACAACCCTGCACCGGCGTGGGATGCGCACCATGGCCTGACGGTTGATGGTCAAATGGATCAGTGGCCCGGGGCGGACTACATGTGGCGCGTCGTGCTCATGCTCGGGGCGTTCCCCGCGGTGGCCACCTTCTACTGGCGGATGAAGATGCCCGAGACCGCCAGGTACACCGCTCTCATCGAGGGTAACGCCAAGCAGGCTACCAACGACATGCAGAAAGTGCTGGAGATCCGCATCGACGAGGAGCAGGAGAAGCTCTCCAAGTTTAGGGCGGCCAACGAATACTCTCTGCTGTCCATGGAGTTTGCGCGGCGTCACGGCTTGCACCTCATCGGCACCACCACCACGTGGTTCCTCCTCGACATCGCCTTCTACAGCCAGAACCTGACACAAAAGGACATCTTCCCAGCCATCAACCTCACCGGCACCCCGGGCTCCATGAACGCGCTCAAAGAGGTGTTCGTGATATCACGAGCCATGTTCCTCATCGCCCTCTTCGGCACTTTCCCCGGTTACTGG**GTCACCGTGGCACTCATCGACAAGATGGGAAG***gtatgtactccctccggtccttttaagtcagcatataagttttgttcgaagtcaacgtatctctacattcatcgaacttatagaaaaaagtatcaatattcacaaagccaaatcaatatagttttccatagtgtatatatttgatattatagatgttcatagtttttaaatataaatttgatgaaactttgcaaagtttgactggacaaaatcctaatatgcggagtaaaaaaggccggagggagtacgtacctatatgcatgcatggctcatgcgcgatcgacttggttagtttcgttacacctacacattaatttgagcatccatgaattgaatgcag***GTACTTGATCCAGCTCCTTGGTTTCTTCATGATGTCCCTGTTCATGCTAGTGATGGGCATCAAATACGAATACCTCAAGGACAAGGGCCACGCCCTGTTCGCCATCCTCTACGCGCTCACATTCTTCTTCGCAAACTTCGGCCCCAACAGCACCACCTTCGTGCTGCCGGCCGAGCTATTCCCCACGCGTGTCCGCTCTACATGCCACGCCATCAGCGCCGCGTCAGGCAAGGCAGGTGCCATCGTTGCCGCCTTCGGGGTGCAGACCCTCACCCTCAAGGGCGACCCCAAGCACATGAAGCAGGCGCTCATCCTTCTCTCTGTCACCAACATGTTCGGCTTCTTCTTCACCTTCCTCGTCCCTGAGACGATGGGCCGATCGCTCGAAGAGATCTCCGGCGAGGACGGCAACGTTGCCGGCGCCGCCGCTGGGCATGTGGACAAGGACGTCG**AAAAGGCCCCTCCTTCAAGCACCGAATGGCAGCCACCATCGTCCATGAATTAAgtaatgcatgagaacacgcacacatgcacgcacgcaagcgtaataatatcatcttatcttctgctatatatatatatatatatatatatatatatatatatatatatatatatatatcagctagcgaattggaaaggggacaagaataataataacggcaaccagctgcatgctacttaagttgccaggtggcacttggtttgagagtttgttgttttatctacacgtgtaatattattgcactaggattgcttgtgttgtgtgtgataccatccattgtgttgttaaggttatattgaatttcagtgtggatgtatgagcattgctactttataatcatgtcaagtttacagaattaaataaccatgtcaaatatgtgctgtttttgtttgcgggatag

**TaPht1.11 Chr4BS**

TRIAE_CS42_4BS_TGACv1_329038_AA1097260/ IWGSC WGA v0.4 chromosome 4B scaffold 46890

agttctgctgaaaacagcgtcagtccgggttagtttcattcaaatcatgcaagttagagtccaaaacaagggcaaaagtgtttggcaaagtagatacgttggagacgtatcaaccaacagctgcgcgacacgtggcacctctatgccgacggggagagttcctatgggctgacctaacacaaccgggggggagttccactggtcaaagcccgtctgtgttaagtcaaagggctagatctcgtggtcaaacgctacagggttaggcgggacggggccctggaggggtagcaatgccaccggagcgttgcactggcccctcatatatgctgggtggtgtggtggcatgtctgaagaggcggcacgcgtctccggggtgtggcccccctcacggacggcgacgccgccggtacctggaggggcgaaacggacgcgtcgggtctacacggaagggaccttgctatgggtttggccgggatgttgctccaaagtgttcttatgggctgacctaacacaactggggggagagttccactggtcaaagcccgtccgtgtaaagacaaagggctagatctcgtggtcaaacgctacaaggttaggcgggacgggggcctgggggcggtagcaatgacatcggagcgtcgcaccgggccctcacacatgcggggtggtgtggtggtatgtccgaagaggcggcacgcgccttcaggttgtggcccccctccaagtgacgggggcactgccttgcgtggaggggggccacaccgcggagccccaagatgggcgtctacgcatgcctaaacactttcacacatgcatcaggacccgtgcgatgtttcagtggcatagctacaccccgaatcccctccactaaccagattccctctgtgtcgatcgtttcccctttccgtgacacggcgggagcgacgtccgtgaggggggcaatcgatataccatcggggtatgttttctgtttagataaggcacatttatcttgtggaaaaataaaaaataaaaaataaaataaagtaaaagaaaaacataataaaataaaatgtatgcccacggcagggccgtcggcataggtgcgcacacggacgtacaatcccacggatcgatgacgtggcaccgcacgcgggtagatgacatggcgcgcccaggtcaggagcaaaatattttttatacggagcacatcccatgacgatggacagcgatacagaaatacatcagccatcacggcatcaagcctcacacaatctcaacgcatttgatcgaatggccatgaacttgaccgggactctgctaaatctcagctgaccgactaacgttaagcaaaacagtcttttttattaccacacacgaccgatgattcagacagttggcctgcaaccgttggaaaactaagtttgacagctggacctccctttgttcttacgggactcctatacctgcctatcgccgacgagcaaggggggtcgactggttgcggaatatcctgccaaaaagacacatttgtgccttagttaccttcgctcctgccatgtcgaggtgcgttccccagcactagcttccaagtaaatttcacgagcgagacaaaataagcacacgcctcgcttcccgccattttctagatgggaacggctgcttgtttattttatttttcgtctttgtcaagggtattattctgtcgtgtatgtttgtcccggtgtattaaccttgtacaagtatgcccgtgtgtgtgcaatgggtgagaaatgtatgatccgagtcgtatgcggaatatgctgccagccaaccagcacgaggcagacggaggctacgagcgacgacgacagttttccctcgttaccgccgtcctctgcttcatcgtcataatctccggccggagcacagttataagaaccatggcgagcaagcagcaaagcaccaaccacgggagttgagttgagagttgagacacggtctcataaaagcgccctttatcgaaaaaagttaggaccgagATGGCGGAGAATGGGGCGGGCGGCGGCGGAGGGGGGCAGAACCTCGCGGTGCTGGACGCGCTGGACTCGGCGCGCACCCAGATGTACCACATGAAGGCCATCGTCATCGCCGGCATGGGCTTCTTCACCGACGCCTACGACCTCTTCTGCATCACCACCGTCTCCAAGCTGCTCGGCCGCCTCTACTACCCGGACAGCAACGCCGACATCGGCAAACCCGGCACCATGCCCGTGCGCATCAACAACATGGTCACGGGGGTCGCGCTCGTCGGCACCCTCATGGGCCAGCTCGTCTTCGGCTACTTCGGGGACAAGCTCGGCCGCAAGCGCGTCTACGGGATCACGCTCGTCCTCATGGCCGTCTGCGCCATCGGCTCCGGCCTCTCCTTCGGGAGCTCCGCCAGCGCCGTCATCGGCACGCTCTGCTTCTTCCGCTTCTGGCTCGGCTTCGGCATCGGCGGGGACTACCCGCTCTCCGCCACAATCATGTCCGAGTACGCCAACAAGAAGACCCGCGGCGCCTTCATCGCCGCCGTCTTCGCCATGCAGGGGGTCGGAATCATCTTCGCCGGCCTCGTGTCCATGATCGTCTCCGCCATTTTCCTCCACTACAACCCTGCACCGGCGTGGAATGTGCACCATGGCTGGACAGTTGATCATCAGATTGAGCAGTGGCCCGGGGCGGACTACATGTGGCGCGTCGTGCTCATGCTCGGGGCATTCCCCGCGTTGGCCACCTTCTACTGGCGGATGAAGATGCCCGAGACCGCCAGGTACACCGCTCTCATCGAGGGTAACGCCAAGCAGGCTACCAACGACATGCAGAAAGTGCTGGAGATCCGCATCGACGAGGAGCAGGAGAAGCTCTCAAAGTTCAGGGCAGCCAACGAATACTCTCTGCTGTCCATGGAGTTCGCGCGGCGTCACGGTTTGCACCTCATCGGCACCACCACCACGTGGTTCCTCCTCGACATCGCCTTCTACAGCCAGAACCTCACACAAAAGGACATCTTCCCAGCCATCAACCTCACCGGTCCCCCGGGCACCATGAGTGCCCTCAAAGAGGTGTTCGTGATATCACGAGCCATGTTCCTCATCGCCCTCTTCGGTACTTTCCCAGGCTACTGGGTCACCGTGGCCCTCATCGACAAGATGGGAAG*gtatgtactccctccggtcctttttagtcagcatataagtttttgtctgatatcaaagtatctctactttcatcgaacttacagaaaaaaaattatcaacattcacaaagccaaataaatatttttatgttcattttgaaatgaagttttatagtgtatatatttatttattatagatattgatattttttttaacataaatttggtacgaagtttgactggacaaaaatctagtatgcggggtaaaaagggcaggagggagtacgtacctatatgcatgcatggctcatgcgagaccgaattggttagttttgttacacctacacattaatttgagcatccatgaattactgcag*GTACTTGATCCAGCTCCTTGGTTTCTTCATGATGTCCTTGTTCATGCTAGTGATGGGCATCAAATACGAATACCTCAAGGACAAGGGCCACGCCCTATTCGCCATCCTGTACGCGCTCACATTCTTCTTCGCAAACTTCGGGCCCAACAGCACCACCTTCGTGCTGCCGGCCGAGCTATTCCCCACGCGTGTCCGCTCTACATGCCACGCCATCAGCGCCGCGTCGGGCAAGGCAGGCGCCATCGTCGCCGCCTTCGGGGTGCAGACCCTCACCCTCAAGGGCGACCCCAAGCACATGAAGCAGGCGCTCATTCTCCTCTCTGTCACCAACATGTTCGGCTTCTTCTTCACCTTCCTCGTCCCCGAGACGATGGGCCGATCGCTCGAAGAGATCTCCGGCGAGGACGGCAACGTTGCCGGCGCGGCCGCTGGGCATGTGGACAAGGATGTCGAGAAGGCCCCTCCTTCAAGCACCGAATGGCAGCCACCATCGTCCATGAATTAAtgcatgacaccacgcatcttatcttctatatcagggaatttagaaagggggcaagaataacggcgaccagctgcatgctacttaagttgccaggcacttggtttgagagtttgttttatctacacgtataatattgcaataggattgcttgtgtgtgataccatccattgtgttgttaaggttatattgagttttcagtgtggatgtatgagcattgactagggatttcccttctcaatcatcaatatgtattgctatacttataataataatgcatgtcaagtttaatgaatttttttaaggggtaaccaagtttacagaattaaataccatgcacgtcgagtaggcatgcatatatgtgcactgcgttttttttcttgtcgtacagagttaatttgta

**TaPht1.11 Chr4DS**

TRIAE_CS42_4DS_TGACv1_362305_AA1178710/ IWGSC WGA v0.4 chromosome 4D scaffold 52428

ccaagtccatatactgagaattacttttttgttccttgcaagtccctatattgagaattcctataatctgggttgaggaatagtagtacctaatggtagctgcaagtccctatactgagaattaatttgattccaatgccgagttttatttgctttccacgtcagtagttttgctacactatagtagtgataatccccgtggcaattattttggtgattagatcatcacacagggacaatacgaggagctttcaagcaacaattcgagagacagtctgtgtttggccttgggagatgctcaactataaagcacagacacggggacggagagacggggatacaggatacaaaatttcagaaacagccattcagggatacggtgagtatatatgaaatataaattaaataaaaatatgccatgtaatatagagttaagacagaaaattaattagaaagagatcataatagagaatactgccccatttggtgtctcttttatcaagtgcttgattgatcctcatccccacatgtcatgttgtcattgcaagctacatccatcttgcaaaacacatgaaacacaaacagtaaagaagattagaaggcaagacataattggaagacaatacaaacacatgaaaacaattcatgattttttgtctttattatttcatgttgagtttgctatggtaatgtgtttcacaatgatctttgtattctttatgaataaaacatagatttcacagttattaatgatatgtaatttctttattatttaatggctagaagttagttttctggacttagtaagtactctatgttaagagtcagcttactttaaactttgccttagcgctgttattccgaactgaacatcaatattctattaaatatttgaattctaaagactgaagcctgacctaggagtccattactaataccatgtccttagaccaaataaaattgaccttctttttattgaccaataccatgctccactgttgtctactgattatagttttgtactttgattactgacaaaatatggctctacttaaattctattgtaaaacgcaacgcgcgtcatcaacgatctagtactcgttgacagtggcggagcttcgacaaggccgagtgggccgtggcccgcccaggtcaggagcaaaaatatttttgatacggagcacatcccatgacgatggacagcgatactgaaatacatcagccattacggcatcaagcctcacataatctcaatgctagtgatcgaatggccatgaacttgaccgggactctgctaaatctcagctgactgactgagtgaggtctaggaaaacagtcttttttattaccacacacgaccgatgattcagacagttggcctgcaaccgttggaaaactaagtttgacagctggacctccctttgttcttacggtacctacctatcgccgaccagaaaggggggttcgactggttgcggaatatcctgccaaaaagacacatttgtgccttagttaccttcgctcctgccatgtcgaggtgcgttccccagcactagcctccaagtaaatttcacgagagagacaaaataagcacatgcctcgcttcccgccattttctagatgggaacggctgcttgtttatttttcgtctttgtcaagggtattattctgtcgtgtatgtttgtcccggtgtattaaacttgtagaagtatgcccgtgtgtgttcaattggtaagaaatgtatgatccgagtagtatgcggaatatgctgccagccaaccagcacgaggcagacggagcctacgagcgatgaggacagttttcccttgttgccgccgtcctctccttcatcgtcattatctccggccggagcacagttataagaaccatggcgagcaagcagcaaagcacaaacacacggaagttgagttgagagttgagacacagtctcatagtctcataaaagcgccctttatcggaaaaaaaaagttgggaccgggATGGCGGAGAATGGGGCGGGCGGCGGAGGGGGGCAGAACCTGGCGGTGCTGGACGCGCTGGACTCGGCGCGCACCCAGATGTACCACATGAAGGCCATCGTCATCGCCGGCATGGGCTTCTTCACCGACGCCTACGACCTCTTCTGCATCACAACCGTCTCCAAGCTGCTGGGCCGCCTCTACTACCCGGACAGCAACGCCGACATCGGCAAACCCGGCACCATGCCCGTGCGCATCAACAACATGGTCACAGGGGTCGCGCTCGTCGGCACACTCATGGGCCAGCTCGTCTTCGGCTACTTCGGGGACAAGCTCGGCCGCAAGCGCGTCTACGGGATCACGCTCGTCCTCATGGCCGTGTGCGCCATCGGCTCCGGCCTCTCCTTCGGGAGCTCCGCCAGCGCCGTCATCGGCACGCTCTGCTTCTTCCGCTTCTGGCTCGGCTTCGGCATCGGCGGGGACTACCCGCTGTCCGCCACAATCATGTCCGAGTACGCCAACAAGAAGACCCGCGGCGCCTTCATCGCCGCCGTCTTCGCCATGCAGGGCGTCGGCATCATCTTCGCGGGCCTCGTGTCCATGATCGTCTCCGCCATTTTCCTCCACTACAACCCTGCACCGGCGTGGGATGCGCACCATGGCCGGACGGCTGATGGTCAAATGGATCAGTGGCCCGGGGCGGACTACATGTGGCGCGTCGTGCTCATGCTCGGGGCGTTCCCCGCGGTGGCCACCTTCTACTGGCGGATGAAGATGCCCGAGACCGCCAGGTACACCGCTCTCATCGAGGGTAACGCCAAGCAGGCTACCAACGACATGCAGAAAGTGCTGGAGATCCGCATCGACGAGGAGCAGGAGAAGCTCTCCAAGTTTAGGGCGGCCAACGAATACTCTCTGCTGTCCATGGAGTTTGCGCGGCGTCACGGCTTGCACCTCATCGGCACCACCACCACGTGGTTCCTCCTCGACATCGCCTTCTACAGCCAGAACCTGACACAAAAGGACATCTTCCCAGCCATCAACCTCACCGGTCCCCCGGGCACCATGAGCGCCCTCAAAGAGGTGTTCGTGATATCACGAGCCATGTTCCTCATCGCCCTCTTCGGTACTTTCCCAGGCTACTGGGTCACCGTGGCACTCATCGACAAGATGGGAAG*gtacgtacctatatcatattatgaacaaaaaatataaaattgtgaacaatttttttgtctatcgagtaaaatagtcattatccctcaatcatgctttacaaataagctcacatttttcctagatcagaaatgggcacatgcaatctttataaaaaaattaggattgaaaaaaaatgacacgttcatacaaacgaaagaagattcaatgtttggtttttaggcctaaaacagacattcttttgtactaataatttagtggcaacaagaggtcaaacatggccagtttggctatagtttgactctcctcccggaagcaccttgtgctatttgtagctgtccgtatgtacataggttctgtctaaagtagtagtgaaaattttaaagtttttggagggggctggcttcagcctattgaagtctgccgtctgcctactagacacgccaccatctatatgtacgcatgcataactcatgcacacacagtccatgcgcgaccgacttggttggttttgttacaccctacacatttgagcatctatgaattgactgcatgcag*GTACTTGATCCAGCTTCTTGGTTTCTTCATGATGTCCCTGTTCATGCTAGTGATGGGCATCAAATACGAATACCTCAAGGACCACGGCCACGCCCTGTTCGCCATCCTGTACGCGCTCACATTCTTCTTCGCAAACTTCGGCCCCAACAGCACCACCTTCGTGCTGCCGGCCGAGCTATTCCCCACGCGTGTCCGCTCTACATGCCACGCCATCAGCGCCGCGTCAGGCAAGGCAGGCGCCATCGTCGCGGCCTTCGGGGTGCAGACCCTCACCCTCAAGGGCGACCCCAAGCACATGAAGCAGGCGCTCATTCTCCTCTCTGTCACCAACATGTTCGGCTTCTTCTTCACCTTCCTCGTCCCCGAGACGATGGGCCGATCGCTCGAAGAGATCTCCGGCGAGGACGGCAACGTTGCCGGCGCGGCCGCTGGGCATGTGGACAAGGATGTCGAGAAGGCCCCTCCTTCAAGCACCGAATGGCAGCCACCATCGTCCATGAATTAAtgcatgacaagttgacaacacacgcacaagcacgtgtgcggaataatatcatcttatcttctgctatactatcagctagcgaattggaaaggggacaagaataataacggcaaccagctgcatgcatgctacttaagttgccaggcacttggtttgagagtttgttgttttatctacacgtgtaatattattgcactaggattgcttgtgttgtgtgtgataccatccattgtgttgttaaggttatattgaattttcagtgtgaatgtacgagcattgactaaggatcgatttcccttctcaatcgtcaatatgtattgctatacttataataatgcatgtcaagtttaatgaatttttttaggggtaaccaagtttacagaattaaataaccatgcat

**TaPht1;12 Chr2AS**

TRIAE_CS42_2AS_TGACv1_112554_AA0340780, IWGSC WGA v0.4 chromosome 2A scaffold 909; truncated gene with putative additional intron or insertion, early stop-codon and missing 3’ part

gaaatggatctggtaaccgagaaaaataactgatactaaagtgtacctagagaaaaaaaactctaagtaagaatcaatctaaactaacaaaagagtttcctatttccctcaggtttatcctctttgctacacattggaaatatacgcagtcggcactttatattagcttccgaaggtgtagatttttcgggtctcaagatgcagtctattagaggcaatagcaccggttatgttcaatagatttggatggctgtttagcaatatgatttgagctgcataggtgtacgtagcattcgattcaagttacggttgtcacatccttatttgatgaaccttttattttatatccacattttaaagacttgatatgttgcaataaaaaagggttgcgatgtatatgaaccgatgtagataggccaggagcttctcttaattcacggtcatttgtcatatacatggcgatacaacaatatgctccttgaagtttccttcatacactcgcacatagggtaactaagcgtggaggctataagaaagtgtagcttttatatacagctttggatcagtatcaacaaacacgccagcgcctgcaactttttcaccatttcatcggtgcagtgcaaatgccatggcggcagccaacacaggcactgATGGCACGGAAGCAGCTCAAGGTCCTCCATGCCCTCGACATTGCGACGACGCAGGTGTACCACTTCACCGCCATCGCAATCGCCGGCATGGGCTTCTTCACTGATGCCTACGACCTCTTCTCCATCTCCCTCGTCACCGACCTCCTCT*gttggcccaaaccagacgccaagtactttctggcgacggcgacgaccgacgacgaacgagatgacaaacaaagctcgttgatgaactcgatgaattgcttttacgccgagacgcaccgcagtacaaagctaatcttccagcaatcgatctatctgtatgagtatgtagtactagtacgtacgagcacttctagccggaatcacaacttgatcgagctatctcatgtagatagcgcaacacacaagaatcgattgggattgccacacgcatgtatcatgcgtttctttattgctttggttctcacgatgtgttttacaaggccggggtagacacatatatatagtagtacaagttagctaccaccaatactactcggtttgctaattagagttctactagaatacacgcacgcatacgagtcagacacacaccggcgaccgggggaattagtcgggtttaattccaacatcctcggcgcatct*ACGGCGTCCTCCCTGTCGGCGTCTCGGCGCTTGTTAATGGTGTTGCACTATGCGGCACCGTGGTTGGGCAGCTCTTCTTCGGATGGCTTGGCGACAAGGTGGGCCGGCGGCACATCTATGGTGTCACCCTGAAGCTCATGGTCATCTGCTCCATCGCCTCCGGCCTCTCCTTCCACCATTCGCGCAAGAGCGTCATTACCACGCTCTGCTTTTTTCGTTTTTGGCTTGGCTTCGGTATCGGTGGCGACTATCCACTCTCTGCTACGATCATGGCGGAGTACGCAAATAAGAAGACTCGTGGCGCCTTCATTGCCGCTGTATTCGCCATGCAG*gtgcaaccatacaacacgattgtgtcttaatttttggttgtgtgaatcaatcaatctccattttgcaaaaaatatcccttgattacatttttatttgtctgttgtcttgtaattgcag*GGTCTAGGAAATCTTGCTGCTGGAATAGTTGCCATAATCGTCTCACAGTCGTTCAGGCATGCACCAGGATATGACCATGACCCTCACTGGCACGCTGACTATGCGTGGCGTATCATTCTCATGGTAGGCGCCATCCCTGCTATCCTGACTTATTATTGGCGCATGAGGATGCCTGAGACGGCACGCTTTACGGCGCTCATAGCTAAGGACATCAAGAAAGCTTCTTCAGACATGGCCTTGGTCCTTAACATCGACATTGTGGCTGAAAAATAAGAGGCTGATGTGTTCAACAGAGAGCATGAGTTTGGTTTCTTCACCATGGAGTTCCTCCATCGACACGGCCTCCACCTCCTTAGcacctccacgcgctttggaccatggagtttgataagaaacctataaaaaacccaaagaagttcgatcagaaaccaatcagaaagataaaccaagaatagaaaaacctagccaaaagtttccgctatgtgacatgagattgagagatctattatttgtgtccactgcataccattgtccgtacgtatgttcaactggcgacgagtgtggcgcatgaccgcgtcgctgccgagaggccggtccgtccggacatgcggacgtagacgtagacgtccgtcaagattgctacggggctgcgtgcgtgctgtggcatgcagtgtgcgtgcagtgtgatgtatcccttcgtctcaaaataaatatcttaattttgtattaactttagtacaaagttgtattaaggtcaagacatttattttgatatggagggagtatataatacggtgcaagtccctggacacaacaaagtgtgtgctcaactacggcggcagctg

**TaPht1;12 Chr2BS**

TRIAE_CS42_2BS_TGACv1_145945_AA0450350/ IWGSC WGA v0.4 chromosome 2B scaffold 21713

aaggaggatgacccccggcctctacatctgggcgatgcatacggccactttattaattattctcacaagactttacaaagtcatacaacagtaagacaaaagccgccatttaagcaacaaagtgtcgctacacctatccagttgatgaacgggcgcagatagcctgagcctagtaccaaacagacatcacagccaagcctaacatctaagacctgagaccccaacctagccacttgccgggtctagggcacacactggtccggcgtgctctcagaggccgccgccgccaacttccaccgctccatcttcagaactgtactgatgcatcaaccttactcggtccagctattgtcgacgccaccacggcgcccaacggcacctcctccctgcgcgcaaacagctgaacacgtcgcggtcgccactaatacacctcagcgccatgctgccaagtaccaccagccgacaccgcttgacgcccttggaggatatgtcgtgcgtagcacctgccgaccaggcatgaccaagcgtagcacctgtcggtcaggcatgacttgacatctccaccgaagctccgtgcaagatgaagccgctccacctcctgcctctgacttccagcgctgctccacaaaacgatgctcccaagagaaaaacgacaccgcaatgccgccatcgtccggtctggaacaccagatcctagggtttcccccggagcaatacgagtgggtcgatggtagtcacatgacgatgccttcgtcaaggtaatgacatggaacgccgccattgcccgcttcggctcggttttcaccggcaactacatcttcccgactcgcagctggtgccagatgatggatctcgagatccgaacacccagcctcaggccgatcacctctgacggaagagatgaccaccaccaccggctgcgccgctcagaacagatctgatcggaggtgccgccgacgaaaccaccaggccctccacgccgccgtcgccgatctgaaggcagcatgcaggccaccgcagccaagccggccgccgccgaccgcagccgccgccgccacccgaagggccatccgcaacgcctgcagccctgacctccgccgcgccaagctgtcgccgaccgaggatggaccggcctcccgccgcctgcagccatccgccgcgccgcagatcccagatcggtcgcgccaccacatgccttggggtccgccccacaccggagacgcgcgagagaggagatcccccgccaccgccgtcgcccggcggcgtcttccggcggcggcggagggagaggaggagggagtttgcgccggcggctgctaggtttctggccgcccgagtcgccctagggggaggacgacggggggctgagatgagtttcctgatacatcatgttgcaataaaaaatggttgcggtttatattaaccgatgtagataggccatgagcttcttattctcgaaaaaaagaaaaaaagaatttgaaattaatcataaaatagtctggatatatagggtatacagctatggtacatgaagcacatgcatttactttgatcaatggtgttcaacttttgttcattcattaattcagctttgagaatgtacaacatatccggaggaattgttagattccacgaagctacgtccaaattccctctgtatcatccatgttcgactgtatattcctcatgtaaatcacacgtagtaacaaccaattcaccgtttctcacatcaggggacggctataccaagatacgggatcatgcttacttcacggtaatttgtcccatacatggcgatcgacacaacaatatgctccttgaagtttccttgagaagctcgtgcatagagtaactaggcgtagaggctttaagaaagtgtggcttttatatacacttttggatcaaaatgtctcagtatgaacaaacacgccaacacctacaacctttttcaccatttccttcctgcagtgcaaatgccatggcggcggccgacacaggcaatgATGGCACGGAAGCAGCTCAAGGTGCTCCATGCCCTCGACGTGGCCACGACCCAGGTGTACCACTTCACCGCCATCGCGATCGCCGGCATGGGCTTCTTCACTGACGCCTATGACCTCTTCTCCATCTCCCTCGTCACCGACCTCCTCGGCCGTATCTACTATGAGGACGGTGTCCTCCCTGTCGGCGTCTCGGCGCTTGTTAACGGTGTTGCACTATGCGGCATGGTGGTCGGGCAGCTCTTCTTTGGATGGCTCGGCGACAAGGTGGGCCGGCGGCACATCTATGGTGTCACCCTGAAGCTCATGGTCATCTGCTCCATTGCCTCCGGCCTCTCCTTCCACCATTCACGCAAGAGCGTCATTACCGCGCTCTGCTTTTTTCGTTTTTGGCTTGGCTTTGGTATCGGTGGCGACTATCCACTCTCTGCTACGATCATGGCGGAGTATGCAAATAAGAAGACTCGTGGCGCATTCATTGCCGCTGTATTCGCCATGCAG*gtgcaaccatacaacacgattgtgtcttaatttttggttgtgtgactcaatcaatctccattttgcaaaaaagatcccttcattatatttttatttgtctgttgtcttgtaattgcag*GGTCTAGGAAATCTTGCTGCTGGAATAGTTGCCATAATCGTCTCACAGTCATTCAGGCATGCACCAGGATATGACCATCACCCTCACTGGCACGCTGACTATGTGTGGCGTATTATTCTCATGGTAGGCGCCATCCCTGCTATCCTGACTTATTATTGGCGCATGAGGATGCCCGAGACGGCACGCTTTACGGCGCTCATAGCTAAGGATATCAAGAAAGCTTCTTCAGACATGGCCTTGGTCCTTAACATCGACATCGTGGCTGAAAAAGAAGAGGCTGATGTGTTCAACTGAGAGCATGAGTTTGGTTTCTTCACCATGGAGTTCCTCCATCGACATGGCCTCCATCTCCTTAGCACCATGATCTGTTGGTTTATGCTTGACATGTCATTTTACCTGCTCAACCTGTTCATGAAGAACATCTTTACCGAAGTTCAATACATCAAAGATGCAAGCACAATGAGCCCGCTTGAGCAAACATACAAGATAGCAAGAACCCAAGCTCTCATTACCGTCATTGGCACGTTGCCGGGCTTCTTCTTTGCAATTAAGTTCATGGACAGAATTGGTCGAATCAAGATGCAAATTGCAGGATTCATTATGATGAGTGTCTTCATGCTCGGGCTTGCTATTCCACAAGTGTTATCGAAAACGATATGGGATTCTCGCTATGGGAACATCTACTTCATTGTCATTTACTCGGCGATAATGTTCTTCACTGACTTCGGACCCAACACGACCACTTTCATCCTTCCAGCAGAGATCTTCCCAGCACGTATGCGGTCAACATGCCAGGGCATAGCCGGTGCTGGCGGGAAGGGTGGTGCTATCACTGGTGTGCTTTGGTTCCTATATACAAATAAAGGTCTCCCAATTATTCTCTTCGTGCTAGTTGGTTGCAACATAATTGGCTTGGTGTTCACGCTCATCTTACCAGAAACCAAAAAGAGGTCTCTTGAAGAGGTCACTGGTGAAAGAGGAAATGATGAGGACCAGGGAGGTTTTTCTCTTGTGAGAACACCTCTATTTACTATATAGtgccaaagaatataccatgtggccctttgatgtaaaacgaaaacatgcaagtagctatttgttcttcatagtagaagtccattaattagtactccctccatagcaaaaagcttgtcctaaatttgtctagatatggatatatctagacatgttttagtgttagatacatttgtatctagataaatctaagacaagatttttgggacggaagtaatagttaatgagattgttggaattctggaaaccacaagtacagaacgatatgactatcaaaaccttttcctaataagcataaataaaaagcatctttgtcttttttagcaataggccaaatcccaataagcataaataaaaagcatcttctatcaatacaaacacacagaatccccatgcgggtctc

**TaPht1;12 Chr2DS**

TRIAE_CS42_2DS_TGACv1_177711_AA0582920/ IWGSC WGA v0.4 chromosome 2D scaffold 42730

atgcaatgcaagattagagaaatggatctggtaaccgagaaaaataactgatactaaagtatacctagagaaaaaaactctaagtaagaatcaatctaaatttacaaaaaagtttcctatttccctcaggtttatcctcttcgctacacattggaaatatacacagtcggcactttatattagcttccgaaggtgtagaattttcgggtctcaagatgcactctattagaggcaatagcacaggttatgttcaatagatttggatggctgtttagcaatatgatttgagctgcataggtgtagcattcgattcaagttacggttgtcacatctttgatgaaccttttattttatattcacattttaaagacttgatatgttgcaataaaaaatggttgcgatgtatatgaaccgatgtagataggccaggagcttctcttattctcgaaaaaaagaaaaagaattagaaattaatcataaaatagtctggatatatagggtgtacagctatggtacatgaagcacaggcgtttactttaatcaatggtgttcaacttttgttcattcgttaattcagctttgataatgtacaacatatccggaggaattgttagattccacgaagctacctccaaattccatctctctatcatatatgttccaccgtatattcctcatgcaaatcacacatagtaacaaccgattcaccatttttcgcatcaggggacggctataccaagatactggatcatgcttagttcacggtcatttgtcatatacatggcgatacaacaatatgctccttgaagtttccttcatacactcgcacatagggtaactaagcgtggaggctataagaaagtgtggcttttatatacagctttggatcagtatcaacaaacacgccagcgcctgcaactttttcaccatttcatcggtgcagtgcaaatgccatggcggcagccaacacaggcattgATGGCACGGAAGCAGCTCAAGGTGCTCCATGCCCTCGACATTGCGACGACGCAGGTGTACCACTTCACCGCCATCGCAATCGCCGGCATGGGCTTCTTCACTGATGCCTACGACCTCTTCTCCATTTCCCTCGTCACCGACCTCCTCGGCCGCATTTACTACACGGACGGTGTCCTCCCTGTCGGCGTCTCGGCGCTTGTCAACGGTGTTGCACTATGCGGCACGGTGGTCGGGCAGCTCTTCTTCGGATGGCTCGGCGACAAGGTGGGCCGGCGGCACATCTATGGTGTCACCCTGAAGCTCATGGTCATCTGCTCCATCGCCTCCGGCCTCTCCTTCCACCGTTCACGCAAGAGCGTCATTACCACGCTCTGTTTTTTTCGTTTTTGGCTTGGCTTTGGCATCGGTGGCGACTATCCACTCTCCGCTACGATCATGGCGGAGTACGCAAATAAGAAGACTCGTGGCGCCTTCATTGCCGCTGTATTCGCCATGCAG*gtgcaaccatacaacacgattgtgtcttaatttttggttgtgtaaatcaatcaatctccattttgcaaaaaagatcccttgattatatttttatttctctgttgtcttgtaattgcag*GGTCTAGGAAATCTTGCTGCTGGAATAGTTGCCATAATCGTCTCACAGTCATTCAAGCATGCACCAGGATATGACCATGACCCACACTGGCACGCTGACTATGTGTGGCGTATAATTCTCATGGTAGGCGCCATCCCTGCTATCCTGACTTATTATTGGCGCATGAGGATGCCCGAGACGGCACGCTTTACGGCGCTCATAGCTAAGGACATCAAGAAAGCTTCTTCAAACATGGCCTTGGTCCTTAACATCGACATCGTGGCTGAAATAGAAGAGGCTGATGTGTTCAACAGAGAGCATGAGTTTGGTTTCTTCACCATGGAGTTCGTCCATCGACATGGCCTCCACCTCCTTAGCACCATGATCTGTTGGTTTATGCTTGACATGTCATTTTACCTGCTCAACCTGTTCATGAAGAACATCTTTACCGAAGTTCGATTCATCAAAGATGCAAGCACAATGAGCCCGCTTGACCAAACATACAACATAGCAAGAACCCAAGCTCTCATTACCGTCATTGGCACGTTGCCGGGCTTCTTCTTTGCAGTCAAGTTCATGGACAGAATTGGTCGAATCAAGATGCAAATTGTAGGATTCATTATGATGAGCGTCTTCATGCTCGGGCTTGCTATTCCACAAGTGTTATCGAAAACGATATGGTATTCTCGCTACGGGAACATCTACTTCATTGTCATTTACTCGGCAATAATGTTCTTCACTGACTTCGGCCCCAACTCGACCACTTTCATCCTTCCAGCAGAGATCTTCCCAGCACGTATGCGGTCAACATGCCACGGCATAGCCGGTGCTGGCGGGAAGGGTGGTGCTATCACTGGTGTGCTTTGGTTCCTATATGCCAATAAAGGTCTCCCAATTATTCTCTTCGTGCTAGTTGGTTGCAACATAATTGGCTTGGTGTTCACGCTCATCTTACCAGAAACCAAAAAGAGGTCCCTTGAAGAGGTCACTGGTGAAAGAGGAAATGATGAGGACCAGGGAGGTTTTTCTCTTGTGAGAACACCTCTATTTACTATATAGtgccaaagaatataccatgtggccctttgatgtaaaacgaaaacatgcaagtagctatttgttcttcacagtagaagtccattaattagtactccctccattgcaaaaagcttgaccaagatttgtctagatacggatatatctagacatgttttagaattagatacatttgtatctagataaatctaagacaaaatttttgggacgtaggtaataattaatgagattgttggaattctggaaaccacaagtataaaacgatatgactatctaaaccttttcctaatttaaagacattataactgcgcatctttcgagccctctcaggccattaggctccacagccgcccgatcatcacttgcaggtgtttctggccgtcagattagctcttaatcccac

**TaPht1;13 Chr2AS**

TRIAE_CS42_2AS_TGACv1_112554_AA0340810/ IWGSC WGA v0.4 chromosome 2A scaffold 909

**partial RT-PCR fragment HG764745 (bold)**

gacaaatctagtccggctagtcactgtcccagaagtaagaacaaacaggccagaggatgactttcgagactgacgactttgactcttcgattccatgtcaatctggcaaggaatatgccccccggagctcagaaaatgcctttttctcagctcatagtttagtatttagtcaagtctttgcctctactatgcgtccatgcatgactcacacacatgagcccagaaaagtttgaagaacaaaagggctaaggtggatgatctgccttgtgaataacatgccgacaaatttgtggctgcattttttttaccgcaagacacattaattagacgacttgacaattcacagattaattacagaaaatcgggctaaaaccagtacaaccacacaaaccgtgaaggaacatgaaaatatcagggcacagctggccgacctaccacacaagagatatagcatgcacgccgttgatgagagaatcatggcggaggtagcagacaatcatcgtcgtaagtcactcctctctgagcaagcgactctgacattgccactagcaaccaccccctcacaacaatggccgcgcgatgccaaactaagaacacccaaacaaccgggccagacacgtcgaggacgaggggcaactcggacttcaatgacgagcctcacggaagaaagttgcaaaccttgggctggagatcgtcgagcactgaaacaccctttcacaaggaacccgcaagatccccgaattcaccactacacaccaccatcgccgccacacatgagcgtatcctggactaccgtgcaaatataatttagtaaaattagtacgtgggtgtagcaaaactcgatgtgattgtacgatgcgcatgtatgtagtacgtacgtgttgtgtgtatgtgtagtcgcagcaaatgaaacggtgagggtgtgaatattcgtggtccacgtgcacgcaggtttgttggtagaaatggcgatccgcgtgtgcgaatcctagtcaattagcaggcgagacaaggaaccgagacagcgttagcaagcagaggcaaagctgctatttggtgcagcgctggtccgatccgttgacagagaattttattttctttacggttagctcggacttcactcaacttgaggtatactttctcaactctcaagtctcagcagcaaattaaagtgagacacgtggaatatcaatcgaagaatacacgcgtttcaaaacgaggaagatgctgctgccgaacactcctggcctgtatatatatatatatgccacccaagcgcttctctcgcccgatcgatgtattcagttcttcagtttttgctactgcctaggaaaccaaggtcagggaaaccgggtagcatcaggtatgtacctatttattggcttttgctgtcttttttgaacaaaccattttctgctccatatatgcatgcatcatcctcacatgcatactactggttttccatctacccaactcaaatatcggtcttaattttagttctaattttcctgccaaaaatgacccagatatattatatatgaagttcatcaaaactacaaagcaccaaaaacataacaaaaattgcatcacggtccttaaaacgatggcgcgactgctgtcatcacaataacgagccgccggcgtttgctgtcactgaggcagaccatgtcgataatagccgagaaattttcgtgcaaagcacgagcgccatcaaggctggaggcatgttccgcttatcgtttagtttaatagtagttttgacgtagacatgcatgccaagttcttccaaactgttctgttcgaatcactctatcgtagacgtttggtccacgtagaggtcgctcgagaagttcgatcaacgagtgtgtcgctatccagaactccacatgcttatctatgtgataacctccgaaaatcaagtgatctgacattgacatctcaggtagtcacgagcctacacgtacgtgcctcaggcctttgactaacacgagccaaacgccATGGCTCGAGAGCTCAAGGTGCTCGGCGCGCTGGATGCCGCCAAGACGCAGTGGTACCACTTCACGGCCATCGTGATCGCCGGCATGGGCTTCTTCACCGACGCCTATGACCTCTTCTCCATCTCCCTCGTCACCAAGCTGCTGGGCCGCATCTACTACTTCGACCCGAGCTCAGCCGCCCCGGGCTCGCTCCCGCCCAACGTCTCGGCCGCTGTCAACGGCGTCGCCTTCTGCGGTACCCTCGCCGGCCAGCTCTTCTTCGGATGGCTCGGCGACAAGATGGGCCGGAAGAAGGTCTACGGCATGACGCTCATGATCATGGTGCTCTGCTGCGTCGCCTCCGGCCTCTCCTTCGGCTCCACTCCGAACTCCGTCATGGCCACGCTCTGCTTCTTCCGCTTCTGGCTCGGCTTCGGCATCGGCGGCGACTACCCGCTCTCCGCCACCATCATGTCTGAGTATGCCAACAAGCGCACCCGTGGCGCCTTCATCGCCGCCGTATTCGCCATGCAGGGGTTCGGCAACCTCACCGGCGGCGTCGTCGCGATCATCGTCTCGGCTGCGTTCAAGGAGCGGTTCGACGCGCCCGCGTACAGGGACGACCGGGCCGGCTCCACCGTCCCGCAGGCAGACTACGTGTGGCGCATCGTCCTCATGTTTGGCGCTGTCCCGGCGCTGCTCACCTACTACTGGCGCATGAAGATGCCCGAGACGGCGCGCTACACGGCGCTCGTCGCCAAGAACGCCAAGCTGGCCACGTCCGACATGGCGCGAGTGCTCAACGTCGAGCTCGTCTCCGACGAGCCGGAGCAGCCGCTGCCCGTCGGACATGGTGACCGCGAGCAGTTCGGGCTCTTCTCCAAGGAGTTCGTTCGGCGCCACGGCCGGCACCTGCTGGGCACGACGGTGTGCTGGTTCGTCCTCGACATCGCCTTCTACTCGCAGAACCTGTTCCAGAAGGACATCTACACGGCGGTGGAGTGGCTGCCGAGGGCGGACACCATGAACGCGCTGCAGGAGATGTTCAAGATCTCGCGCGCGCAGACGCTGGTCGCGCTGTGCGGCACCATCCCGGGGTACTGGTTC**ACGGTGTTCCTCATCGACGTCGTCGGCCGCTTCGCCATCCAGCTCGGCGGCTTCTTCTTCATGACGGCCTTCATGCTGGGCCTCGCCGTGCCGTACCACCACTGGACGACCCCCGGGAACCACGTGGGCTTCGTCGTCATGTTCTCGCTCACCTTCTTCTTCGCCAACTTCGGGCCCAACTCCACCACCTTCATCGTGCCGGCAGAGATCTTCCCGGCACGGCTGCGGTCGACGTGCCACGGCATCTCGGCGGCTGCCGGGAAGGCGGGCGCCATCGTGGGGTCGTTCGGGTTCCTGTATGCTGCCCAGAGCACTGATTCCACGAAGACGGACGCCGGGTACCCGCCGGGCATCGGCGTCCGCAACTCGCTGTTCGTGCTCGCCGGGTGCAACGTGGTCGGGTTCCTGTTCACGTTCCTCGTGCCGGAGCCCAACGGGAAGTCGCTGGAGGAGCTCTCCGGTGAGAACGAGGTGGATGACGCGCCTGAAGATGCCTCATCTGCGGCAGCCGGGGAGGACAGGAGCACGCCGGCACCAGACGTTTGAtagaagttacagaatatgtttgcgtaatggca**ttatcacttgctgcatcggctgtcaaagcattgggttggagtgcagtttcttttactctgattgcaggatcttgttggaagcagtggaagaaaagaatctactggtaaaatatggacattggaagatcagaccggtgcttgcagattttctcaactccttcagtgttagccatattcctagatctcaaaatcctatttctcacaatctagcaaagaaagctttctctatcagatctttccttacttgctcttttctatgtagcaaggcttctagatgtaaattcaatatggctttgaaaaccatatcaaaactactgtatgtacactgtttaggctgttaatgaataaaatgtgagccctctatgagg

**TaPht1;14 Chr4AL**

TRIAE_CS42_U_TGACv1_641255_AA2089900/ IWGSC WGA v0.4 chromosome 4A scaffold 11991

cctcctacagataaggtgtgctttatagaaaaattgaagtatgagtgattgaagacggaatcatgcctttatgggtaatttcaatcttctaggcatgtgacatagcagaagtacacggaatcatgcctttataaattgcaacttgacgaggcacatccttcctcatagcaaccgcaacaaccactctctgttctatcaaggaaaaaaagtagcacccattctaagaatctggccacttcaattccttaaaaattacattgttatccacctatgcttgttgctacttcttctattcagacatgttagcacatcagataggtgaaattctaagatgtattggaaacacaagggaatgggagacatcaagagagaagcactttcctcagaaagaacattgatggagtcgttccggcgaatcttgggtagggggtcccaagctagtactctagataacatagacgcgaggttttacctaggttcggactctttcaaagagataataccctacgtcctgctttgattgtgtattgattttggatgaagtacatagtacatgtgtgtccaccacgagattgttgatgatcttccacaagctctacccctagtttatatagatattgtggggtctagggttacagataggtcggctacgtatacggtagatcgacgtgtagaagatatctaagtctcgtattatgcgccaagtcttcggaacattccttccatacgccatgggccttcaggacatggcccactagtacaccgcaataggagtccttggcccgagccaccttgccgggagacggcgtggtgggtaccccctggtctgggacaccaccaaacatggtcatgaatttccaatagccgccgtcatgctatgtcgttgggttcggtgcttgtaactctaaatgcatattcatatgctctagattcaagttctggctataatatgtgaaatgtgaaatgaatgccttacataaatcacgagtccatgatcgaataatggaggattctagaaatttcccaaagcccagaatacaatggcgatcgacaaacttgaacagaaccaccctaaagataaactttgtgcatggagtaggggcggcgcggcggcagcgtggctaggtttactgcccggttggccacgcgtggctcctttcataacaagcatcggggtaagatgagtatatattatcggattcccgtcttacatatatgagacccatgggttgttaatagacactcggcctacagtggccgctgtcacagagctttcaaggggtgggaggacagccacgtggcggctgcttatgccgagccccttgcataagccgagtgcttttctcacattatgccaagatttgtgtagaaacaaagttctttttgtgatgtactcggcctgtgtgaagataacccgagccacagccggttgccatcggtttttttcagagcactcggcttatggtgtactacgccgggtggcagataatttacacttgatttacgcgtttaaacactaggcaacatgtatttttcccgtagcggtacatgtttataagtgtttgcgtatgtaacgtgtttttaaaaaagtatccatttaatgcgacattacacgtcaacaaaaaatatcctttattctatcaagaatgactaatcttatcttgttatttccgtccttgtgttgtttcagtacggaacagctgtcgtcgtcttcattctatcgccgtgttccttttttattagaaagcgtaggcacattcgtcagcagagctgccaattttatgtattctctcggagaaagtaaggtggatcgatgttattcagctgggggctggggcgccctctcgtaattacattccacaggacattcgtgcagttgtataataggagcagagacaacatttgaatcgccactttatatatccagttggcttgcagaaatcagaaagaggttatatatatggtaaacatgaagcacttggtttactataccacgcacaATGTTCACGACCTGGCCTGCGAGGAGGCACGCGTGCAGCTCCCTCTTCTGCCACCTGCATGGCGCCGGCAGCGCCATGCTGTACCGCGTGCTGGACGCCGTGACGTCGGTGAAATGCGAGACGCGGCGGGCTCGCAAGCAGATCAAGGTGCTGCAGGCCCTCGACGTCGCCGGGACGCAGCTGTACCACTTCACCACCATCGTCATCGCCGGCATGGGCTTTTTCACGGACGCCTACGACCTGTTCTCCGTCTCCCTGATCGCCGACCTCCTGGGCCACATCTACTACCACTCGGCAGACGGCAAGCTCCCCGGCAATGTCGCGGGCGCTGTCAGCGGCGTGGCGCTCTGCGGCACGGTCCTGGGGCAACTCTTCTTCGGCTGGCTCGGCGACAGGATGGGGCGGAAGCGGATCTACGGCGTCACGCTCAAGCTCATGGTGGTGTGCTCACTCGCGTCCGGCCTCTCCTTCCACAACAAGCCCAAGTGCGTCGTGGCCACGCTGTGCTTCTTCCGCTTCTGGCTTGGCTTCGGCGTCGGCGGCGACTACCCGCTCTCGGCGACCATCATGTCCGAGTATGCCAACAAGAGGACTCGCGGAGCCTTCATAGCAGCTGTCTTCGCTATGCAG*gtgtgtaagtaaaatcatcacaatattttttggagccagtgctgcatctcattcttaatctacaatctgcttctcttttctctggtctaactcagcaaaagataatattttaatcatccttaccctattatactttgtctgcataaag*GGTCTTGGAAACCTCGCTGCTGGGGCTGTTGTTCTGGTGCTCTCTGCGAGGTTCAAGAACACGGCCACGTATGAGACTGACCAGCTTGGGCAAGCGGACTACGTGTGGCGCATTGTGCTCATGCTGGGCGCCGTTCCTGCACTCCTCACCTACTACTGGCGCATGAAGATGCCTGAGACGGCGCGCTACACGGCGCTCATCGCCAAGAACCTCAAGCTAGCAGCATCCGACATGGCCACGGTCCTTGACATCGACTTCGTGTCAGACGCGGACGCGGACGCCATCGTGAAGCAGGACGAGTTCGGCCTCTTCTCCATGGAGTTCCTTCACAAGCATGGTCGCCAGCTCCTCTGCACGACTACATGTTGGTTCGTCCTCGACGTTGTCTTCTACTCTCTCAATCTCTTCATGAAGGATATCTTCAACAACATCGGCTGGTTTGGAGACGCGACCATGATGAGCCCTCTCGAGCAGACCTATAAGATAGCTCGCACGCAGGCCATCATCGTGGTCGGCGGTTCCCTGCCAGGTTACTTTCTCACCGTGCTATTCGTCGACCGCATCGGCCGCATCAAGATCCAACTCATGGGGTTCACCATGATGACCATTTTCATGATCGTACTCGCCGCGCCTTACAAGTTTTGGTCCAAACCCAACATGCACATAGGCTTCGCCATCATGTACGCCTTGATCCTCTTCTTCGCAAACTTCGGCCCCAACTCCACCACATTCATCCTGCCCACGGAGATATTCCCGACGCGCCTGCGGTCGACGTGCAACGGCATATCGGCCGCCGGGGGAAAGTGTGGTGCAATCATCGGTGTTCTCTGGTTCCAGTATTCTCATACAAGCATCCGGAGCTCTCTGCTTCTGCTGGCAGGGTGCAACCTGGTTGGAGTCATGTTCACTCTTGCCTTGCCCGAGTCCAAAGGGATGTCACTCGAGGACATCACGGGGGAAATGGAGGAAGACAACGAACCACCAGAAGAATCTAAAACGGTTGCTGAAGCTGAGTTCATCTACAGCGTGGAAATTTCGTAAccaatactgtccctccccgttgatggttttagtgtcttctcgaaattcatcaaactttgagaacctgtatctttgacatgtttggacttatatatagtgtttattgtgaaatggggctgaacaatgcatatctatatctatatctatctatacctaccaataaagcaaggtgtgtttctccaattttttcatccgttcgtaaggtgcgtttctccaattttttcattcgttcgtaaggtgcgtttcttcaattttttcatccattcatccatattattttttagatttttttagtatccgaggtggtactattttttgcacacgcacgtccaaaataataatgacaactggcctcaacccatccgaagcacacagcccatgctctgtccccgatgatgtc

**TaPht1;14 Chr1BL**

TRIAE_CS42_1BL_TGACv1_030553_AA0093860/ TGACv1_scaffold_030553_1BL

Incomplete, 28 bp deletion leading to early stop codon.

NNNNNNNNNNNNNNNNNNNNNNNNNNNNNNNNNNNNNNNNNNNNNNNNNNNNNNNNNNNNNNNNNNNNNNNNNNNNNNNNNNNNNNNNNNNNNNNNNNNNNNNNNNNNNNNNNNNNNNNNNNNNNNNNNNNNNNNNNNNNNNNNNNNNNNNNNNNNNNNNNNNNNNNNNNNNNNNAGACTACGTGTGGCGCATAGTACTCATGCTCGGCGCCGTTCCTGCCCTGCTCACCTACTACTGGCGCATGAAGATGCCCGAGACGGCGCGCTACACCGCGCTCATCGCCAAGAACCTCAAGCTAGCGGCGTCTGACATGGCCGCGGTCCTCGACATCGACTTCGTGTCCGACATGGATGCGGAGGCCGTCGTTAAGCAGGACGAGTTTGGCCTCTTCTCCATGGAGTTCCTTCACAAGCATGGCCGCCAGCTCCTCGGAACCACCGTGTGCTGGTTCGTCCTCGACGTCGTCTTCTACTCCCTCAACCTCTTCATGAAGGACATCTTCAGCGGCATCGGCTGGTTTGGAGACGCGGCTGAGATGAGCCCTCTCGAGCAGACCTACAAGATAGCCCGCACGCAGGCCATCATCGTGGTCGGCGGTTCCCTACCAGGGTACTTCCTCACTGTCCTCTTCGTTGACCGCATCAGCCGCATCAAGATCCAGCTCATGGGGTTTACCGTGATGACCATCTTCATGACCGGGCTCGCCGCGCCCTACAAGTTCTGGTCCAAACCCAGCATGCATGCAGGCTTCGCCATCATGTATGCATTGATCCTCTTCTTCGCAAACTTTGGCCCTAACTCCACCACCTTCATCCTACCCACCGAGATATTCCCGACGCAGCTGCCGGGGGTAAGTGTGGTGCCATCATCGGTGTTCTCTGGTTCCAGTATTCTCATACGAGCATCCGGAGCTCTCTCCTTCTTCTAGCAGGGTGCAACCTGGTTGGAGTCATGTTCACTCTTGCCTTGCCGGAATCCAAAGGGATGTCACTCGAGGATATCACCGGAGAAATGGAGGAAGAAAGTGAACCATCTCAAGAATCTGCAACGATTGCTGAAGTTGAGTTCATCCACAGCGTGGAAATTTTGTAAccagtaccattcatcccggttgctgattttagtgtcttctcaaagttactcaaacattggaaacttgtatctttgaaatgtttggattatatagtcttcaatgtgacatgggatgggatagactccaaacgaagtggcaataacaaaactaaatctaggcatagcgaatagtagagcaactttcagttttatcccgtcctttactcctatccatattacttttcgctggttcagtacaactgtattacagaattgtactagagcagcgacaagaaatccctatgagcctacaagatattgaacccaggaccagggaggttgtgagcacgttgttagccactagaaccaaccagatttggtgatacatatgtagcacagtttcttatcaacaagaatcgatttactgtagcagaccaaaccaccactgtacgaccgggcgggtcacg

**TaPht1;14 Chr7DS**

TRIAE_CS42_7DS_TGACv1_621820_AA2026930/ IWGSC WGA v0.4 chromosome 7D scaffold 25190-1

taactaaaaattatttaaaacaacaattaaaggactaaaacaactacataagcaaaacagtaatgttttaattaaaatcctaatttaatattttctaaaaataagcaaataaattcttactgtgacaacaatctaacatgtgactaggagcaaaaaacctaaattaaaaaatatttttaaactcaaattattcacaatctagggtttgatgcaaatttggaaaaatcatatttaaatcattcaaatttgaatactaatggcacaaacagaaactagagaaaattttgaatctaatgcaaaaacaatcactcaagaatatcaaatatcctaaaagatataagcaatataaacagaaactaaaaacaaaaaacaaatggtaaaacataaaacaaatttaagaacccctttagtcccggttcgtggctccaaccgggaccgggactacctttagtcctggttcgagccacgaaccgggactaaagggcatcgcaccctttagttccggttggagccacgaaccaggactaaaggtccaaaggtagtcctggttcgagccacgaaccgggactaacgggcatcgcgccctttagtctcggttggaggcatgaaccgggactaatgtctgcccggcgccctagccgctcgaaccgggactaatgctcacattcgtcccggttcgtaatgcaaccaggactaatgtgtagattgagattggaccaaagccctgttttctactagtgatacgccatgggccttcaggagatggcccattggtaaaccgcaacgagggtccttggcccgagccaccttgccgggagatgacgtgctgggtaccctctggtccgggacaccatcaaacatggtcatgaatttccaatagccgtcatcgagctatgtcgttgggttcggtgcttgtaacgctaaatgcatattcacatatgctatagattcattgcaagtttcggctataatattttatagaaataatatgatgaaggttgaaatgtgaaatgaatgccttacataaatcatgagtcaatgatcaaataatggaggattttagaaatttctcaaagcccagaatccaatggcgatcgacaaacttgaacagaaccaccccaaagataaacttgatgcatggagtaggggaggcgccgcggcagcatggctaggtttacagcccggtcaaccacgtgtggctccttttattacaagcgtcggggtaagcagagtatacattatcggaaactcggcttacatatatgagacccatgggttgtcaatagacactcgcctacagtggccactgtcacagagatgtcaaggggtgggaggacggccatgtggcggctgcttatgccgagccccttgcataagccgagtgcttttctcacagtatggcaagatttgtttacaaacaaagttctttttgtgttgtactcggcctgtgtgaagataagccgagccacatccggttgtcatgtgtttttttcagagcactcggcttagggtgcactatgcctggtggccgacaatttacacttgatttacggttaaacactaggcaacatgtgtttttcccatagtggtatgtgtttatgagtgtcctcctatgtaacatgtttccaaaaaagtatattttcaatgcgacgttacgtgtcaataaaaaaatccttttgttctatcaacaatgactaatcttatcttgttgtttccgtccttctgttgtttcagtaaggaacagctgtcgtcgtcttcattctataaccgtcttccttctttagaaagcgtaggcacgttcgtcagcaaagctgccaattttatgtgttcttcggacaaagtaaggtggatgctatgcagctgggggctggggcgccctcgtaatcatactatttcacaggacattacgtgcagttgtataataagagcagagacaacatttgaatcgccactttatatatccagtaaacatgaagcacttggtttactaccacgctacaATGTTCACGACCTGGCCTGCGAGGAGGCACGCGTGCAGCTCCCTCTTCTGCCACCTCCATGGCGCCGGCAGCGCCATGCTGTACCGCGTGCTGGACGCGGTGACGTCGGTGAAATGCGAGACGCGGCGGGCTCGCAAGCAGATCAAGGTGCTCGAGGCCCTCGACGTCGCCGGGACGCAGCTGTACCACTTCACCACCATCGTCATCGCCGGCATGGGCTTCTTCACCGACGCCTACGACCTGTTCTCGGTCTCCCTCATCGCCGACCTCCTGGGCCGCATCTACTACCACTCGGCGGACGGCAAGCTTCCCGGCAAGGTCGCGGGCGCCGTTAGCGGCGTGGCGCTCTGTGGCACCGTCCTGGGGCAGCTCTTCTTCGGCTGGCTCGGCGACAGGATGGGGCGGAAGCGGATCTACGGCGTCACGCTCAAGCTTATGGTGGTGTGCTCGCTCGCGTCCGGCCTCTCCTTCCACAACAAGCCCAAGTGTGTGGTAGCCACGTTGTGCTTCTTCCGCTTCTGGCTCGGCTTCGGCATCGGCGGCGACTACCCGCTCTCCGCAACCATCATGTCTGAGTATGCCAACAAGAGGACTCGGGGAGCTTTCATAGCAGCGGTCTTCGCTATGCAG*gtgtgtaagtaatactttttttttgagggaacggtgtgtaagtaatactattacaatgttcatttgaaaatcctagacttacggacaacaagtcacggagggagcatacgggattacctgtccacctgctaatctcccttcacctcctgattgaagtgtggaccagaccatctgattttcaatcaacagtttccatgtcagcctgtaggctctagtccgtgagtttgagtctcaatcatgacatataacatagaaatggaaagaaaaaataaaaataaaagtttacatgaatctccatgcaatattcaagtctactgagatttagcagcactacaacaaaaaaaacaatatttcagtccttaccatattatacttgctctgtataaag*GGTCTCGGGAACCTCGCTGCTGGGGCTGTCGTTCTGGTGCTCTCTGCGAGATTCAAGAACACGGCCGCGTACGAGACTGACCAGCTTGGGCAAGCGGACTACGTGTGGCGCATTGTACTCATGCTCGGCGCCGTTCCCGCCCTCCTCACCTACTACTGGCGCATGAAGATGCCCGAGACGGCGCGGTACACGGCGCTAATCGCCAAGAACCTCAAGCTAGCGGCGTCCGACATGGCCGCGGTCCTCGACATCGACTTCGTGTCGGAGGCGGACGCGGACGTTGTCCAGCAGGACGAGTTTGGCCTCTTCTCCATGGAGTTCCTTCACAAGCATGGCCGCCAGCTCCTCGGCACCACCGTCTGCTGGTTCGTCCTCGACGTCGTCTTCTACTCCCTCAATCTCTTCATGAAGGACATCTTCAACAACATCGGCTGGTTTGGAGACGCGGCCATGATGAGCCCTCTCGAGCAGACCTACAAGATAGCTCGCACGCAGGCCATCATCGTGGTCTGCGGTTCCCTGCCAGGGTACTTCCTCACCGTCCTCTTCATCGACCGCATCGGCCGCATCAAGATCCAGCTCATGGGGTTCACCATGATGACCATCTTCATGATCGGGCTCGCCGCGCCCTACAAGTTCTGGTCCAAACCAAGCATGCACGTAGGCTTCGCCATCATGTACGCCTTGATCCTCTTCTTCGCAAACTTCGGGCCCAACTCCACCACCTTTATCCTGCCCACGGAGATATTCCCGACGCGGCTGCGGTCGACGTGCAACGGCATATCGGCCGCCGGGGGTAAGTGTGGTGCCATCATCGGAGTTCTCTGGTTCCAGTATTCTCACACGAGCAACCGGAGCTCTCTTCTTCTGCTGGCGGGGTGCAACCTAATTGGAGTTATGTTCACTCTTGCCTTGCCGGAGTCCAAAGGGATGTCACTCGAGGATATCACCGGGGAAATGGAGGAAGAAAGCGAACCACCTGGAGAATCTGCAATGATTGCTGAAGCTGAGTTCATCCACAGCGTAGAAATTTCGTAAccagtgacgtccatcctggcccgttgctgatcttggtgtctactcagcaaaattactcaaacattgagaacttgtatctttgagatgtttggttatatatagcgttcactgtgacatgagatgggatatactccaagcaaagtggtaataagacaactaaaattaggcatcgcaaataatagagcaaacttttgttctaccccaagaatggtcgggccgatcatcatcccaagaattgcatagccgcaccacttactaagggacgcagcttcagtgccgtaaaggcacctgcttttgggtccctgacatgtgggccagccacctgttgggtccacatgtcataggcacaaaggcaggtgccttaaggcaccgtagggcagtcccttcctaagggccgctt
